# Conjectures and refutations: Species diversity and phylogeny of *Australoheros* from coastal rivers of southern South America (Teleostei: Cichlidae)

**DOI:** 10.1371/journal.pone.0261027

**Published:** 2022-12-09

**Authors:** Carlos A. Santos de Lucena, Sven Kullander, Michael Norén, Bárbara Calegari

**Affiliations:** 1 Laboratório de Ictiologia, Museu de Ciências e Tecnologia da Pontifícia Universidade Católica dRio Grande do Sul, Porto Alegre, Rio Grande do Sul, Brazil; 2 Department of Zoology, Swedish Museum of Natural History, Stockholm, Sweden; 3 Department of Vertebrate Zoology, National Museum of Natural History, Smithsonian Institution, Washington, DC, United States of America; Pontificia Universidade Catolica do Rio Grande do Sul, BRAZIL

## Abstract

Morphological and genetic analyses of species of *Australoheros* focusing on those distributed in coastal rivers from the Rio de La Plata north to the Rio Buranhém, support recognition of 17 valid species in the genus. Eight species are represented in coastal rivers: *A acaroides*, *A*. *facetus*, *A*. *ipatinguensis*, *A*. *oblongus*, *A*. *ribeirae*, and *A*. *sanguineus* are validated from earlier descriptions. *Australoheros mboapari* is a new species from the Rio Taquari in the Rio Jacuí drainage. *Australoheros ricani* is a new species from the upper Rio Jacuí. Specimens from the Rio Yaguarón and Rio Tacuary, affluents of Laguna Merín, and tributaries of the Rio Negro, tributary of the Rio Uruguay are assigned to *A*. *minuano* pending critical data on specimens from the type locality of *A*. *minuano*. *Australoheros taura* is a junior synonym of *A*. *acaroides*. *Australoheros autrani*, *A*. *saquarema*, *A*. *capixaba*, *A*. *macaensis*, *A*. *perdi*, and *A*. *muriae* are junior synonyms of *A*. *ipatinguensis*. *Heros autochthon*, *A*. *mattosi*, *A*. *macacuensis*, *A*. *montanus*, *A*. *tavaresi*, *A*. *paraibae*, and *A*. *barbosae*, are junior synonyms of *A*. *oblongus*. *Heros jenynsii* is a junior synonym of *A*. *facetus*.

## Introduction

About 530 species of the freshwater fish family Cichlidae have been recorded from the Neotropical region, over 400 of them in South America, the remaining in Middle America. The majority of the South American cichlid species pertain to the Amazon and Orinoco River basins and other tropical rivers. Three genera, however, are exclusive to more southern rivers, including the Rio Paraná basin, and coastal rivers from the La Plata basin north to the Rio São Francisco. Among those, *Gymnogeophagus* Miranda Riberio, 1918, with 18 species, is restricted to the lower Paraná, Paraguay, and Uruguay drainages, with a few species also occurring in the Laguna dos Patos basin. ‘*Geophagus’ brasiliensis* (Quoy & Gaimard, 1824) represents a group of species in need of revision, distributed along the coast from the Laguna Merín drainage north to the Rio Coruripe at 9°S, but also present in the upper Rio Uruguay and some tributaries of the Rio Paraná [1, 2]. The third genus, subject of this paper, is *Australoheros* Říčan & Kullander, 2008. It comprises 31 nominal species with a combined distribution in rivers and lagoons along the South American Atlantic coast from tributaries of the lower Rio Paraná and the Rio de La Plata north to the Rio Buranhém at about 16°S, but also present in the Uruguay River basin and some tributaries of the major rivers Paraná and São Francisco. Most of the information about *Australoheros* is contained in the two seminal works by Říčan and Kullander from 2006 [3] and 2008 [4], describing the genus and most of its southern species, and outlining the intrageneric phylogeny. It was followed by several short species descriptions and re-descriptions chiefly by Ottoni and collaborators, 2008–2013 [5–12], focusing on species from the northern part of the distribution of the genus, but also Říčan et al. in 2011 [13].

*Australoheros* comprises medium-sized species with lengths only exceptionally exceeding 100 mm standard length (SL); the largest specimens recorded in the present study were about 140 mm SL. Ringuelet et al. [14] reported specimens of *Australoheros facetus* Jenyns, 1842, with 161–162 mm SL at age 10 years, and larger specimens almost 180 mm SL that were not aged. Species of *Australoheros* were predominantly recorded from small streams or vegetated lentic habitats. Limited observations on food in various species suggest a diet based on small fish and benthic invertebrates such as insect larvae, shrimps, and small crustaceans, supplemented with some vegetable matter [15–19]. Based on an analysis of swimming mode in *A*. *facetus*, Gómez et al. [20] (concluded that *A*. *facetus* may be defined as ‘a specialist in maneuvering in structurally complex habitats, with certain capacity of acceleration to capture invertebrates and small fishes’. The majority of the species of *Australoheros* shares a general body shape, moderately deep and laterally compressed, with short head and caudal peduncle, blunt snout, small mouth with short teeth except for enlarged anterior teeth in the upper jaw, and a long anal fin with 5–8 spines. All species share a colour pattern dominated by dark vertical bars across the sides. Although some localities are close to the sea, there are no records of *Australoheros* from saline waters. *Australoheros facetus* was recorded as feral in rivers in the Iberian Peninsula in Europe [21–23] and in Chile [24]. Laboratory observations showed that *A*. *facetus* is a territorial, biparental substrate brooder, commonly noted for its aggressive behaviour [25]. Similar behaviour was reported for other species of the genus based on aquarium observations [26, 27]. Reliable information on fecundity has not been reported; 300–1000 eggs per spawning was reported for *A*. *facetus* in aquarium [28]. The combination of territoriality, aggressivity, parental brood care, and generalist feeding were highlighted as factors facilitating invasive progress of *A*. *facetus* at least in the Mediterranean region [17, 18].

The taxonomic history of *Australoheros* started in 1842 with Jenyns’s [29] description of *Chromis facetus* collected by Charles Darwin in Maldonado on the coast of Uruguay, followed by Castelnau’s description in 1855 [30] of *Chromys oblonga*, stated to be from the from the Rio Tocantins basin in Goiás, Brazil. In 1862, Günther [31] referred *C*. *oblonga* to *Heros* Heckel, 1840, a genus erected for Neotropical cichlids possessing more than four anal-fin spines [32]. Next to *Heros oblongus*, Günther described *Heros autochthon* based on specimens without precise locality but stated to be from Brazil and donated by Lord Stuart [31]. Better provenience was introduced by Steindachner in 1869 [33], describing *Heros Jenynsii* from Montevideo in Uruguay, and Hensel in 1870 [34], describing *Heros acaroides* from Porto Alegre and the Rio Cadeia in the state of Rio Grande do Sul, Brazil.

With the exception of *Chromys oblonga*, the type of which was in a poor state of preservation already at the time of description [30], the descriptions of these species were very similar and they were variously synonymised by later authors. In 1875, Steindachner [35], ignoring *C*. *oblonga*, synonymised *Heros* with *Acara* Heckel, 1840 [32], and *H*. *jenynsii* and *H*. *acaroides* with *Chromis facetus*. He referred to *Acara faceta* as a south Brazilian species and identified specimens from southeastern Brazil as *Acara autochthon*.

In 1904, Pellegrin [36] synonymised *Heros jenynsii* and *H*. *acaroides* with *Chromis facetus* in the genus *Cichlasoma* Swainson, 1839, but maintained *Heros autochthon* and *Chromys oblonga* as valid species in *Heros*. He observed, however, that *H*. *autochthon* might differ from *C*. *facetum* only in the absence of a lower lip frenum, and that *Chromys oblonga* might be the same species as *H*. *autochthon*. In 1905, Regan [37] synonymised *Heros* under *Cichlasoma* (as *Cichlosoma*, an unjustified emendation of *Cichlasoma*), and considered *Heros autochthon*, *Chromis facetus*, and *Chromys oblonga* to be valid species of *Cichlasoma*, in which genus they remained until 1983 when Kullander [38] separated *Australoheros facetus* from *Cichlasoma–*but without proposing a new generic name or addressing other nominal species. By that time, Haseman [39] had synonymised *Heros autochthon* and *Chromys oblonga* under *Cichlasoma facetum*. Two species currently in *Australoheros* were described under the temporary genus designation ‘*Cichlasoma*’, namely ‘*Cichlasoma’ tembe* Casciotta, Almirón & Gómez, 1995, and ‘*Cichlasoma’ scitulum* Říčan & Kullander, 2003.

In their phylogenetic analysis of 2006 Říčan and Kullander [3] demonstrated an unexpected high diversity of species in *‘Cichlasoma’* from southern South America. They named the new genus *Australoheros*, with *Chromis facetus* as type species, but did not propose names for the new species that they distinguished. Eventually, in 2008 Říčan and Kullander [4] revised the genus and described nine species of *Australoheros* from the Rio Uruguay basin and tributaries of the lower Rio Paraná, four of which new: *Australoheros forquilha*, *A*. *charrua*, *A*. *minuano*, and *A*. *guarani*. Two species from coastal rivers in eastern Uruguay were identified as *A*. *facetus* and *A*. cf. *facetus*, respectively. A putatively undescribed species from the Rio Jacuí basin in Brazil was left with the interim name *Australoheros* sp.“Jacui”; *Heros acaroides*, from the same river basin, was not mentioned.

In 2011 Říčan et al. [13] followed up with descriptions of another two species from the Uruguay and Iguaçu drainages—*Australoheros angiru* Říčan, Piálek, Almirón & Casciotta, 2011 (misidentified in 2008 [4] as *A*. *kaaygua*), and *A*. *ykeregua* Říčan, Piálek, Almirón & Casciotta, 2011 *–*further exposing considerable richness of endemic species of *Australoheros* in the Rio Uruguay basin. Meanwhile, Ottoni and Cheffe [7] described a new species, *Australoheros taura*, from the upper Rio Taquari (Rio Jacuí basin) in the State of Rio Grande do Sul, Brazil. That paper did not mention *Heros acaroides*, described from the same river basin; it was revalidated in 2010 by Schindler et al. [11], who designated and illustrated a lecotype and reported *Australoheros acaroides* from a few localities in the Rio Jacuí basin, Laguna dos Patos, and Lagoa Mirim in Rio Grande do Sul.

Ottoni et al. [5] described *Australoheros ribeirae* Ottoni, Oyakawa & Costa, 2008 from the coastal Rio Ribeira de Iguape, followed by descriptions by Ottoni and Costa [6] in the same year of another nine new species of *Australoheros* from localities in the Sudeste region of Brazil, including tributaries of the Rio Paraíba do Sul, short coastal rivers in the state of Rio de Janeiro, and a distant location in the Rio Doce drainage (*Australoheros autrani*, *A*. *barbosae*, *A*. *ipatinguensis*, *A*. *macaensis*, *A*. *macacuensis*, *A*. *muriae*, *A*. *paraibae*, *A*. *robustus*, and *A*. *saquarema*). *Chromys oblonga* and *Heros autochthon*, described from the same region, were not mentioned. Ottoni described *Australoheros capixaba* Ottoni, 2010 [80] from coastal rivers in the State of Espírito Santo, and Ottoni et al. [10] added *Australoheros perdi* Ottoni, Lezama, Triques, Fragoso Moura, Lucas & Barbosa, 2011 from a lake in the Rio Doce basin.

All coastal species of *Australoheros* share a colour pattern dominated by dark vertical bars and are also moderately variable in meristic data and proportional measurements. Based on a morphology-based phylogenetic analysis including data from Ottoni’s descriptions and additional specimens from southeastern Brazil, Říčan et al. [13] concluded that all species described by Ottoni and collaborators 2008–2010 [5–8] made up a monophyletic clade (single species) sister to the *Australoheros* assemblage in the Uruguay and lower Paraná drainage basins. Říčan et al. [13] pointed out that all northern putative species apparently lacked autapomorphies and might represent a case of excessive splitting, as suggested also by Říčan et al. [40] in a phylogenetic analysis of heroine cichlids. Ottoni [9] responded to Rican et al. [13] with descriptions in 2012 of three additional species–*Australoheros mattosi* from the Rio São Francisco basin, *A*. *montanus* from the Rio Paraíba do Sul basin, and *A*. *tavaresi* from the Rio Tietê basin–and discarded Říčan et al.’s analysis [13] as it would not be ‘suitable to test the species from the southeastern Brazil’. In the same paper, on the basis of photographs, he designated and figured as lectotype of *Heros autochthon* a putative syntype which he determined was not a specimen of *Australoheros*. The three remaining syntypes were dismissed as not ‘allowing positioning’, probably meaning that they could not be identified to genus. Without having examined the specimen, he stated that the holotype of *Chromys oblonga* would be in a too bad state of preservation to allow identification as a species of *Australoheros*. *Australoheros sanguineus*, described by Ottoni 2013 [12] from the Rio Cubatão in the state of Santa Catarina, Brazil, is the most recently described species of *Australoheros*.

Meanwhile, several phylogenetic analyses were published that include species of *Australoheros*. Kullander [41] included *‘Cichlasoma’ facetum* (actually, specimens of both *A*. *facetus* and *A*. *acaroides*) in a morphological phylogenetic analysis of South American cichlids. The analysis recovered *‘Cichlasoma’ facetum* nested in a clade with other cichlid species with elevated number of anal-fin spines. This clade, encompassing also the majority of the Middle American cichlids, was recognised as the tribe Heroini within the subfamily Cichlasomatinae. Cichlasomatinae Kullander,1998 postdates the tribe (with no subfamily) Therapsini Allgayer, proposed in an aquarium society magazine in 1989 [42] but not used afterwards and rejected by Říčan et al. [40]; thus, Cichlasomatinae takes priority over Therapsini A (cf. International Commission of Zoological Nomenclature 1999: Article 35.59) [43], but Heroini is a junior subjective synonym of Therapsini. Given the alternative classification of cichlids recognising only a single subfamily in the neotropics–, Cichlinae–proposed by Smith et al. [44], Cichlasomatinae is adjusted to a tribe, and Heroini and Therapsini to subtribes with no change of relative priority.

Říčan and Kullander [3], using large sets of morphological characters and mitochondrial gene sequences from heroin cichlids–including five species of *Australoheros–*demonstrated the monophyly of *Australoheros* and its position among heroin cichlids. The morphological analysis was extended with the recognition of four distinct species groups, the forquilha group (*A*. *forquilha*, *A*. sp. “Jacuí”, *A*. *tembe*), the scitulus group (*A*. *scitulus*, *A*. *charrua*), the kaaygua group (*A*. *kaaygua*, *A*. *minuano*), and the facetus group (*A*. *facetus*, *A*. cf. *facetus*, *A*. *guarani* [4]. That analysis was expanded on by Říčan et al. [13] in 2011 to include also the new species *A*. *ykeregua*, and *A*. *angiru* (called *A*. *kaaygua* in that paper). Říčan et al.’s [40, 45] phylogenetic analyses of Middle American cichlids included only three species of *Australoheros–A*. *angiru*, *A*. *facetus*, and *A*. *scitulus*–which were recovered as monophyletic among Middle American Heroini, distant from South American Heroini. Other molecular phylogenetic studies also recovered *Australoheros* nested among Middle American Heroini and not with other South American Heroini [44, 46].

Most recently, Ottoni et al. [47] presented a combined phylogenetic and species delimitation analysis of species of *Australoheros* mainly from the Brazilian Sudeste region, based on a fragment of the *mt-cyb* gene. The study concluded that the Sudeste region held nine or 11 species, synonymising five of those considered valid by Ottoni up to 2013 [12]. The analysis included only a few of the southern species of *Australoheros*, almost all represented only by sequences available from GenBank.

Concerns have been raised [13] about the validity of the several species of *Australoheros* proposed recently, particularly since the species diversity had gone up from three valid species (*A*. *facetus*, *A*. *tembe*, *A*. *scitulus*) before the genus was named in 2006 [3], to 28 species in 2013 [12]. The genus was traditionally studied from two different geographical perspectives, encompassing either species from the Sudeste region of Brazil, or from the Uruguay and Paraná basins. This is most clearly seen in the available phylogenetic analyses which are focused on either the southern species or the northern species. Furthermore, nomenclatural uncertainty persists because type specimens of species described in the 19th Century have not been matched against material collected recently.

Although this was never clearly expressed in taxonomic and phylogenetic papers dealing with the genus, but obvious from the treatment in taxonomic and phylogenetic publications, a hypothesis of evolution and speciation was implicitly proposed, namely that there is at least significantly elevated endemic species diversity in the extreme northern and southern ranges of the genus, and that the northern and southern species represent reciprocally separate phylogenetic lineages, similar to species flocks. This view was nevertheless never tested although it might have significant implications for understanding of the biogeography, conservation and evolution of the southern South American fish fauna.

To close the geographical gap in *Australoheros* taxonomy, and to investigate the conflicting conclusions of Říčan et al. [13] and Ottoni et al. [47], we analysed a large and representative number of specimens of coastal species of *Australoheros* with respect to traditional morphological characters, and performed a molecular phylogenetic analysis based on mitochondrial genes. The focus group consists of samples from coastal regions, primarily coastal streams in Uruguay, the Laguna dos Patos basin in Brazil, and rivers along the Brazilian coast to the Rio Itaúnas basin, representing taxa that were never before analysed in the same context but which were already indicated as distinct from the species in tributaries of the middle and upper Rio Uruguay. Supplemented with the mitochondrial *cytochrome b*, the analysis was based on the mitochondrial c*ytochrome c oxidase subunit I* gene, the ‘DNA barcode gene’, which was very little used in cichlid systematics so far but which has proven efficient in species delimitation analyses of teleosts and for which there is a huge and growing body of knowledge for its application in species delimitation and phylogenetic analysis [48, 49]. The analysis also gave us reason to address the application of the Unified Species Concept of de Queiroz [50] and to remark on the difference between a formal description of a species in a nomenclatural context, and the actual demonstration of species status.

## Material and methods

**Fig 1:**
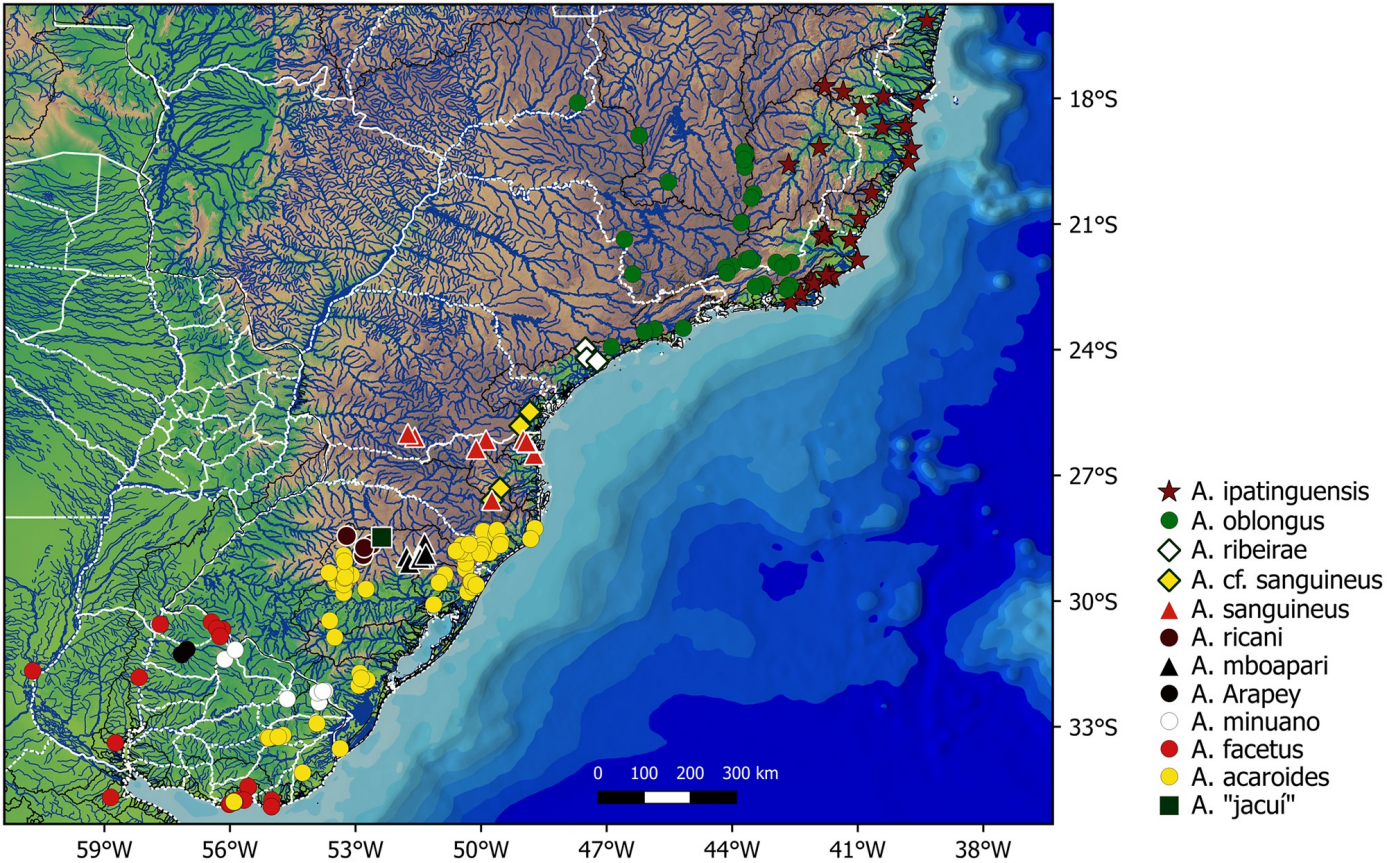
Sampling sites of coastal species of *Australoheros*. A symbol may cover more than one sampling site.

Most specimens examined were already part of museum collections. Complementary specimens were collected in Brazil in the states of Rio Grande do Sul, Santa Catarina, Rio de Janeiro, and Minas Gerais under permit ICMBio numbers 44694–2 to 5. In the field, individuals were euthanised in an overdose of clove oil following protocols and recommendations of Lucena et al. [51] and Use of Fishes in Research Committee [52]. Muscle samples for molecular analysis were preserved in 98–99% ethanol and stored in freezer at about -20 °C; voucher specimens were fixed in 10% formalin, and eventually transferred to 70% ethanol for long term storage. Sampling sites are shown in Fig 1.

Specimens examined are specified under each species account, and are catalogued in the following institutions:

BMNH The Natural History Museum, London;
CIMC Divisão de Fauna, Grupo Especial de Estudo e Proteção do Ambiente acuático do Rio Grande do Sul, Pelotas;
CZNC Divisão Ictiológica da Coleção Zoológica Norte Capixaba, Universidade Federal do Espírito Santo, São Mateus;
LBP Laboratório de Biologia de Peixes, Universidade Estadual Paulista, Instituto de Biociências, Campus de Botucatú, Botucatú;
MCP Museu de Ciências e Tecnologia da Pontifícia Universidade Católica do Rio Grande do Sul, Porto Alegre;
MNHN Muséum national d’Histoire naturelle, Paris;
MHNG Muséum d’Histoire naturelle, Genève;
MNHN Museo Nacional de Historia Natural, Montevideo;
MNRJ Museu Nacional, Rio de Janeiro;
NMW Naturhistorisches Museum, Wien;
MUHNAC Museu Nacional de História Natural e da Ciência, Universidade de Lisboa, Lisboa;
MZUSP Museu de Zoologia da Universidade de São Paulo, São Paulo;
NRM Swedish Museum of Natural History, Stockholm;
NUP Coleção Ictiológica do Núcleo de Pesquisas em Limnologia, Ictiologia e Aquicultura da Universidade Estadual de Maringá, Maringá
NPM Instituto de Biodiversidade e Sustentabilidade, Universidade Federal d Rio de Janeiro, Macaé;
Universidade Estadual de Maringá, Maringá;
STRI Smithsonian Tropical Research Institute, Panamá;
UFBA Museu de Zoologia, Universidade Federal da Bahia, Salvador;
UFRGS Coleção de Peixes, Universidade Federal do Rio Grande do Sul, Porto Alegre;
UNICTIO Laboratório de Ictiologia, Universidade do Vale do Rio dos Sinos, São Leopoldo;
UVN Universidade Federal de Viçosa, Viçosa,;
ZFMK Zoologisches Forschungsinstitut und Museum Alexander Koenig, Bonn;
ZMB Museum für Naturkunde, Berlin.
ZSM Zoologische Staatssammlung, München

Other institutional repositories mentioned:

DZUFMG Departamento de Zoologia da Universidade Federal de Minas Gerais, Belo Horisonte;
MACN-ict Colección Nacional de Ictiología, Museo Argentino de Ciencias Naturales Bernardino Rivadavia, Buenos Aires;
MLP Museo de La Plata, La Plata;
MTD Senckenberg Naturhistorische Sammlungen Dresden, Dresden;
UFRJ Instituto de Biologia, Universidade Federal de Rio de Janeiro, Rio de Janeiro;
UMZC University Museum of Zoology, Cambridge.

### Species concept

Under the Unified Species Concept [50], species are concepts (or hypotheses) recognised from evidence of lineage separation. We explored specimens for such evidence using morphological characters, DNA-based phylogenies, and coalescence analysis.

### Morphology

Measurements and counts were obtained as described by Kullander [53] except that snout and head lengths represent point to point measurements. Projected measurements as used by Říčan Kullander [4] and Kullander [53] may be preferable for small species and specimens, on which the calliper tip adds considerably to the measurement, but are time-consuming and not likely more accurate in large preserved specimens which usually are slightly asymmetric due to post-mortem pressure in holding containers or museum jars. Projected measurements give slightly shorter values, more so in specimens with a deep lachrymal bone.

Distance measurements were analysed with the statistics program SYSTAT, v.13 [54] (descriptive statistics, regression analysis, Mann-Whitney U Test, ANCOVA, plots). Sheared Principal Component Analysis was performed with log-transformed data [55]. The first component was identified as multivariate size, and the following components identified as representing distance vectors insofar as variance was >1. In the two-dimensional plots of PCA scores, ellipses only facilitate identification of species samples; ellipses are centered on sample means of the two component axes; the major axes reflect the standard deviations of the x and y values, and the orientation reflects the sample covariance between each x and y value. The size of the ellipse reflects a probability set to the default 0.6827, ensuring that the ellipse fits into the diagram. Enclosing hull instead of ellipse was used when required for clarity.

The count of scales along the side was made in the horizontal row immediately above that containing the lower lateral line, and excluded the scale on the cleithrum as well as any scale inserted on the caudal-fin base. The count of scales in the lower lateral line excluded any scale on the caudal fin, but included the last scale on the caudal peduncle although it may extend over the caudal-fin base. The count of scales on the cheek was made along an imaginary line down from the middle of the orbit. The count of predorsal scales was made along the midline of the occiput and was approximate as those scales are irregularly arranged and often embedded without free margins. Scale rows around the caudal peduncle were counted on the middle of the caudal peduncle.

Vertebrae were counted on X-radiographs and reported as precaudal+caudal; the first caudal vertebra is that with the haemal spine inserted behind the anteriormost anal-fin pterygiophores [56, 57]. This convention, used by Říčan et al. [13] and Říčan and Kullander [4] typically gave 13 precaudal vertebrae in *Australoheros*. The X-radiograph published by Ottoni [12] shows the next vertebra labelled as the first caudal vertebra. As checked on cleared and stained specimens, our first caudal vertebra had a complete haemal arch, and may also have short parapophyses.

The posteriormost dorsal- and anal-fin soft ray was commonly composed of two rays sharing the same distal radial. In intact specimens, it could be difficult to decide if the last ray was integer or composite, whereas the condition was shown clearly in X-radiographs. Variation in count of soft-fin rays may reflect variation in the determination of the posteriormost ray, but as a rule the last compound ray was registered as composed of two rays also in specimens not X-radiographed.

Proximal radials of the dorsal- and anal-fin rays were reported by Říčan and Kullander [4] and Ottoni and collaborators [5–12]. Proximal radials are those elements of the fin ray support that are proximal to and articulating with the distal radials to which the fin rays attach. In cichlids the first two anal-fin spines articulate on the same compound proximal radial. The last two soft rays may share the same proximal+distal radial or not. The number of anal rays should match the number of proximal radials plus one, but it may be difficult to identify the posterior proximal radials on X-radiographs, and a count of fin-rays may be more accurate. The number of proximal radials supporting the dorsal fin corresponds to the number of spines+soft fin rays, except that the last two rays may share proximal radial. Counting radials may be purposeful when spines and soft fin rays are lost or damaged.

The terms isognathous, prognathous, and retrognathous refer to the relative projection of the lower jaw (without lips) when the mouth is closed, describing the condition respectively where the upper and lower jaw bite at the same anterior extension; the lower jaw projects slightly anterior of the upper jaw; and the upper jaw projects slightly anterior of the lower jaw. In cichlids, jaws are typically isognathous, as in *Australoheros*. Prognathous jaws are found in many piscivorous cichlids (e.g. species of *Crenicichla* Heckel, 1840, *Petenia splendida* (Günther, 1862). Retrognathous lower jaw must be very rare in cichlids, as we did not find any record of this condition. Prognathous jaws were recorded but not confirmed in several descriptions of species of *Australoheros*, e.g. Říčan and Kullander [4], and Ottoni and Costa, [6], and seems rather to refer to the inclination of the lower jaw. In very large specimens of *A*. *facetus* and *A*. *acaroides*, the lower jaw was relatively thick and angled, and closed slightly before the upper jaw. In all other *Australoheros*, the jaws were equal in anterior extension or the upper jaw closing slightly in advance of the lower. Retrognathous jaws reported in *Australoheros taura* [7] apparently refers to the extension of the upper lip and not to a short lower jaw.

Gill-rakers counted were those externally (laterally) on the first gill arch, where they were present on the epibranchial and ceratobranchial portions, and there was usually also one situated in the joint between the epibranchial and ceratobranchial. The epibranchial gill rakers were two or three in number and occasionally difficult to count in poorly preserved or very rigid specimens. Thus, only the ceratobranchial number was tabulated. Gill rakers on the medial side were difficult to examine on whole specimens without lifting the gill cover and first gill-arch to the extent that either potentially suffered damage.

Ottoni and collaborators used the same description for the gill-raker count, but provided three numbers. Ottoni et al. [10] provided an explanation in the description of *Australoheros perdi*: ‘Gill rakers (25) from the first gill arch are in two series, one in the inner side and the other in the outer side, all of whom may fall out of the specimen easily under manipulation and can only be correctly studied in cleared, stained and dissected specimens; the count is expressed as a formula (x + y; where x refers to the ceratobranchial rakers and y those from the epibranchial together with possible rakers from the intermediary cartilage); inner and outer rakers from each bone are added and expressed by a single number.’ In their table however, they report ‘Gill-rakers on first ceratobranchial 15(2)– 16(3) + 5(1)– 6(2)– 7(2)’. It corresponds to 15–16 medial, and 5, 6, or 7 lateral. Gill rakers were not mentioned elsewhere in that paper.

Sex was determined in adult specimens by the shape of the genital papilla which was conical or spindle-shaped in males, tipped by several short filaments and with a narrow terminal opening; in females a short thick-walled tube with a transverse or v-shaped wide aperture.

**Fig 2:**
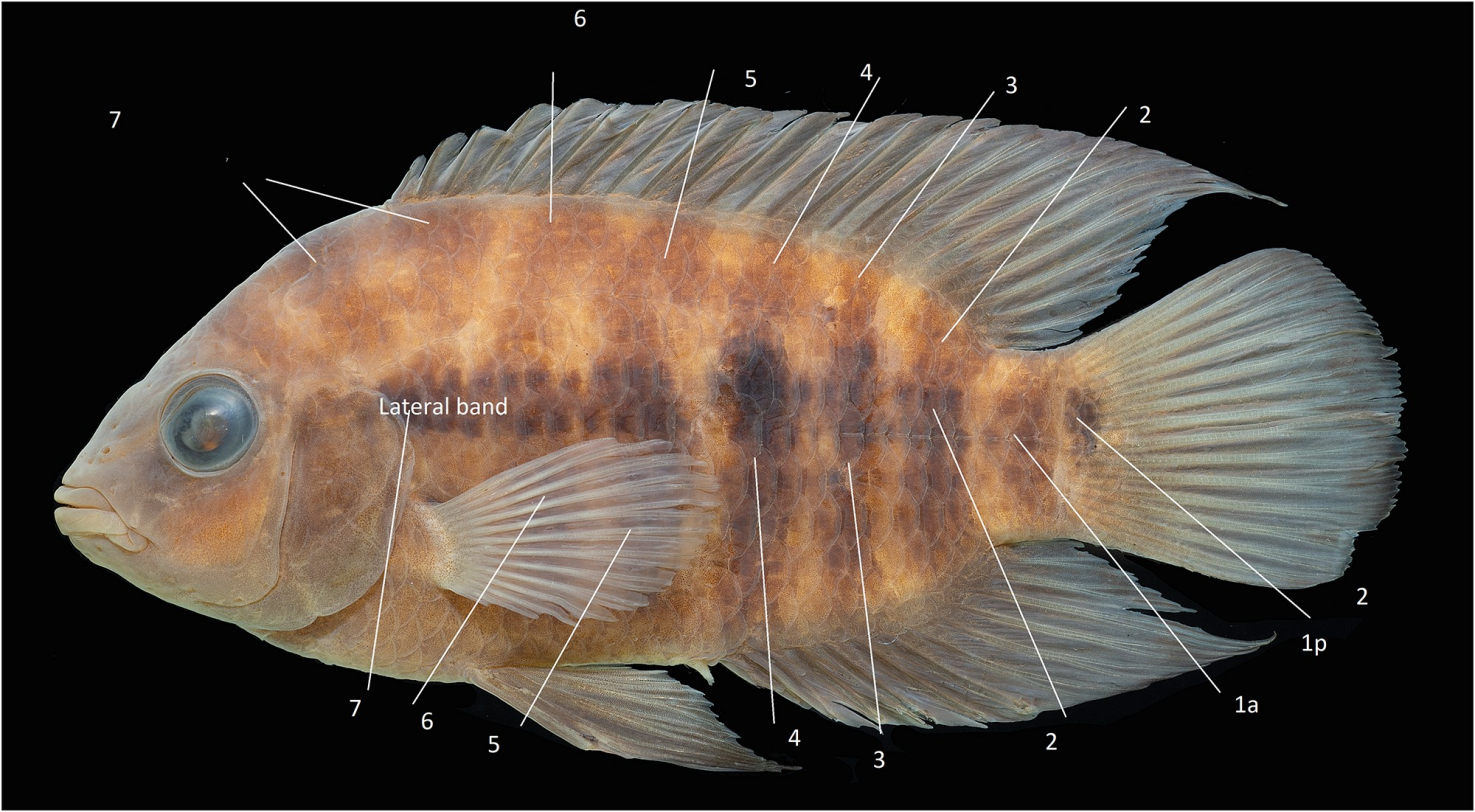
Map of numbering of vertical dark lateral bars in in *Australoheros*.

The colour pattern terminology followed Říčan et al. [58]. The vertical dark bar on the caudal peduncle and the dark blotch at the base of the caudal fin were considered to be expressions of the same marking (anterior and posterior divisions of Bar 1, Bar 1 a and 1p, respectively). The remaining bars were numbered in succession from posterior to anterior as in Říčan et al. fig. 1 [58]; (Fig 2).

Bars 2–5 were expressed as narrow dark bars extending from the dorsum to the lower side, and were present in all species of *Australoheros*; Bars 4–6 were located in succession between the head and the anal fin, and called abdominal bars. Bar 5 was either entire, as one of three abdominal bars; or split vertically, resulting in four abdominal bars. Bar 7 was usually obsolete, expressed as a dark patch close to the gill opening, and two, divergent stripes, one crossing the head well above the orbit; the other extending to the dorsal-fin base. We distinguished between the portion of the dark bars that extended down for most of the side, which were vertically oriented and which usually were clearly formed by black or brown pigment restricted by scale borders; and the dorsal portion which was uniformly grey-brown or brown and mostly slanting slightly caudad from the rest of the bar, although this portion of Bars 4–6 could also have a rostrad inclination. The two portions corresponded to the intersegmental dorsal blotches recognised by Říčan et al. [58]). When the dorsal part of Bar 6 slanted caudad from the dorsal-fin base to the rostrad slanting upper portion of Bar 5 (or, rarely, Bar 5 and Bar 4) they combined to form a Y mark). Similarly, it was bar portions that formed ring-like marks enclosing a lighter area immediately below the anterior dorsal-fin base. Interbars are the light bars between the dark bars, and were numbered from 0 (between Bar 1a and Bar 1p) to 5 (between Bars 5 and 6).

Live colours have been advocated as critical for determination of species of *Australoheros* [13, 59]. Because of fragmentary data, live colours are not given extensive consideration here. The popular English name chameleon cichlid applied on *Australoheros facetus* indicates that colouration may be variable in individuals of *A*. *facetus* at least. The name may, however, reflect mainly the melanophore pattern as described by Bade in 1923 [60]: ‘Körperfarbe wechselnd, in der Regel braungelb oder grünlich, bald mit hellen, bald bunteren breiten Binden geziert, die sich auch auf die Flossen ausdehnen. Diese Binden können oft ganz verblassen, es zeigt sich dann nur eine schwarze Mittellinie oder oft nur einige bunte Flecke’. Live *Australoheros* may be just black (*A*. *facetus*), or with a reflecting green or blue body with minute patches of red or orange all over, or sides red; and caudal fin pale or deep red in its entirety, or along all or part of the margin of the caudal fin. The breeding colour is typically yellow with contrasting black markings and yellow or red eye [13, 27, 59, 61, 62]. Available information by species is almost exclusively anecdotal; that is, colour terminology was not standardised, does not relate to behaviour or sex, and concerns specimens in aquaria, stressed in a small photo tank, handheld in air, or dropped on the ground. Photos in aquarium journals may appear in different hues.

Photographs of whole specimens were made of specimens immersed in alcohol in photo aquaria, under variable light conditions and with different cameras and depicting specimens in different states of preservation and with various post-mortem deformations. Narrow unsharp mask was applied and the background set to black, eliminating scratches, dirt, and reflections in areas outside of specimens. Images are representative of specimen habitus and melanophore colour pattern, but other colours are affected by preservation and lightning.

Osteological preparations of whole specimens cleared and stained with Alizarin Red S and Alcian Blue using the method of Taylor and Van Dyke [63] were available as follows: *A*. *acaroides*, NRM 37035, 56.2 mm SL; NRM 37037, 63.4 mm SL.—*A*. *minuano*: NRM 51079, pt., 69.7 mm SL.—*Australoheros angiru* NRM 49159, 65.1 mm SL; ZSM 23060, pt., 52.2 mm SL.—*A*. *facetus*, NRM 37040, 61.1 mm SL.—*A*. *scitulus*: NRM 49899 pt., 53.8 mm SL.—*A*. *ykeregua*: ZSM 23060 pt., 48.4 mm SL; ZSM 23482, 73.3 mm SL.—*A*. sp. “Jacuí”: MZUSP 30411 (P), 60.8 mm SL; MZUSP 30411(L), 70.6 mm SL.

### DNA processing

For the genetic analysis, a 654 basepair (bp) fragment of the 5′ end of the mitochondrial c*ytochrome c oxidase subunit I* gene (*mt*-*co1*) and the complete or nearly complete mitochondrial *cytochrome b* gene (*mt-cyb)* was sequenced from morphologically identified specimens. At NRM, DNA was extracted, and *mt-co1* sequences obtained as described by Kullander et al. [49]. *mt-cyb* was amplified using the primers CYTB_GlufishF (AACCACCGTTGTTATTCAACTACAA) and CYTB_TrucCytb (CCGACTTCCGGATTACAAGACCG), with the PCR cycling: 94 °C for 4 min; 40 * (94 °C for 30s; 52 °C for 30s; 72 °C for 60s); 72 °C for 8 min. The PCR products were processed as described by Kullander et al. [49].

At MCP, total genomic DNA was isolated using the DNeasy Blood & Tissues Kit (Qiagen). All polymerase chain reactions (PCR) were performed in 25 μL reactions using 1.25 μL of 10 μM of each primer, 12.5 μL of water, 8 μL of HotStarTaq Master Mix Kit (Qiagen; 3 μM MgCl_2_ buffer and 400 μM of each dNTP) and 2 μL (10–50 ng) of DNA extract. The gene *mt*-*co1* was amplified using the primers FISH-F1 (5′TCAACCAACCACAAAGACATTGGCAC 3′) and FISH-R1 (5′ TAGACTTCTGGGTGGCCAAAGAATCA 3′) designed by Ward et al. [64], with a PCR temperature protocol, as follows: initial denaturation at 95 °C for 15 min; 35 repetitions of the cycle of 95 °C for 30 s, 49 °C, 52 °C, 54 °C for 20 s each, and 72 °C for 60 s; with a final extension at 72 °C for 10 min. Whenever amplifications failed, a touchdown annealing temperature protocol was used by 40 repetitions of the cycle of 94 °C for 30 s, 52 °C for 20 s, 50 °C for 10 s, 48 °C for 10 s, 46 °C for 10 s, 44 °C for 20 s, and 72 °C for 60 s; and final extension at 72 °C for 10 min. The gene *mt-cyb* was amplified using the primers FishcytbF (5’ ACCACCGTTGTTATTCAACTACAAGAAC 3’) and CYTB_TrucCytb (5′ CCGACTTCCGGATTACAAGACCG 3′) [65], with a touchdown PCR temperature protocol as follows: initial denaturation at 95 °C for 15 min; 35 repetitions of the cycle of 95 °C for 30 s, 58 °C, 56 °C, 54 °C for 20 s each, and 72 °C for 90 s; with a final extension at 72 °C for 10 min. Whenever amplifications failed, a second touchdown annealing temperature protocol was used at 57 °C, 55 °C, 53 °C for 20 s each. To test PCR results, the amplicons were stained with a mix of Blue Juice (diluted at 1:2 in Milli-Qwater) plus gel red (1 μL diluted to 1 mL of the prepared Blue Juice mix), and then tested by horizontal gel electrophoresis on a 4% agarose gel. Results of the amplicons were visualised and registered by photography with an ultraviolet camera. The amplicons were purified and sequenced in both directions using two distinct sequencing services: Macrogen Europe Inc. (Amsterdam, The Netherlands; for NRM samples) and Functional Biosciences, Inc. (Madison, Wisconsin, USA; for South American museum samples).

The obtained *mt-coI* sequences were aligned with all 600 bp or longer corresponding sequences of *Australoheros* available from GenBank [66] and BOLD [67], for a total of 118 sequences. The obtained *mt*-*cyb* sequences were aligned with all corresponding 950 bp or longer sequences of *Australoheros* available from GenBank for a total of 136 sequences. The sequences were aligned using Geneious [68] with the MAFFT plug-in [69], and ends were manually trimmed to a total alignment length of 652 bp for *mt-coI* and 1144 bp for *mt-cyb*. New nucleotide sequences were uploaded to GenBank; GenBank accession numbers and voucher metadata for all new sequences are given in Table 1; for downloaded sequences in S1 Table in S1 File. Gene names and symbols follow the Zebrafish Information Network [70] nomenclature conventions.

**Table 1:** New sequences of *Australoheros* and *Cichlasoma* generated, with GenBank accession numbers, collection identifiers, and collection site.

GB *mt-cyb*	GB *mt-coI*	Collection	Cat.no.	Tissue no.	Species	Country/state	Municipality	Locality	Lat.	Long.	Basin
OK020110	MZ969503	NRM	52247	3321	*A*. *scitulus*	U-AR	Rincón de Pacheco	Rincón de Pacheco, 1 km from estancia buildings, cañada tributary to Río Cuareim	-30.67267	-56.18095	Cuareim
OK020102	MZ969505	NRM	54196	3911	*A*. *minuano*	U-YCL	Centurión	Stream crossing Ruta 26 Melo-Tacuarembó, about km 389, tributary to Río Negro	-32.33807	-54.63249	Negro
OK020096	MZ969506	NRM	52565	3927	*A*. *facetus*	UY-AR	Artigas	Arroyo Catalán Grande, tributary to Rio Quaraí, below bridge on road 30	-30.84183	-56.23881	Cuareim
OK020097	MZ969507	NRM	52591	3950	*A*. *facetus*	UY-CA	El Pinar	El Pinar, isolated pool	-34.79812	-55.88403	Laguna del Diario
OK020100	MZ969508	NRM	54147	3972	*A*. *minuano*	UY-CL	Centurión	Centurión, Arroyo Ceibal down from bridge	-32.15172	-53.78197	Yaguarón
OK020101	MZ969509	NRM	54147	3973	*A*. *minuano*	UY-CL	Centurión	Centurión, Arroyo Ceibal down from bridge	-32.15172	-53.78197	Yaguarón
OK020103	MZ969510	NRM	55067	4014	*A*. *minuano*	UY-CL	Chuy	Small stream crossing Ruta 26, near Posta del Chuy	-32.41394	-53.87114	Tacuary
OK020104	MZ969511	NRM	55068	4015	*A*. *minuano*	UY-CL	Centurión	Centurión, Arroyo Ceibal down from bridge	-32.15172	-53.78197	Yaguarón
OK020105	MZ969513	NRM	55152	4079	*A*. *minuano*	UY-CL	Ramón Trigo	stream tributary to Rio Negro crossing Ruta 26 Melo–Tacuarembó, at about km 389	-32.33807	-54.63249	Negro
OK020098	MZ969514	NRM	55720	4514	*A*. *facetus*	UY-MA	Maldonado	Laguna del Diário	-34.90479	-55.00810	Laguna del Diario
OK020111	MZ969515	NRM	55338	4858	*A*. *scitulus*	UY-AR	San Luis	Arroyo Catalán Grande, tributary to Rio Quaraí, below bridge on road 30	-30.84183	-56.23881	Cuareim
OK020107	MZ969518	NRM	61526	8106	*A*. *minuano*	UY-CL	Centurión	Paso del Centurión, southern end of small lake slightly east of Río Yaguarón	-32.14265	-53.72955	Yaguarón
OK020106	—	NRM	61397	8123	*A*. *minuano*	UY-CL	Centurión	small stream crossing Ruta 7 ca 15 km from Paso del Centurión	-32.18462	-53.90626	Yaguarón
OK020133	—	NRM	61491	8179	*A*. *acaroides*	UY-RV	Rocha	Velázquez	-34.09770	-54.26470	Cebollatí
OK020094	MZ969519	NRM	61491	8180	*A*. *acaroides*	UY-RV	Rocha	Velázquez	-34.09770	-54.26470	Cebollatí
OK020093	MZ969520	NRM	61427	8187	*A*. *acaroides*	UY-RV7TT	Rincón de Gutiérrez	Tributary to Arroyo de las Averías at Estancia El Ytay close to rroyo de las Pavas	-33.24110	-54.84550	Olimar
OK020099	—	NRM	70180	12623	*A*. *acaroides*	UY-TT	Vergara	Tributary to Arroyo del Parao under bridge, Ruta 18, W of Vergara	-32.91998	53.92171	Olimar
OK020095	—	NRM	70181	12786	*A*. *acaroides*	UY-TT	Vergara	Tributary to Arroyo del Parao under bridge, Ruta 18, W of Vergara	-32.91998	53.92171	Rio Olimar Grande
OK020122	MZ969522	UFRGS	19072	CIC125	*A*. *ipatinguensis*	BR-MG	Pingo d’Áqua	Lagoa Juiz de Fora	-19.69929	-42.48242	Doce
OK020121	MZ969523	UFRGS	19062	CIC126	*A*. *ipatinguensis*	BR-MG	Pingo d’Áqua	Lagoa Tiririca, left margin	-19.74719	-42.44560	Doce
OK020120	MZ969525	UFRGS	18879	CIC128	*A*. *ipatinguensis*	BR-RJ	Silva Jardim	Rampart on road between Gaviões and Japuíba	-22.57583	-42.56083	São João
OK020131	MZ969526	UFRGS	19024	CIC130	*A*. *ipatinguensis*	BR-ES	Conceição da Barra	córrego on local road to ES-416, near border with Bahia	-18.29639	-39.79444	Itaúnas
OK020118	MZ969531	CZNC	1099	CIC162	*A*. *ipatinguensis*	BR-ES	Cristal do Norte	Rio Itaúnas	NO DATA	NO DATA	Itaúnas
OK020112	MZ969540	LBP	14581	CIC186	*A*. *acaroides*	BR-RS	Júlio de Castilhos	Stream without name	-29.31878	-53.63311	Soturno
OK020128	MZ969543	LBP	2133	CIC189	*A*. *ribeirae*	BR-SP	Pedro de Toledo	Rio do Peixe	-24.27653	-47.22589	do Peixe
OK020119	MZ969550	MNRJ	47279	CIC283	*A*. *ipatinguensis*	BR-RJ	Macaé	Rio dos Quarenta at BR-101 bridge, about 500 m from the interchange with highway RJ107	-22.21833	-41.75889	Macaé
OK020124	MZ969551	MNRJ	47247	CIC285	*A*. *oblonga*	BR-RJ	Cachoeiras do Macacu	Rio Macacu, at mouth of Rio Branco, Japuíba	-22.55528	-42.69472	Macacu
OK020125	MZ969561	MNRJ	47336	CIC295	*A*. *oblongus*	MR-MG	Mar de Espanha	Dammed tributary of Córrego Cachoeirinha, in Fazenda Jardim	-21.92528	-42.97722	Paraíba do Sul
OK020132	MZ969562	MNRJ	47336	CIC296	*A*. *oblongus*	MR-MG	Mar de Espanha	Dammed tributary of Córrego Cachoeirinha, in Fazenda Jardim	-21.92528	-42.97722	Paraíba do Sul
OK020126	MZ969567	MNRJ	47365	CIC301	*A*. *oblongus*	MR-MG	Juiz de Fora	tributary of Rio do Peixe, between Toledo and Torreões	-21.82361	-43.58806	do Peixe
OK020127	MZ969568	MNRJ	47365	CIC302	*A*. *oblongus*	BR-MG	Juiz de Fora	Tributary of Rio do Peixe, between Toledo and Torreões	-21.82361	-43.58806	do Peixe
OK020123	MZ969575	MCP	49295	CIC309	*A*. *oblongus*	BR-MG	Jaboticatubas	Rio Cipó, tributary of Rio das Velhas	-19.28250	-43.72972	São Francisco
OK020114	MZ969578	MCP	49695	CIC311	*A*. *acaroides*	BR-RS	Bom Jesus	Rio Manoel Leão on road Silveira-Rondinha	-28.63556	-50.07853	Pelotas
OK020116	MZ969583	MCP	50424	CIC321	*A*. *acaroides*	BR-RS	Faxinal do Soturno	Rio Soturno, bridge close to road between Nova Roma and Faxinal do Soturno	-29.53525	-53.48061	Soturno
OK020117	MZ969584	MCP	50425_F5	CIC322	*A*. *acaroides*	BR-RS	Júlio de Castilhos	Arroio Felicio, road between Parque das Esculturas and Júlio de Castilhos	-29.31844	-53.63272	Soturno
OK020130	MZ969585	MCP	50427_F9	CIC323	*A*. *ricani*	BR-RS	Espumoso	Rio Morcego, local road beteen Espumoso and Soledade	-28.89861	-52.81806	Jacuí
OK020115	MZ969587	MCP	5042_5_F6	CIC325	*A*. *acaroides*	BR-RS	Júlio de Castilhos	Arroio Felicio, road between Parque das Esculturas and Júlio de Castilhos	-29.31844	-53.63272	Jacuí
OK020129	MZ969588	MCP	50427_F10	CIC326	*A*. *ricani*	BR-RS	Espumoso	Rio Morcego, local road beteen Espumoso and Soledade	-28.89861	-52.81806	Jacuí
OK020113	MZ969594	MCP	48688	CIC34	*A*. *acaroides*	BR-RS	Bom Jesus	Rio dos Touros on road Rondinha-Pascoal, next to Usina Hidrelétrica Rio dos Touros	-28.64556	-50.28500	Pelotas
OK020108	MZ969603	MCP	40635_E6	E6	*A*. *sanguineus*	BR-SC	Joinville	Rio Cubatão Norte on road Quiriri de Baixo	-26.14250	-48.99583	Cubatão
OK020109	MZ969604	MCP	40635_E7	E7	*A*. *sanguineus*	BR-SC	Joinville	Rio Cubatão Norte on road Quiriri de Baixo	-26.14250	-48.99583	Cubatão
—	MZ969502	NRM	36435	121	*A*. *scitulus*	UY-CO	Rosario	Arroyo Rosario drainage: Aroyo Colla, 500 m upstream mouth into Arroyo Rosario	-34.31861	-57.33694	Río de la Plata
—	MZ969504	NRM	52219	3322	*A*. sp. *“*Arapey”	UY-SA	Valentin	10 km from Valentin, below road 31 bridge,small stream within Rio Arapey drainage	-31.27546	-57.15563	Arapey
—	MZ969512	NRM	55104	4051	*A*. *scitulus*	UY-TA	Las Toscas	Arroyo Caraguatá at Los Talas, under bridge	-32.15788	-55.02453	Negro
—	MZ969516	NRM	56420	4957	*A*. *facetus*	UY-MA	Maldonado	Laguna del Diário	-34.90479	-55.00810	Laguna del Diario
—	MZ969517	NRM	56421	4958	*A*. *facetus*	UY-MA	Maldonado	Laguna del Diário	-34.90479	-55.00810	Laguna del Diario
—	MZ969521	MCP	53174_C10	C10	*A*. *acaroides*	BR-RS	Bom Jesus	Rio dos Touros, below barrage on road Rondinha São Joaquim	-28.64583	-50.28556	Pelotas
—	MZ969524	UFRGS	19037	CIC127	*A*. *ipatinguensis*	BR-ES	Pedro Canário	Córrego do Engano on road between Pedro Canário and Montanha	-18.20806	-40.04528	Itaúnas
—	MZ969527	UFRGS	18937	CIC131	*A*. *ipatinguensis*	BR-ES	Vargem Alta	Rio Novo, on local road to road between Vargem Alta and Iconha	-20.66000	-40.96528	Novo
—	MZ969528	UFRGS	12591	CIC132	*C*. *portalegrense*	BR-SC	Meleiros	Sanga do Engenho on road between Meleiro and Forquilhinha	-28.78750	-49.54556	Araranguá
—	MZ969529	UFRGS	19912	CIC160	*A*. *acaroides*	BR-SC	Siderópolis	Rio Jordão, upstream of Cachoeira Bianchini	-28.58658	-49.52350	Jordão
—	MZ969530	CZNC	1027	CIC161	*A*. *ipatinguensis*	BR-ES	São Mateus	Córrego Canivete	NO DATA	NO DATA	São Mateus
—	MZ984107	MCP	48666	CIC169	*A*. *acaroides*	BR-RS	Sao Francisco de Paula	Arroio Ribeirao on road RS 453 (Rota do sol), tributary of Rio Tainhas	-29.23278	50.37472	
—	MZ969532	MCP	48666A	CIC170	*A*. *acaroides*	BR-RS	São Francisco de Paula	Arroio Ribeirão on road RS 453 (Rota do sol), tributary of Rio Tainhas	-29.23278	-50.37472	Antas
—	MZ969533	MCP	48666	CIC171	*A*. *acaroides*	BR-RS	São Francisco de Paula	Arroio Ribeirão on road RS 453 (Ruta do sol), tributary of Rio das Antas	-29.23278	-50.37472	Jacuí
—	MZ969534	MCP	48666	CIC174	*A*. *acaroides*	BR-RS	São Francisco de Paula	Arroio Ribeirão on road RS 453 (Ruta do sol), tributary of Rio das Antas	-29.23278	-50.37472	Jacuí
—	MZ969535	MCP	48688	CIC176	*A*. *acaroides*	BR-RS	Bom Jesus	Rio dos Touros on road Rondinha-Pascoal, next to Usina Hidrelétrica Rio dos Touros	-28.64556	-50.28500	Uruguay
—	MZ984108	MCP	48666	CIC177	*A*. *acaroides*	BR-RS	Sao Francisco de Paula	Arroio Ribeirao on road RS 453 (Rota do sol), tributary of Rio Tainhas	-29.23278	50.37472	
—	MZ969536	MCP	48688	CIC178	*A*. *acaroides*	BR-RS	Bom Jesus	Rio dos Touros on road Rondinha-Pascoal, next to Usina Hidrelétrica Rio dos Touros	-28.64556	-50.28500	Uruguai
—	MZ969537	MCP	49007	CIC179	*A*. *oblongus*	BR-MG	Jaboticatubas	Stream on road Jaboticatubas-Cardeal Mota, Rio Cipó drainage	-19.31917	-43.67944	São Francisco
—	MZ969538	MCP	49007	CIC180	*A*. *oblongus*	BR-MG	Jaboticatubas	Stream on road Jaboticatubas-Cardeal Mota, Rio Cipó drainage	-19.31917	-43.67944	São Francisco
—	MZ969539	LBP	14564	CIC185	*A*. *acaroides*	BR-RS	Agudo	Stream without name	-29.60092	-53.28067	Jacuí
—	MZ969541	LBP	14463	CIC187	*A*. *acaroides*	BR-RS	Caraá	Stream without name	-29.77614	-50.44281	dos Sinos
—	MZ969542	LBP	14389	CIC188	*A*. *oblongus*	BR-SP	Ubatuba	Rio Escuro	-23.48440	-45.17042	Escuro
—	MZ969544	LBP	1050	CIC198	*A*. *sanguineus*	BR-SC	Papanduva	Tributary of Rio São João	-26.36748	-50.11915	São João
—	MZ969545	LBP	6527	CIC199	*A*. *oblongus*	MG	Jaboticatubas	Tributary of Rio Cipó	-19.46119	-43.72031	São Francisco
—	MZ969546	LBP	9105	CIC200	*A*. *ipatinguensis*	BR-RJ	Campos dos Goytacazes	Lagoa do Brejo Grande	-21.67333	-41.28306	Paraíba do Sul
—	MZ969547	LBP	11171	CIC202	*A*. *ipatinguensis*	BR-RJ	Campos dos Goytacazes	Lagoa Brejo Grande	-21.67850	-41.28008	Paraíba do Sul
—	MZ969548	NPM	5307	CIC274	*A*. *ipatinguensis*	BR-RJ	Macaé	Parque Nacional Restinga de Jurubatiba Lagoa Cabiunas, Lagoa de Jurubatiba	-22.30139	-41.69333	Cabiunas
—	MZ969549	MNRJ	47279	CIC282	*A*. *ipatinguensis*	BR-RJ	Macaé	Rio dos Quarenta at BR-101 bridge, about 500 m from the interchange with highway RJ106	-22.21833	-41.75889	Macaé
—	MZ969552	MNRJ	47291	CIC286	*A*. *ipatinguensis*	BR-RJ	São José de Uba	Córrego São Domingos at bridge of RJ198, 200 m from crossing with BR393	-21.31556	-41.86611	Muriaé
—	MZ969553	MNRJ	47307	CIC287	*A*. *ipatinguensis*	BR-RJ	Itaperuna	Bridge over Córrego São Domingos, on road in direction of Fazenda São Domingos	-21.26222	-41.79639	Muriaé
—	MZ969554	MNRJ	47307	CIC288	*A*. *ipatinguensis*	BR-RJ	Itaperuna	Bridge over Córrego São Domingos, on road in direction of Fazenda São Domingos	-21.26222	-41.79639	Muriaé
—	MZ969555	MNRJ	47307	CIC289	*A*. *ipatinguensis*	BR-RJ	Itaperuna	Bridge over Córrego São Domingos, on road in direction of Fazenda São Domingos	-21.26222	-41.79639	Muriaé
—	MZ969556	MNRJ	47346	CIC290	*A*. *ipatinguensis*	BR-MG	Santa Rita de Jacutinga	Bridge in tributary of Córrego da Lagoa, road Passa Vinte-Santa Rita do Jacutinga	-22.16722	-44.13000	Bananal
—	MZ969557	MNRJ	47346	CIC291	*A*. *oblongus*	BR-MG	Santa Rita de Jacutinga	Bridge in tributary of Córrego da Lagoa, road Passa Vinte-Santa Rita do Jacutinga	-22.16722	-44.13000	Bananal
—	MZ969558	MNRJ	47346	CIC292	*A*. *oblongus*	BR-MG	Santa Rita de Jacutinga	Bridge in tributary of Córrego da Lagoa, road Passa Vinte-Santa Rita do Jacutinga	-22.16722	-44.13000	Bananal
—	MZ969559	MNRJ	47346	CIC293	*A*. *oblongus*	BR-MG	Santa Rita de Jacutinga	Bridge in tributary of Córrego da Lagoa, road Passa Vinte-Santa Rita do Jacutinga	-22.16722	-44.13000	Bananal
—	MZ969560	MNRJ	47346	CIC294	*A*. *oblongus*	BR-MG	Santa Rita de Jacutinga	Bridge in tributary of Córrego da Lagoa, road Passa Vinte-Santa Rita do Jacutinga	-22.16722	-44.13000	Bananal
—	MZ969563	MNRJ	47336	CIC297	*A*. *oblongus*	BR-MG	Mar de Espanha	Dammed tributary of Córrego Cachoeirinha, in Fazenda Jardim	-21.92528	-42.97722	Paraíba do Sul
—	MZ969564	MNRJ	47336	CIC298	*A*. *oblongus*	BR-MG	Mar de Espanha	Dammed tributary of Córrego Cachoeirinha, in Fazenda Jardim	-21.92528	-42.97722	Paraíba do Sul
—	MZ969565	MNRJ	47336	CIC299	*A*. *oblongus*	BR-MG	Mar de Espanha	Dammed tributary of Córrego Cachoeirinha, in Fazenda Jardim	-21.92528	-42.97722	Paraíba do Sul
—	MZ969566	MNRJ	47365	CIC300	*A*. *oblongus*	BR-MG	Juiz de Fora	tributary of Rio do Peixe, between Toledo and Torreões	-21.82361	-43.58806	do Peixe
—	MZ969569	MNRJ	47367	CIC303	*A*. *oblongus*	BR-RJ	Sapucaia	Córrego Santa Rita in Santa Rita	-22.03417	-42.79667	Paquequer
—	MZ969570	MNRJ	47367	CIC304	*A*. *oblongus*	BR-RJ	Sapucaia	Córrego Santa Rita in Santa Rita	-22.03417	-42.79667	Paquequer
—	MZ969571	MNRJ	47367	CIC305	*A*. *oblongus*	BR-RJ	Sapucaia	Córrego Santa Rita in Santa Rita	-22.03417	-42.79667	Paquequer
—	MZ969572	MNRJ	47367	CIC306	*A*. *oblongus*	BR-RJ	Sapucaia	Córrego Santa Rita in Santa Rita	-22.03417	-42.79667	Paquequer
—	MZ969573	MNRJ	47367	CIC307	*A*. *oblongus*	BR-RJ	Sapucaia	Córrego Santa Rita in Santa Rita	-22.03417	-42.79667	Paquequer
—	MZ969574	MCP	48652	CIC308	*C*. *portalegrense*	BR-RS	Maquiné	Arroio Carvão	-29.53806	-50.24558	Tramandaí
—	MZ969576	MCP	21272	CIC31	*A*. *acaroides*	BR-RS	Ibarama	Stream tributary of Arroio Caidinho	-29.37722	-53.09889	Jacuí
—	MZ969577	MCP	49694	CIC310	*A*. *acaroides*	BR-RS	Bom Jesus	Stream tributary of Rio dos Touros ca 4 km NE of BR 285 (secundary road)	-28.68500	-50.21417	Pelotas
—	MZ969579	MCP	49984	CIC312	*A*. *acaroides*	BR-RS	Bom Jesus	Rio Manoel Leão on roadSilveira-Rondinha	-28.63556	-50.07853	Pelotas
—	MZ969580	MCP	48993	CIC313	*A*. *acaroides*	BR-RS	Bom Jesus	Rio dos Touros on road Rondinha-Pascoal, next to Usina Hidrelétrica Rio dos Touros	-28.64556	-50.28500	Pelotas
—	MZ969581	MCP	50385	CIC318	*A*. *acaroides*	BR-SC	Timbé do Sul	Rio Amola Faca, tributary of Rio Itoupava	-28.83986	-49.80000	Araranguá
—	MZ969582	MCP	42383	CIC32	*A*. *oblongus*	BR-MG	Taquaruçu de Minas	Small stream on road between Taquaruçu de Minas and Jabuticabas	-19.64000	-43.69806	São Francisco
—	MZ969586	MCP	50385_E1	CIC324	*A*. *acaroides*	BR-SC	Timbé do Sul	Rio Amola Faca, tributary of Rio Itoupava	-28.83972	-49.80000	Araranguá
—	MZ969589	MCP	51163_A	CIC327	*A*. *acaroides*	BR-RS	Cotiporã	Rio das Antas on road Faria Lemos-Cotiporã below Arroio Pedrinho	-29.08922	-51.63864	Antas
—	MZ969590	MCP	51163_B	CIC328	*A*. *acaroides*	BR-RS	Cotiporã	Rio das Antas on road Faria Lemos-Cotiporã below Arroio Pedrinho	-29.08922	-51.63864	Antas
—	MZ969591	MCP	51163_4	CIC329	*A*. *acaroides*	BR-RS	Cotiporã	Rio das Antas on road Faria Lemos-Cotiporã below Arroio Pedrinho	-29.08922	-51.63864	Antas
—	MZ969592	MCP	48464	CIC33	*A*. *acaroides*	BR-RS	Jaquirana	Parque Estadual Tainhas	-29.08478	-50.36603	Tainhas
—	MZ969593	MCP	51293_(36)	CIC330	*A*. *angiru*	BR-SC	Arabutã	Rio Jacutinga at camping MEC-SETTE near Arabutã (right margin)	-27.16122	-52.14833	Uruguay
—	MZ969595	MCP	48671	CIC35	*A*. *acaroides*	BR-RS	Bom Jesus	Rio das Antas at mouth of Arroio Moraes on road to Bom Jesus	-28.79833	-50.42889	Antas
—	MZ969596	MCP	48687	CIC36	*A*. *acaroides*	BR-RS	Bom Jesus	Rio das Antas at mouth of Rio Camisas on road Jaquirana-São José dos Ausentes	-28.84167	-50.30000	Antas
—	MZ969597	MCP	48666	CIC37	*A*. *acaroides*	BR-RS	São Francisco de Paula	Arroio Ribeirão on road RS 453 (Ruta do sol), tributary of Rio das Antas	-29.23278	-50.37472	Jacuí
—	MZ969598	UNV	1261	CIC85	*A*. *ipatinguensis*	BR-MG	Pingo d’Áqua	Lagoa Cristal, Pingo d’Água	-19.61736	-42.79599	Doce
—	MZ969599	UNV	1306	CIC86	*A*. *ipatinguensis*	BR-MG	Pingo d’Áqua	Lagoa Cristal, Pingo d’Água	-19.61736	-42.79599	Doce
—	MZ969600	UNV	1312	CIC87	*A*. *ipatinguensis*	BR-MG	Pingo d’Áqua	Lagoa Cristal, Pingo d’Água	-19.61736	-42.79599	Doce
—	MZ969601	DZSJR	1306	CIC93	*A*. *ipatinguensis*	BR-MG	Pingo d’Áqua	Pingo d’Áqua	-19.72786	-42.40840	Doce
—	MZ969602	MCP	53174_D1	D1	*A*. *acaroides*	BR-RS	Bom Jesus	Rio dos Touros, below barrage on road Rondinha São Joaquim	-28.64583	-50.28556	Pelotas

### Species delimitation methods

Species delimitation was tested with two methods. Geneious with the plug-in Species Delimitation [71] was used to obtain uncorrected pairwise genetic distances (*p*-distances), as recommended by Srivatsan and Meier [72]; Bayesian Poisson Tree Processes (bPTP) [73] was run via the bPTP web server (https://species.h-its.org/ptp/). MrBayes ‘-allcompat’ option was used to create fully resolved trees for analysis with bPTP.

### Phylogeny reconstruction

Phylogenetic analysis was performed with the parallel-computing version of MrBayes v3.3 [74, 75]. *Cichlasoma portalegrense* (Hensel, 1870) was designated outgroup in the *mt-coI* analyses; *Amphilophus citrinellus* (Günther, 1864) and *Neetroplus nematopus* Günther, 1867 in the *mt-cyb* analyses. Data was partitioned according to codon position (first, second, third) and parameters estimated separately for each partition. The GTR + Γ + I model was used as suggested by ModelTest2 [76]. Samples were taken every 1000 generations, and the first 25% of samples were discarded as ‘burn-in,. The analysis was run for ten million generations. Convergence was confirmed with MrBayes and Tracer v1.6 [77].

### Map and toponomy

Geographic coordinates for our own collecting sites were recorded in decimal degrees (DD), but were converted to degrees minutes seconds (DMS) for presentation in material lists. Coordinates from other collectors may have been in DD or DMS, or even decimal minutes (DM), and were converted for uniform presentation. The distribution maps were constructed with DD coordinates in QGis (https://qgis.org/) using free shape and raster files available from databases of ANA (Agência Nacional de Águas, Brazil: http://www.snirh.gov.br/hidroweb).

Some rivers are shared between Brazil and Argentina, or Brazil and Uruguay. We use the standard English names for basins and countries (Argentina, Paraguay, Uruguay, Paraná), but local names for shared rivers based on collecting site. The Rio Iguaçu is known as Iguazú in Argentina and Paraguay. Brazil has a political subdivision in regions and states. The regions and states concerned here are the Sul (Rio Grande do Sul, Santa Catarina, Paraná) Sudeste (São Paulo, Espírito Santo, Minas Gerais, Rio de Janeiro), and Nordeste (Bahia and other states). Ottoni and collaborators, e.g. Ottoni and Costa 2008 [6] consistently refer to southeastern Brazil in the sense of the Sudeste region. For that region, we use the name Sudeste to emphasise that it is an administrative unit. To simplify reference to two major geographical groups of species of *Australoheros*, we distinguish between the southern group (Sul region), and the northern group (Sudeste and Nordeste regions).

## Results

### Distinguishing characters of *Australoheros*

All species of *Australoheros* shared the following plesiomorphic character states at suprageneric levels: pelvic-fin rays I.5 (Acanthopterygii), circumpeduncular scales 16 (7 above and below lateral lines; most Cichlasomatinae and many other Cichlidae), principal caudal-fin rays 16 (Cichlidae, with rare exceptions); presence of six dark vertical bars on side (Bars 1a–6), stripes or blotches on the head and anterior side homologous with bars 7–9, and dark blotch on the caudal-fin base (Bar 1p) (Cichlasomatinae) [41, 58].

The morphological parsimony analysis by Kullander (1998 [41] recovered *Australoheros* (as ‘*Cichlasoma facetum*’ (actually based on specimens of both *A*. *acaroides* and *A*. *facetus*) in a clade of other South American heroine genera, but did not yield autapomorphies for the genus. Synapomorphies included the long and co-ossified hypurapophyses; the uncinate process of the first epibranchial wider than the anterior arm; and the lachrymal bone extensively overlapping the next infraorbital bone; none of those character states are unique for the genus. Říčan and Kullander [3] characterised *Australoheros* as distinct from other heroin cichlids by lower number of scales (modally less than 25 scales in the E1 row); lower vertebral count (13+13 or 13+14); chest scales nearly as large as flank scales, and two autapomorphies: (1) juveniles with unique xanthophore patch dorsally and ventrally on the caudal-fin base and (2) a unique breeding colour element: abdominal vertical bars (Bars 5 and 6) interrupted by a light horizontal area along the upper side. The latter colour characteristic is also expressed in non-breeding individuals. To those characteristics we add here the short skin projections from the genital papilla in mature males, a unique character among Neotropical cichlids. Tasseled male genital papilla was described by Trewavas [78] from some species of the African cichlid genus *Oreochromis* Günther, 1862.

### Nomenclature

The oldest name for a species in the genus *Australoheros* is *Chromis facetus* Jenyns, 1840. There is a recorded single preserved specimen representing the material used by Jenyns for the description of *C*. *facetus* in the collections of the Museum of Zoology, University of Cambridge, UMZC No F.6640. We were not successful in obtaining a photograph or X-radiograph of that specimen. Nevertheless, with precise provenance, it is uncontested as the holotype of *A*. *facetus*, which is the type species of *Australoheros*, fixed by Říčan and Kullander [3].

Ottoni et al. [47] suggested conditional synonymisation among species of *Australoheros* described by Ottoni and collaborators [5, 6, 8–10] from the Sudeste region. One analysis recognised *A*. *autrani*, *A*. *barbosae*, *A*. *muriae*, and *A*. *robustus* as valid species incorporating nominal species *A*. *robustus*, *A*. *mattosi*, *A*. *muriae*, *A*. *paraibae*, and *A*. *saquarema*. This clade is specified in their phylogram, fig. 2, which shows clades formed by *A*. *tavaresi*, *A*. *paraibae*, and *A*. *barbosae*; *A*. *mattosi*, and *A*. *robustus*; and *A*. *saquarema* and *A*. *autrani*. A second analysis recognised *A*. *autrani*, *A*. *barbosae*, *A*. *ipatinguensis*, and *A*. *robustus* as valid species incorporating nominal species *A*. *mattosi*, *A*. *muriae*, *A*. *perdi*, *A*. *paraibae*, *A*. *saquarema*, and *A*. *tavaresi*. The analysis illustrated with a bPTP tree (their fig. 3), in which *A*. *paraibae* and *A*. *tavaresi* are nested with *A*. *barbosae*; *A*. *ipatinguensis*, with *A*. *perdi*; *A*. *saquarema* with *A*. *autrani*; and *A*. *robustus* with *A*. *mattosi*. There is no explicit statement concerning relative priority, and for both analyses, the selected species name for each analysis is explained with respect to ‘following chronological priority for zoological names… hereafter called *A*. *autrani*, *A*. *barbosae*, and *A*. *robustus* [first analysis]/*A*. *autrani*, *A*. *ipatinguensis*, and *A*. *robustus*’[second analysis].

Among recently described nominal species of *Australoheros* from the Sudeste, the species described by Ottoni and Costa in 2008 [6] and Ottoni in 2010 [8] have priority over any later described species, and the option of first reviser action on precedence among simultaneous synonyms is restricted to the 2008 and [6] 2012 [9] names. Because Ottoni et al. [47] (a) provided two scenarios for possible fixation of precedence of names, and used the expression ‘hereafter called …’ and (b) on p. 57 proposed a hybrid delimitation (‘congruence of these species delimitation methods (bPTP, WP, CBB…’), there is ambiguity in what taxa they were considering. To overcome this ambiguity, we select here *A*. *paraibae* to have priority over *A*. *barbosae* whenever the two are considered to be the same species; *A*. *autrani* to have priority over *A*. *saquarema* whenever the two are considered to be the same species; *A*. *ipatinguensis* to have priority over *A*. *autrani* whenever the two are considered to be the same species; *A*. *ipatinguensis* to have priority over *A*. *muriae* whenever the two are considered to be the same species; *A*. *tavaresi* to have priority over *A*. *montanus* whenever the two are considered to be the same species; and *A*. *montanus* to have priority over *A*. *mattosi* whenever the two are considered to be the same species. Our analysis confirmed only three species for the Sudeste region, of which one represents *A*. *oblongus*. The other species are *A*. *ipatinguensis* and *A*. *ribeirae*.

### Species diversity

Based on the morphological and genetic analysis reported below, we recognise 18 valid, formally named species in *Australoheros*, nine of which pertain to coastal rivers (*A*. *acaroides*, *A*. *facetus*, *A*. *ipatinguensis*, *A*. *minuano*, *A*. *oblongus*, *A*. *ribeirae*, *A*. *sanguineus*, *and* two species formally named herein). The remaining nine species, presented in S2 File are entirely or primarily restricted to the middle and upper Rio Uruguay and tributaries of the lower Rio Paraná. Author and suggested synonymy are listed in Table 2. In addition, we recognise distinctive units that may represent distinct species, but for which supporting evidence was insufficient. Those include *A*. sp. “Jacuí”, already distinguished as *A*. sp. jacui by Říčan and Kullander [3] and as *A*. sp. “Jacui” by Říčan and Kullander [4]; a genetically distinct sample of *A*. *minuano* or *A*. *facetus*, here referred to as *Australoheros*. “Arapey”; and samples probably representing *A*. *sanguineus*, but then only based on the distribution records, and referred to as *Australoheros* sp. cf. *sanguineus*.

**Table 2:** Nominal species of *Australoheros* in order of description, and recommended synonymy.

Current name	Original name	Author	Year of description	Current combination
*Australoheros facetus*	*Chromis facetus*	Jenyns	1842	*Australoheros facetus*
*Australoheros facetus*	*Chromys oblonga*	Castelnau	1855	*Australoheros oblongus*
*Australoheros facetus*	*Heros autochthon*	Günther	1862	*Australoheros oblongus*
*Australoheros facetus*	*Heros Jenynsii*	Steindachner	1869	*Australoheros facetus*
*Australoheros facetus*	*Heros acaroides*	Hensel	1870	*Australoheros acaroides*
*Australoheros tembe*	*’Cichlasoma’ tembe*	Casciotta, Gómez & Toresani	1995	*Australoheros tembe*
*Australoheros scitulus*	*’Cichlasoma’ scitulum*	Říčan & Kullander	2003	*Australoheros scitulus*
*Australoheros kaaygua*	*Australoheros kaaygua*	Casciotta, Almirón & Gómez	2006	*Australoheros kaaygua*
*Australoheros forquilha*	*Australoheros forquilha*	Říčan & Kullander	2008	*Australoheros forquilha*
*Australoheros charrua*	*Australoheros charrua*	Říčan & Kullander	2008	*Australoheros charrua*
*Australoheros minuano*	*Australoheros minuano*	Říčan & Kullander	2008	*Australoheros minuano*
*Australoheros guarani*	*Australoheros guarani*	Říčan & Kullander	2008	*Australoheros guarani*
*Australoheros ribeirae*	*Australoheros ribeirae*	Ottoni, Oyakawa & Costa	2008	*Australoheros ribeirae*
*Australoheros autrani*	*Australoheros autrani*	Ottoni & Costa	2008	*Australoheros ipatinguensis*
*Australoheros barbosae*	*Australoheros barbosae*	Ottoni & Costa	2008	*Australoheros oblongus*
*Australoheros ipatinguensis*	*Australoheros ipatinguensis*	Ottoni & Costa	2008	*Australoheros ipatinguensis*
*Australoheros macacuensis*	*Australoheros macacuensis*	Ottoni & Costa	2008	*Australoheros oblongus*
*Australoheros macaensis*	*Australoheros macaensis*	Ottoni & Costa	2008	*Australoheros ipatinguensis*
*Australoheros muriae*	*Australoheros muriae*	Ottoni & Costa	2008	*Australoheros ipatinguensis*
*Australoheros paraibae*	*Australoheros paraibae*	Ottoni & Costa	2008	*Australoheros oblongus*
*Australoheros robustus*	*Australoheros robustus*	Ottoni & Costa	2008	*Australoheros oblongus*
*Australoheros saquarema*	*Australoheros saquarema*	Ottoni & Costa	2008	*Australoheros ipatinguensis*
*Australoheros capixaba*	*Australoheros taura*	Ottoni & Cheffe	2009	*Australoheros acaroides*
*Australoheros capixaba*	*Australoheros acaroides*	Ottoni	2010	*Australoheros ipatinguensis*
*Australoheros perdi*	*Australoheros perdi*	Ottoni, Lezama, Triques, Fragoso-Moura, Lucas & Barbosa	2011	*Australoheros ipatinguensis*
*Australoheros ykeregua*	*Australoheros ykeregua*	Říčan, Piálek, Almirón & Casciotta	2011	*Australoheros ykeregua*
*Australoheros angiru*	*Australoheros angiru*	Říčan, Piálek, Almirón & Casciotta	2011	*Australoheros angiru*
*Australoheros mattosi*	*Australoheros mattosi*	Ottoni	2012	*Australoheros oblongus*
*Australoheros montanus*	*Australoheros montanus*	Ottoni	2012	*Australoheros oblongus*
*Australoheros tavaresi*	*Australoheros tavaresi*	Ottoni	2012	*Australoheros oblongus*
*Australoheros sanguineus*	*Australoheros sanguineus*	Ottoni	2013	*Australoheros sanguineus*
*Australoheros ricani*	—	Present paper	2022	*Australoheros ricani*
*Australoheros mboapari*	*—*	Present paper	2022	*Australoheros mboapari*

### Meristics

Meristic frequency data for most of the species of *Australoheros* are presented in Tables 3–9.

**Table 3:** Number of vertebrae in species *of Australoheros*.

Vertebrae	12+13	12+14	12+15	13+12	13+13	13+14	13+15	14+12	14+13	14+14
*A*. *acaroides*				1	**20**	4				
*A*. *angiru*				2	**21**	1				
*Heros autochthon* (ST)					3					
*A*. *minuano* (Uruguay)	2	1		1	**6**	5				
*A*. *acaroides* (Pinares)					**45**	2				
*A*. *charrua*				1	1	3				1
*A*. *facetus*					**23**	6				
*A*. *forquilha*						**4**	2			
*A*. *guarani*					6					
*A*. *ipatinguensis*				3	**19**					
*A*. *kaaygua*					1					
*A*. *mboapari*						1				
*A*. *minuano* (Brazil)		1		1	**8**					
*A*. *oblongus (*HT)					1					
*A*. *oblongus*	2			2	**28**			2		
*A*. sp. “Jacuí”				2	**16**					
*A*. *sanguineus*				2	**5**					

Modal frequencies marked in bold for samples with N> 5. HT = Holotype; ST = Syntypes.

**Table 4:** Number of scales along the middle of the side (E1 row scales) in species *of Australoheros*.

	23	24	25	26	27	Total
*A*. *facetus* (Portugal)		**1**	**8**			9
*A*. *acaroides*	3	**74**	41			122
*A*. *angiru*	1	**13**	4			18
*Heros*. *autochthon*		**3**				3
*A*. *minuano* (Uruguay)	1	10	**12**	1		24
*A*. *“*Arapey*”*		**2**	1			3
*A*. cf. *sanguineus*		**4**				4
*A*. *acaroides* (Pinares)	1	**24**	16	1		42
*A*. *charrua*			**6**	2		8
*A*. *facetus*		**31**	15	1		47
*A*. *forquilha*				**9**		9
*A*. *guarani*		**8**				8
*A*. *ipatinguensis*	21	**42**				64
*A*. *kaaygua*		1	1	1		3
*A*. *mboapari*			7	**17**	4	28
*A*. *minuano (*Brazil)		**4**	1			5
*A*. *oblongus*		**1**				1
*A*. *oblongus*	10	**69**	2			81
*A*. *ribeirae*	2	**3**				5
*A*. sp. “Jacuí”		**10**	3			
*A*. *ricani*			**7**	6		13
*A*. *sanguineus*	2	**16**	1			19
*A*. *scitulus*		6	**28**	3		37
*A*. *tembe*			**6**			6

Modal frequencies marked in bold for samples with N> 5. HT = Holotype; ST = Syntypes.

**Table 5:** Number of lateral line scale in species *of Australoheros*.

upper	12	13		14				15					16							17						18						19					20	
lower	8	8	9	7	8	9	10	6	7	8	9	10	5	6	7	8	9	10	12	5	6	7	8	9	10	6	7	8	9	10	11	7	8	9	10	13	9	10
*A*. *acaroides*				1	2		1	2		4	8	3		1	3	7	14	6			1	4	18	18	5		4	5					1					
*A*. *acaroides* (Pinares)		2	1			1			2	5	3	1			2	6	7	1				1	4	3				2	1									
*A*. *angiru*																						1	1	1			2	1	3									
*A*. cf. *sanguineus*																1						3																
*A*. *charrua*																							3	2	1				1			1						
*A*. *facetus*				1				1			1			1		3	8	2				2	6	6	4			5	1				1	1	1		1	1
*A*. *facetus* (Portugal)																	3	1					2	2				1										
*A*. *forquilha*																								1	1			3	1	1			1	1				
*A*. *guarani*														1			1					1	3				1											
*A*. *ipatinguensis*				1	1						1		1	1	8	10	3		1	1	1	4	5	1			4	2										
*A*. *kaaygua*																								1				1		1								
*A*. *mboapari*																		1						2	2	1			5	6	1			3	4			1
*A*. *minuano* (Brazil)						1											2	1											1									
*A*. *minuano* (Uruguay)	1							1				1		2		5	4			1	1	1	1	1				1	3							1		
*A*. *oblongus*								1	2	3					11	15	4			1	3	14	9	1		1	7	2	1				1					
*A*. *oblongus* HT																																						
*A*. *ribeirae*										1						2							1															
*A*. *ricani*															1	1	3							4	1		2	4	1									
*A*. *sanguineus*				1	1										1	2	1				2	1	3	1			1	2					1					
*A*. *scitulus*										1						2	2	1			1	1	3	7	4	2	2	5	3	1				1				
*A*. sp. "Jacuí"												2				1		1				1	1	3										2	1			
*A*. *tembe*																	1						2	1	1													
*H*. *autochthon* ST										1					1								1															

Modal frequencies marked in bold for samples with N> 5. HT = Holotype; ST = Syntypes.

**Table 6:** Number of dorsal-fin rays in species of *Australoheros*.

Spines	XIV	XIV	XIV	XV	XV	XV	XV	XV	XV	XVI	XVI	XVI	XVI	XVI	XVII	XVII	XVII	XVII	XVII	XVII
Soft rays	9	11	12	8	9	10	11	12	13	5	7	8	9	10	11	7	8	9	10	11
*A*. "Arapey"							1						1	1						
*A*. *acaroides*				1	4	12	3				1	3	42	**45**	4		4	9	3	
*A*. *acaroides* (Pinares)						2	1						16	**17**	2		1	9	2	
*A*. *angiru*						1	1				1		**20**	9			1	1		
*A*. *cf*. *sanguineus*							1						1	1					1	
*A*. *charrua*													1	3				1	2	
*A*. *facetus*						1	1	1				2	5	**36**	4				1	
*A*. *facetus* (Portugal)						3	2							3	1					
*A*. *forquilha*							1							3	6					
*A*. *guarani*						1								5				2		
*A*. *ipatinguensis*		1		1	4	18	7		1			2	7	**28**	2					
*A*. *kaaygua*					1								1	1						
*A*. *mboapari*													3	**12**	9				2	2
*A*. *minuano* (Brazil)						2							1	6	1					
*A*. *minuano* (Uruguay)	1				1	3	3						1	13				2		
*A*. *oblongus*					1	5	1			1		2	25	**39**	8	1	9	15	2	
*A*. *oblongus* HT														1						
*A*. *ribeirae*					1								1	1	1		1			
*A*. *ricani*			1			3	4							4				1		
*A*. *sanguineus*					4	2	1					2	4	**6**						
*A*. *scitulus*													4	2		1	6	32	4	
*A*. sp. “Jacuí”			1		2	14	1											1		
*A*. *tembe*												1	**5**							
*A*. *ykeregua*					1		2					1	1	**7**	5		1			
*H*. *autochthon* ST													1	2						

Modal frequencies marked in bold for samples with N> 5. HT = Holotype; ST = Syntypes.

**Table 7:** Number of anal-fin rays in species of *Australoheros*.

Spines	V	V	V	V	V	VI	VI	VI	VI	VI	VII	VII	VII	VII	VII	VIII	VIII	VIII	VIII	IX	IX	IX
Soft rays	6	7	8	9	10	6	7	8	9	10	6	7	8	9	10	6	7	8	9	6	7	8
*A*. “Arapey”							1	1														
*A*. *acaroides*	1						1	15	14	2	1	20	**58**	9			4	5	1			
*A*. *acaroides* (Pinares)								3	3			12	**22**	2	2		2	3	1			
*A*. *angiru*							2	4				12	**14**			1						
*A*. cf. *sanguineus*									1				1	2								
*A*. *charrua*												3	5									
*A*. *facetus*				2			3		**11**			6	4									
*A*. *facetus* (Portugal)				1				3	5													
*A*. *forquilha*								6	2				2									
*A*. *guarani*								6	1				1									
*A*. *ipatinguensis*						1	1	4	14	1	1		12	**25**	4		1	4	2			
*A*. *kaaygua*						1		1	1													
*A*. *mboapari*			2	1	1		2	**18**	3			1										
*A*. *minuano* (Brazil)								2	3	1			1									
*A*. *minuano* (Uruguay)							6	**17**				1										
*A*. *oblongus*							1	1	3		1	14	**45**	10		3	18	9	3			
*A*. *oblongus* HT													1									
*A*. *ribeirae*									1				3				1					
*A*. *ricani*				3	1			**5**	4													
*A*. *sanguineus*												5	**9**	4								
*A*. *scitulus*																	**14**	10	1	6	18	1
*A*. sp. “Jacuí”			4	9			1	3														
*A*. *tembe*							5				1											
*A*. *ykeregua*		1					7	**8**														
*H*. *autochthon* ST													2	1								

Modal frequencies marked in bold for samples with N> 5. HT = Holotype; ST = Syntypes.

**Table 8:** Number of pectoral-fin rays in species of *Australoheros*.

	11	12	13	14	15
*A*. *facetus* (Portugal)		1	2	**6**	
*A*. *acaroides*		21	**84**	6	
*A*. *angiru*		**5**	**5**		
*Heros autochthon* ST		1	**3**		
*A*. *minuano* (Uruguay)		3	**21**		
*A*. “Arapey”			**3**		
*A*. cf. *sanguineus*		2	**2**		
*A*. *acaroides* (Pinares)		6	**33**	3	
*A*. *charrua*		1	7		
*A*. *facetus*		3	**18**	15	11
*A*. *forquilha*			**6**	3	
*A*. *guarani*		1	**7**		
*A*. *ipatinguensis*	1	15	**42**	3	
*A*. *kaaygua*		1	**2**		
*A*. *mboapari*		1	**16**	11	
*A*. *minuano* (Brazil)		2	**3**		
*A*. *oblongus*		10	**57**	12	
*A*. *ribeirae*		1	**4**		
*A*. sp.” Jacuí”		3	**13**	9	
*A*. *ricani*			3	**9**	
*A*. *sanguineus*		1	**15**		
*A*. *scitulus*		2	**26**	9	
*A*. *tembe*			**4**	2	

Modal frequencies marked in bold for samples with N> 5. ST = Syntypes.

**Table 9:** Counts of ceratobranchial gill rakers in species of *Australoheros*.

	5	6	7	8	9
*A*. “Arapey”			1	2	
*A*. *acaroides* (Pinares*)*			**12**	16	
*A*. *acaroides*	1	13	**75**	17	
*A*. *angiru*	1	**8**	1		
*A*. cf. sanguineus		1	3		
*A*. *charrua*	1	**5**	2		
*A*. *facetus*		1	20	**26**	
*A*. *facetus* (Portugal)	1		1	**5**	2
*A*. *forquilha*			4	5	
*A*. *guarani*			**7**		
*A*. *ipatinguensis*		6	**25**	15	1
*A*. *kaaygua*		1	1		
*A*. *mbapoari*	3	**24**	1		
*A*. *minuano* (Brazil)	1	6	1		
*A*. *minuano* (Uruguay)	1	2	15		
*A*. *oblongus*		7	**56**	18	
*A*. *oblongus* HT			1		
*A*. *ribeirae*		1	3		
*A*. *ricani*			5	**7**	1
*A*. *sanguineus*		**6**	4		
*A*. *scitulus*	4	**25**	6		
*A*. sp. "Jacuí”		6	2	**5**	
*A*. *tembe*	1	2	1	1	
*H*. *autochthon* ST			1	1	1

Modal frequencies marked in bold. HT = Holotype; ST = Syntypes.

The modal number of vertebrae was 13+13, and a modal total of 26 in most species (Table 3). Higher numbers prevailed in *A*. *scitulus*, *A*. *tembe*, *A*. *ykeregua*, *A*. *charrua*, and *A*. *forquilha*, typically with 14 or 15 caudal vertebrae. Only one observation was made on *A*. *mboapari*. Vertebral data were not available for *A*. *ribeirae*.

The number of scales along the middle of the side (E1 row scales) (Table 4) was relatively constant, with a common modal number of 24 scales, some species with a short range; but only *A*. *mboapari* standing out with 25–27 scales. The lowest numbers were obtained from very small specimens, and may reflect a systematic error. *Australoheros scitulus*, *A*. *tembe*, *A*. *ricani*, and *A*. *forquilha* had more than 24 E1 scales. Most of the specimens of *A*. sp “Jacuí” had 24 E1 scales, contrasting with 25–26 in the sympatric *A*. *ricani* specimens. Low numbers in *A*. *ipatinguensis* distinguished it from *A*. *oblongus* (Mann-Whitney U test, p = 0.001), but in specimens over 40 mm SL, the difference was reduced (p = 0.051).

The number of lateral line scales was were generally between 16 and 20 (average 17) in the upper lateral line and 6–10 (average 8) in the lower lateral line (Table 5).

The number of dorsal-fin rays (Table 6) were modally XVI.10. *Australoheros* sp. “Jacuí” was exceptional in having modally XV.10, and only four out of 18 specimens with 16 spines. In *A*. *ipatinguensis* 39% of the specimens had XV.10, making for an almost bimodal distribution in *A*. *ipatinguensis*, with XVI.10 the most frequent number, shared with *A*. *oblongus A*. *tembe*, *A*. *sanguineus*, *A*. *minuano*, *A*. *mboapari*, *A*. *guarani*, and *A*. *facetus*. In *A*. *acaroides*, XVI.9 and XVI.10 were almost equally frequent. Although with distinct modal of XV.10, *A*. *ipatinguensis* and *A*. *oblongus* showed very wide variation in scattered counts. Several species had 17 dorsal-fin spines but the count was frequent only in *A*. *oblongus* and *A*. *scitulus*, the latter with modally XVII.9, representing the greatest number of dorsal-fin rays for the genus.

The number of anal-fin rays (Table 7) showed mainly within-species variation. Well-represented species had modals VII.8 (*A*. *acaroides* and *A*. *ipatinguensis*). Only four species are represented by specimens with 5 anal-fin spines, the remainder typically 6–7. *Australoheros* sp. Jacuí” was outstanding with a high frequency of 5 anal-fin spines, whereas 5 spines was an exceptional number in *A*. *facetus* and *A*. *ykeregua*. *Australoheros mboapari* and *A*. *tembe* had exclusively or modally 6 anal-fin spines (one specimen of *A*. *tembe* with 7). Eight anal-fin spines were relatively frequent in *A*. *oblongus*, *ipatinguensis*, *A*. *ribeirae*, and *A*. *sanguineus*, and prevalent in *A*. *scitulus*, which was the only species in the genus with frequently 9 anal-fin spines. *Australoheros ipatinguensis* and *A*. *oblongus* had the same mode (7) but different frequencies of anal-fin spines (predominantly 6*–*7, and 7–8, respectively).

The number of pectoral-fin rays (Table 8) showed a clear mode of 13 in all species.

Gill raker counts (Table 9) showed little variation, predominantly 6 or 7. Říčan and Kullander [4] considered variation in gill-raker numbers as useful for delimiting species, but the considerable overlap in our data did not meet that expectation.

Counts of predorsal scales, teeth and rows of scales on the cheek are presented in the species accounts only, as no comparative statistics was attempted for those. The count of predorsal scales had limited reproducibility; the scales were slightly irregularly arranged and usually it would have been necessary to remove the skin to expose the scales properly, and this was not done. In many specimens the cheek scales were exposed and easy to count, but often the scales were covered beneath a thin skin layer, and defied confirmation of a correct count. Large specimens had more rows of cheek scales than small specimens, but no reliable data is available for analysis as we deemed it more valuable to leave the skin layer intact than to sacrifice it for scale counts. No variation species specific condition was found in the count of teeth; the number varied with specimen size, and in specimens with jaws well fixed, it was difficult or not recommended to open the mouth with force to observe the teeth. All specimens examined had 16 circumpeduncular scales.

### Morphometrics

Distance measurements are reported for each species in Tables 10–19. Linear regression on standard length was established for all measurements with sample N> 5). Biplots of each measurement against standard length did not show any variation suggesting different species. In general, the biplot scatters showed more variation among larger specimens than smaller specimens, suggesting that larger specimens had been more exposed to factors affecting body shape over time, or that there was a shift in relative allometry in larger specimens, e.g. reduced growth along the long axis.

**Table 10:** *Australoheros facetus*. Proportional measurements (per cent of SL) and linear regression parameters.

	N	Min	Max	Mean	SD	r	a	b
SL (mm)	47	23.8	147.4	89.9	30.0			
Head length	47	33.2	38.6	35.6	1.5	0.99	0.271	0.352
Snout length	47	9.9	15.3	12.9	1.3	0.99	-2.240	0.158
Body depth	47	45.1	54.0	49.3	2.5	0.99	-3.770	0.534
Orbital diameter	47	8.0	14.3	10.4	1.3	0.97	2.034	0.078
Head width	47	17.2	21.7	18.8	1.0	0.99	-1.100	0.202
Interorbital width	47	9.7	15.1	12.3	1.3	0.99	-2.809	0.150
Preorbital depth	47	4.6	8.7	7.0	1.0	0.99	-1.759	0.092
Upper jaw length	47	9.5	13.2	11.1	0.9	0.99	-1.768	0.133
Lower jaw length	47	13.1	17.3	15.1	1.1	0.98	-0.894	0.162
Caudal-peduncle depth	47	16.5	21.0	18.7	1.1	0.99	-1.393	0.205
Caudal-peduncle length	47	5.4	11.1	8.0	1.1	0.92	0.120	0.078
Pectoral-fin length	46	22.3	37.4	30.1	2.6	0.97	-2.240	0.013
Pelvic-fin length	47	26.5	42.6	33.8	4.7	0.98	-8.723	0.449
Last dorsal-fin spine length	44	14.3	21.4	18.0	1.6	0.96	-0.460	0.187

**Table 11:** *Australoheros oblongus*. Proportional measurements (per cent of SL) and linear regression parameters.

	N	Min	Max	Mean	SD	r	a	b
SL (mm)	78	38.3	113.0	64.5	15.6			
Head length	78	33.8	40.0	37.0	1.4	0.99	1.787	0.341
Snout length	78	10.2	16.0	12.2	1.0	0.97	-1.694	0.149
Body depth	78	41.7	53.6	46.9	2.6	0.98	-3.256	0.522
Orbital diameter	78	9.7	15.3	12.3	1.2	0.92	2.694	0.079
Head width	78	17.3	20.9	19.0	0.8	0.99	-0.532	0.199
Interorbital width	78	10.3	14.6	12.2	0.9	0.98	-1.851	0.152
Preorbital depth	78	4.6	7.6	5.8	0.6	0.97	-1.381	0.080
Upper jaw length	78	9.4	12.9	11.3	0.7	0.98	-0.833	0.127
Lower jaw length	78	14.1	18.1	15.9	0.9	0.97	-0.133	0.161
Caudal-peduncle depth	78	15.7	21.3	18.3	1.1	0.98	-1.569	0.208
Caudal-peduncle length	78	5.0	9.4	6.6	0.8	0.86	0.509	0.058
Pectoral-fin length	78	26.2	36.8	31.0	1.8	0.97	-0.864	0.324
Pelvic-fin length	77	28.2	54.3	38.9	5.9	0.94	10.126	0.555
Last dorsal-fin spine length	76	16.0	22.8	19.2	1.4	0.97	-1.526	0.217

**Table 12:** *Australoheros acaroides*. Size (SL), proportional measurements (per cent of SL), and linear regression parameters.

	N	Min	Max	Mean	SD	r	a	b
SL (mm)	105	30.1	114.4	68.9	18.9			
Head length	105	32.5	40.0	35.7	1.3	0.99	1.794	0.289
Snout length	105	8.7	16.5	12.9	1.4	0.97	-2.05	0.161
Body depth	107	35.2	54.2	45.9	2.9	0.98	-1.061	0.476
Orbital diameter	105	7.9	14.4	11.0	1.2	0.95	2.247	0.002
Head width	105	16.1	20.7	18.2	1.0	0.98	0.191	0.179
Interorbital width	105	9.2	14.3	11.6	1.1	0.97	-1.591	0.141
Preorbital depth	105	4.1	9.1	6.9	1.0	0.98	-1.843	0.098
Upper jaw length	104	8.6	13.0	10.8	0.8	0.98	-0.8	0.121
Lower jaw length	104	12.7	16.4	14.8	0.8	0.98	2.96	0.143
Caudal-peduncle depth	105	15.5	19.9	17.4	0.9	0.99	-1.093	0.191
Caudal-peduncle length	105	6.4	10.8	8.6	0.9	0.95	-0.957	0.101
Pectoral-fin length	105	20.3	31.6	27.6	1.8	0.97	0.588	0.266
Pelvic-fin length	105	25.0	36.2	29.6	2.3	0.97	-1.732	0.232
Last dorsal-fin spine length	104	12.7	20.4	17.2	1.6	0.92	0.355	0.167

**Table 13:** *Australoheros minuano* from Uruguay. Size (SL), proportional measurements (per cent of SL), and linear regression parameters.

	N	Min	Max	Mean	SD	r	a	b
SL (mm)	18	36.8	92.9	56.2	19.1			
Head length	18	34.0	37.7	35.3	1.0	1.00	0.734	0.339
Snout length	18	9.9	14.0	11.6	1.2	0.99	-1.745	0.150
Body depth	18	42.7	47.0	44.7	1.3	1.00	-0.091	0.448
Orbital diameter	18	9.7	13.4	11.6	1.1	0.98	1.814	0.081
Head width	18	17.0	19.4	18.1	0.6	1.00	0.014	0.181
Interorbital width	18	9.8	12.6	11.0	0.8	0.99	-1.283	0.135
Preorbital depth	18	4.9	7.9	6.0	1.0	0.99	-1.712	0.093
Upper jaw length	18	8.6	11.2	9.7	0.8	1.00	-1.37	0.100
Lower jaw length	18	12.4	14.6	13.2	0.7	0.99	-0.073	0.133
Caudal-peduncle depth	18	15.3	18.2	16.9	0.7	0.99	-0.279	0.174
Caudal-peduncle length	18	7.9	10.3	9.2	0.8	0.98	-0.417	0.100
Pectoral-fin length	18	24.5	30.0	27.9	1.4	1.00	-0.971	0.299
Pelvic-fin length	18	25.8	29.8	27.3	1.1	1.00	-1.45	0.301
Last dorsal-fin spine length	18	15.2	18.8	16.6	0.9	0.98	-0.497	0.176

**Table 14:** *Australoheros ribeirae*. Size (SL) and proportional measurements (per cent of SL).

	N	Min	Max	Mean	SD
SL (mm)	4	63.4	79.9	70.3	6.9
Head length	4	35.3	36.4	35.9	0.5
Snout length	4	11.2	14.5	12.7	1.3
Body depth	4	45.3	49.6	47.6	2.2
Orbital diameter	4	10.8	12.0	11.3	0.5
Head width	4	18.8	19.6	19.1	0.4
Interorbital width	4	12.5	13.0	12.9	0.2
Preorbital depth	4	6.4	7.0	6.7	0.2
Upper jaw length	4	9.5	12.4	11.0	1.2
Lower jaw length	4	10.9	15.7	14.2	2.2
Caudal-peduncle depth	4	17.7	19.2	18.4	0.8
Caudal-peduncle length	4	7.1	8.5	7.6	0.6
Pectoral-fin length	4	26.1	30.8	28.5	2.0
Pelvic-fin length	4	28.5	36.0	33.4	3.4
Last dorsal-fin spine length	4	16.2	19.9	17.6	1.6

**Table 15:** *Australoheros ipatinguensis*. Size (SL), proportional measurements (per cent of SL), and linear regression parameters.

	N	Min	Max	Mean	SD	r	a	B
SL (mm)	52	26.8	97.8	56.0	17.6			
Head length	52	34.2	41.4	37.5	1.3	0.99	1.098	0.132
Snout length	52	9.0	13.6	11.8	1.0	0.98	-0.720	0.132
Body depth	52	44.7	53.1	48.9	2.2	0.99	-2.063	0.530
Orbital diameter	52	10.1	15.9	13.0	1.3	0.97	2.019	0.090
Head width	52	17.4	21.3	18.9	0.8	0.99	-0.819	0.205
Interorbital width	52	10.4	14.7	12.0	0.9	0.99	-1.337	0.146
Preorbital depth	52	3.5	7.4	5.5	0.8	0.98	-1.179	0.078
Upper jaw length	52	9.0	12.4	10.7	0.7	0.99	-1.096	0.126
Lower jaw length	52	13.5	17.3	15.4	0.7	0.99	-0.630	0.155
Caudal-peduncle depth	52	17.2	20.4	18.7	0.8	0.99	0.429	0.061
Caudal-peduncle length	52	5.3	8.8	7.0	0.9	0.91	-0.146	0.341
Pectoral-fin length	50	28.5	34.4	31.2	1.4	0.99	-1.456	0.341
Pelvic-fin length	52	29.9	52.0	36.1	5.0	0.97	-7.655	0.510
Last dorsal-fin spine length	52	16.4	22.7	20.3	1.2	0.98	-0.639	0.217

**Table 16:** *Australoheros sanguineus*. Size (SL), proportional measurements (per cent of SL), and linear regression parameters.

	N	Min	Max	Mean	SD	r	a	b
SL	16	40.2	84.2	62.0	14.6			
Head length	16	34.8	41.3	37.1	1.9	0.98	3.187	0.316
Snout length	16	9.7	14.0	12.5	1.2	0.98	-1.446	0.150
Body depth	16	45.4	55.0	50.3	2.8	0.99	-3.671	0.565
Orbital diameter	16	10.2	14.2	12.0	1.4	0.90	2.366	0.080
Head width	16	18.0	21.3	19.4	0.9	0.98	0.790	0.180
Interorbital width	16	11.8	15.6	13.2	1.0	0.97	-0.638	0.143
Preorbital depth	16	5.1	7.5	6.5	0.7	0.97	-1.013	0.083
Upper jaw length	16	9.7	14.1	11.3	1.0	0.93	0.685	0.102
Lower jaw length	16	13.6	17.6	15.2	1.0	0.96	1.148	0.010
Caudal-peduncle depth	16	18.0	20.5	19.2	0.8	0.98	0.184	0.189
Caudal-peduncle length	15	5.8	8.9	6.8	0.8	0.90	-0.563	0.078
Pectoral-fin length	16	28.0	34.3	31.1	1.9	0.95	1.161	0.291
Pelvic-fin length	15	30.9	42.7	35.8	4.0	0.95	-5.144	0.447
Last dorsal-fin spine length	15	17.5	22.5	19.5	1.4	0.95	0.977	0.179

**Table 17:** *Australoheros mboapari*. Size (SL), proportional measurements (per cent of SL) and linear regression parameters.

	HT	N	Min	Max	Mean	SD	r	a	b
SL	101.7	16	62.1	107.9	85.3	14.6			
Head length	32.7	16	32.0	34.5	33.3	0.6	1.00	-0.4070	0.338
Snout length	16.3	16	13.5	16.3	15.0	0.9	0.99	-3.6250	0.194
Body depth	43.6	16	40.7	45.8	43.9	1.4	0.99	-4.8930	0.498
Orbital diameter	8.8	16	8.7	9.8	9.3	0.4	0.96	1.062	0.080
Head width	16.8	16	15.6	18.0	17.1	0.6	0.98	-1.574	0.190
Interorbital width	11.6	16	9.3	12.7	11.1	0.9	0.98	-3.485	0.153
Preorbital depth	9.1	16	7.4	9.7	8.8	0.8	0.97	-2.571	0.119
Upper jaw length	9.7	16	8.8	10.9	9.8	0.6	0.97	-1.655	0.118
Lower jaw length	12.4	16	11.7	14.2	13.0	0.7	0.96	2.432	0.101
Caudal-peduncle depth	16.4	16	15.5	18.0	16.9	0.7	0.98	-1.009	0.182
Caudal-peduncle length	10.7	16	7.7	11.2	9.3	1.0	0.89	-1.527	0.111
Pectoral-fin length	24.2	16	23.7	28.7	26.4	1.6	0.96	-3.71	0.309
Pelvic-fin length	30.2	16	24.5	34.8	29.9	3.4	0.97	-12.323	0.448
Last dorsal-fin spine length	13.4	15	13.4	17.5	15.3	1.1	0.90	1.584	0.134

HT = Holotype.

**Table 18:** *Australoheros ricani*. Size (SL), proportional measurements (per cent of SL) and linear regression parameters.

	HT	N	Min	Max	Mean	SD	r	a	b
SL (mm)	75.5	13	56.9	109.9	78.5	15.1			
Head length	34.7	13	33.3	36.5	34.7	1.0	0.99	1.789	0.323
Snout length	13.5	13	12.5	15.0	13.8	0.8	1.00	-2.895	0.176
Body depth	44.9	13	40.5	44.9	42.0	1.4	0.99	1.903	0.395
Orbital diameter	10.2	13	8.7	10.7	9.9	0.6	0.97	2.478	0.067
Head width	16.8	13	15.7	17.4	16.4	0.5	0.99	0.274	0.161
Interorbital width	9.5	13	8.6	10.8	9.7	0.6	0.98	-1.812	0.127
Preorbital depth	7.0	13	6.9	8.6	7.6	0.6	0.99	-2.329	0.361
Upper jaw length	10.9	13	10.2	11.5	10.9	0.4	0.99	0.373	0.114
Lower jaw length	14.3	13	13.3	15.7	14.3	0.6	0.98	0.771	0.133
Caudal-peduncle depth	17.4	13	15.2	17.5	16.2	0.7	0.98	1.099	0.177
Caudal-peduncle length	11.8	13	8.0	11.8	10.5	0.9	0.91	0.119	0.103
Pectoral-fin length	25.8	13	22.5	28.4	26.9	1.8	0.94	2.240	0.239
Pelvic-fin length	28.1	13	22.1	31.0	26.6	2.0	0.92	-0.133	0.267
Last dorsal-fin spine length	15.6	13	14.7	17.4	15.7	0.7	0.97	1.532	0.137

HT = Holotype.

**Table 19:** *Australoheros* sp. “Jacuí”. Size (SL), proportional measurements (per cent of SL), and linear regression parameters.

	N	Min	Max	Mean	SD	r	a	b
SL (mm)	10	84.0	115.2	95.9	10.9			
Head length	10	33.0	36.3	34.6	1.3	0.95	6.828	0.274
Snout length	10	13.4	15.5	14.6	0.6	0.95	-1.674	0.164
Body depth	10	44.2	48.2	45.9	1.5	0.96	5.660	0.400
Orbital diameter	10	8.7	10.4	9.7	0.5	0.94	3.923	0.055
Head width	10	16.7	18.3	17.4	0.5	0.96	0.594	0.168
Interorbital width	10	10.4	12.0	11.6	0.5	0.98	-2.498	0.143
Preorbital depth	10	8.1	8.6	8.3	0.2	0.98	0.088	0.084
Upper jaw length	10	10.4	11.4	11.0	0.3	0.98	-0.139	0.111
Lower jaw length	10	13.1	14.6	13.7	0.5	0.95	-0.351	0.141
Caudal-peduncle depth	10	15.6	18.0	16.7	0.7	0.94	4.117	0.124
Caudal-peduncle length	10	8.8	12.1	10.2	0.9	0.75	0.298	0.099
Pectoral-fin length	10	25.9	27.9	27.0	0.7	0.99	4.877	0.219
Pelvic-fin length	10	29.0	33.1	31.1	1.3	0.94	-1.090	0.322
Last dorsal-fin spine length	10	14.8	18.1	16.5	1.0	0.89	0.298	0.162

Tables 10–19 show proportional measurements without adjustment for allometry. Limiting the size range to the shared size range 40.2–84.2 mm SL–with *A*. *ribeirae* excluded on account of too limited size range and *A*. sp. “Jacuí” out of range–showed considerable overlap, but also tentative differences between the species S3 File. *Australoheros mboapari* had a smaller orbit and tended to shorter head than the other species, but also *A*. *ricani* had a small orbit; *A*. *ricani* and *A*. *mbapoari* both had deeper preorbital than the other species, but overlapping each other; *A*. *mbapoari* and *A*. *minuano* tended to shorter lower jaw than the other species.

Box plots S4 File were influenced by actual size range and sample size for a species. *Australoheros oblongus* and *A*. *ipatinguensis* showed identity or complete overlap for all characters; comparison of Tables 11 and 15 shows that values were the same or very close.

Proportional measurements apparently had some resolving power only for the species that also were confidently identified by other characters.

**Fig 3:**
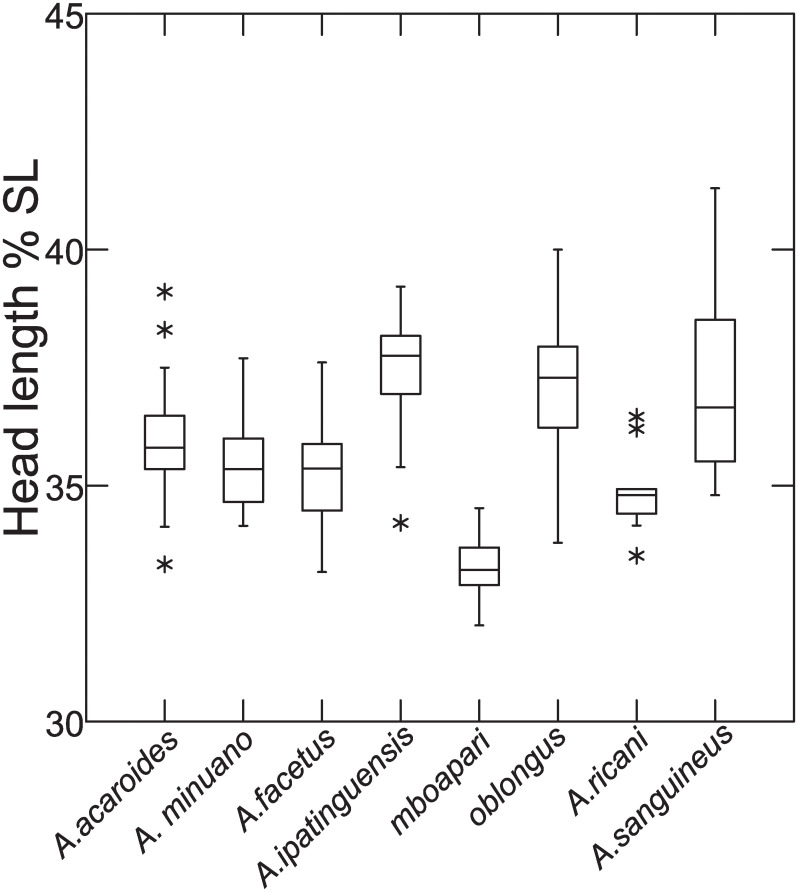
Box and whisker plot of last dorsal-fin spine length in per cent of SL in nominal species *Australoheros capixaba*, *A*. *A*. *mattosi*, and *A*. *tavaresi*.

The subset of specimens from the Sudeste assigned to nominal species based on locality and/or type status had some limitations as some samples were small (<6 specimens). The remaining samples showed variation in medians, but also wide standard length ranges. Adjusting the data set to samples with more than five specimens, and a shared SL range length from 33 to 86 mm, only three samples remained, representing the nominal species *A*. *capixaba*, *A*. *mattosi*, and *A*. *tavaresi*, which all overlapped on all measurements, except that the *A*. *capixaba* sample was marginal with a range and discrete median for the length of the last dorsal-fin spine at 21–22% (Fig 3). This may support the recognition of two species in the Sudeste region, as *A*. *capixaba* falls under *A*. *ipatinguensis* whereas *A*. *mattosi* and *A*. *tavaresi* fall under *A*. *oblongus* but it could also be an artefact due to the small size of the *A*. *capixaba* specimens.

**Fig 4:**
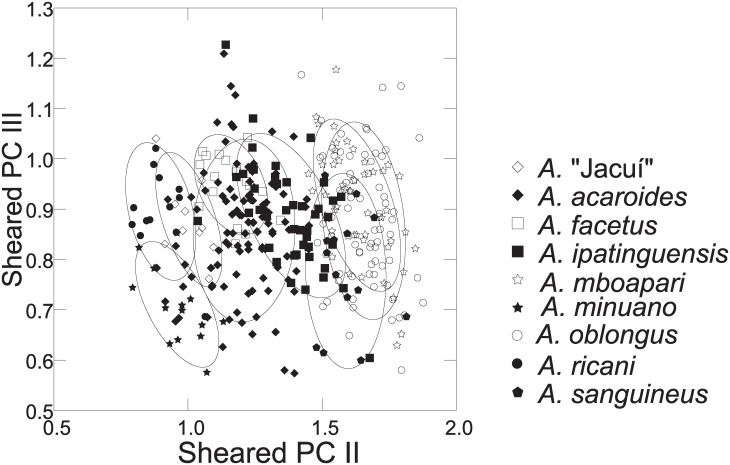
Plot of specimen scores of PC II on PC III from PCA of 15 distance measurements from pooled coastal species of *Australoheros*, and *A*. “Jacuí.” (Table S4.1 in S4 File).

The Principal Component Analysis of distance measurements in all coastal species (Fig 4) suggested limited differentiation, with three well represented species (*A*. *facetus*, *A*. *acaroides*, and *A*. *ipatinguensis*) covering a large part of the total variation. Components 2 and 3 explained up to 98.3% of the variance and had high loadings on preorbital depth, pectoral-fin length, caudal-peduncle length, pelvic-fin length, and length of last dorsal-fin spine.

Pelvic-fin length was expected to be sexually dimorphic, with longer pelvic fin in males. Only samples of *A*. *acaroides* (38 females, 53 males), *A*. *facetus* (18 females, 24 males), *A*. *ipatinguensis* (21 females, 17 males) and, *A*. *oblongus* (23 females, 49 males) have good representation of both sexes. There were no obvious sex differences in relative pelvic-fin length in *A*. *facetus*, *A*. *minuano*, *A*. *ricani*, *A*. *mboapari*, *A*. sp. “Jacuí”, or *A*. *oblongus* (ANOVA, p > 0.05). In *A*. *acaroides* there was significant effect of sex on the relative length of the pelvic fin (ANOVA, p = 0, F = 15.991). In each species, except *A*. *oblongus*, the largest females and males were similar in size. In *A*. *oblongus* the largest male was 113.0 mm SL, and the largest female 81.4 mm SL. The pelvic fin was often incomplete, with broken tip or individual rays, complicating interpretation of this measurement, but being paired, opposite side fins could substitute for each other if one was damaged. The caudal peduncle-length was a very short measurement which showed considerable variation over a span from 1.7 to 13.2 mm. For both SL and caudal-peduncle length measurements it was difficult to properly align the base of the caudal fin/posterior end of the caudal peduncle because the region was covered with dark pigmentation. For standard length, slightly bending the caudal fin exposed the crease marking the root of the caudal fin/posterior point of the standard length measurement. A minor error in the measurement of the standard length should not affect the measurement precision significantly. A small miss on the caudal peduncle length, however, would be significant on this short measurement. The length of the caudal peduncle may be better evaluated by counting the contained vertebrae on X-radiographs. Further PCAs were made without the pelvic-fin and caudal-peduncle lengths. The pectoral fin was also frequently damaged, but not to the extent of the pelvic fin. Most of the well-preserved specimens in this study dated to the last century, whereas recently collected specimens typically had damaged fins and twisted body. The last dorsal-fin spine was often broken near the apex and the location of the base required locating the distal radial under the basal scale cover of the dorsal fin without actually observing it, but the measurement had good reproducibility and may indicate aspects of evolution of the spines of the dorsal fin as predator deterrent or holder for various colour marks as in *Australoheros* and many other cichlids.

**Fig 5:**
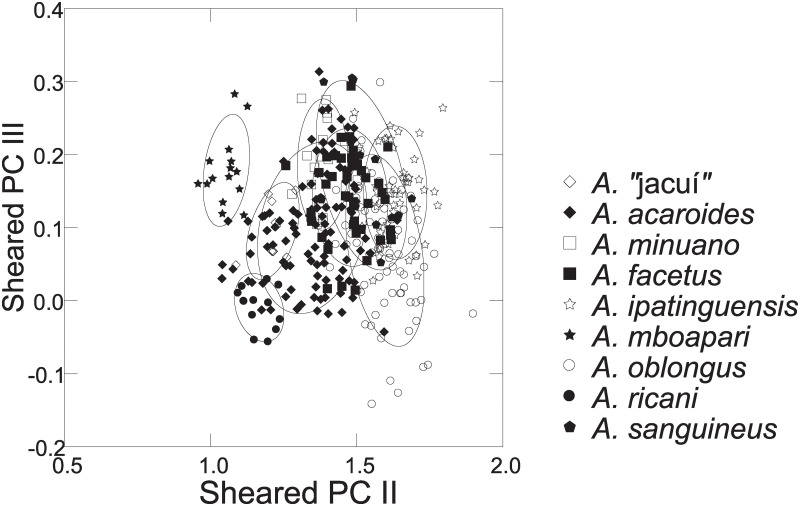
Plot of scores of PC II on PC III from PCA of 13 distance measurements from pooled coastal species of *Australoheros*, and *A*. “Jacuí.” (Table S4.2 in S4 File).

A smaller measurement set Table S4.2 in S4 File (without pelvic-fin length and caudal-peduncle length) highlighted snout length, preorbital depth, upper and lower jaw lengths, and length of last dorsal-fin spine (Fig 5). This suggests that variation may be strongest at the anterior end of the body. Notably, none of the width-related characters (head width and interorbital width) had much weight. That could mean that the analysis mainly measured changes in anteroposterior direction, characterising young specimens [79]. Species morphospaces were for the most part overlapping, but with distinct groupings of *A*. *mboapari*, and *A*. *ricani*. *Australoheros facetus*, *A*. *acaroides*, and *A*. *minuano* were mainly overlapping, like *A*. *sanguineus*, *A*. *ipatinguensis*, and *A*. *oblongus*.

To reduce effects of ontogenetic variability, a PCA was run on specimens with size constrained to 60–80 mm SL (Table S4.3 in S4 File). The result was similar to that from the larger sample. Pelvic-fin length and caudal-peduncle length dominated when included; when excluded, highest loadings were obtained from snout length, preorbital depth, lower jaw length, upper jaw length, and last dorsal-fin spine.

**Fig 6:**
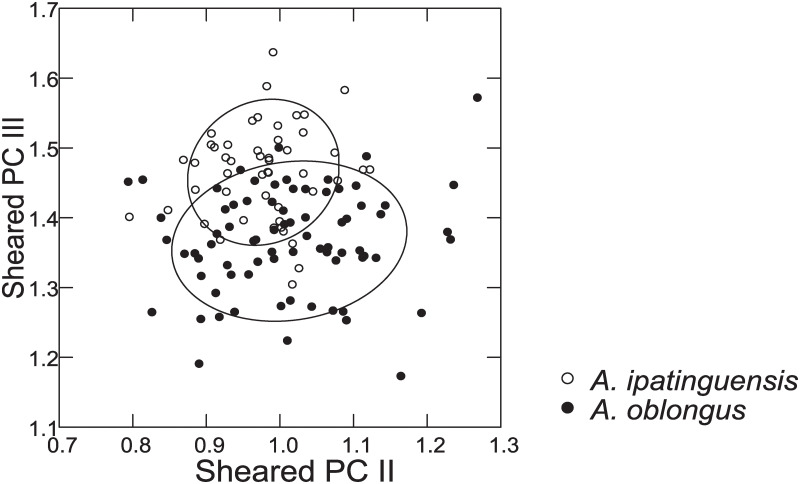
Plot of scores of PC II on PC III from PCA of 13 distance measurements from pooled samples of *Australoheros ipatinguensis* and *A*. *oblongus*.

Separating the major geographical groups S4 File gave only little improved resolution. PCA of only the Sudeste species showed *A*. *ipatinguensis* and *A*. *oblongus* broadly overlapping, with higher loadings on orbit, and upper and lower jaws (Fig 6, Table S4.4 in S4 File). The regression on standard length for these measurements was the same, however (ANCOVA, p >0.05).

**Fig 7:**
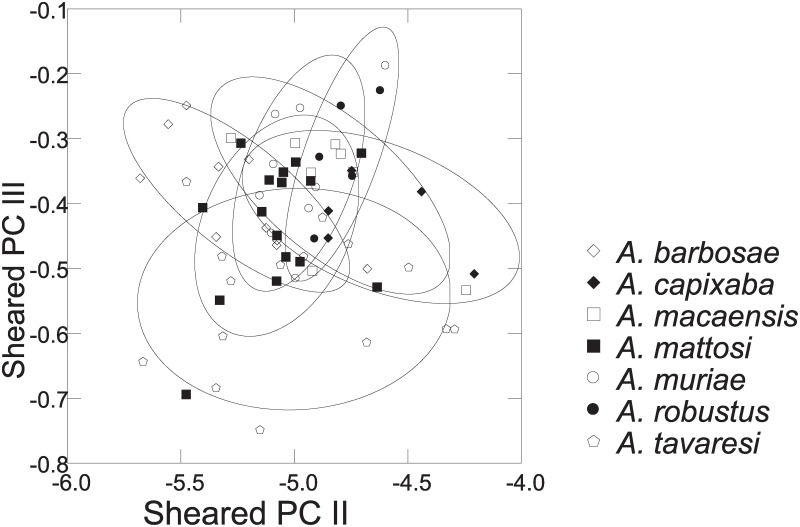
Plot of scores of P C III on PC II from PCA of 15 distance measurements of northern species of *Australoheros*, assigned to nominal species based on locality and/or species status, represented by minimum five specimens. (Table S4.6 in S4 File).

Breaking up the Sudeste species into samples representing type localities of nominal species described by Ottoni et al. (2008–2012), resulted in very small samples of some species, e.g. one specimen for *A*. *perdi*, and three for *A*. *ipatinguensis*. Only *A*. *tavaresi* and *A*. *mattosi* were well represented, by 17 specimens each. The PCA of the pooled subdivided Sudeste sample (Fig S4.5 in S4 File) and a further reduced sample (Fig 7, Table S4.6 in S4 File) of nominal species samples with minimum five specimens showed considerable overlap and no pattern indicating separation of nominal species.

**Fig 8:**
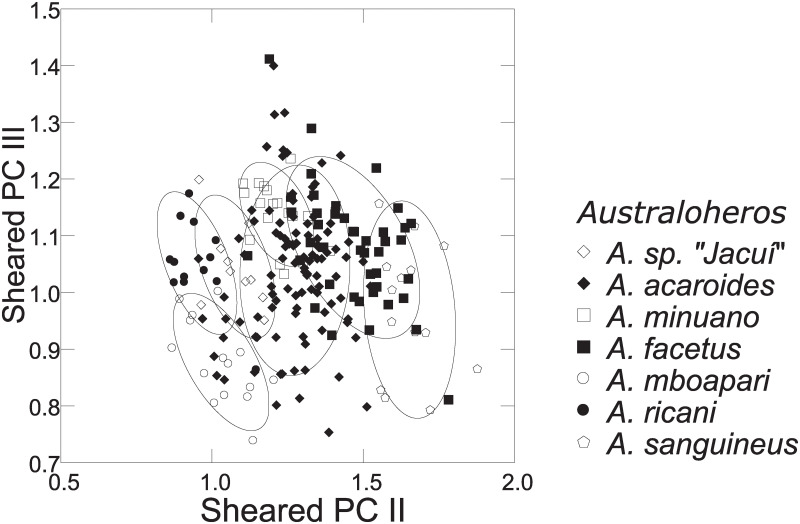
Plot of scores of PC III on PC II from PCA of 15 distance measurements from pooled southern coastal species of *Australoheros* (*A*. *acaroides*, *A*. *minuano*, *A*. *facetus*, *A*. *mbapoari*, *A*. *ricani*, *A*. *sanguineus*), and *A*. sp. “Jacuí.”.

We did not test specifically the species compositions presented by Ottoni et al. [47] for morphological validation, as those were based only on molecular data, but see comments on particular species, below. The separate PCA of southern species (Fig 8, Table S4.7, Fig S4.8 and Table S4.8 in S4 File) showed a distinct aggregate of specimens of *A*. *mboapari*, but did not separate *A*. *ricani*, *A*. *acaroides*, *A*. sp. “Jacuí”, *A*. *minuano*, or *A*. *facetus* from each other. Specimens of *A*. *acaroides* also overlapped with *A*. *ricani*. The PCA supported recognition of *A*. *mboapari* as a distinct species, but was otherwise uninformative for species delimitation.

Delimitation of *A*. *ricani* and *A*. sp. “Jacuí” remains problematic. Both OTUs are known only from the Rio Jacuí-mirim and shared derived characters such as the black soft dorsal fin in females and the long row of minute scales along the base of the dorsal fin. The only sample of *A*. sp. “Jacuí”, from Três Passos, was characterised by overall low meristic data, distinguishing not only from *A*. *ricani*, but also from all other species of *Australoheros*. By contrast, the morphometric analysis did not support species status.

**Fig 9:**
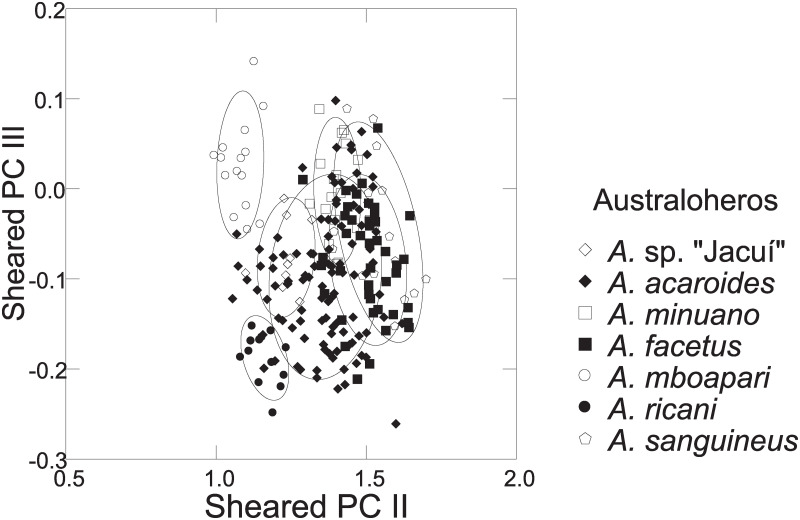
Plot of scores of PC II on PC III from PCA of 13 distance measurements from pooled specimens of *Australoheros* from the Rio Jacuí basin (*A*. *acaroides*, *A*. *facetus*, *A*. *mboapari*, *A*. *ricani*, and *A*. sp. “Jacuí.”.

The PCA of exclusively Rio Jacuí basin species (Fig 9, Table S4.9 in S4 File) attested to distinct species morphospaces and almost support for distinctness of the samples of *Australoheros mboapari* and *A*. sp. “Jacuí”.

Regression analysis distinguished. *Australoheros* sp. “Jacuí” from *A*. *ricani* by significantly different slopes in preorbital depth (p_H0_ = 0.009) and caudal-peduncle depth (p_H0_ = 0.019), and different intercepts (p_H0_<0.05) in body depth, orbital diameter, head width, interorbital width, pelvic-fin length, and length of last dorsal-fin spine. Given the small samples, the differences in conservation and SL ranges (84.0–115.2, average 95.9 mm SL, in the Três Passos sample; 56.9–109.9, average78.5 mm SL in the remainder) call for caution in interpretation of the differences. Apparently, morphometry does not allow separation of *A*. *acaroides* and *A*. *ricani*; or *A*. sp. “Jacuí” from *A*. *ricani*. On the other hand, mitochondrial DNA, squamation, and colour pattern clearly separated *A*. *ricani* from *A*. *acaroides*; and *A*. sp. “Jacuí” was distinct from them both in meristic data.

The samples available of *Australoheros ribeirae* and *A*. *sanguineus* were too small to be informative; *A*. *ribeirae* was represented by only four specimens, not permitting statistical analysis. The scattered representation on PCA plots accorded with the heterogeneous origin of the material of *A*. *sanguineus*, from the Paraná basin as well as different coastal rivers. There was nevertheless no indication that the sample would have included more than one species.

In conclusion, the Principal Component Analysis of traditional measurements provided support for recognition of *A*. *mboapari* as an entity different from all other species of *Australoheros* examined here. This was not supported by traditional ratio data, however, in which all species overlapped (Tables 10–19). Large samples showed wide ranges representing aberrant specimens or samples of reduced breeder sizes. *Australoheros sanguineus* was distinguished with somewhat deeper body than the rest, and *A*. *ricani* and *A*. *mboapari* had more elongate body, longer snout and deeper preorbital than remaining species. Regression analysis of the distance measurements on SL failed the hypothesis of a common slope in all of them (p = 0–0.0212). Variation, however, may have been influenced by different size ranges in species samples, and increased variability among large specimens, irrespective of species. Body depth, indicated as distinguishing *A*. *mboapari* in the PCA, had the same slope as in *A*. *minuano* and *A*. *ricani* when these three species were analysed alone.

Altogether the morphometric analysis suggested that all species are relatively variable, and did not show any species diagnostic character state. Northern and southern species formed broadly overlapping clusters, and within-group analysis failed to recover distinct species. Nevertheless, *A*. *ricani* and *A*. *mboapari*, each formed distinct clusters, although not distinct from the very variable *A*. *acaroides*. The analysis may be impacted by wide size span of the total sample, different preservation states, and the inclusion of precocious specimens. Precocious specimens were observed in *A*. *acaroides*, but may be more common.

### Qualitative morphology

Two morphological characters separated nominal species now contained in *A*. *oblongus* from those in *A*. *ipatinguensis*. *Australoheros oblongus* was slightly more elongate, and usually had an indentation in the frontal contour, reflecting slightly inflated frontal tissue immediately anterior to the dorsal-fin base. It was also characterised by a large caudal spot spanning the middle of the caudal-fin base. By contrast, *A*. *ipatinguensis* was slightly more deep-bodied and smaller specimens had a characteristic steep frontal contour without conspicuous indentation; the caudal spot was concentrated to the upper lobe of the caudal fin, although dark pigment was present on the adjacent lower lobe in in some specimens. The presence of an indentation in the frontal contour of *A*. *ipatinguensis* was related to the collapse of the tissue in the recess for the ascending processes of the premaxilla, but might also have reflected a slight difference in orientation of the recess, which was not further investigated. Very large specimens of *A*. *ipatinguensis* showed a frontal indentation, but could be recognised by the characteristic caudal spot. The frontal indentation recalled Chakrabarty’s [80] character 68, ‘Dorsal head profile: interorbital concavity’, which reflects a protruding premaxillary pedicel (cf. Barel et al. [81]). Chakrabarty coded the state in ‘*C’*. *facetum* as absent or weak, with the alternative present and deep. The species identity of Chakrabarty’s *‘C’*. *facetum* was not verified.

In 1898 Jordan and Evermann [82] separated the genera *Cichlasoma* and *Heros* on the presence of a frenum in the former, absent in the latter. This distinction was criticised and considered as artificial by Pellegrin in 1904 [36], but nevertheless adopted by him, placing *Chromys facetus in Cichlasoma*, and *Heros autochthon* and *Chromys oblonga* in *Heros*. Judging from Pellegrin’s fig. 37, he distinguished between a state where the lower lip fold is continuous, versus a state where the lower lip fold is discontinuous (absent symphysially). In 1905, Regan [37] distinguished *Cichlasoma facetum* with ‘fold of the lower lip not continuous’ and *Chromys oblonga* and *Heros autochthon* with ‘fold of the lower lip continuous’. According to our observations all species of *Australoheros* possess a continuous lower lip fold, but also a frenum, which is a short median membrane attaching to the very root of the lip fold and not exposed externally. This frenum, however is not the same as the lip fold in cichlid species and which may be interrupted by a smooth median surface.

The lower pharyngeal tooth plate in cichlids shows variation in shape and tooth shape corresponding to diet [79, 81] and was figured for species of *Australoheros* by Casciotta et al. [83, 84] and Říčan and Kullander [4, 85]. As far as studied and illustrated, variation in bone and tooth shape is limited, and there are no studies of ontogenetic series or even larger samples, enabling a phylogenetic analysis. Lower pharyngeal tooth plates are reported here under the respective species.

Říčan and Kullander [4] and Říčan et al. [13] distinguished species of *Australoheros* from the Uruguay and Paraná drainages by combinations of scale and colour characters. Southern species except *A*. *facetus*, but including *A*. *sanguineus*, shared a distinctive colour pattern, expressed in a lateral band consisting of dark scale margins or blotches on scale rows 0 and E1, ending with Bar 4. As the dark markings interdigitate, the lateral band has a remote likeness to a zipper, and consequently it is referred to here as a zipper band. In *A*. *facetus*, juveniles had a distinct broad lateral band, lost in large adults, but the zipper marking was absent at all sizes. The zipper marking was present also in the species from the Uruguay and lower Paraná basins, and the same or similar marking was recorded in *A*. *oblongus*. In the breeding colour pattern the scale blotches merge to form a single continuous lateral band indistinguishable from the lateral band in adult *A*. *facetus* and *A*. *ipatinguensis* and *A*. *oblongus*. The presence of four rather than three abdominal bars occurred in low frequency in *A*. *facetus*, and *A*. *acaroides*, and *A*. *minuano* among southern the species, but was very rare in *A*. *oblongus* and *A*. *ipatinguensis*. The fourth bar represented a split Bar 5 (Říčan et al. [58]).

Říčan and Kullander (figs 8A, 9A–C) [4] and Říčan et al. [13] described a scale pattern unique among cichlids, consisting of a row of minute scales paralleling the dorsal-fin base cephalad to the anterior dorsal-fin insertion, close to which the row may be doubled. This scale character state was reported from *Australoheros forquilha*, *A*. *tembe*, *A*. *ykeregua*, and *A*. sp. “Jacuí”. We found it also in *A*. *mbapoari* and *A*. *ricani*. In remaining species of *Australoheros* the minute scales were present, but reached only to the middle of the dorsal fin-base or shorter as illustrated by Říčan and Kullander, figs. 8b D–I [4]. Because *A*. *tembe* did not group with *A*. *ykeregua+A forquilha* in the *mt-cyb* tree or the morphological parsimony tree presented by Říčan et al. [13], the condition cannot be a synapomorphy of *A*. *tembe A*. *forquilha*, *A*. *ykeregua* and *A*. sp. “Jacuí”–the forquilha group of Říčan and Kullander [4]–but may relate to the inferred or observed rheophily of those species–*A*. sp. *“*Jacuí”, *A*. *ricani*, and *A*. *mboapari*.

Ottoni et al. [5] and Ottoni and Costa [6] introduced five osteological characters in northern species and *A*. *facetus*, namely the length of the anterior arm of epibranchial 1, with states long (all Sudeste species) or short (*A*. *facetus* only); process on second vertebra [neurapophysis of preural 2], with states truncate (*A*. *ribeirae* only) or pointed; ectopterygoid width, with states slender (*A*. *ribeirae* only in Ottoni et al. [5]; also *A*. *ipatinguensis* in Ottoni and Costa [6] or wide; and epibranchial 2 processes with the two states: long [epiphyseal]processes [one at each end] (*A*. *autrani*, *A*. *macacuensis*, *A*. *macaensis*, *A*. *muriae*, and *A*. *saquarema*) or two short processes [one at each end] (*A*. *barbosae*, *A*. *facetus*, *A*. *ipatinguensis*, *A*. *paraibae*, and *A*. *robustus*). In later papers by Ottoni and collaborators the reference to the preural 2 neurapophysis was dropped, and osteological data were absent from the descriptions of *A*. *taura* and *A*, *sanguineus*. The only species with ‘short anterior arm’ was *A*. *facetus* and *A*.*ipatinguensis* (sensu Ottoni and Costa 2008 [6]); the only species with narrow ectopterygoid were *A*. *ribeirae* and *A*. *ipatinguensis* (sensu Ottoni and Costa 2008 [6]).

It is not obvious what ‘anterior arm long’ refers to. The relative length of the anterior arm and uncinate process of epibranchial 1 has phylogenetic significance. Casciotta and Arratia [86] (character 28) found that an uncinate process and anterior arm of the first epibranchial of equal length or the uncinate process slightly the longer to be the state in the majority of American cichlids including their material identified as *A*. *facetus*. The alternative state of a much longer uncinate process in comparison with the anterior arm was found in some chaetobranchines, crenicichlines, and geophagines. In our cleared and stained specimens of *A*. *minuano*, *A*. *acaroides*, *A*. *angiru*, *A*. *facetus*, *A*. *scitulus*, *A*, *ykeregua*, and *A*. *sp*. “Jacuí” the anterior arm and uncinate process are equal in length, as also shown by Ottoni and Costa (fig. 4) [6]. This character, and the length of the epiphyseal processes of the second epibranchial, where it also is not clear if how long–a relative measure–is defined. In our cleared and stained specimen of *A*. *facetus* the separation of the anterior arm and uncinate process was relatively shorter than in specimens of *A*. *acaroides*, *A*. *angiru*, *A*.*minuano*, *A*. *scitulus*, *A*. *ykeregua*, and *A*. “Jacuí” in which the anterior arm and uncinate process branch at the middle or only slightly more distad on the curved bone observed in situ in dorsal view, a condition illustrated also by Casciotta (figs 30 and 34) [86]. We take this condition to be ‘short anterior arm’. The body of epibranchial 2 showed variation in width, possibly explaining the variation in the relative length of the epiphysial ends. Among our cleared and stained specimens, slender epiphysial projections were observed in *A*. *angiru*, *A*. *acaroides*, and *A*. *ykeregua*, interpreted as ‘long projections’.

The ectopterygoid was of the same shape in our cleared and stained specimens of *Australoheros*. *minuano*, *A*. *acaroides*, *A*. *facetus*, *A*. *scitulus*, *A*. *ykeregua*, and *A*. sp. “Jacuí”, corresponding to the wide state of Ottoni and Costa (fig.3) [6]), as illustrated by Casciotta (fig. 24) [86]. The figure of a narrow ectopterygoid in Ottoni et al. (fig. 5) [5] and Ottoni and Costa (fig. 3) [6] suggests a teratological condition.

In our cleared and stained specimens the preural 2 neurapophysis varied slightly in shape. In lateral aspect it typically had a tulip-shaped or beaker-shaped outline with a broad proximal ‘stalk’ and a wider distal portion with an emarginate apex opposed to epural 2. In one of our cleared and stained specimens of *Australoheros acaroides* it was very short, with a narrow, pointed apex; in our cleared and stained specimen of *A*. *facetus* it was relatively narrow, with a pointed apex similar to one of our specimens of *A*. *ykeregua* and the *A*. *facetus* specimen illustrated by Casciotta (fig. 47) [87]). In the specimen of *A*. *scitulus* it had a broad arrow-shaped tip and truncate apex. In one of the specimens of *A*. *angiru* it was broad, short and truncate apically.

Given that the only osteological variation recorded seems to be in the epiphyses of the endochondral pharyngobranchials 1 and 2, that those are subject to continuous growth, potentially varying with specimen size, and that specimen size was not documented, the osteological characters described by Ottoni et al. [5] and Ottoni and Costa [6] do not demonstrate strong evidence of species differentiation.

### Molecular species delimitation: *mt-coI*

**Fig 10:**
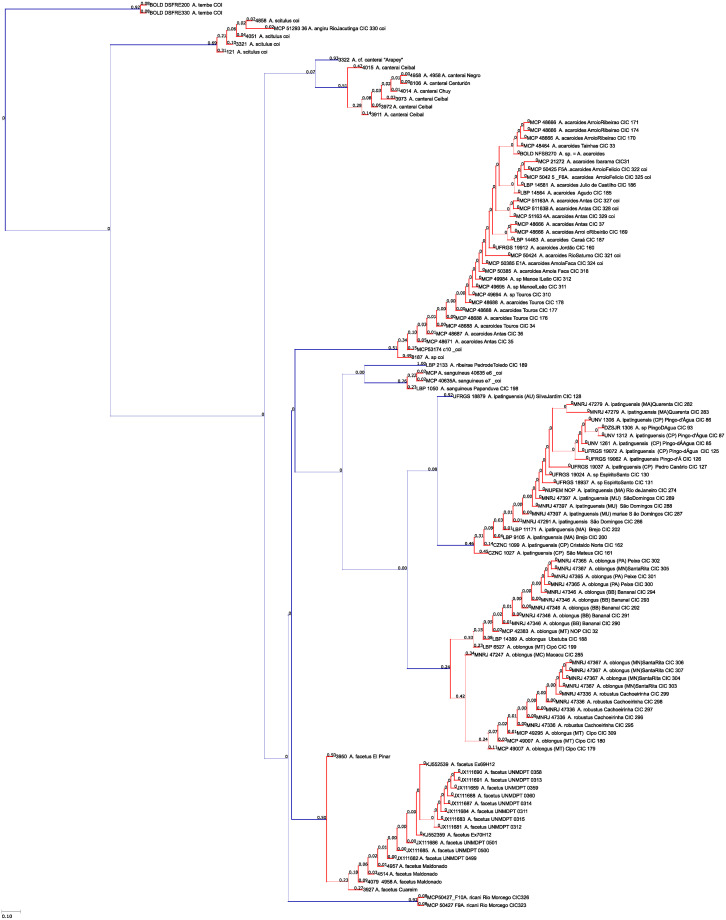
Delimitation of species of *Australoheros* based on bPTP analysis of *mt-coI* sequences. Blue lines lead to suggested species; red lines combine within-species branches. Node numbers are percentage support values generated by bPTP. OTU names are as those considered as valid species; nominal species identified by locality are indicated by codes inserted within parentheses: AU = *A*. *autrani*; BB = *A*. *barbosae*, CP = *A*. *capixaba*; MA = *A*. *macaensis*, MC = *A*. *macacuensis*; MU = *A*. *muriae*; PB = *A*. *paraibae*.

The bPTP analysis of *mt-coI* suggested species units with bPTP-specific support values ranging from 0.2 to 1 (Fig 10). Clades with few species had strongest support (*Australoheros tembe*, *A*. *sanguineus*, *A*. *ribeirae*, putative *A*. *autrani*, and *A*. “Arapey”.) *Australoheros angiru* and *A*. *scitulus* were paired in one unit. Remaining units recognised *A*. *acaroides*, *A*. *facetus*, *A*. *ricani*, *A*. *ribeirae*, *A*. *sanguineus*, *A*. *oblongus A*. *ipatinguensis*, and *A*. *minuano*.

Uncorrected *p-*distances (Table 20, S1 Table) separated the coastal species from the Uruguay and Paraná basin species (*A*. *angiru*, *A*. *scitulus*, and *A*. *tembe*) by more than 4% minimum *p*-distance. The Sudeste and the southern coastal species separated by 3–4% minimum *p*-distance (3.07 in *A*. *facetus* to 4.14% in *A*. *ricani*) but only 2.76% in *A*. *sanguineus*, and the two groups could be analysed separately: (non-zero *p*-distance only).

**Table 20:** Uncorrected *p*-distances in *mt-coI* between MOTUs of *Australoheros*.

*A*. *acaroides*	*acaroides*	*oblongus*	*ipatinguensis*	*ricani*	*"*Arape*y"*	*canterai*	*facetus*	*sanguineus*	*ribeirae*	*tembe*	*scitulus*	*angiru*
*A*. *oblongus*	3.83		1.53	4.14	3.99	3.99	3.07	2.76	3.07	7.98	5.67	8.9
*A*. *ipatinguensis*	3.83	1.69		4.14	3.83	4.29	3.07	2.76	3.07	7.82	5.37	8.9
*A*. *ricani*	3.83	4.14	4.14		3.53	3.83	3.07	3.53	3.83	7.21	6.6	9.66
*A*. *"*Arapey*"*	3.22	3.99	3.83	3.53		0.92	1.99	3.07	3.37	7.06	5.37	8.44
*A*. *canterai*	3.53	3.99	4.29	3.83	0.92		1.99	3.37	3.53	7.06	5.06	8.13
*A*. *facetus*	2.61	3.07	2.76	3.07	1.99	1.99		2.61	2.3	6.29	5.21	8.59
*A*. *sanguineus*	3.37	2.76	2.76	3.53	3.07	3.37	3.07		1.99	7.21	5.52	8.9
*A*. *ribeirae*	3.83	3.07	3.07	3.83	3.37	3.53	2.3	1.99		7.82	6.44	9.82
*A*. *tembe*	6.6	7.98	7.82	7.21	7.06	7.06	6.29	7.21	7.82		5.52	3.68
*A*. *scitulus*	5.67	5.67	5.37	6.6	5.37	5.06	5.21	5.52	6.44	5.52		3.68
*A*. *angiru*	9.2	8.9	8.9	9.66	8.44	8.13	8.59	8.9	9.82	8.74	3.68	

*Cichlasoma portalegrense* serves as outgroup measure. Species names are those considered valid in this study. Nominal northern species based on locality are identified by codes in parenthesis: AU = *A*. *autrani*; BB = *A*. *barbosae*, CP = *A*. *capixaba*; IP = *A*. *ipatinguensis*; MA = *A*. *macaensis*; MC = *A*. *macacuensis;* MN = *A*. *montanus*; MT = *A*. *mattosi*; MU = *A*. *muriae*; PB = *A*. *paraibae*; RB *= A*. *robustus*. Each entry in the first column has an abbreviated locality indicated; Specimen identifiers refer to NRM tissue numbers (numeric only), MCP tissue numbers (CIC numbers); and Genbank or BOLD identifiers. Refer to Table 1 and S1 File for full metadata.

Minimum *p*-distance between *A*. *oblongus* and *A*. *ipatinguensis* was 1.69, much less than the separation from *A*. *ribeirae* (3.07). The internal *p*-distance in *A*. *oblongus* was 0.31–0.77%; in *A*. *ipatinguensis* 0.77–1.23%. The large internal span in *A*. *ipatinguensis* marked the influence of the Silva Jardim specimen, without which the internal *p*-distance would be 0.15–0.92%, The rest of the samples showed minimum *p*-distances compatible with a species divergence threshold of 2–3%. The wide internal divergence demonstrates the wide geographical distribution of *A*. *ipatinguensis* by specimens representing terminals in the distribution from the Rio Paraíba do Sul to the middle Rio Doce.

Among the southern species, two sequences stood out: the UFRGS 19207 sequence–from a tributary to the Lagoa dos Barros, very seaward in the Laguna dos Patos basin–which was most similar to sequences of *A*. *acaroides* but still very different from the rest; and MCP 50424 from the Rio Soturno, which was very different from all other *A*. *acaroides* sequences. Distances reported below excluded those two specimens, but they were included in S1 Table. Internal non-zero variation was lower than external divergence, which exceeded 2% for all species, in *A*. *ricani* by more than 4%.

*Australoheros minuano* had an internal *p-*distance of 0.15–0.46%. The only specimen of *A*. “Arapey” was most similar to *A*. *minuano*, but 0.92–1.07% different. *Australoheros minuano* and *A*. *facetus* differed by only 2.3–2.6%, indicating close phylogenetic relationship.

Only one sequence of *A*. *ribeirae* and two of *A*. *sanguineus* were available. The *A*. *sanguineus* sequences were identical and differed from that of *A*. *ribeirae* by only 1.99%, calling for further analysis of the validity of *A*. *sanguineus*.

**Fig 11:**
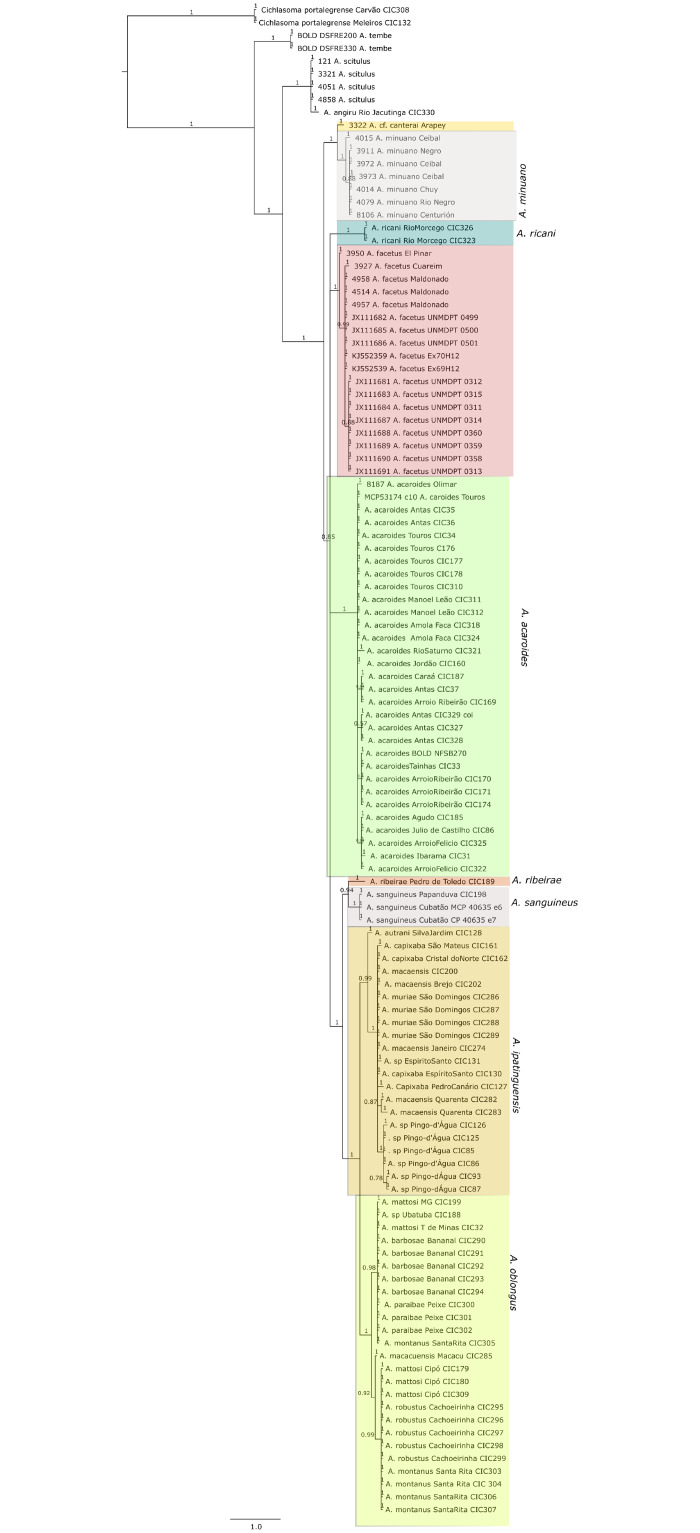
Phylogenetic tree of species of *Australoheros* based on Bayesian inference of sequences of *mt-coI*. Coloured rectangles and names to the right cover valid species; names of nominal species identified by locality contained within rectangles.

The Bayesian phylogenetic tree based on *mt-coI* (Fig 11) has distinct clades (bpp = 0.99–1) for *A*. *tembe*, *A*. *scitulus*, *A*. *angiru*, *A*. *ricani*, *A*. *facetus*, *A*. *acaroides*, *A*. *ribeirae*, *A*. *sanguineus*, *A*. oblongus, and *A*. *ipatinguensis*. In the *A*. *minuano*+*A*. “Arapey” clade, the Arapey specimen was distinct, reducing bpp to 0.88.

### Molecular species delimitation: *mt-cyb*

**Fig 12:**
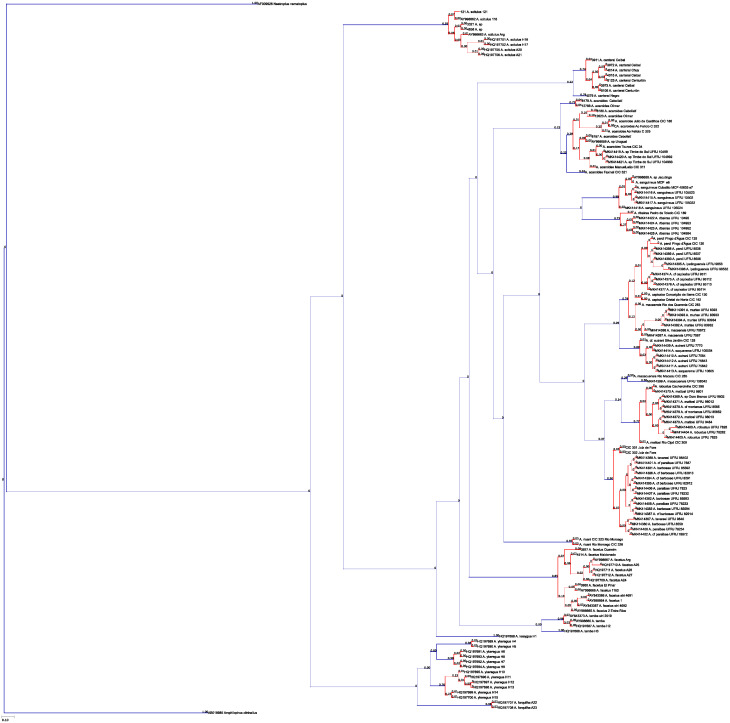
Delimitation of species of *Australoheros* based on bPTP analysis of *mt-cyb* sequences. Blue lines lead to suggested species; red lines combine within-species branches. Node numbers are percentage support values generated by bPTP. OTU names are as those considered as valid species; nominal species identified by locality are indicated by codes inserted within parentheses: AU = *A*. *autrani*; BB = *A*. *barbosae*, CP = *A*. *capixaba*; MA = *A*. *macaensis*, MC = *A*. *macacuensis*; MU = *A*. *muriae*; PB = *A*. *paraibae*.

The bPTP analysis of *mt-cyb* (Fig 12) suggested 22 ingroup species of *Australoheros* with support values from 0.28 to 1. The analysis may have overdone splitting as it found two units of *A*. *ykeregua* from the same provider, and two units of *A*. *acaroides*, *A*. *tembe*, and *A*. *minuano*. Sudeste samples were grouped in six species, among which the two Macacu samples ended up as separate species.

**Fig 13:**
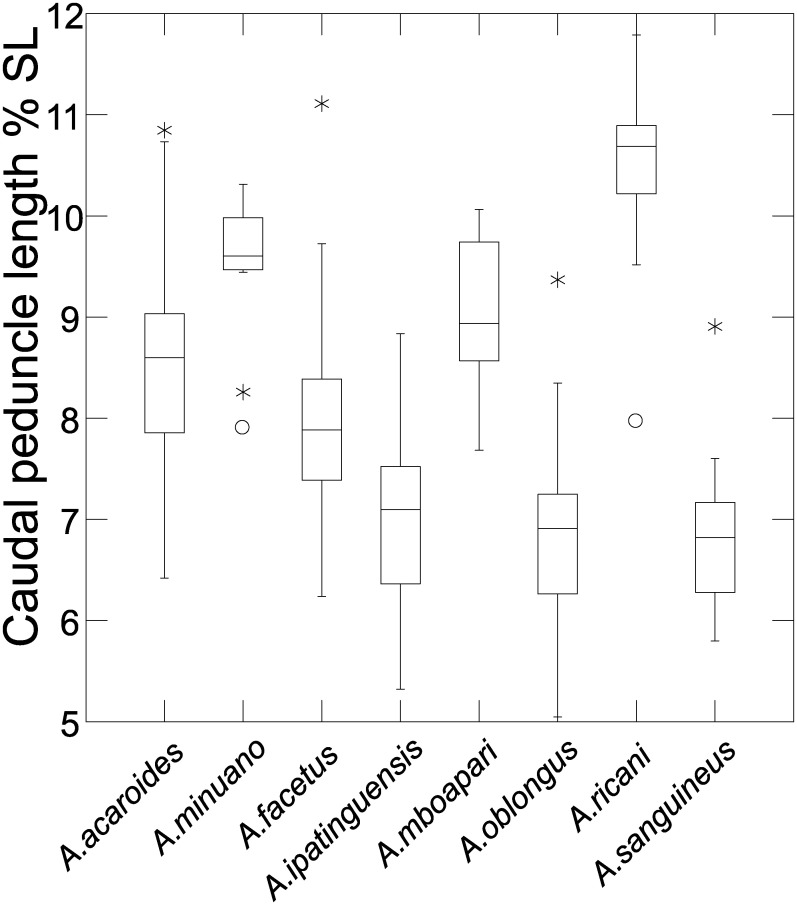
Phylogenetic tree of species of *Australoheros* based on Bayesian inference of *mt-cyb* sequences. Coloured rectangles and names to the right cover valid species; names of nominal species identified by locality contained within rectangles.

The Bayesian phylogenetic tree of *mt*-*cyb* (Fig 13) showed very little divergence, recognising clades *A*. *ykeregua*, *A*. *forquilha*, *A*. *scitulus*, *A*. *tembe*, *A*. *kaaygua*, *A*. *facetus*, *A*. *minuano*, *A*. *acaroides*, *A*. *ricani*, *A*. *sanguineus*, *A*. *ribeirae*, *A*. *oblongus* and *A*. *ipatinguensis*. Posterior probabilities provided credibility to the clades *A*. *forquilha*, *A*. *ykeregua*, *A*. *scitulus*, *A*. *kaaygua*, *A*. *minuano*, *A*. *acaroides*, *A*. *ricani*, *A*. *ribeirae*, *A*. *sanguineus*, *A*. *ipatinguensis* and *A*. *oblongus* (bpp 0.94–1); only *A*. *facetus* (bpp 0.84) appeared contestable.

Mitochondrial *mt-cyb* sequences published by Říčan and Kullander [3] and Říčan et al. [13] were included in the Bayesian phylogenetic analysis (Fig 13). that analysis, fig. 2, and that of Říčan and Kullander (fig. 1) [3], AY998659 (*A*. sp. uruguai from Salto, here called *A*. *minuano*), and AY998658 (*A*. sp. jacutinga from the Rio Iguaçu basin; here called *A*. *angiru*), were sister taxa. In our tree, AY998659 (*A*. sp. uruguai) ended up inside *A*. *acaroides*, and AY998658 (*A*. sp. jacutinga) clustered with *A*. *sanguineus*, calling into question the locality information for AY998658 and AY998659.

### Phylogenetic interrelationships

The *mt-cyb and mt-co1* trees (Figs 11 and 13) showed the same branching pattern as far as taxa overlap. The Sudeste specimens minus *A*. *ribeirae* formed two clades, each with only minimal branch lengths.

*Australoheros ribeirae* and *A*. *sanguineus* were sister species. The *A*. *ribeirae+A*. *sanguineus* clade made up one branch in a trichotomy with the southern and Sudeste clades. The bpp value for this node was relatively low in the *mt-cyb* tree (Fig 13: 0.63); but higher in the *mt-coI* tree (Fig 11: 0.94). Among southern species, the ingroup had *A*. *ricani* as sister clade to the rest. *Australoheros facetus* and *A*. *minuano* were sister species, and this clade was sister to a trichotomy of *A*. *acaroides*, the *A*. *ribeirae*+*sanguineus* clade, and the Sudeste clade with two species.

The concatenated Bayesian tree of *mt-cyb* and *mt-coI* sequences S5 File suffered from the unequal, samples and also short length of many of the *mt-cyb* samples. In general it followed the pattern of the single-gene trees but with *A*. *tembe* non-monophyletic (different clades for *mt-*co*I* and *mt-cyb*).

There was a very clear separation of southern and northern species, and restricted overlap in distribution pointing to mainly allopatric speciation. Nevertheless, several species were recorded from more than one river drainage, e.g. *Australoheros oblongus* in the Doce, Macacu, Paraná, Paraíba do Sul and São Francisco basins; *A*. *facetus* in the Uruguay and Paraná basins, and *A*. *acaroides* in the Uruguay and Jacuí basins; *A*. *ipatinguensis* present in several coastal rivers and lagunar affluents, and *A*. *sanguineus* in the Iguaçu and Cubatão basins. Sympatry of *A*. *oblongus* and *A*. *ipatinguensis* in the Paraíba do Sul drainage, suggested relatively recent dispersal. *Australoheros ipatinguensis* was widespread in near-coast areas, extending far north, beyond the Rio Doce drainage, whereas *A*. *oblongus* was mainly montane. In the south, the Salto de Jacuí was probably a barrier separating *A*. *ricani* and *A*. *acaroides*. *Australoheros acaroides* was parapatric with *A*. *mboapari* in the Rio Taquarí.

### Species accounts

Here we summarise information on nominal species and discriminating characters of valid species. The species accounts are ordered chronologically by year of original description. Species from more interior localities in the Paraná and Uruguay River basins are presented in S2 File.

#### *Australoheros facetus* (*Jenyns*, 1842)

*Chromis facetus* Jenyns, 1842 [29]: 104 (holotype UMZC FF6640, not seen; type locality Maldonado, in a lake of fresh water).

*Heros Jenynsii* Steindachner, 1869 [33]: 149 (syntypes NMW 17324–27 (4), 58722 (1); type locality Montevideo; full description in Steindachner, 1870 [88]: 202, pl. II).

*Definition*. Based on the position in the *mt*-*cyb* and *mt-coI* trees and minimum uncorrected *p*-distance in *mt-coI* exceeding 2% from all other species of the genus, *Australoheros facetus* is a distinct evolutionary lineage. Specimens of *A*. *facetus* can be distinguished from congenerics by colour pattern and scale cover: uniform dark lateral band present in young specimens, lost in adults; Y mark absent, minute scales below spinous dorsal-fin base absent.

**Fig 14:**
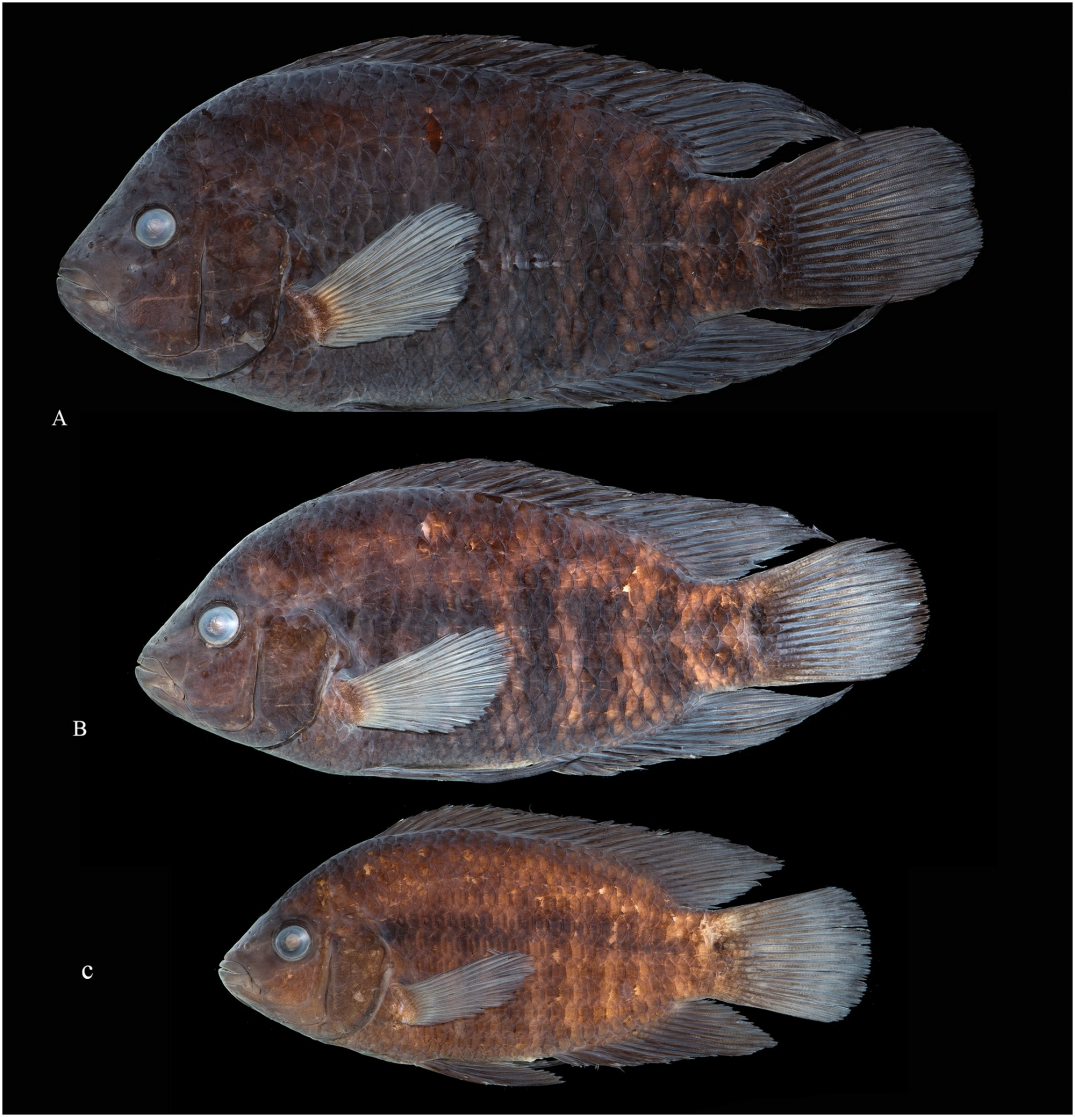
Australoheros facetus. **A.** adult male, 145.4 mm SL; NRM 55720. **B** adult female, 108.8 mm SL; NRM 55720. **C.** young female, 55.4 mm SL; NRM 55720. All from Uruguay: Maldonado: Laguna del Diario.

*Description of sample*. Based on specimens from the type locality (NRM, 54218, 55720, 56035). Proportional measurements are given in Table 10, meristic data in Tables 3–9. Refer to Fig 14 for general aspect of adults.

Adults (Fig 14), about 50 mm SL, and larger, moderately elongate, dorsum slightly elevated matching ventral profile: laterally compressed, more so caudad. Frontal contour ascending at about 40°, straight, slightly curved close to dorsal-fin base, which straight or slightly curved, gently descending at base of soft dorsal fin. Caudal peduncle margins straight, about parallel, or slightly sloping. Very slight indentation in frontal contour anterior to orbit. Nuchal protuberance absent. Prepelvic contour straight or nearly so, descending at about 20°; abdominal contour straight horizontal; anal-fin base slightly ascending. Orbit lateral, removed from frontal contour, in anterior half head, slightly dorsal to midaxis level of body. Gill cover with distinct indentation at dorsal junction of opercle and subopercle.

**Fig 15:**
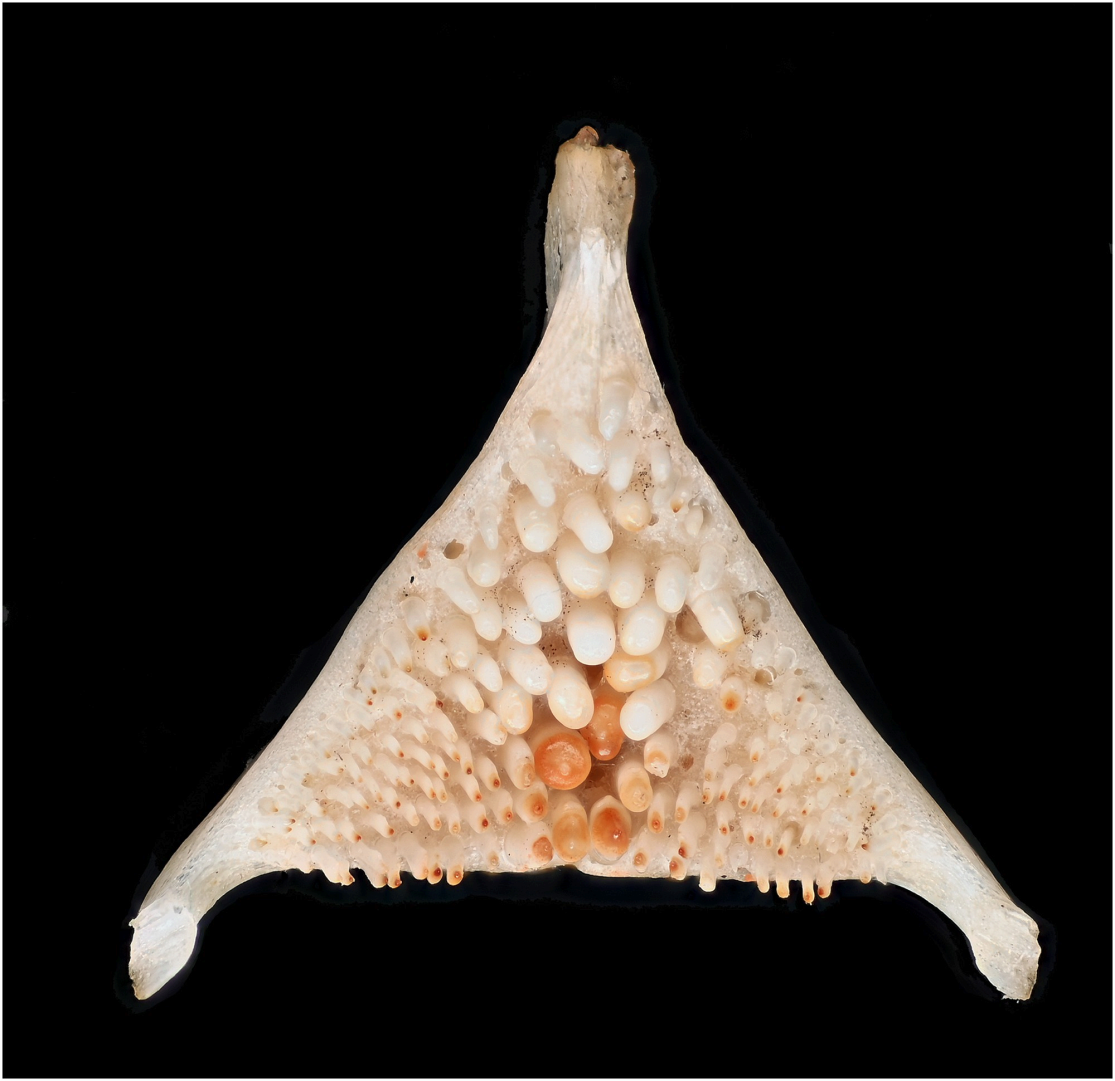
*Australoheros facetus*, NRM 55720, 100.5 mm SL; lower pharyngeal tooth plate in occlusal.

Mouth terminal, below level of orbit; tip of maxilla exposed, not reaching to vertical from anterior margin of orbit. Jaws isognathous; tooth band of lower jaw closing opposite or posterior to upper tooth band. Lips moderately thick, both lip folds interrupted symphysially. Jaw teeth in 2–3 rows, in juveniles only one row. Six anterior teeth in outer row in both jaws slightly larger than rest, pointed, slightly recurved; small second cusp present lingually, second row teeth much smaller than outer teeth, caniniform, at least some teeth with small second cusp lingually; teeth in third row, if present, minute, caniniform. Teeth in outer hemiseries 8–14 in each jaw. Abraded teeth absent. Lower pharyngeal tooth plate (Fig 15) relatively slender, length 70.2% of width; dentigerous area length 39% of dentigerous area width. Anterior teeth subconical, slightly retrorse; 9 wide teeth in anteroposterior row along median suture, apex tuberculate on middle teeth, posterior teeth slightly compressed, bicuspid with antrorse distal cusp; laterad and posterolaterad on bone plate, teeth gradually smaller and compressed; about 19 teeth in posterior row. Microbranchiospines present externally on ceratobranchials 2–4.

Predorsal scales irregular, exposed distally, about 11–14 along midline. Scale rows on cheek 3–4; cheek naked next to rictus. Scales between dorsal fin and upper lateral line 2 large and one small anteriorly; one large and a pair of small scales posteriorly. Dorsal fin anterior insertion at vertical from insertion of pectoral-fin base; first dorsal-fin spine about ½–3/5 length of last spine, length increase gradually from first to last spine, subequal from fifth or sixth spine. Soft dorsal fin with broad or slightly elongated pointed tip, rays 5 and 6 longest, extending to about vertical through middle of caudal fin or slightly further. Anal-fin spines increasing slightly in length from first to last; soft dorsal fin with broad tip, soft rays 4 and 5 forming broad or elongated tip, reaching at most to slightly beyond vertical through middle of caudal fin. Caudal fin with rounded or subtruncate posterior margin.

**Fig 16:**
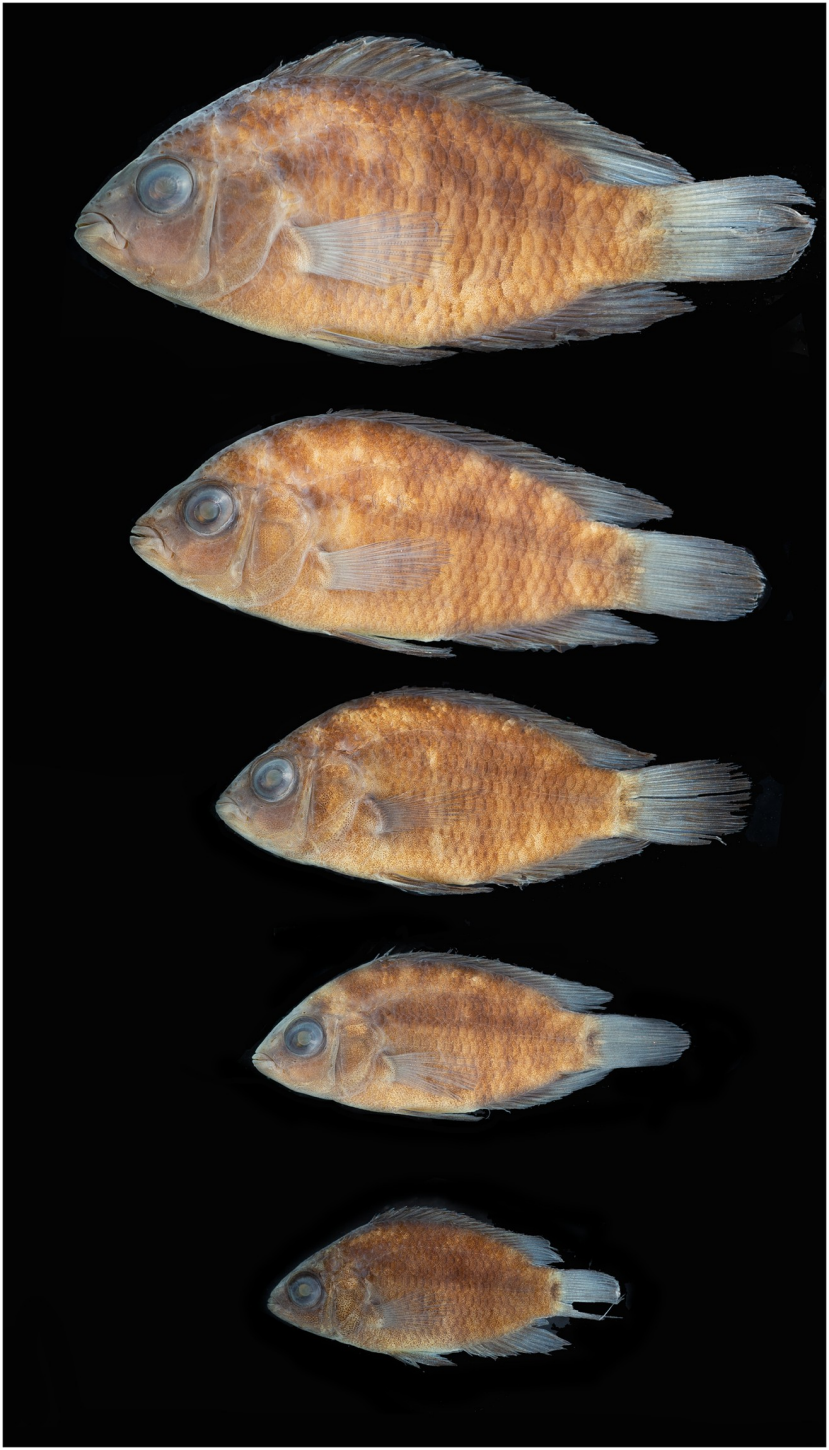
*Australoheros facetus*, ontogenetic series; from bottom to top 23.8–45.3 mm SL; NRM 54218; Uruguay; Maldonado: Laguna del Diario.

Pectoral fin inserted slightly anterior to pelvic-fin insertion, at a distance from pelvic-fin spine corresponding to 1½ length of pelvic–fin base. Pectoral-fin tip rounded; reaching nearly to vertical from genital papilla or to anterior insertion of anal fin. Pelvic fin long, pointed, first soft ray longest, reaching to base of third anal-fin spine, or first soft ray with filamentous tip reaching slightly longer. Small specimens with more rounded vertical fins, pelvic fin with rounded tip, reaching to anal-fin insertion or slightly shorter (Fig 16).

Bases of dorsal, anal, and caudal fins scaled: in adults, dorsal-fin base with basal scales from 9th, 10^th^, 13^th^ or 14^th^ spine, and narrow band with rows of up to five from small interradial scales from next spine in row; soft portion with slightly wider thick basal scale layer, with up to seven scales in a row and basally one or two2 rows. Specimens 27.1–45.2 mm SL (NRM 54218) show gradual development of dorsal-fin squamation from single row of embedded scales close to dorsal-fin base but no scales on fin, to adult condition; 32.4 mm specimen smallest with basal scales from, 14th spine, and single interradial scales posteriorly. Anal fin in adults with single row of basal scales, on soft portion added a dense layer of basal scales, up to four between two rays; exposed anal-fin scales absent in juveniles, present in specimen 45.2 mm SL. Caudal fin with dense layer of scales basally, rows of small interradial scales reaching to middle of fin; in juveniles under 50 mm SL caudal fin scaled only basally.

*Colouration in preservative*. Adult specimens (Fig 14) overall dark with dark beige or brown ground colour, not contrasting with darker vertical bars. Cheek and gill cover dark brown, shading to black ventrally; abdomen, underside of head, black, prepelvic area dark or light grey. Unpaired fins black or dark brown; caudal fin progressively lighter apically, and with approximately round black blotch at base of fin, immediately dorsal to lateral line, or caudal blotch extending across caudal-.fin base, but paler below lateral line. Pectoral fin pale grey, semitransparent. Pelvic fin dark grey to black.

Vertical bars black, slightly wider than Interbars; Bar 1a slightly curved, convex anteriad, between dorsal and ventral margins of caudal peduncle; Bar 2 slightly curved, between posterior bases of dorsal an anal fin; Bar 3 straight, from about middle of base of anal fin dorsally to base of caudal fin; Bar 4 straight, from bases of anterior three spines of anal fin dorsally to base of dorsal fin, but may be interrupted where crossing upper lateral line; Bars 5 and 6 extending dorsally only to E1 scale row or slightly more dorsad; Bar 7 represented by dark pigment close to gill opening, in E 0 and E1 scale rows, and present at occiput as two dark transverse stripes; Bar 9 as two dark parallel stripes crossing snout. A slightly lighter zone along the upper lateral line may form the dorsal limit of ventral sections of bars 5–6; dorsally, bars represented as blotches close to dorsal-fin base. Indistinct narrow black or brown horizontal band on E1 row scales from head to Bar 2; band variably discontinuous or not across Interbars. Black blotch absent from Bar 4 in adults.

Young and juveniles (Fig 16) nearly uniform pale brown with indistinct markings. Vertical bars on side brown; Bars 5–6 not clearly defined, but present from the smallest, about 10–14 mm SL, and in four juvenile specimens four abdominal bars are indicated. An indistinct, irregular dark band along middle of side, and in some specimens an indistinct brown blotch in Bar 4 where crossing band; fins lighter than in adults, and vertical bars extended as blotches onto the base of the dorsal fin in some specimens; Bar 1p at middle of base of caudal fin in smallest specimens.

view.

*Geographical distribution* (Fig 1). Rio Uruguay basin in the Rio Cuareim, lower Rio Paraná in Argentina; short coastal rivers along the coast of Uruguay from Maldonado to Montevideo.

**Fig 17:**
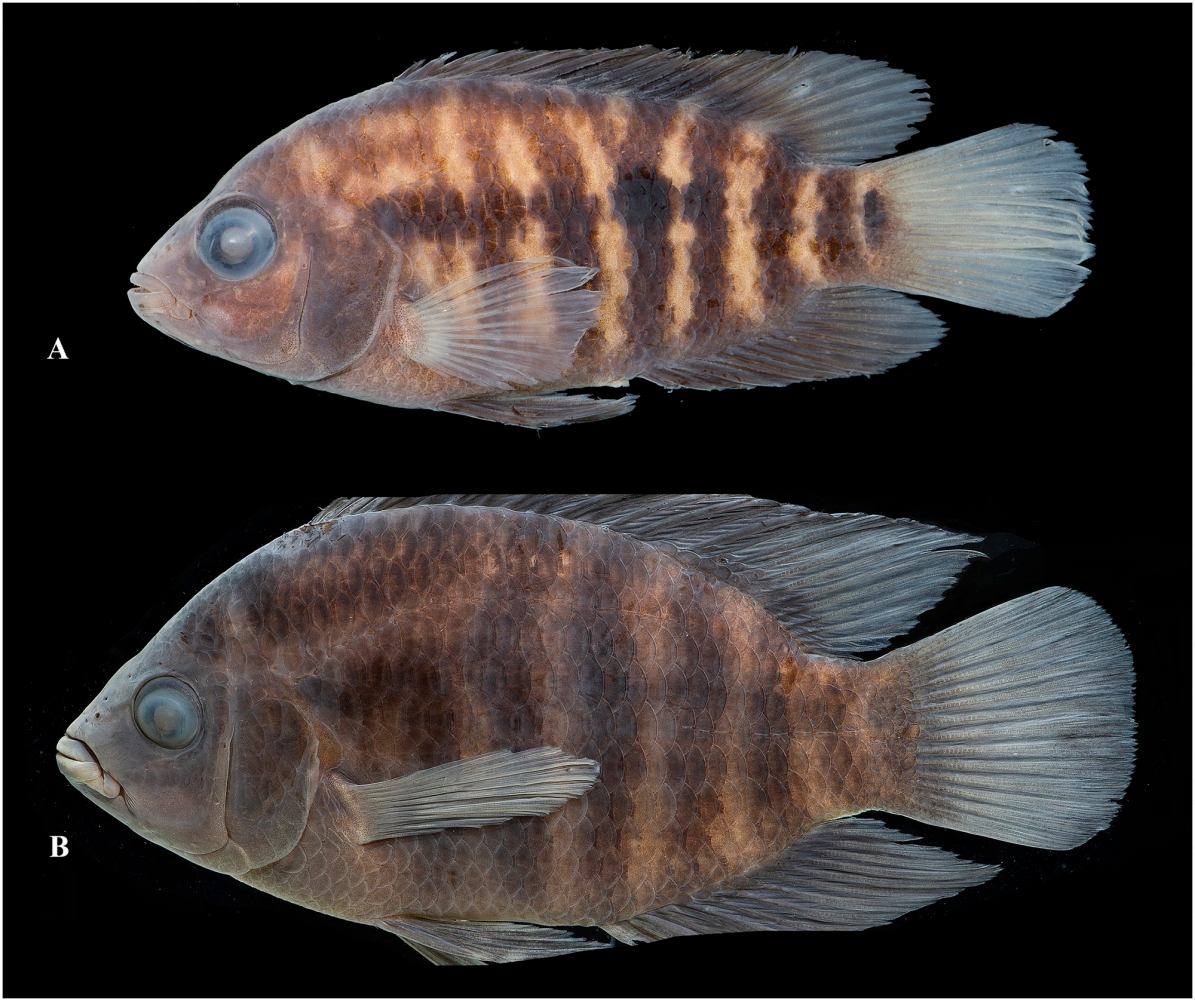
**A.** *Australoheros acaroides*, adult female, 49.1 mm SL; NRM 4799: Uruguay: ‘Pinares Laguna’. **B.** *Australoheros facetus*, young male, 83.5 mm SL: NRM 5441; Canelones: El Pinar: El Pinar, isolated pool.

*Comments*. Říčan and Kullander [3, 4] distinguished two species of *Australoheros* in coastal areas of Uruguay east of Montevideo. One was identified as *A*. *facetus*, characterised by modally 6 anal-fin spines and 4 abdominal bars, but with abdominal bars incompletely separated in their dorsal portion in 80–90% of the specimens. The other species, characterised by 7 anal-fin spines and 4 abdominal bars in 88% of the specimens, was referred to as *A*. cf. *facetus*. The latter species was represented mainly by a single sample of 74 specimens with stated locality El Pinar in Canelones (NRM 48074, 48078, 47999). Almost all specimens in this sample were juveniles or precocious breeders, elongate and with contrasting melanophore pattern (Fig 17A). All other specimens available from El Pinar were deep-bodied like the type series (Fig 17B), and none of those showed a definite breeding colouration.

Revision of the *A*. cf. *facetus* sample suggested that it mainly represented *A*. *acaroides*, as supported by the anal-fin spine count (Table 7: modally 6 in *A*. *facetus*, modally 7 in *A*. *acaroides* and the ‘El Pinar’ sample), and that the locality information may not be correct.

The El Pinar sample was collected by Felipe Cantera in November 2000. The handwritten label accompanying the specimens said ‘Pinares Laguna’, which indicates the name of a lagoon, or a lagoon in a place called Pinares; but does not point clearly to the city of El Pinar east of Montevideo, In the city of Maldonado, there is a quarter with the name Pinares, not far from Laguna del Sauce and Laguna del Diario. The ‘ Pinares’ sample included also one specimen of *A*. *scitulus*, and one specimen with the non-breeding colour pattern of *A*. *acaroides*. At the time, Cantera was travelling widely in Uruguay, collecting fishes and reptiles or guiding tourists. His home in Salinas served as holding compound for live fishes destined for the aquarium trade, shipped principally to Europe. We excluded measurements from the *A*. cf. *facetus* sample from the present analysis because it was possible, even probable, that it was not correctly labelled, and it was not known how long it was maintained in captivity. It may have been collected in Pinares but neither *A*. *acaroides*, nor *A*. *scitulus*, would be expected from either the Laguna del Sauce or Laguna del Diario, in or near Maldonado. The sample more likely came from Valentines, which was an important collecting site for Cantera. The *A*. *scitulus* specimen may be a contamination in a holding tank. Cantera referred to small inland lakes as lagunas (Kullander, pers. comm. with Felipe Cantera), suggesting that the Pinares locality was not necessarily a coastal lagoon. Říčan and Kullander [4] listed the sample on page 5 both in the material list for *A*. *facetus* along with other samples of *A*. *facetus* (top of the page), and separately in the description of *A*. cf. *facetus* (bottom of the page). Their map, fig. 1 [4],) has a symbol for *A*. cf. *facetus* both near Montevideo and Valentines, suggesting that some of the material listed at the top of page 5 includes *A*. *acaroides*. The meristic data for *A*. cf. *facetus* in table 7 [4]), however, seems to have been taken only from the ‘Pinares’ sample.

The greater Montevideo area was represented by samples of *Australoheros facetus* from El Pinar, San José de Carrasco, and the lower Rio Pando. NRM 39509, from Laguna del Tronco, also close to Montevideo, consisted of young adults, relatively deep-bodied, with relatively light ground colour and contrasting light brown vertical bars, except for one female specimen in breeding colour pattern which was relatively slender and with intense black markings and chest. Two specimens in this sample had irregular bars resulting in a count of four abdominal bars. Out of 14 Argentinian specimens 4 abdominal bars were recorded in four specimens. By contrast all other specimens from Montevideo, including El Pinar, Arroyo Pando, and San José de Carrasco, were similar to the specimens from Maldonado being relatively deep bodied, and overall dark without strong contrast between vertical bars and ground colour, and all possess 3 abdominal bars, except that four out of 25 juveniles (NRM 56035, 25, 11–14 mm SL) from the type locality possessed 4 abdominal bars. The contrasting colouration and 4 abdominal bars prevalent in the ‘El Pinar’ specimens may reflect local conditions of growth and early breeding influencing body development and correlated pigment pattern. Similar small-size breeding specimens were observed also in *A*. *acaroides* from the Rio Jacuí basin.

Samples of *Australoheros* from the Rio Uruguay basin in Argentina, Rio Arapey, and tributaries to the Rio Cuareim corresponded in colour and body shape to recently collected specimens from Montevideo and Maldonado.

*Australoheros facetus* occurs in several distinct drainage basins in Uruguay. There is no connection between the Rio Pando and the Laguna del Diario. It seems equally unlikely that there would be any dispersal of *Australoheros* along the extensive shallow sandy playas extending from Montevideo to Maldonado. Maldonado and Laguna del Diario are located at the southern tip of the Cuchilla Grande range which forms the divide between rivers draining to the Rio Negro and ultimately the Rio Uruguay, or to the Rio Santa Lucía, and rivers draining to the Laguna Merín (Yaguarón, Cebollatí, India Muerta, Olimar).

There are no records of *A*. *facetus* from Uruguay between the southeastern localities and the Cuareim localities. This may be a sampling artifact, but it was notable that in Argentina, the species was reported mainly from Buenos Aires and the Paraná basin, with few records from Argentinian tributaries of the Rio Uruguay [86]. Three specimens from the Rio Arapey basin are discussed here under *Australoheros minuano*. They were identified as *A*. *facetus* based on morphology, but one of them had a distinct *mt*-*coI* haplotype. According to Casciotta [86], *A*. *facetus* is also present in Paraguay, but he did not provide any metadata. Říčan et al. [13] reported two *mt-cyb* sequences of *A*. *facetus* from Itapúa (27°05’26.26’’S, 55°,53’13.2’’W) but did not comment on this record. The coordinates point to Arroyo San Roque, a tributary of the Rio Paraná near Encarnación, Paraguay. The *mt-cyb* sequence grouped with those of *A*. *facetus* from Argentina and Uruguay (Fig 13).

Steindachner [33, 88] reported on specimens of *Australoheros* from the vicinity of Montevideo. Six specimens (NMW 17383–17385, 17389–17390) were identified as *Heros facetus*, and one of these was illustrated [88]. The drawing shows a specimen with wide open gape and decoloured caudal fin suggesting that it may have died from suffocation and was preserved after death. An unspecified number of specimens formed basis for the description of a new species, *Heros Jenynsii*, illustrated by Steindachner [88]) with a well-preserved specimen with distinct vertical bars, conforming in scale pattern, colouration, and habitus to *Australoheros facetus* as defined here. The illustration shows the mouth closed and the jaws isognathous.

Říčan Kullander [4] used Steindachner’s description of *Heros facetus* as a diagnosis for *A*. *facetus*, emphasising the upwards directed mouth and projecting lower jaw, while still including *Heros jenynsii* in the synonymy of *A*. *facetus*. Říčan and Kullander [4] characterised *A*. *facetus* in a wide sense by unique prognathous lower jaw, usually four abdominal bars, and unique reduced scale cover on the dorsal and anal fins.

Říčan and Kullander [4] did not have access to fresh material of *A*. *facetus* from the type locality. The present material included that of Říčan and Kullander, excluding the ‘El Pinar’ sample, but with addition of more specimens from additional localities, most important of which are large specimens from El Pinar and San José de Carrasco, and a series of specimens from the Laguna del Diario.

Eleven of the 12 measured specimens from Laguna del Diario had 6 anal-fin spines, and only one had 7 spines; among the 25 juvenile specimens not measured, 17 had 6 spines, and eight had 7 spines. Whereas there evidently were differences in the modal number of anal-fin spines between different geographical areas, count ranges overlapped, and consequently did not support recognition of different species in Uruguayan coastal rivers. Jenyns [29] counted 6 anal-fin spines in the holotype of *A*. *facetus*. Steindachner (1870) reported 6 anal-fin spines in his material from near from Montevideo. Argentinian specimens, however, showed more variation (six with 7, five with 6, as did specimens from the Rio Cuareim (one with 5, four with 6 and two with 7). While the vast majority of Uruguayan *Australoheros acaroides* possessed 7 anal-fin spines, rarely 6 or 8, anal-fin spine number is indicative of species, but not unique for either species.

*Australoheros facetus* is one of the earliest tropical fish species to have been kept and raised in Aquarium. It was imported from ‘La Plata’ to Germany in 1894, and reproduced in the same year [28]. There was apparently an earlier importation, to France in 1889 [28], but in that case, the geographical origin was not reported and the species identity remains uncertain. In the aquarium hobby in the United States and Europe, *A*. *facetus* was known as chanchito, a Latin American Spanish word meaning piglet. The earliest use of the name chanchito in aquarium literature may be traced to Dürigen in 1894 [89], who explained the name as referring to the compressed body and high arched (‘hochgewölbten’) back, reminding of a swineback ridge. In 1955, Axelrod and Schultz [90], with an illustration showing *Amatitlania nigrofasciata* (Günther, 1867), used the English name Chameleon cichlid (in limited use in German as Chamäleonsfisch employed by Geyer [91] in 1896 and perhaps earlier), instead of chanchito, and this name replaced chanchito in English literature.

The vernacular names chanchito, chanchita, as well as castañeta, are confirmed for Argentina [14, 92]), but only castañeta for Uruguay [93]. It seems more likely that the name refers to the sound emitted by specimens being handled than to suine morphology. Based on the vernacular name, the first individuals reaching the German ornamental fish market came from Argentina although Brazil was also noted as origin of late 19th Century imports to Germany [91]. No particular vernacular name seems to be recorded for species of *Australoheros* in Brazil, where cichlids are addressed with the general acará or cará. Malabarba et al. [94] used cará amarelo for *A*. *acaroides*, but it looks like a recent construction. Santos [95] used Acará-cascudo for *Cichlasoma facetum*], but this name has been applied also on species of *Cichlasoma* [96].

*Material examined*. NMW 17383 (1), 17384–17385 (2), 17389 (1), 17390 (1); Montevideo, registered March 1869.–NMW 17324–17325 (2), 17324 (1), 58722 (1), 17326 (1); Montevideo; registered March 1869; syntypes of *Heros Jenynsii*.—Argentina: Rio de la Plata coastal streams: NRM 33035, 2, 96.6–108.3 mm SL; Buenos Aires: Rio de la Reconquista, 100 m downstream of Represa La Reja, 3 km S of village La Reja; 34°41’S 58°50’57’’W; S. Körber 18 Mar 2008.–NRM 33033, 3, 78.2–108.6 mm SL; Aquarium, originally from Buenos Aires: Río de la Reconquista, La Reja, below dam; S. Körber, unknown date.—Argentina: Rio Paraná basin: ZFMK 39750–39751, 2, 97.5–106.2 mm SL; Santa Fé: Lago del Sur in Santa Fé, 31°6.5’S 60°7.5’W [31°39’52.6"S 60°42’47.9"W]; S. Koerber et al., 18 Jan 2000.—ZFMK 39781, 3, 109.1–114.6 mm SL.—Argentina: Rio Uruguay basin: NRM 33050, 1, 40.6 mm SL; Entre Ríos: Gualeguaychú: Arroyo El Ñancay, where crossing RN14, 33°22’51’’S 58°44’14’’W; S. Körber, 28 Sep 1995.—ZFMK 39517–39518, 2, 83.0–85.1 mm SL: Entre Ríos: Colón: Ubayay: Estancia Los Monigotes on road to harbour, 31°49.64’S, 58°10.74’W. Koerber, R. Filiberto, J.O. Fernandez Santos, 2 Feb 2002.—ZFMK 39771–72, 1,95.9 mm SL; Entre Ríos: Colón: Ubayay: Estancia Los Monigotes downstream of ZFMK 39517–39518, 31°49’64’S, 58°10.74’’W; S. Koerber, R. Filiberto, J.O. Fernandez Santos, 2 Feb 2002.—Uruguay, Rio Uruguay basin: NRM 51073, 1, 24.7 mm SL: Artigas: Rio Cuareim basin: Escuela Piedra Pintada [about 30°30’2"S 56°26’28"W]; P. Laurino et al.,16 Aug 2003.—NRM 52237, 4, 125.2–147.4 mm SL: Artigas: Rincón de Pacheco, 1 km from estancia buildings, cañada tributary of the Rio Cuareim, 30°40’22"S 56°10’51"W; S. Kullander et al., 9 Oct 2004.—NRM 52565, 2, 8.6–101.7 mm SL; Artigas: Arroyo Catalán Grande, tributary of the Rio Cuareim, below bridge on road 30, 0°50’30.6"S 56°14’20"W; S. Kullander et al., et al. 11 Oct 2004.—NRM 52635, 1, 102.5 mm SL; Rio Cuareim basin: tributary of the Arroyo Macedo, about 17 km on dirt road from road 30 to Rincón de Pacheco, 30°39’15"S 56°18’43"W; S. Kullander et al., 8 Oct 2004.—NRM 70401, 35 juveniles; Artigas: Arroyo Falso Mandiyu, Ruta 3, Km 589.850, below bridge, 30°33’06"S 57°40’06"W; S. Kullander et al., 15 Feb 2007.—Uruguay, Rio de La Plata coastal streams: NRM 54218, 5: 23.8–45.3 mm SL; NRM 56598, 1, tissue NRM 56606, 1, tissue; NRM 56607, 1, tissue; Maldonado: Laguna del Diario, 34°54’19"S 55°00’29"W; S. Kullander et al., 31 Oct 2005.—NRM 55720, 7, 55.4–145.4 mm SL; NRM 56035, 25, 11–14 mm SL; NRM 56430–56446, 26, not measured; Maldonado: Laguna del Diario, 34°54’17S 55°00’29"W; S. Kullander et al., 21 Oct 2007.—NRM 37036, 1, c&s; NRM 37038; 1, c&s [macerated]; NRM 37039, 1, c&s [macerated]; NRM 37040, 1, c&s; NRM 39509, 14, 58.2–98.1 mm SL; Canelones: Arroyo Pando basin; Laguna del Tronco at Salinas, 3.7 km from Montevideo on Ruta Nacional 1 to Atlántida, 500 m off road toward Pando, 34°46’00"S 55°54’00"W; F. Cantera et al., 20 Jan 1997.—NRM 39552, 1, 102.9 mm SL; Canelones: Laguna Arenera de Carrasco at San José de Carrasco, 18 km on road Montevideo-Atlántida, 200 m off Ruta Nacional 1, 34°51"S 56°01’0"W; F. Cantera et al., 15 Jan 1997.—NRM 43453, 6, 13.7–26.9 mm SL; Canelones: Salinas, Arroyo Tropa Vieja, about 34°47’232"S 55°52’1.6"W; F. Cantera, 18 Dec 1997.—NRM 43485, 1, 143.5 mm SL; Canelones, San José de Carrasco, easternmost pond, 34°50’08"S 55°59’16.0"W; F. Cantera, 29 Nov 1997.—NRM 50997, 1, juv.; Canelones: Arroyo Caracoles, on road 8, Km 59, at margin of Soca village, under bridge; S. Kullander et al. 15 Oct 2004.—NRM 51068, 5, 30.3–37.9 mm SL; Canelones: Arroyo Pando, side arm; P. Laurino et al., 23 Mar 2003.—NRM 51069, 2, 48.2–54.1 mm SL; Canelones: Arroyo Pando, stream; P. Laurino et al., 23 Mar 2003.—NRM 52238, 8, 24.9–64.4 mm SL; Canelones: Arroyo Sarandí on Highway 18, Km 54, under bridge, 34°45’02"S 55°40’04"W; S. Kullander et al., 15 Oct 2004.—NRM 52591, 2, 76.0–95.2 mm SL; 54412, 2, 83.5–103.1 mm SL; Canelones: El Pinar: El Pinar, isolated pool; 34°47’53"S 55°53’03"W; S. Kullander et al., 13 Oct 2004.—Portugal: Rio Guadiana basin: MUHNAC uncat., 1, 107.6 mm SL; Algarve: Ribeirão Odeleite alto at Vila Real; J. Nadeira, J. Alves and E. Santos.—MUHNAC uncat., 1, 93.6 mm SL; Alto Alentejo: Évora harbour; F. Barroso, G. Texeira and V. Ferreira, 31 Jan 1981.—MUHNAC uncat., 76.1 mm SL; Baixo Alentejo: Rio Ardila at Santo Amador; 25 Jul 1985.—MUHNAC uncat. 6, 8.33–115.8 mm SL; Alto Alentejo: Rio Degebe at Amieiera; 5 Jun 1980; G. Teixeira and V. Ferreira.

#### *Australoheros oblongus* (Castelnau, 1855)

*Chromys oblonga* Castelnau, 1855 [30]: 14 (holotype MNHN 9485; type locality le Tocantins (Province de Goyaz).

*Heros autochthon* Günther, 1862 [31]: 299 (syntypes BMNH 1961.7.7.2–4, one syntype not found; type locality Brazil).

*Australoheros barbosae* Ottoni & Costa, 2008 [6]:213, (holotype, UFRJ 7558; type locality Brazil: Estado de Minas Gerais: between Município de Passa Vinte and Santa Rita da Jacutinga: tributary from [sic] Rio Bananal, rio Preto basin, 24 km from Passa Vinte, street between Passa Vinte and Santa Rita da Jacutinga).

*Australoheros paraibae* Ottoni & Costa, 2008: 222 [6], fig. 11 (holotype UFRJ 7559; type locality Brasil: Estado de Minas Gerais: Município de Juiz de Fora: stream tributary to rio do Peixe, between Toledo and Torreões).

*Australoheros robustus* Ottoni & Costa, 2008: 224, fig. 12 [6] (holotype MNRJ 32180; type locality Minas Gerais: Mar de Espanha: stream Cachoeirinha, tributary from [sic] córrego do Areia, rio Paraíba do Sul basin; spelled *A*. *rostrunrobustus* on p. 212).

*Australoheros macacuensis* Ottoni & Costa, 2008: 216, fig. 8 [6] (holotype UFRJ 7254; type locality Brazil: Estado do Rio de Janeiro: Município de Cachoeiras de Macacu: rio Japuíba).

*Australoheros mattosi* Ottoni, 2012:85, fig. 1 [6] (holotype UFRJ 0752; type locality Brazil, Minas Gerais state: tributary of rio das Velhas, between Santana do Pirapama and Jequitibá, rio São Francisco basin).

*Australoheros montanus* Ottoni, 2012 [9]: 86, fig. 3 (Holotype MNRJ 32555; type locality Brazil: Rio de Janeiro state: Carmo, córrego Tanque, tributary of the rio Paraíba do Sul, locality of Passa Três (21°49’S/42°32’W).

*Australoheros mattosi* Ottoni, 2012 [9]:85, fig. 1 (holotype UFRJ 0752; type locality Brazil, Minas Gerais state: tributary of rio das Velhas, between Santana do Pirapama and Jequitibá, rio São Francisco basin).

*Australoheros tavaresi* Ottoni 2012 [9]: 92, fig. 7 (holotype MZUSP 50675 A; type locality Brazil: São Paulo state: Guarulhos, lagoon on the margin of the rio Tietê).

*Definition*. Based on the position in the *mt*-*cyb and mt-coI* trees and minimum uncorrected *p*-distance in *mt-coI* exceeding 2% from all other species of the genus, *Australoheros oblongus* is a distinct evolutionary lineage. No morphological autapomorphy was detected; however, *A*. *oblongus* is geographically isolated from all other species of *Australoheros* except *A*. *ipainguensis* from which large adults can be distinguished by a steep frontal contour with a slight indentation at level of orbits; only exceptionally presence of Y bar; caudal spot at middle of caudal-fin base instead of usually entirely or predominantly located on upper lobe of caudal fin; lateral band typically irregular and narrow, but may be represented by dark blotches similar to the zipper band in southern species of *Australoheros*.

**Fig 18:**
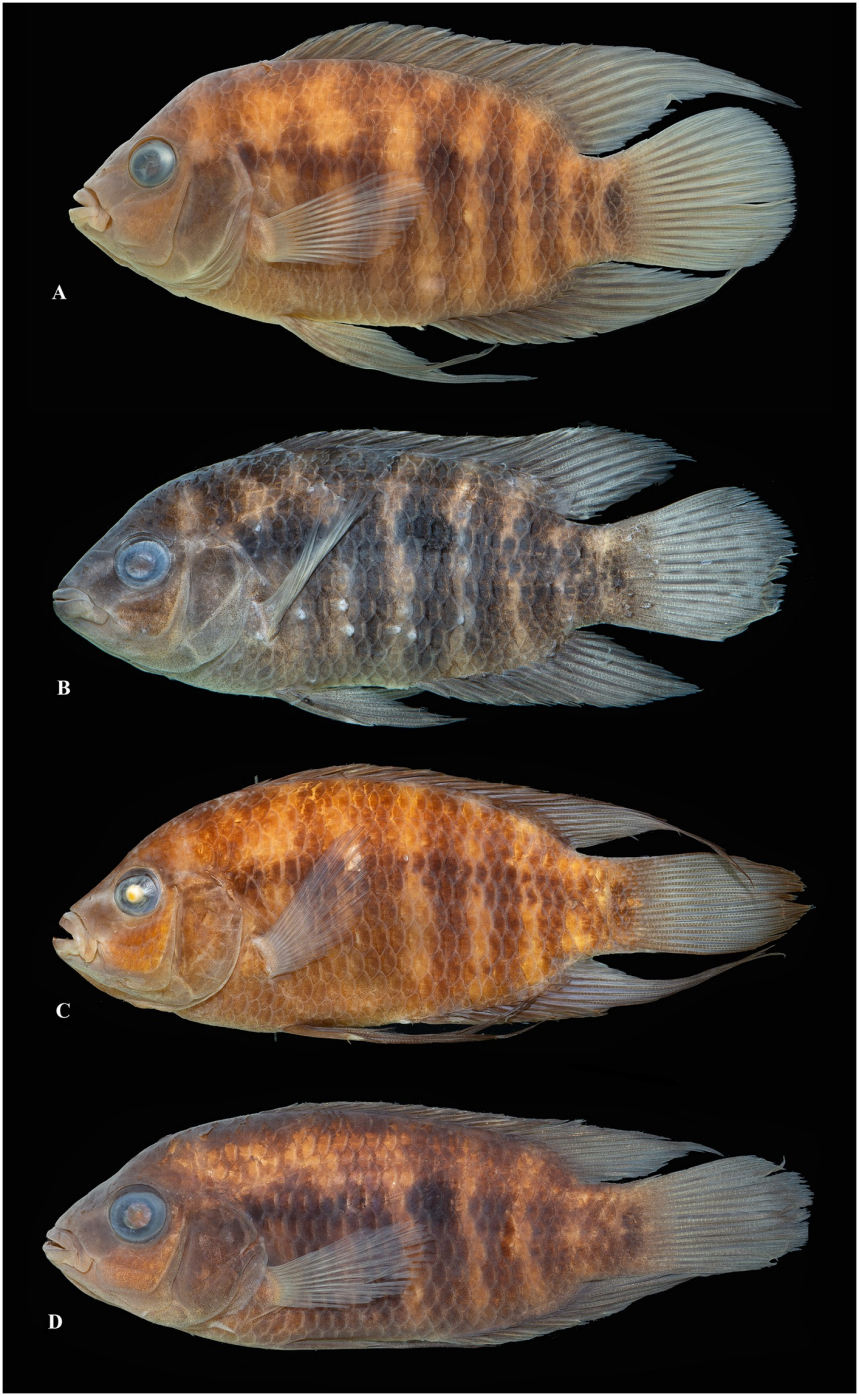
*Australoheros* oblongus. **A.** Adult male, 78.6 mm SL; MCP 287195; Brazil: Minas Gerais: Abadia dos Dourados: Rio Grande drainage: Rio Preto on road Palmito–Abadia dos Dourados. **B**. Adult female, 76.9 mm SL; MCP 49007; Brazil: Minas Gerais: Cardeal Mota: Rio Cipó drainage, arroio on road Jaboticatubas to Cardeal Mota. **C.** Adult male, 84.0 mm SL; LBP 17525; Brazil: Minas Gerais: Bom Jardin de Minas: Rio Paraíba do Sul drainage: Cachoeira do Pacau, Ribeirão da Bom Jardim de Minas, reversed. **D.** Adult male, 67.4 mm SL; MCP 31877; Brazil: Minas Gerais: Ouro Preto: upper Rio Doce basin: Lagoa do Manso in Parque Estadual de Itacolomi, reversed.

*Description of sample*. Meristic data are given in Tables 3–9 proportional measurements in Table 11. For variation in body shape, see Fig 18.

Sexes isomorphic except for vertical and pelvic fins slightly longer in some large males than in large females, and genital papilla in males slender, conical, in females wider, blunt. Moderately elongate, dorsal and ventral contours approximately equal in curvature; frontal contour ascending slightly curved or straight, continuous with slightly curved dorsal-fin base contour; some large specimens with straight dorsal-fin base contour and steep frontal contour raising to sharp bend anterior to origin of dorsal-fin base; but nuchal protuberance not observed; slight indentation in frontal contour at level of orbit present or absent. Prepelvic contour slightly curved, similar to predorsal contour. Caudal peduncle contours slightly curved or straight. Abdominal contour straight horizontal, except slightly convex in females. Anal-fin base slightly convex, ascending. Head short, laterally compressed. Snout short, blunt, subtriangular in lateral aspect, narrowly rounded in dorsal aspect. Mouth terminal, forward directed, at level of lower margin of orbit or slightly more dorsal on specimens with large eyes. Lips moderately thick. Jaws equal in anterior extension; maxilla and premaxilla not reaching to vertical from anterior margin of orbit. Orbit removed from frontal contour, in middle of head length, in upper half of head. Teeth in outer hemiseries in upper/lower jaw 9–14/10–18. Inner teeth in 2 rows, exceptionally 3 rows in lower jaw. Bicuspid teeth observed in holotype only. Gill rakers externally on first gill-arch 2–3 epibranchial, one in angle and 5–7 ceratobranchial. Microbranchiospines present externally on 2nd to 4th gill-arch.

Scales on body finely ctenoid. Predorsal midline scales about 10–14, about half size of flank scales, covered by skin, only part with exposed margin, irregularly arranged, weakly ctenoid. Cheek scales in 3–4 (rarely 2) rows, cycloid, covered by skin. Opercular scales cycloid, covered by skin or margin partly exposed. Accessory lateral line scales absent from caudal fin. Between first upper lateral line scale and dorsal-fin origin 3 large scales. Between last upper lateral line scale and dorsal fin one large and one small scale. Prepelvic scales about half size of flank scales, embedded in skin, without free margin. Lateral chest scales ctenoid, with exposed margin, about 2/3 size of flank scales. Fin scales ctenoid. Row of minute scales along dorsal-fin base from 9th–13th spine, interradial scales from between 11th to 15th spine caudad, on soft fin in one or two rows with up to 7 scales between two soft rays, scales present also on last two interradial membranes. Anal fin with narrow basal scale layer from 4th to 6th spine, on soft-rayed portion up to 5 scales in interradial row.

Dorsal–fin spines increasing in length to 6th from which subequal, last spine longest, soft dorsal-fin rounded or subacuminate in young; in large adults with long pointed tip, 4th or fifth ray longest, reaching to end of caudal fin or slightly longer. Soft anal fin rounded or subacuminate in young; in adults with long pointed tip, 4th or fifth ray longest, reaching to end posterior 1/4th of caudal fin. Pectoral fin rounded, 4th or 5th ray longest, reaching to vertical from genital papilla or base of first or second anal-fin spine. Pelvic fin pointed, first ray longest; in young reaching to base of 3rd anal-fin spine; in adults produced, at most to soft anal fin-fin base. Caudal fin rounded.

*Colouration in preservative*. Ground colour fawn or pale grey. Bars 1(a, p) to 6 present, dark brown or dark grey, 2–3 scales wide; Bar 6 absent above lateral band or extending only short distance above it; Interbars 1–2 scales wide; lateral band on E1 scale row extending from gill cleft caudad to Interbar 4; Bar 5 integer, extending dorsally at most to dorsal-fin base. Bars vertical or dorsal and ventral portions of bars 2–4 slightly caudally inclined. Bars 5 and 6 exceptionally forming Y-mark dorsally. Dorsal portions of Bar 7 present, expressed as short rostrad inclined brown or grey bars crossing anterior dorsal-fin base and occiput, respectively. Midlateral black or dark brown blotch present in Bar 4 on E1 scales and parts of adjacent scales; corresponding portions of bars 2 and 3 may be slightly widened or more intensely pigmented but distinct spots absent from bars 2 and 3. Bars 2–4 extending dorsad onto base of dorsal fin. In Bars 2–6, vertical black stripe may be present at middle or posterior margin of scales below scale row 0. Caudal spot (Bar 1p) distinct, forming vertical bar across middle of caudal-fin base. Vertical fins pale grey, semitranslucent. Pelvic fin dark grey distally, lighter medially.

*Geographical distribution* (Fig 1). Small streams in the Rio Paraíba do Sul basin; Rio Tietê and other basins of the Rio Grande (headwaters of the Rio Paraná); tributaries of the upper Rio São Francisco and upper Rio Doce; Rio Guandi and Rio Macacu basins.

*Comments*. The holotype of *Australoheros oblongus*, 96.9 mm SL, was in a very bad state of preservation. The general habitus, conforming to *Australoheros*, was well preserved but the body was soft and completely decoloured. Almost all scales were lost; many fin rays were broken and several were detached from their pterygiophores. Scale pockets showed scales to have been proportionally large, about 24 along the middle of the side, predorsal scales 10 or 11, cheek scale rows 4. Dorsal-fin rays XV.10. Anal-fin rays VII.8. Vertebrae 13+13 = 26. The anterior jaw teeth were slightly enlarged, and in the upper jaw also bearing a minute secondary cusp.

According to Castelnau [30] the single specimen basis for the description of *Chromys oblonga* was in a bad state of preservation already at the time of description, lacking scales and pectoral fins. It was possibly for that reason that Castelnau did not provide an illustration. The holotype is clearly a specimen of *Australoheros*, and the only record of *Australoheros* from the RioTocantins basin. Both Pellegrin [36], who had access to the holotype, and Regan [37] noted the similarity to *Heros autochthon*.

Three heroine cichlid genera, *Pterophyllum* Heckel, 1840, *Hypselecara* Kullander, 1986, and *Mesonauta* Günther, 1862, were recorded from the Araguaia and Tocantins rivers. Among them, *Pterophyllum* is distinguished by its very small scales and rounded body shape, and very long dorsal, anal, and pelvic fins [53]. Species of *Mesonauta* are very different in body shape from the holotype of *A*. *oblongus*, being deep-bodied, strongly compressed, with pointed snout and about straight predorsal contour; also possessing very long pelvic fin [97]. *Hypselecara temporalis* (Günther, 1862), the only species of that genus recorded from the Rio Tocantins, redescribed by Kullander [53], is similar in fin-ray counts to the holotype of *A*. *oblongus*, and other species of *Australoheros* (dorsal-fin rays XVI–XVII.10–12; anal-fin rays. VI–VIII.8–10; [53]. It has relatively large scales but still slightly more than in most species of *Australoheros* (25–28 [53] vs 23–26; 27 in *A*. *mboapari*; about 24 in the holotype of *A*. *oblongus*); and more vertebrae (13+14, 14+13, 14+14 [53] vs usually 13+13 as in the holotype of *A*. *oblongus*). *Hypselecara temporalis* differs in body shape and colour pattern from all species of *Australoheros*. Juveniles are elongate, with markedly pointed snout; adults are moderately to conspicuously deep-bodied, with gently rounded dorsal and ventral contours, with a straight ascending, steep, and only slightly indented frontal contour. The body depth range 45.5–58% SL is higher than in any species of *Australoheros*, and in the holotype of *A*. *oblongus* (about 25%). The colour pattern includes a large dark blotch on the middle of the side, a horizontal lateral band, and only indistinct vertical bars. The colour pattern of the holotype of *A*. *oblongus*, however, is unknown. Bicuspid teeth, present in the holotype of *A*. *oblongus* were recorded from species of *Mesonauta* and *Pterophyllum*, but not from *Hypselecara* [53, 97].

The overall shape of the holotype of *A*. *oblongus*, the markedly concave frontal contour, the relatively large scales, large symphysial teeth, bicuspid upper jaw teeth, and fin-ray counts altogether suggest that it represents a species of *Australoheros*. The expedition during which it was collected started from Rio de Janeiro and went north by way of Magé, Mariana, Ouro Preto, Belo Horizonte, and Catalã before reaching Goiás and the Rio Tocantins basin [98, 99]. It thus passed the Rio Paraíba do Sul, tributaries of the Rio Doce, the Rio São Francisco, and the Rio Paranaíba (Rio Paraná basin). It remains possible that the specimen of *A*. *oblongus* was collected on some other occasion and erroneously attributed to a collection made in the Rio Tocantins, or that Castelnau’s observations in the Tocantins basin were made on some other cichlid species obtained in the Rio Tocantins, e.g. *Hypselecara temporalis*, mistakenly confused with an *Australoheros* specimen from another collection site. No other specimens of *Australoheros* have been reported from the Rio Tocantins or Amazon basins, supporting the hypothesis that the holotype of *Chromys oblonga* was not collected in the Rio Tocantins basin. The possibility of mislabelling must also be considered; despite Castelnau’s (1855: 14) claim ‘remarqable par la forme très allongée de son corps’, MNHN 9484 is not conspicuously elongate. The body proportions (97 mm SL; approximately 120 mm TL; body depth 38 mm), however, agreed with Castelnau’s description (length12½ cm, body depth 4 cm). The meristic data were also similar or identical. Counts and measurements did not refute the identification of the specimen as the holotype of *A*. *oblongus*.

Günther [31] described *Heros autochthon* in quite some detail, indicating that the description was based on four specimens (listed as *a*, *b*, *c*, *d*), presented by Lord Stuart. Regan [37] referred *H*. *autochthon* to *Cichlosoma* and listed three specimens of *Cichlosoma autochthon* with Brazil, Lord Stuart as provenance. To this he added one specimen without data, two from Theresopolis [state of Rio de Janeiro], collected by Göldi, and one from Porto Real ((state of Rio de Janeiro), collected by Hardy du Dréneuf. In the rest of the paper, type specimens were clearly indicated, but types were not mentioned in the entry for *Cichlosoma autochthon*. The significance of Regan’s list is that in 1905 only three specimens marked as donated by Lord Stuart were identified in the BMNH collection. These were most likely BMNH 1961.7.7.2–4, which were registered by M. Hudson and Humphry Greenwood in 1961. Greenwood left a note with that object that ‘The original description lists 4 types. The fourth specimen listed in Regan’s catalogue has been examined and no justification found for including it.’ ‘Regan’s catalogue’ refers to a catalogue maintained by Regan separate from the museum register, and into which he pasted cataloguing data from his publications. The fourth specimen was probably BMNH 1961.7.7.6, which was not registered on that date, but later. It was still not registered in 1992, when SK examined the specimen. The specimens in BMNH 1961.7.7.2–4 were somewhat soft and had lost much of the dark pigmentation, but were otherwise in good condition, and clearly represent a species of *Australoheros*. BMNH 1961.7.7.6 was in a poor state of conservation. It was full of encrustations and decalcified as a consequence of having been stored in zinc chloride. It represents a species of *Australoheros*, but because of the difference in preservation, its status as syntype may be doubted. Throughout its catalogue record it has been separated from the three specimens reasonably certain to be syntypes of *H*. *autochthon*.

Based only on photographs of the specimens in the type series of *A*. *autochthon*, Ottoni [9] stated that three of the specimens did not allow ‘positioning’, probably means identification at genus level)] on account of the state of preservation, and declared that the fourth specimen ‘does not belong to the genus *Australoheros*, not possessing interruptions of trunk bars 6–7, probably, even not belonging to the tribe Heroini.’ Nonetheless, Ottoni declared the fourth specimen as lectotype of *Heros authochthon*. This supposedly resulted in a lectotype for a non-heroin cichlid species which was not identified; an awkward situation not covered by the International Code of Zoological Nomenclature [43]. *Australoheros*. Ottoni also claimed that the likely type locality of *Heros autochthon–*Rio de Janeiro–based on the fact that Lord Stuart was based there, is unjustified, but did not present an alternative. Rejection of both the locality and the generic identification would remove the need to include *A*. *autochthon* in assessing the validity of the many nominal species meanwhile described from the Sudeste. As obvious from our examination of the syntypes, the three specimens in BMNH 1961.7.7.2–4, were in relatively good condition considering their age, and represent a species of *Australoheros*. The specimen selected as lectotype by Ottoni, was in a poor state of conservation, but still recognised as representing a species of *Australoheros*. There is, however, no evidence that the proposed lectotype was ever part of the type series. Consequently, Ottoni’s lectotype designation was a suboptimal choice because of the poor state of preservation and unproven type status of the specimen. It was also an unnecessary act because it did not resolve any taxonomic problem, but rather could have created a problem if indeed the selected specimen were of a different species. In the absence of any contrary evidence, and given the species identity, metadata stating that the syntypes were donated by Lord Stuart, with locality Brazil makes it most plausible that the type series was indeed acquired by Lord Stuart in Rio de Janeiro.

Charles Stuart, Lord Stuart de Rothesay (1791–1845) was a British diplomat, for some time Envoy to Portugal and Brazil. He visited Rio de Janeiro in July 1825 to negotiate the independency of Brazil on behalf of the King of Portugal. We have no information about later visits to Brazil. The BMNH collection is in possession of one more fish specimen (*Sargus unimaculatus*) and several bird specimens [100]. Gray [101]) listed 21bird objects in BMNH donated by Lord Stuart, each with 1–2 specimens. Seventeen of them had Brazil as locality, the other four South America. These records show that Lord Stuart possessed a sizeable collection of specimens from Brazil, not just some odd fish specimens. It is not documented exactly when he acquired these specimens, when they were acquired by the BMNH, or whether they were obtained by Stuart in Brazil or given to him by some other person before or after his visit to Brazil. There is, however, no better explanation, based on the scant metadata and species concerned, than that at least those with Brazil as locality were acquired in connection with Lord Stuart’s brief visit to Rio de Janeiro in 1825.

Examination and X-radiographs of all three syntypes of *H*. *autochthon* supported the identification as specimens of *Australoheros*. Whereas live colours are not known, fin-ray, scale, and vertebral counts fall within the ranges of *Australoheros*. There are no character states known that separate *Heros autochthon* from *Chromys oblonga*. Both type series were collected in southeastern Brazil, an area from where two species of *Australoheros* are recorded. The type specimens are relatively elongate, similar to *Australoheros* in general, and present a steep, slightly indented frontal contour, present also in some specimens from inland localities in the Sudeste and different from the generally more rounded profile and steep, straight frontal contour of *A*. *ipatinguensis*. Shape variation within *Australoheros* was extensive, however, so that reliable distinguishing characters could not be established based on body contours or proportions. Nonetheless, we synonymise here *Heros autochthon* with *Chromys oblonga* for want of any indication of species distinctness. To this synonymy we add *A*. *barbosae*, *A*. *macacuensis*, *A*. *mattosi*, *A*. *paraibae*, *A*. *robustus*, and *A*. *tavaresi*.

Table 21 summarises a selection of characters taken from the species diagnoses in the original descriptions of nominal species that list differences among Sudeste species [5, 6, 8–11]. The diagnoses are complex, e.g., more than 100 words in one sentence for *A*. *sanguineus*, incorporating author citation and external references; consequently mainly numerical characters were added to the table, and where explicit comparison with a taxon was lacking–primarily *A*. *ribeirae*, the first in the suite–data was added from the description of that species. Ottoni et al. never presented a morphology-based phylogenetic analysis. In their molecular analysis [47], it is also absent. Ottoni et al. [47] did not adjust the morphological definitions according to the novel MOTUs, or explain why some samples could not be identified to species; our review thus is based on the original diagnoses, but may then not reflect the conclusions of the molecular analysis.

**Table 21:** Comparative matrix of character states based on a semi-random subset of data in diagnoses of nominal species from the Sudeste with emphasis on species 12–19 as numbered here.

Nominal species	D SPINES	D RAYS	A SPINES	A RAYS	RIBS	P RAYS	EPIB 1	EPIB 2	D RADIALS	A RADIALS	E1 SCALES	ECTOPT	CPLEN	LOWER JAW	SNOUT	BAR 7 WIDTH	BODY DEPTH	A SPINE	MOUTH	PREORB	A SCALES	LAST D SPINE
***A*. *ipatinguensis***	15	10–11	7	9	10	14	LONG	Short	24–25	13	25–26	Narrow	6.6–8.0	17.0–19.2	Common	Same width	47.3–51.2	14.3–15.6	Isognath	56.6–60.3	From spine 6	14.2–16.6
***A*. *macaensis***	16	10–11	7–8	8–9	11	14	Long	Long	25	13–14	25–28	Wide	7.6–10.8	20.0–22.9	Common	same width	44.0–48.2	13.6–17.1	Isognath	55.0–58.8	From spine 6	12.8–16.4
***A*. *muriae***	15	11–12	6–8	9–10	10–11	14–15	LONG	Long	24–25	N/A	27–29	Wide	7.1–8.9	19.4–27.6	Common	Same width	43.8–50.1	14.3–17.0	Isognath	56.3–72.7	From spine 6	14.0–17.3
***A*. *autrani***	15–16	10–12	7–8	9–10	10	14	LONG	Long	25–26	13–14	25–28	Wide	10.2–11.2	18.0–20.6	Common	same width	45.7–50.9	15.9–17.1	Isognath	54.9–60.8	From spine 6	
***A*. *saquarema***	16	10–11	7	9	10	14	LONG	Long	25–26	14–15	25–27	Wide	6.7–9.0	20.0–21.9	Common	Same width	44.0–48.2%	15.9–17.1	Isognath	66.0–69.1	From spine 6	13.9–16.9
***A*. *capixaba***	15–16	10–11	6–7	8	10–11	14	LONG	Long	23–25	12–13	26–28	Wide	9.6–11.4	16.8–22.2	Common	Same width	42.6–50.3	14.0–17.2	Isognath	54.1–68.8	From spine 6	13.0–18.1
***A*. *perdi***	14–16	9–11	6–8	8–10	11	11–13	LONG	Long	22–24	13	25–27	Wide	9.3–11.6	19.6–28.4	Common	same width	40.0–56.0	14.4–20.8	Isognath	61.8–84.0	From spine 6	13.3–20.4
Total variation	14–16	10–12	6–8	8–10	10–11	11–15	Long		22–26	12–15	25–29		6.6–11.6	16.8–28.4			40.0–56.0	14.0–20.8		54.1–85.8		13.3–20.4
***A*. *barbosae***	16	10–11	7–8	9–10	**11**	14–15	LONG	Short	24–26	13–14	25–28	Wide	5.5–8.7%	17.3–23.3	Common	same width	44.6–49.0%	13.8–15.9	Isognath	60.5–65.3	From spine 6	13.4–15.8%
***A*. *macacuensis***	16	10–11	7–8	8–9	**10–11**	14	LONG	LONG	24–25	13	25–28	Wide	5.1–7.9%	20.3–22.9%	Common	Posterior wider	46.6–49.8%	13.8–16.5%	Isognath	62.2–65.4%SL	From spine 6	12.6–15.9%
***A*. *paraibae***	15–16	10	7–8	8–9	**10**	13–14	LONG	Short	25	13–14	26–128	Wide	6.4–8.1%	19.8–21.9	Common	Same width	42.6–46.1%	12.2–13.3%	Isognath	60.4–65.2%SL	From spine 6	11.9–13.5%
***A*. *tavaresi***	16–17	10–11	7–8	8–9	**11**	14–15	Long	Short	26	13	27–29	Wide	9.8–11.6%	19.6–21.7	Common	same width	39.0–42.2%	14.1–16.4	Prognath	51.2–60.0%SL	From spine 6	13.5–15.8%
***A*. *montanus***	15–17	9–10	6–8	8–9	**10–11**	13–14	LONG	Short	24–25	13	25–28	Wide	10.1–11.9%	19.3–25.7	Common	Posterior wider	41.1–48.9%	12.4–17.0%	Isognath		From spine 6	12.2–17.1%
***A*. *robustus***	17	8–9	7–8	7–8	**9–10**	13–14	LONG	Short	24	N/A	26–28	Wide	7.4–9.2%	20.0–25.2%	Robust	Same width	43.7–46.0%	13.3–15.6%	Isognath	53.3–68.7%	From spine 6	13.1–15.6%
***A*. *mattosi***	16–17	9–10	6–8	8–9	11	14	N/A	N/A	24–25	12	25–29	Wide	7.6–10.8	17.7–25.4%	Common	same width	41.5–45.2%	13.5–17.3%	Isognath	56.7–64.0	From spine 3	13.2–15.4%
***A*. *ribeirae***	16	9–10	6–7	8	11	14	long	Long	N/A	N/A	24–26	Narrow	6.6–9.1	17.7–23.1	Common	Same width	47.4–51.3%	N/A	Isognath	64.2–73.3%	N/A	16.0–16.8%
Total variation	15–17	8–11	6–8	7–10	9–11	13–15			24–26	12; 13–14	24–29		5.5–11.9%	19.3–25.7%			39.0–51.3%	12.2–17.3		51.2–73.3		11.9–17.1%

The upper half represents *Australoheros ipatinguensis*, the lower part *A*. *oblongus* and *A*. *ribeirae*. Within each part, pink cells mark absence of differences between nominal species; green boxes mark unique character states, i.e., potential autapomorphies; yellow boxes mark the complement of green boxes, i.e., potential synapomorphies, but more likely symplesiomorphies. Data include ratios (body depth per cent of SL; preorbital depth percent of head length, caudal-peduncle length per cent of SL; length of last dorsal-fin spine per cent of SL; length of last anal-fin spine per cent of SL), colour pattern (relative width of Bar 7), meristic data (dorsal-fin spines, anal-fin spines, dorsal-fin soft rays, anal-fin soft rays, ribs, pectoral-fin rays, E1 scales, distal radials supporting dorsal fin, distal radials supporting anal fin), osteology (length of epibranchial 2 epiphyses, length of epibranchial 1 arm, width of ectopterygoid), qualitative characters (shape of snout robust or not robust; relative length of lower jaw; position of most anterior scale on anal-fin base).

The tabulated proportional measurements show series of overlaps between nominal species, i.e. there were no unique character states in those characters. Two character states in *A*. *mattos*i appear to be diagnostic: the 12 anal-fin radials (pterygiophores) and the anal-fin squamation starting at the third anal-fin spine. Carmo and Triques [102] listed 12–13 radials in *A*. *mattosi*; 13 radials is a common count recorded in nominal species of *Australoheros*. They described the anal fin-squamation as ‘scales originating from 4th spine to 1^st^ ray’ which gives a range up to the eight spine. The anal-fin scale character state was also not confirmed in our specimens from the Rio São Francisco basin.

All diagnoses listed a few differences specific for a compared species. Although this was not stated, and comparisons typically were made between two or more species at a time, these multiple comparisons resulted in a unique combination of character states for each nominal taxon. Comparisons with Uruguayan species were uncontroversial (except the vertebral count made with different methods) and mostly invariant. Osteological characters were discussed above, p.00.

Ottoni and Costa [5, 6] recognised two character states in the vertical bars among their Sudeste species of *Australoheros*: presence of a forked Bar 5 in *A*. *ipatinguensis* and *A*. *paraibae*; absent in *A*. *autrani*, *A*. *saquarema*, *A*. *macacuensis*, *A*. *macaensis*, *A*. *barbosae*, *A*. *muriae*, and *A*. *robustus*. It is uncertain what forked bar 5 refers to. It may not refer to the Y mark as their figs 1, captioned as *A*. *autrani*), 9 ((captioned as *A*. *macaensis*), and 13 (labeled as *A*. *saquarema*), show the Y mark. If it refers to the vertically split ventral portion of Bar 5, none of their images shows that condition.

The type series of *Australoheros barbosae* consists of the holotype, 59.3 mm SL, and 17 paratypes, 24.9–96.2 mm SL, from the Rio Preto, a tributary of the lower Rio Paraíba do Sul. The diagnosis refers to multiple species comparisons, mainly concerning fin-ray counts differing from one or another nominal species No autapomorphic character was indicated.

*Australoheros paraibae* was based on the holotype, 50.9 mm SL, and 11 paratypes, 22.1–61.1 mm SL, all from the Rio do Peixe, a tributary of the middle Rio Paraíba do Sul. The diagnosis is very long and exclusively based on multiple species comparisons, including meristic data, and various proportions involving preorbital length, body depth, and dorsal fin-spine length, differing from one or another nominal species According to the to the description *A*. *paraibae* has a dark spot in the ‘caudal peduncle zone’, but on fig. 11 in the original description [6], there is a blotch at the base of the caudal fin and only a faint vertical bar covering the lateral extent of the caudal peduncle.

*Australoheros robustus* was described based on the holotype 74.5 mm SL), and 68 paratypes, 17.3–74.5 mm SL, from the Córrego da Areia, a tributary of the upper Rio Paraíba do Sul. The diagnosis is mainly based on multiple comparisons of meristic data distinguishing from one or another species, but also robust snout. The criterion of ‘robust’ was not specified in the description, but may reflect stoutness or sturdiness in distinction from something comparatively delicate. The image, fig. 2 [6] of the ventral aspect of the anterior part of the head of a paratype each of *A*. *Robustus* and *A*. *muriae* does not have an annotation as to what to look for.

*Australoheros macacuensis* was described based on the holotype, 67.0 mm SL, and 22 paratypes, 17.3–83.2 mm SL, all from the Rio Macacu basin, south of the Rio Paraíba do Sul, draining to the Baía de Guanabara. The diagnosis was based on multiple two-species comparison character states in combination, including a large number of meristic data, proportional measurements, colour details, and osteology, all of which shared by one or more congeneric nominal taxon. *A*. *macacuensis* was, however explicitly distinguished from congeneric species in the Sudeste as having the posterior leg of Bar 7 wider than the anterior versus both legs equal in width. The individual on fig. 8 in the original description has Bars 6, 7 (both legs), and 9 (upper) conspicuously wide. In our specimens, the pigmentation in that area is less contrasted, and indecisive concerning the state of the character. Ottoni [13]) attributed the same character state to *A*. *montanus*, and we confirmed this in MNRJ 47367 from near the type locality of *A*. *montanus*, but we observed this also in a specimen from the type locality of *A*. *muriae*, MNRJ 47291.

*Australoheros montanus* was described based on the holotype, 78.9 mm SL, and 38 paratypes, 19.4–102.5 mm SL, all from the Rio Paquequer basin, a tributary of the Rio Paraíba do Sul. The diagnosis mentions posterior arm of trunk Bar 7 wider than anterior one (shared with *A*. *macacuensis*); complete red bar on posterior margin of caudal fin; caudal peduncle length 10.1–11.9% SL; not having depression on snout; 22–24 teeth along posterior margin of ceratobranchial 5; trunk bars usually forked ventrally; anal-fin base squamation beginning at the sixth anal-fin spine; mouth isognathous. No autapomorphy was provided.

*Australoheros mattosi* was described based on the holotype, 80.4 mm SL, and 8 paratypes, 43.0–92.6 mm SL, from tributaries to the Rio São Francisco. The diagnosis made special reference to the anal-fin base squamation beginning at the third anal-fin spine (vs at the sixth spine in other autrani group species; 12 proximal anal-fin radials, isognathous mouth, and last dorsal-fin spine length 13.2–15.4% of SL. In our specimens of *A*. *oblongus*, scales on the base of the anal fin were typically present from the fifth or sixth anal-fin spine, but in at least one specimen from the São Francisco basin they were present from the fourth spine. See also remarks above. Specimens of *A*. *oblongus* from the upper Rio São Francisco may present small dark spots on the caudal fin, but were not mentioned in the original description of *A*. *mattosi*.

Carmo and Triques [102] studied the morphology of specimens identified as *Australoheros mattosi* from tributaries of the Rio das Velhas and the Rio Cipó and noted several differences from the original description in colour and body shape, but did not challenge the species identification.

*Australoheros tavaresi* was based on the holotype, MZUSP 50675A, 46.6 mm SL, and 12 paratypes, 28.8–65.8 mm SL, from the Rio Tietê and tributaries. The diagnosis is very long and detailed, chiefly comparing proportional measurements with most other species in the genus, but also stating that *A*. *tavaresi* differs from all congeneric species except *A*. *facetus* by having prognathous mouth (vs. isognathous), with reference to fig. 8 for illustration. Fig 7 and in the original description 8 show specimens in poor state of preservation, with mouth slightly open and jaws definitely isognathous. On the image of the holotype (fig. 7) it looks like it is in very bad condition, decoloured, and with trashed fins. Specimens of *Australoheros* from the Tietê examined by us had isognathous jaws. We note that the image of the holotype of *A*. *tavaresi* (fig. 7) shows only a vestige of caudal spot, and it was also relatively small in our specimens from the Tietê, whereas according to the original description [8]. *A*. *tavaresi* should have a ‘conspicuous rounded caudal-fin base spot’, also expressed as ‘spot on caudal-fin peduncle (well developed round spot)’.

*Specimens examined*. All from Brazil. MNHN 9485, 1, holotype of *Chromys oblonga*. 96.9 mm SL. le Tocantins (Province de Goyaz).—BMNH 1961.7.72–4, 3, syntypes of *Heros autochthon*, 82.1–89.2 mm SL. Brazil, Lord Stuart.—MCP 31877, 6, 49.1–67.4 mm SL; Minas Gerais: Ouro Preto: upper Rio Doce basin; Lagoa do Manso in Parque Estadual de Itacolomi, 20°22’30’’S, 43°32’30’’W; A.L.B. Magalhães, 10 Jul 2002.—NUP 15340, 9, 49.1–72.5 mm SL; Minas Gerais: Mariana: Rio Doce basin: Córrego Frazão, tributary of the Rio Piracicaba, 20°16’27"S, 43°29’18’’W. G.N. Salvador, 14 Aug 2012.—MNRJ 47418, 8, 31.4–80.8 mm SL; Rio de Janeiro: Miguel Pereira; Rio Guandu basin: confluence of the Rio Santana with the Rio Pocão, below bridge on the road Marcos da Costa, between center of Miguel Pereira and Paty; 22°29’14’’S 43°26’53’’W; M.R. Britto, C.R. Moreira and D.F. Moraes Jr., 7 Jun 2016.—LBP 14389, 3, 40.2–78.3 mm SL; São Paulo: Ubatuba: Rio Escuro at Folha Seca, 23°29’03.8"S 45°10’13.5"W; G.J.C. Silva, J.M. Henriques, J.C.P. Alves, B. Belo and R. Devidé, 7 Feb 2011.

Assigned to nominal species: [*Australoheros barbosae*]: LBP 17525. 1, 84.0 mm SL; Minas Gerais: Bom Jardin de Minas: Rio Paraíba do Sul basin: Cachoeira do Pacau, Ribeirão da Bom Jardim de Minas, 22°02’27’’S, 44°09’43’’W; J.C. Oliveira, A.L. Alves and L.R. Sato, 14 Oct 2001).—MCP 42368, 2, paratypes of *A*. *barbosae*, 38.3–58.3 mm SL; Minas Gerais: Passa Vinte: between municipios of Passa Vinte and Santa Rita da Jacutinga, tributary of the Rio Bananal, tributary of the Rio Preto, 22°’00 ’00’’S 44°00’00’’W; C. Moreira, 2 May 2007.—MNRJ 47346, 8, 35.1–82.8 mm SL; Minas Gerais: Santa Rita de Jacutinga: Rio Paraíba do Sul basin, ridge over tributary of the Córrego da Lagoa (tributary of the Rio Bananal), on the side of the gate of Fazenda do Ronco on the road between Passa Vinte and Santa Rita de Jacutinga, 22°10’2’’S 44°7’48’’W; D.F. Moraes Jr., E.B. Neuhaus and V. Brito, 12 May 2016.Topotypical specimens of *A*. *barbosae*).—MNRJ 47353, 2, 17.1–58.9 mm SL; Minas Gerais: Santa Rita de Jacutinga: Rio Paraíba do Sul basin: Córrego da Lagoa (tributary of the Rio Bananal), on road between Passa Vinte and Santa Rita de Jacutinga, 22°9’59’’S 44°7’44’’W; D.F. Moraes Jr., E.B. Neuhaus and V. Brito, 12 May 2016. Topotypical specimens of *A*. *barbosae*.—[*Australoheros paraibae*]: MCP 42367, 2, paratypes of *Australoheros paraibae*, 50.1–50.2 mm SL; Minas Gerais: Juiz de Fora: Rio Monte Verde, tributary of the Rio do Peixe, near Monte Verde, about 21°52’29.6’’S 43°31’27.5"W; W. Costa and G. Souza, 20 Aug 1991.—MCP 44194, 2, 39.6–42.6 mm SL; Rio de Janeiro: Petrópolis: Rio Paraíba do Sul basin: artificial lake with water from the Rio dos Araras, tributary of the Rio Itaipava, 22°26’03’’S 43°15’20’’W; J. F. Pezzi da Silva, 8 Apr 2003.—MNRJ 47365, 4, 52.4–64.5 mm SL; Juiz de Fora: tributary of the Rio do Peixe between Toledo and Torreões, 21°49’25’’S 43°35’17’’W; D.F. Moraes Jr., E.B. Neuhaus and V. Brito, 12 May 2016.—[*Australoheros robustus*]: MCP 42583, 2 paratypes, 39.3–41.2 mm SL; Minas Gerais: Mar de Espanha: Córrego Cachoeirinha, tributary of the Córrego da Areia near Fazenda Cachoeirinha, 12°55’00’’S 42°57’00’’W; D.F. Moraes Jr., J. H. Gomes and T. Aguiaro, 3 Mar 1990.—MNRJ 47336, 21, 20.4–69.4 mm SL; Minas Gerais: Mar de Espanha: Rio Paraíba do Sul basin: dammed tributary of the Córrego Cachoeirinha on Fazenda Jardim, 21°55’31’’S 42°58’38’’W; D.F. Moraes, Jr., E.B. Neuhaus and V. Brito, 11 May 2016.—[*Australoheros macacuensis*]: MCP 42365, 1, paratype of *A*. *macacuensis*, 55.7 mm SL; Rio de Janeiro: Cachoeiras de Macacu: tributary of Rio Guapi-Açu, 3 km from RJ-112 22°28’00.0"S 42°37’00.0"W; M. Britto, C. Moreira, F. Pupo and D. Almeida, 29 Jan 1998.—MCP 26135, 5, 23.0–57.0 mm SL; Rio de Janeiro: Cachoeirinha de Macacu: Rio Batatal, tributary of the Rio Macacu in Faraó, 22°28’00’’S 42°39’22’’W; C.S.R.F. Bizerril,10 Jul 1989.—MNRJ 47247, 2, 55.4–65.7 mm SL; Rio de Janeiro: Cachoeiras de Macacu: Rio Macacu basin: Rio Macacu at mouth of the Rio Branco, Japuíba, 22°33’19S 42°41’41’’W; P.A. Buckup, M.R. Britto, C. R. Moreira, D.F. Moraes, Jr., S.A. Santos and G.S. Araujo, 28 Apr 2016. Topotypical specimens of *A*. *macacuensis*.—[*Australoheros montanus*]: MCP 43130, juvenile, paratype of *A*. *montanus*; Rio de Janeiro: Carmo: Córrego Glória, right bank tributary of the Rio Paquequer between Córrego Pedra Branca and Córrego São José; 21°55’0"S 42°35’0’’W; D.F. Moraes Jr., H. São Tiago and E.P. Caramaschi, 2 Aug 1990.—MNRJ 47367, 5, 27.3–72.5 mm SL. Rio de Janeiro: Sapucaia: Rio Paraíba do Sul Basin: Córrego Santa Rita at Santa Rita, 22°2’3’’S 42°47’48’’W; D.F. Moraes, Jr., E.B. Neuhaus and V. Brito, 12 May 2016. Close to type locality of *A*. *montanus*.—[*Australoheros mattosi*]: LBP 6527, 1, 57.1 mm SL. Minas Gerais: Jaboticatubas: Rio São Francisco basin: tributary of Rio Cipó, 19°27’40.3’’S, 43°43’13.1’’W; R.A.S. Teixeira, G.J.C. Silva, A.T. Ferreira and J.M. Henriques, 18 Jun 2008.—MCP 42383, 1, 67.4 mm SL. Minas Gerais: Taquaruçu de Minas: small stream on road between Taquaruçu de Minas and Jabuticatubas, 19°38’24’’S 43°41’53’’W; T.P. Carvalho, F.C. Jerep and C.A. Cramer, 10 Jan 2008.—MCP 49295, 1, 40.2 mm SL; Minas Gerais: Jabuticatubas: Rio Cipó, tributary of the Rio das Velhas, 19°16’57.0"S 43°43’47.0"W; R.E. Reis, P. Lehmann and E. Pereira, 25 Jul 2005.—MCP 42942, 1, 88.3 mm SL; Minas Gerais: Jabuticatubas: stream on road between Jabuticatubas and MG-010 in direction of Serra do Cipó, 19°27’38’’S 43°43’1’’S; T.P. Carvalho, F. C. Jerep and C.A. Cramer, 10 Jan 2008.—MCP 47231, 1, 70.7 mm SL; Minas Gerais: Carmo do Paranaíba. Córrego do Bálsamo, 10 km on São Bento asphalt road, 18°53’12’’S 46°13’33’’W; R. E. Reis et al., 22 Jan 2012.—MCP 49007, 2, 43.0–76.9 mm SL. Minas Gerais: Cardeal Mota: Rio Cipó basin, arroio on road Jaboticatubas and Cardeal Mota, 19°19’9’’S 43°40’46’’; R.E. Reis, P. Lehmann and E. Pereira, 25 July 2015.—MZUSP 16176, 4, 56.9–81.4 mm SL; Minas Gerais: Esmeraldas: Rio São Francisco basin: Ribeirão Macacos, Fazenda Paraíso; D.S. Rocha, 3–8 Apr 1977.—MCP 49008, 2, 43.7–57.9 mm SL. Minas Gerais: Cardeal Mota: Rio Cipó, tributary of the Rio das Velhas, 19°16’57’’S 43°43’47’’ W; R. E. Reis, P. Lehmann and E. Pereira, 25 Jul 2015.—MZUSP 37923, 5, 58.1–86.7 mm SL; Minas Gerais: Lagoa da Prata: Rio São Francisco upstream from Três Marias dam, [19°59’51.6"S 45°31’40.3"W]; COVEVASF, Aug 1982.—[*Australoheros tavaresi*]: LBP 847, 1, 71.7 mm SL; São Paulo: Embu Guaçu; Ribeirão Santa Rita (Represa Billings), 23°55.594’S 46°53.336’W; C. Oliveira and O.T. Oyakawa, Oct 2000.—LBP 6172, 1, 64.2 mm SL; Minas Gerais: Rio Grande basin: Muzambinho: Córrego da Prata, 21°21’41.9’’S, 46°34’36’’W; G. Costa-Silva, R. Devidé, F. Roxo, L. H G. Pereira, K.T. Abe and J.M. Henriques, 16 Apr 2008. Anterior dorsal spines lost; spine deformity.—LBP 6547, 1, 58.6 mm SL; Minas Gerais: Ouro Fino: Rio da Prata, tributary of the Rio Grande, 22°11’36.6’’S 46°22’44.5’’W; R.A.S. Teixeira, G.J.C. Silva, A.T. Ferreira, J.M. Henriques, 20 Jun 2008.—MCP 17842, 1, 53.5 mm SL; Minas Gerais: Carandaí: Rio Carandaí (tributary of the Rio Grande), above Carandaí, 20°57’17.0"S 43°46’41.0"W; R.E. Reis, S.A. Schaefer, W.G. Saul, 17 Jan 1995.—MCP 20193, 1, 87.7 mm SL; São Paulo: Mogi das Cruzes: stream on road from Mogi das Cruzes to Salesópolis, about 8 km from Mogi das Cruzes, 23°34’13.0"S 46°05’54.0"W; R.E. Reis, J. Pezzi, E. Pereira and J. Montoya, 13 Jan 1997.—MCP 28715, 4, 26.8–78.6 mm SL; Minas Gerais: Abadia dos Dourados: Rio Preto on road Palmito–Abadia dos Dourados, 18°06’15.0"S 47°41’35.0"W; C. Lucena, J. Pezzi, E.H.L. Pereira and A. Cardoso, 23 Jan 2001.—MCP 37166, 2, 50.1–65.0 mm SL; Minas Gerais: Carandaí: Rio Carandaí at Haras Carandaí, 20°58’04.0"S 43°47’15.0"W; R.E. Reis, P. Lehmann and E.L. Pereira, 8 Oct 2004.—NUP 10785, 8, 39.2–113.0 mm SL; São Paulo: Salesópolis: Rio Paraitinguinha, tributary of the Rio Tietê, 23°30’25.6’’S 45°51’11.8’’W; A.P. Marceniuk, Sep 2009.

#### *Australoheros acaroides* Hensel, 1870

*Heros acaroides* Hensel, 1870: 54 [34] (type locality Bei Porto Alegre in stagnirenden Gewässern, 14 syntypes, ZMB7454, 7455, 25109, 25179; lectotype designated by Schindler et al., 2010 [11], ZMB 7455; type locality Rio Cadea.)

*Australoheros taura* Ottoni & Cheffe, 2009 [7]: 155, fig. 1 (holotype UFRJ 7574; type locality Brazil: Estado do Rio Grande do Sul: Município de Bom Jesus: near arroio Barreiro (28°48’S 50°30’W).

*Definition*. Based on the position in the phylogenetic trees based on *mt*-*cyb and mt-coI*, and minimum uncorrected *p-*distance in *mt-coI* exceeding 2% from all other species of the genus, *Australoheros acaroides* is a distinct evolutionary lineage. No morphological autapomorphy was detected. *Australoheros acaroides* is similar only to *A*. *angiru*, *A*. *kaaygua mbapoari*, *A*. *minuano A*. *ricani*, *A*. *sanguineus*, *A*. and *A*. *scitulus* in presence of a zipper band along the middle of the side, which, however, shows as a solid lateral band in sexually active specimens. It differs from *A*. *minuano* in absence of contrasting small dark spots on lower sides; from *A*. *ricani* and A. *mboapari* by row of minute scales not reaching cephalad beyond the middle of the dorsal-fin base, vs reaching to almost anterior insertion of dorsal fin; absence of black blotch on soft dorsal fin vs presence; from *Australoheros sanguineus*, by presence of regular alternating light and dark stripes on lower part of body, vs such stripes absent; in sexually active specimens this stripe pattern may be obsolete or absent.

**Fig 19:**
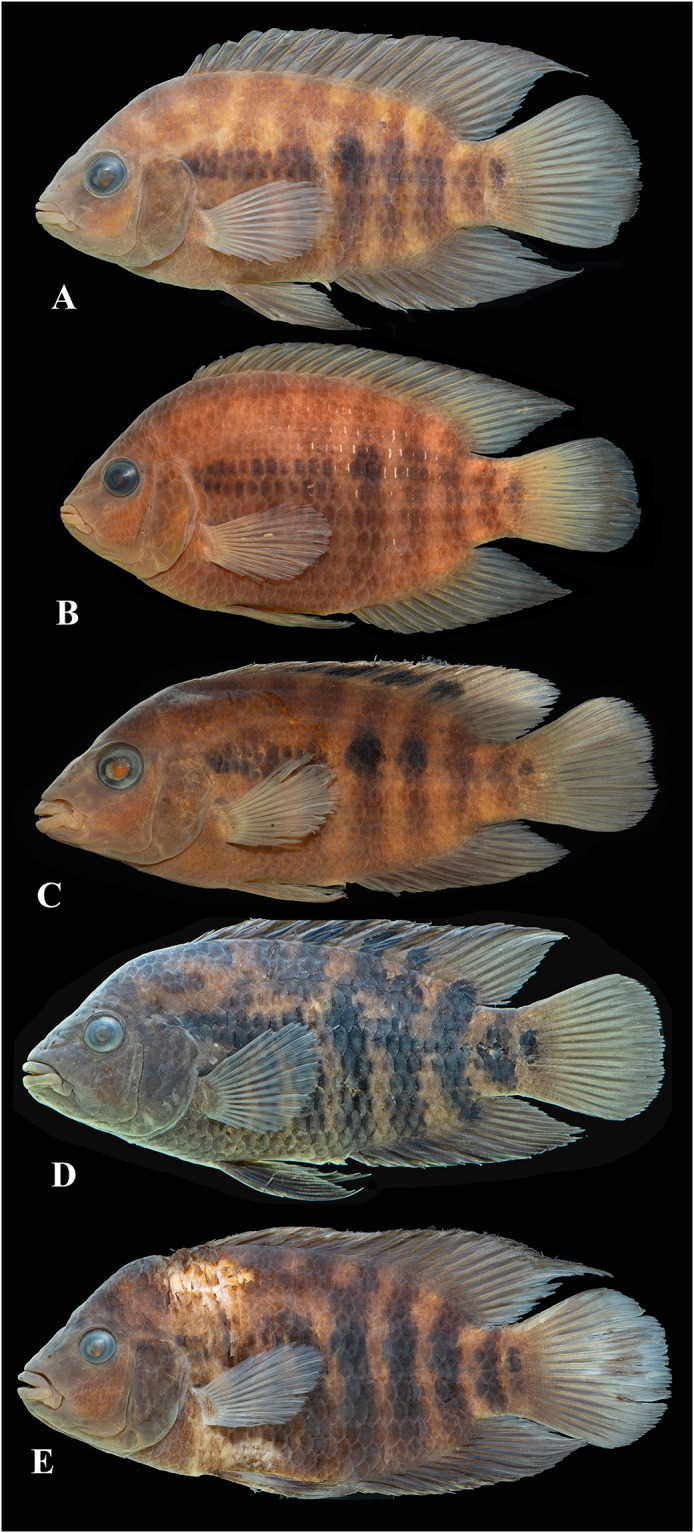
Australoheros acaroides. **A**. Adult male, 73.6 mm SL; MCP 18658; Brazil: Cachoeira do Sul: on road between Candelária and Cachoeira do Sul. **B**. adult female, 92.8 mm SL; MCP 9221; Brazil: Rio Grande do Sul; Restinga Seca: várzea of Rio Jacuí on road Santa Maria–Vera Cruz. **C**. Adult female, 58.5 mm SL; MCP 26548; Brazil: Rio Grande do Sul: Nova Palma: Rio Jacuí basin: Arroio Caemborá close to Caemborá, reversed. **D.** Adult female, 77.2 mm SL.; MCP 49694; Brazil: Rio Grande do Sul: Bom Jesus: Rio Pelotas basin: stream tributary of the Rio dos Touros ca 4 km NE of BR 285 (secondary road). **E.** Adult male, 88.5 mm SL; MCP 26772; Brazil: Rio Grande do Sul: São Francisco de Paula: Rio Tainhas basin: Arroio Ribeirão.

*Description of sample*. Meristic data is given in Tables 3–9, proportional measurements in Table 12. Refer to Fig 19 for variation in general aspect.

Sexes isomorphic except vertical and pelvic fins slightly longer in some large males than in large females. Moderately elongate to deep-bodied, laterally compressed. Predorsal contour straight ascending or with slight indentation anterior to orbits, curved anterior to dorsal fin origin. Nuchal elevation absent in both sexes. Dorsal profile curved, slightly descending to end of dorsal-fin base Caudal peduncle slightly tapering, contours straight. Prepelvic contour slightly curved, straight, or slightly concave anteriorly, convex posteriorly. Abdominal contour straight horizontal, except occasionally slightly convex in females. Anal-fin base contour straight ascending. Head short, laterally compressed. Snout short, blunt, subtriangular in lateral aspect, rounded in dorsal aspect. Mouth terminal, forward directed, close to horizontal line from lower margin of orbit in small specimens, slightly more ventral in large specimens. Lips moderately thick, lip folds interrupted or subcontinuous anteriorly. Jaws equal in anterior extension; maxilla and premaxilla not reaching to vertical from anterior margin of orbit. Orbit removed from frontal contour, in middle of head length, in upper half of head. Teeth in both jaws caniniform, slightly recurved, in outer row slender, increasing in size toward symphysis. No bicuspid teeth observed. Teeth in outer hemiseries in upper/lower jaw 7–13/101–17. Inner teeth in two rows, exceptionally one or three rows in both jaws. Gill rakers externally on first gill-arch 1–2 epibranchial, one in angle and 5–7 (7–8 in Pinares sample) ceratobranchial. Microbranchiospines present externally on 2nd to 4th gill-arch.

Dorsal-fin spines increasing in length to about 6th or 7th, from which subequal, last spine longest, soft dorsal-fin rounded in young, in adults with broad pointed tip, third, fourth or fifth soft fin-ray longest, reaching to about middle or 4/5 of caudal fin. Soft anal fin rounded in young, in adults with a short point formed by fourth soft fin-ray, reaching to about middle or 4/5 of caudal fin. Pectoral fin rounded, fourth or fifth ray longest, reaching to vertical from anal orifice or first anal-fin spine. Pelvic fin pointed, first soft ray longest, reaching to or slightly beyond anal-fin origin. Caudal fin rounded.

Scales on body finely ctenoid. Predorsal midline scales about 11–15, slightly smaller than flank scales, covered by skin, only part with exposed margin, irregularly arranged, mixed cycloid and weakly ctenoid. Chest scales ctenoid, almost size of flank scales. Cheek scales cycloid, in 4–5 series, covered by skin. Opercular scales cycloid, covered by skin. between first upper lateral line scale and dorsal-fin origin 3 large scales. Accessory lateral line scales absent from caudal fin. One large and one small scale separating last scale of upper lateral line from dorsal-fin base. Prepelvic scales anteriorly small, cycloid and without free margin, remaining scales only slightly smaller than flank scales, weakly ctenoid and with free margin. Fin scales ctenoid. Row of minute scales along dorsal fin base from spine X or XII, basal squamation gradually expanded, interradial scales in single series, from spine XII–XVI, on soft fin up to nine scales between two soft rays, scales absent from last two interradial membranes. Soft anal fin with narrow basal scale layer, up to nine scales in interradial row.

**Fig 20:**
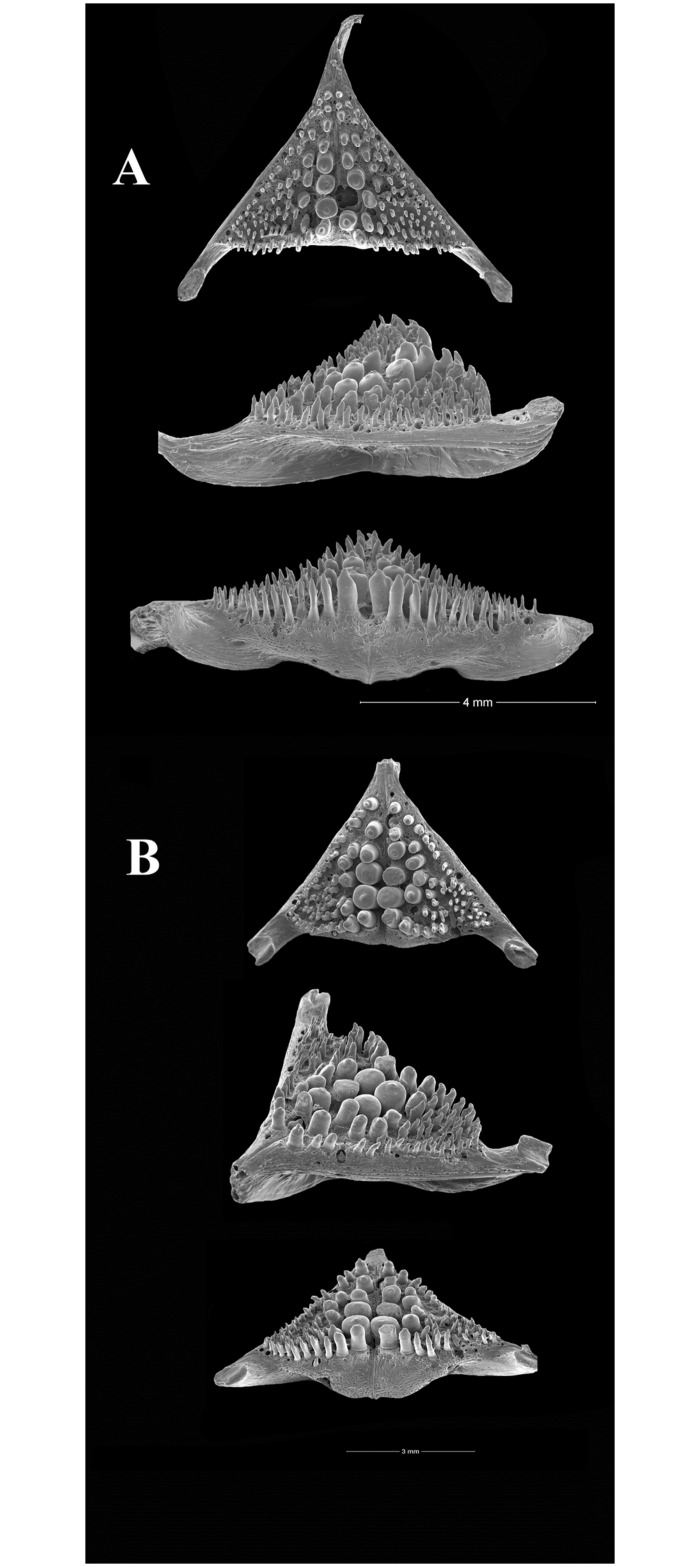
Australoheros acaroides. SEM image of the lower pharyngeal tooth plate. **A.** occlusal, approximately lateral and posterior aspect from MCP 9812, 63.3 mm SL., 63.3 mm SL; Rio Grande do Sul: Tramandai: Terra de Areia: Rio Sanga Funda on road BR 101; **B.** occlusal, latero-occlusal and caudal aspect from MCP 26548, 67.0 mm; Brazil: Rio Grande do Sul: Nova Palma: Rio Jacuí drainage: Arroio Caemborá close to Caemborá. MCP 26548, 67.0 mm.; In Tramandai specimen width 54% of length; dentigerous area width 84% of dentigerous area length. Anterior teeth subconical, slightly retrorse; 5–5 wide teeth in anteroposterior row along median suture, apex tuberculate with a minor central cusp on middle teeth, posterior teeth slightly compressed, bicuspid with antrorse distal cusp; laterad and posterolaterad on plate teeth gradually smaller and compressed; about 20 teeth in posterior row. In Caemborá specimen tooth plate relatively slender; width 76% of length; dentigerous area width 66% of dentigerous area length. Anterior teeth subconical, slightly retrorse; 8–9 wide teeth in anteroposterior row along median suture, apex tuberculate with a minor central cusp on middle teeth, posterior teeth slightly compressed, bicuspid with antrorse distal cusp; laterad and posterolaterad on plate teeth gradually smaller and compressed; about 19 teeth in posterior row. With antrorse distal cusp; laterad and posterolaterad on plate, teeth gradually smaller and compressed; about 20 teeth in posterior row.

Lower pharyngeal tooth plate examined in two specimens (Fig 20).

**Fig 21:**
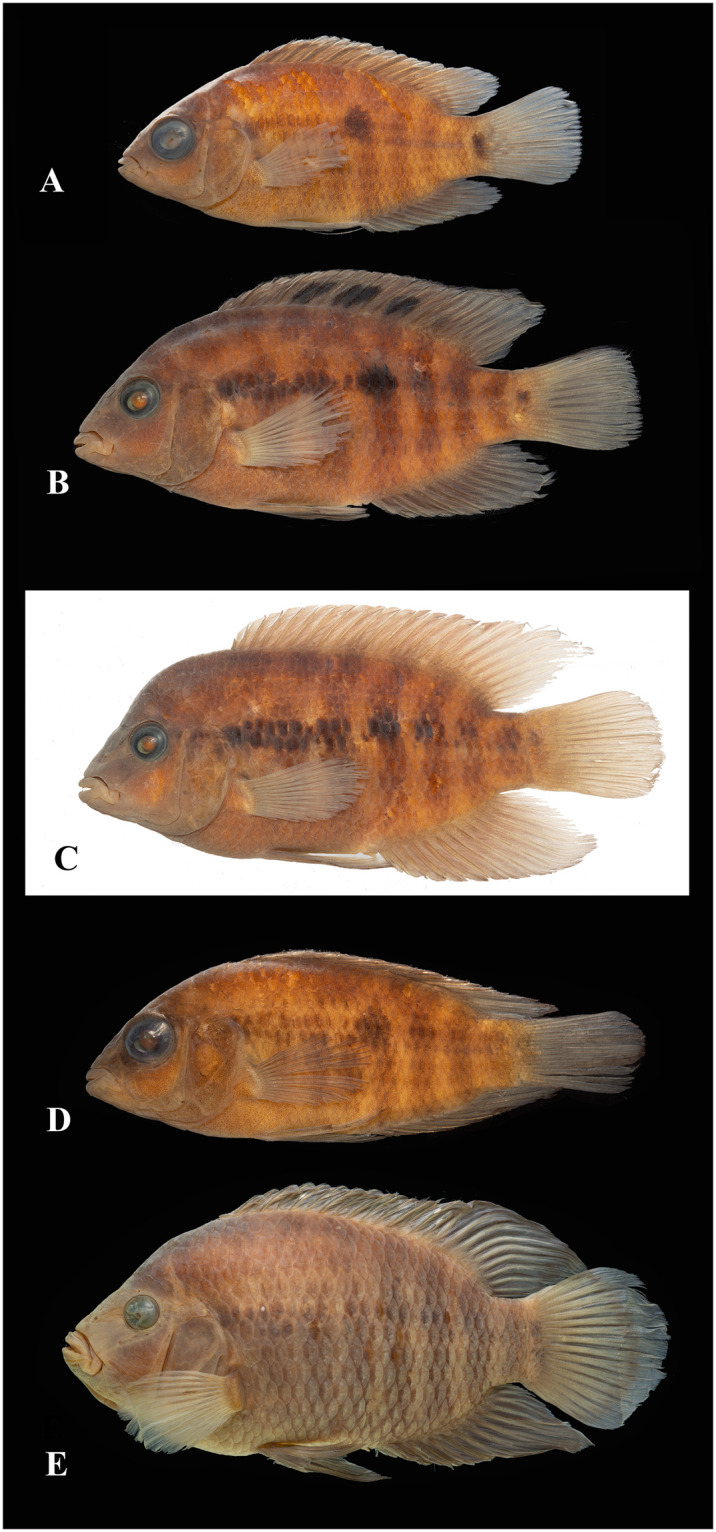
Australoheros acaroides. **A.** juvenile, 32.5 mm SL; MCP 18317 Brazil: São Francisco de Paula: Rio das Antas on road Jaquirana–Bom Jesus. **B**. Adult female, 57.7 mm SL; MCP 26548 Brazil: Rio Grande do Sul: Nova Palma: Rio Jacuí drainage: Arroio Caemborá close to Caemborá. **C**. Adult male, 66.1 mm SL; MCP 26548 Brazil: Rio Grande do Sul: Nova Palma: Rio Jacuí drainage: Arroio Caemborá close to Caemborá. **D**. Paratype of *Australoheros taura*, young, 43.6 mm SL; MCP 42363; Brazil: Rio Grande do Sul: Bom Jesus: Rio Taquari drainage: near Arroio Barreiro, tributary of the Rio das Antas. **E**. Paratype of *Australoheros taura*, adult male, 142.0 mm SL; CICMC12102; Brazil: Rio Grande do Sul: São Francisco de Paula: Rio Taquari drainage: Rio das Antas at PCH Passo do Meio dam.

*Colouration in preservative* (Figs 19 and 21). Adults, about 60 mm and larger with ground colour beige, fawn, or off-white. Bars 1(a, p) to 6 present, 2–3 scales wide; bar 6 absent below lateral band; Interbars 2–3 scales white, except Interbar 1 usually narrower than one scale width; lateral band extending from gill cleft caudad to Interbar 4 or bar 4 composed of dark blotches on scales in rows 0 and E1, forming a pattern reminding of a zipper; dark scale spots on posterior scales may extend the band to include Bar 2; Bar 5 terminating dorsally in lateral band or extending dorsally at most to dorsal-fin base. Bar 5 entire except in a few specimens from the Rio Maquiné (UNICTIO1591), in which split vertically below lateral band. Bars generally vertical, dorsal portions of bars 2–4 usually slightly caudally inclined. Dorsal portions of any combination of Bars 4, 5, and 6 commonly forming a Y mark. Dorsal portions of Bars 5 and 6 otherwise expressed as independent irregular light brownish blotches or combined in various ways. Dorsal portions of bars 7 and 8 present, expressed as short rostrad inclined bars crossing anterior dorsal-fin base and occiput, respectively: occasionally dorsal portions of bars 7 and 8 combined to form a circular marking. Vertical bars may occasionally be doubled, broken, or forming an inverted Y dorsally. Midlateral black blotch present in Bar 4 on E1 scales and parts of adjacent scales; corresponding portions of bars 2 and 3 may be slightly widened or more intensely pigmented but distinct spots absent from bars 2 and 3. Caudal blotch (Bar1p) dark brown or black, present at caudal-fin base immediately dorsal to lower lateral line, commonly expanded ventrad to form a vertical midbasal bar or oval blotch. Brown spot close to margin of scales below level of midlateral band form distinct narrow lines on abdominal side, gradually weaker and disappearing ventrad. Horizontal band formed by dark brown or black spots in E1 and 0 scales, extending to Interbar 4, continued from Bar 4 to caudal-fin base, usually more distinct anteriorly, and reduced to one scale row posteriorly. Black blotch usually present at anterior end of horizontal band and at upper base of pectoral fin. Dorsal fin translucent or pale grey, with grey, dark grey or black blotches at base, continuous with or slightly separate from bars 2 to 6 and/or 7. Colour pattern in sexually active specimens variable. In Jacuí and Taquari specimens, vertical bars 1–4, in females also dorsal-fin blotches, prominently black. In Pelotas, Tubarão and Maquiné samples, females also with abdomen, chest, and lower part of head dark brown or black. Juveniles with nearly uniform sides, vertical bars not contracted; dark spots in two rows making up lateral band, indistinct; large distinct brown or black blotch in bar 4. Caudal-fin blotch absent or distinct, dark brown and midbasal.

The sample from the Rio Tubarão included two strongly pigmented precocious gravid females with deep black spots in the dorsal fin, 37.0 and 37.5 mm SL. The sample MCP 26548 from near Nova Palma showed considerable variation in colour pattern and morphology. Two relatively small females, 47.5 and 48.0 mm SL. in MCP 26995 were the smallest specimens examined with breeding colouration expressed in dorsal-fin blotches (Fig 21). Two small males were unusual in presenting an elevated nape and steep frontal contour (Fig 21).

*Geographical distribution* (Fig 1). Laguna dos Patos basin in the Rio Jacuí drainage basin downstream of Saltos de Jacuí; Rio Taquari, with tributaries Antas and Tainhas; tributaries of the Rio Araranguá, Maquiné, Tramandaí, Tubarão, dos Sinos, and Cadeia; Rios Olimar Grande, Cebollatí, and Piratini, draining to the Laguna Mirím.

*Comments*. The colour pattern is variable within *A*. *acaroides*. There was individual variation in markings, including the shape and intensity of vertical bars and interbars, as well as in the shape and intensity of the midlateral and caudal spot, and the two sides of a specimen may be different. In summary, however, *A*. *acaroides* was similar to most other species of *Australoheros* in possession of a distinct caudal spot, 3 postabdominal vertical bars, 3 (exceptionally 4) abdominal bars below the horizontal band; a midlateral spot; a horizontal band composed of spots on two scale rows; pale brown spots from dorsal portions of bars 5 to 8 in the light area above the horizontal band; and dark spots on the base of the dorsal fin. The breeding colour was expressed in darkening—to black—of the abdomen, chest and lower head, and intensified black vertical bars, contrasting with highlighted light interbars and the light area above the anterior portion of the horizontal band. The two tooth plates examined Fig 20, were very different, although the specimens were of about the same standard length. The specimen from Tramandaí (Fig 20A) was similar to *A*. *facetus*, slender, with many small teeth, whereas the specimen from Nova Palma had a broader tooth plate with fewer teeth (Fig 20B).

*Australoheros acaroides* was represented in our material by samples from lowland tributaries to the Laguna dos Patos basin, including the Rio dos Sinos, lower Rio Jacuí, lower Rio Camaquá, Rio Piratini, Rio São Lourenço, and northern coastal rivers (Tramandaí, Maquiné, Forqueta, Tubarão) to Lagoa do Imarui at Imbatuba. Adult *Australoheros acaroides* from the Laguna Mirim were indistinguishable from those from the Patos watershed, but small specimens, and specimens from the upper Rio Camaquã lacked the horizontal stripes and had somewhat broader vertical bars.

The original description of *Heros acaroides* [34] was brief and not accompanied by an illustration. Hensel stated that among four specimens, the largest was 73 mm SL, suggesting that the main part of the and description, and the locality information (‘Bei Porto Alegre in stagnierenden Gewässern‘) was based on these four. He also described the colour pattern of ‘Eine Anzahl junger Individuen (ohne die Schwanzflosse) nicht über 20 Mm. lang.’ Under a separate heading ‘*Heros sp*.?’, he briefly described a more elongate specimen from the Rio Cadea, similar to the *H*. *acaroides* in colour, but more elongate and in a poor state of preservation.

*Heros acaroides* was sunk into the synonymy of *Australoheros facetus* by Steindachner [35]. Schindler et al. [11] provided a re-description of *A*. *acaroides*, purporting to revalidate the species. In so doing, they did not compare with specimens of *A*. *facetus*, but relied on Říčan and Kullander [3, 4] for data. Essentially, they restricted *A*. *acaroides* to the Laguna dos Patos basin, with the diagnosis*’…* differs from all the species of the *A*. *forquilha*, *A*. *scitulus* and *A*. *kaaygua* groups by having a well developed caudal-fin base spot (*vs*. spot absent or weakly developed as a pigmented narrow bar); from *A*. *minuano* and all the species of the *A*. *facetus* group by having well developed longitudinal stripe (*vs*. weakly developed) and three abdominal bars versus four (except in *A*. *guarani*); from all the species from the *A*. *kaaygua* group by having more pectoral rays (14 *vs*. 12–13); from *A*. *facetus* by having a [sic] isognathous jaw (*vs*. prognathous) and from *A*. *facetus* and *A*. *guarani* by having modally 5 cheek scales rows [sic] (vs. three in *A*. *facetus* and four in *A*. *guarani*).’ Our observations show that in *A*. *acaroides* the expression of Bar 1p is variable; the count of 14 pectoral fin-rays is rare and 13 rays dominant as in congeneric species (Table 8), all species of *Australoheros* are isognath, and that the presence of four abdominal bars is the common state in the genus a; fifth bar occurs at low frequency in all coastal species.

Schindler et al. [11] reported 14 syntypes of *A*. *acaroides*: ZMB 7455 (2, 34.4–71.2 mm SL, from Rio Cadea), ZMB 7454 (3, 16.3–18.2 mm SL from Porto Alegre); ZMB 25109 (2, 34.2–62.6 mm SL, Rio Cadea), ZMB 25179, 7[sic, should be 8] (<18 mm SL, no locality given [Porto Alegre]). Schindler et al. [11] fixed ‘the larger specimen’ in ZMB 7455 as lectotype of *Heros acaroides*. Their fig. 1 shows a specimen with the caption ‘Lectotype of *Australoheros acaroides*, ZMB 7455’. They reported that on the jars containing ZMB 7455 and 25109, the locality is stated as Rio ‘Cadea’, and accepted Rio Cadea as type locality. Apparently, ZMB 25109 was at some time in its curatorial history split from ZMB 7455 and together they represent the four specimens mentioned by Hensel as coming from stagnant water at Porto Alegre; and ZMB 25179 was split from ZMB 7454, and represents the number of young specimens mentioned by Hensel. Whether the specimens came from Porto Alegre or the Rio Cadeia, may not be of critical concern, as the Rio Cadea [= Rio Cadeia] is located less than 50 km north of Porto Alegre, and runs to the Rio Caí, with mouth in the Rio Jacuí near Porto Alegre. There are no recent collections of *Australoheros* from the Rio Cadeia. Hensel lived in São Leopoldo, north of Porto Alegre, 1863–1866 [103], and his collections were apparently shipped back to Germany in at least two shipments up to 1868 [103–105]. Paepke and Schindler [106] listed the syntypes, but some objects with different catalogue numbers: The paralectotype in ZMB 755 is now ZMB 34506; ZMB 25109 is now ZMB 34502; ZMB 25179 is now ZMB 34501 and listed with the correct number of specimens (8).

*Australoheros taura* was described based on the holotype, 65.4 mm SL, from Bom Jesus, Rio Grande do Sul, figured by Ottoni and Cheffe, fig. 1 [7], and 34 paratypes, 20.6–124 mm SL from the Rio das Antas and tributaries. We examined five paratypes (MCP 4263, Fig 21D; CICMC 12102, Fig 21E) and several non-types specimens of *Australoheros* from the Rio das Antas and Rio Tainhas. The five CIMC paratypes, 73.0–124.5 mm SL, could not be located but a specimen from the same locality, CICM 12203, 86.8 mm SL was examined.

Ottoni and Cheffe [7] distinguished *A*. *taura* by a set of characters in combination, including vertebral count, various proportions, projecting upper jaw, live colours, and melanophore colour pattern. The holotype, as figured by Ottoni and Cheffe, fig. 1 [7]) displays the characteristic zipper-like lateral band present in *A*. *acaroides* and this pattern was also present, but indistinct in the small MCP paratypes and the CICM 12203 specimen. The caudal blotch, said to be narrow in the type series, varied from almost obsolete (as shown on Ottoni and Cheffe’s fig. 1 [7], and Fig 21 herein) to black, but expressed only as a dark spot on the upper lobe, and a fainter spot on the ventral lobe. The jaws were isognathous. The *mt-coI* sequences of tissue samples from the Rio das Antas, and the Rio Tainhas were nested in a clade also including specimens identified as *A*. *acaroides* from the Rio Jacuí and Rio Taquari, and other drainages in Rio Grande do Sul. Based on the genetic and morphological data, *A*. *taura* is a synonym of *A*. *acaroides*. It seems that *A*. *taura* was proposed because *A*. *acaroides* was at the time not recognised as a valid species, and the comparative material included almost only *A*. *ribeirae* and data from literature, but no specimens from the rest of the Rio Jacuí drainage.

No autapomorphy was mentioned by Ottoni & Cheffe [7] for *A*. *taura*. The diagnosis was based largely on measurement ratios without compensation for size allometry. Outstanding characters such as ‘lower jaw slightly in front of the upper jaw shorter than upper one (vs lower jaw projecting in front of the upper jaw (not verifiable on the photo, fig. 1, but lengths shown in their table), 6–7 epibranchial plus 15–16 ceratobranchial gill-rakers, and 22 caudal-fin rays were not commented on. In species of *Australoheros*, jaws are isognathous in anterior extension, but the lower jaw (distance dentary symphysis to retroarticular) may be slightly shorter than the upper jaw (distance premaxilla to posterior tip of maxilla).

The paratypes and topotypical specimens examined all had 7 or 8 ceratobranchial gill-rakers. (See Material and methods for comments on gill-raker counts). Except for a few small-sized species with 14, all cichlid species have 16 principal caudal-fin rays, including the examined paratypes of *A*. *taura*.

The image of the holotype shows a specimen with a broad horizontal stripe containing the midlateral spot, and faint vertical bars, but no further markings. The most conspicuous character on the photo is the very large eye, suggesting a juvenile, although the specimen is said to be 65.4 mm SL. The MCP paratypes also had proportionally very large eye and tapering body. We suggest that this condition reflects under-nourishment in aquaria or fishponds. The juvenile MCP paratypes did not possess a caudal blotch; it was present in all other specimens of *A*. *acaroides* from the upper Rio Taquari drainage. All specimens possessed a midlateral blotch and the lateral band was variously expressed but present in most specimens.

The description of *A*. *sanguineus* [12] (included a live colour photo labeled *A*. *sanguineus*. According to the legend the specimen was not preserved. The colour pattern shown is similar to that of both *A*. *sanguineus* and *A*. *acaroides*. In the same paper another photo (fig. 3) of a living fish is labeled as an adult *A*. *acaroides* and with Morevy Cheffe as photographer, but it is actually the same specimen as illustrated by Calviño [62]: fig. 3), only reversed and with darker colour, and according to Calviño) showing a juvenile topotype, 55 mm SL, of *A*. *facetus*. The latter identification seems more reliable as the photo was taken by Calviño of a specimen he himself had collected from the type locality: unless also Calviño had mixed up his photos. Calviño’s fig. 3 and Ottoni’s fig. 3 undoubtedly show the same specimen with the same posture and with the same background, but not necessarily the same exposure as the light spot close to the upper middle portion of the image is above the fish in Calviño’s photograph, and partly covered by the dorsal fin in Ottoni’s version, which is also reversed and darker.

*Material examined*. All from Brazil unless otherwise stated.

ZMB 7455, lectotype of *Heros acaroides*, 72.5 mm SL. Brazil, Rio Grande do Sul, Rio Cadea [= Rio Cadeia. R. Hensel, [1863–1866].

ZMB 7455, paralectotype of *Heros acaroides*, 1, 35.0 mm SL. Same data as lectotype.

ZMB 25109, paralectotypes of *Heros acaroides*, 2, about 40.0–63.0 mm SL. Same data as lectotype.

ZMB 7454, paralectotypes of *Heros acaroides*, 3 juveniles, not measured. Brazil, Rio Grande do Sul, Porto Alegre. R. Hensel, [1863–1866].

ZMB 25179, paralectotypes of *Heros acaroides*, 8 juveniles, not measured. Same data as ZMB 7454.—Rio Grande do Sul: Rio Jacuí basin: Rio Soturno basin: LBP 14575, 1, 77.1 mm SL; LBP 14581, 1 (of 2), 25.5 mm SL: Júlio de Castilhos: stream, without name, 29°19’07.6"S 53°37’59.2"W; F. Roxo et al., 26 Sep 2011.—LBP 14564, 2, 30.0–42.3 mm SL; Agudo: stream without name, 29°36’03.3’’S 53°16’50.4’’W; F. Roxo et al., 26 Sep 2011.—MCP 22729, 11, 22.7–94.8 mm SL; Nova Palma: Arroio do Tigre, about 3 km S of Nova Palma, 29°29’25.0"S 53°28’45.0"W; R. E. Reis, E.H.L. Pereira and V.A. Bertaco, 1 Apr 1999.—MCP 22706, 3, 21.4–80.6 mm SL; Júlio de Castilhos: Arroio Felicio, about 10 km SSE of Júlio de Castilhos, 29°19’6’’S 53°37′59’’W; R. E. Reis, E.H.L. Pereira and V.A. Bertaco, 1 Apr 1999.–MCP 22726, 6, 28.8–50.9 mm SL; Faxinal do Soturno: stream tributary of the Rio Soturno about 4 km NNW of Soturno, 29°32’55’’S 53°27’53’’W; R.E. Reis, E.H.L. Pereira and V.A. Bertaco, 1 Apr 1999.—MCP 50425, 3, 27.5–99.7 mm SL; Júlio de Castilhos: Arroio Felicio, on road between Parque das Esculturas and Júlio de Castilhos, 29°19’6.400’’S 53°37’57.800’’W; T Carvalho, R. Angrizani and J. Chuctaya, 11 Oct 2016.—MCP 50424, 1, 95.6 mm SL; Faxinal do Soturno: Rio Soturno at bridge close to road between Nova Roma and Faxinal do Soturno, 29°32’6.900’’S 53°28’50.200’’W; T. Carvalho, R. Angrizani and J. Chuctaya, 11 Oct 2016.–Lower Rio Jacuí basin: MCP 21486, 1, 54.4 mm SL; Pinhal Grande: Rio Ferreira between Pinhal Grande and Itauba, 29°16’37’’S 53°14’53’’W; R.E. Reis, J.F. Pezzi and E.H.L. Pereira, 11 Oct 1998.—MCP 21272, 4, 32.7–51.4 mm SL; Ibarama: stream tributary of the Arroio Caidinho, 29°22’38’’S 53°05’56’’W; R.E. Reis, J.F. Pezzi and V.A. Bertaco, 23 Aug 1998.—MCP 23753, 6, 32.3–53.5 mm SL; Agudo: Lageado do Gringo about 2 km from UHE Dona Francisca, 29°26’49.0"S 53°15’36.0"W; R.E. Reis, J.F. Pezzi and V.A. Bertaco, 11 Jul 1999.––MCP 25465, 2, 58.8–76.4 mm SL; Agudo: Lageado do Gringo, about 2km from UHE Dona Francisca, 29°26’49’’S 53°15’36’’W; R.E. Reis, J.F. Pezzi, V.A. Bertaco and E.H.L Pereira, 10 Jan 2000.—MCP 25720, 1, 60.8 mm SL; Agudo; Lageado do Gringo about 2 km from UHE Dona Francisca, 29°26’49’’S 53°15’36’’W; R.E. Reis, J.F. Pezzi and V.A. Bertaco, 8 Apr 2000.—MCP 26224, 2, 54.5–56.4 mm SL; Ibarama: stream tributary of the Rio Jacuí at about 3 km from ferry across Rio Jacuí in direction to Dona Francisca dam, 29°25’35’’S 53°14’44’’W; V.A. Bertaco, E. H.L. Pereira and A.R. Cardoso, 25 Aug 2000.––Arroio Corupá to Rio Jacui: MCP 26995, 5, 47.5–62.0 mm SL; Agudo: Arroio Corupá on road between Agudo and UHE Dona Francisca, 29°33’54’’S 53°17’9’’W; R.E. Reis, V.A. Bertaco and E.H.L. Pereira, 1 Feb 2001.—MCP 28984, 1, 68.6 mm SL; Agudo: Arroio Corupá, 29°33’54’’S 53°17’9’’W; R.E. Reis, E.H.L Pereira and V.A. Bertaco, 15 Nov 2001.—Rio Vacacai Mirim to Rio Jacuí: MCP 26548, 13, 33.7–87.2 mm SL; Nova Palma: Arroio Caemborá close to Caemborá, 29°50’’28’S 53°17’50W; V.A. Bertaco, A. Cardoso, C. Kaefer and J. Silva, 11 Nov 2000.—Rio Jacuizinho: Espumoso, 28°58’02.9’’S 52°47’20.3’’W: UFRGS 22014, 1, 73.6 mm SL; K.O. Bonato and J. Ferrer, 22 Feb 2013.—UFRGS 22030, 1, 19.5 mm SL; K. Bonato N. Bertier and A. Hirschmann, 19 Dec 2012.—UFRGS 22031, 1, 22.6 mm SL; K.O. Bonato, A. Hirschmann, A. Hartmann and A. Langoni, 20 Apr 2013. [determination tentative].—MCP 9221, 5, 46.6–92.8 mmSL; Restinga Seca: várzea of Rio Jacuí on road Santa Maria–Vera Cruz, 29°48’0"S, 53°17’0"W; C.A.S. Lucena, L.R. Malabarba and R.E. Reis, 16 Sep 1983.—Rio Ingaí to Rio Jacuí: MCP2 1520, 2, 57.5–82.0 mm SL; Rio Grande do Sul: Fortaleza dos Valos: Rio Jacuí basin, stream tributary of the Rio Ingaí about 300 m SW of Fazenda Colorados, 28°54’29’’S 53°17’8’’W; R.E. Reis, J.F. Pezzi da Silva and E.H.L. Pereira, 11 Oct 1998.—Lageado Pelado to Rio Jacuí: UFRGS 18223, 4, 97.7–114.4 mm SL; Salto de Jacuí: Lageado Pelado, 29°03’00.7"S 53°15’48.6"W; A.P. Dufech and J.L Santos, 4 Nov 2013.—Rio Vacacaí basin to Rio Jacui: MCP 16292, 3;90.7–101.3 mm SL Caçapava do Sul: Arroio Pessegueiro in Passo do Megatério, 30°28’0"S, 53°36’59"W; A. Ramires, 15 Apr 1993.—Rio Pardo basin to Rio Jacuí: MCP 18658, 1, Cachoeira do Sul: on road between Candelária and Cachoeira do Sul, 29°43’0"S, 52°45’0"W; L.R. Malabarba, J.R. Burns, J.F. Pezzi, 21 Jan 1996.—Rio Grande do Sul: Rio Taquarí basin: CIMC 12203. 1, paratype of *Australoheros taura*, 86.8 mm SL; Bom Jesus: Arroio Barreiro, tributary of the Rio das Antas [probably 28°48’S 50°30’W]; M. Cheffe and L. Rosa, 5 Feb 2005.—CIMC 12102, 1, 142.0 mm SL; São Francisco de Paula: Rio das Antas at PCH Passo do Meio dam, [Approx. 28°48’S 50°36’W];M. Cheffe, L. Rosa and R. Baltar, 12 Nov 2004.—MCP 18317, 6 (1, 73.2 mm SL; 5, 15.4–32.5 mm SL)—MCP 20504, 2, 27.5–32.5 mm SL; São Francisco de Paula: Rio das Antas on road Jaquirana–Bom Jesus, 25°48’0’’S, 50°26’0’’W; A. Ramires, 18 Nov 1995.—MCP 26772, 1, 88.5 mm SL. São Francisco de Paula: Rio Tainhas basin: Arroio Ribeirão, 29°12’0’’S, 50°21’0’’W; W. Bruschi, Jr. and L. Daros, 19 Dec 2000.—MCP 32400, 1, 26.4 mm SL; Bom Jesus: Arroio Barreiro about 150 m from its mouth into the Rio das Antas, 28°47’50’’S, 50°25’36’’W; A.R. Cardoso and V.A. Bertaco, 15 Feb 2003.—MCP 32419, 2, 16.7–23.3 mm SL. São Francisco de Paula: Rio Tainhas basin: Rio Tainhas between municipalities of São Francisco de Paula and Tainhas. A.R. Cardoso and V.A. Bertaco, 14 Feb 2003. 28°52’06’’S, 50°27’33’’W.—MCP 42363, 4, paratypes of *Australoheros taura*, 36.0–43.6 mm SL. Bom Jesus: Rio Taquari basin: near Arroio Barreiro, tributary of the Rio das Antas. M. Cheffe and L. Rosa, 6 Apr 2004. 28°48’S, 50°30’W.—MCP 41156, 4, 41.8–68.2 mm SL: Nova Roma do Sul: Rio Taquari basin: Arroio Cansan 2, Rio das Antas basin. J.C. Latini, V.A. Capatti and S. Rodrigues, May 2006. 28°59’59’’S, 51°22’2’’W.—MCP 48464, 1, 25.6 mm SL. Cambará do Sul: Rio Tainhas basin: Parque Estadual Tainhas. P. Lehmann, 29°5’5.200’’S 50°21’57.7’’W.—MCP 48666, 10, 22.4–58.6 mm SL. São Francisco de Paula: Rio Tainhas basin: Arroio Ribeirão on RS-453 (Ruta do sol), 29°13’58’’S, 50°22’29’’W; J. Silva, D. Araújo, R. Angrizani, 11 Mar 2015.—MCP 48671, 1, 41.0 mm SL; Jaquirana: Rio das Antas basin: Rio das Antas at mouth of Arroio Moraes on road to Bom Jesus, 28°47’54’’S, 50°25’44’’W; J. Silva, D. Araújo and R. Angrizani, 11 Mar 2015.—MCP 48687. 14, 22.5–28.6 mm SL; Jaquirana: Rio das Antas basin: Rio das Antas at mouth of the Rio Camisas on road Jaquirana-São Francisco de Paula. J. Silva, D. Araújo and R. Angrizani, 11 Mar 2015. 28°50’30’’S, 50°18’0’’W.—UFRGS 9256. 2, 31.9–32.9 mm SL. São José dos Ausentes: Rio das Antas after fish & pay pond. 28°47’08.0"S 49°58’55.0"W; collector not recorded, 17 March 2001.—UFRGS 9261. 1, 41.4 mm SL. São José dos Ausentes: Rio das Antas, 28°47’07.0"S 49°58’55.0"W; J. Anza, J. Bastos, D. Gelain, T. Dias and T. Hasper, 30 Jul 2001. 28°47’07.0"S 49°58’55.0"W.—UFRGS 21917, 1, 52.8 mm SL. São José dos Ausentes: Arroio São Gonçalo,—tributary of the Rio das Antas, 28°52’09.0"S 50°01’14.0"W; L.R. Malabarba, J. Ferrer, T. Carvalho, R. Angrizani andand E. Wendt, 29 Apr 2016.—Rio Grande do Sul: Rio Maquiné basin: UNICTIO 1358, 2, 40.5–6.7 mm SL; Maquiné: affluent of Rio Maquiné, 29°39ʹ57.3’’S 50°11ʹ14.4’’W; M. Ibere, P. Brites, C. Bartzos, 19 Jan 2013.—UNICTIO 1479, 1, 56.6 mm SL; Arroio Forqueta, 29°32’18.4’’S 50°14’45.6’’W; P. Lehmann and students, 18 Dec 2013.—UNICTIO 1580, 2, 61.9–62.0 mm SL; Maquiné: tributary of Rio Forqueta, 29°32ʹ18.6’’S 50°14’45.4’’W; P. Lehmann, 22 Jan 2014.—UNICTIO 1591. 6, 21.0–91.1 mm SL; Maquiné: left bank tributary of Rio Maquiné, 29°32’18.1’’S, 50°14’45.6’’W; P. Lehmann et al., 14 Mar 2014.—UNICTIO 1627. 10, 37.4–86.5 mm SL. Arroio Carvão, tributary of the Arroio Forqueta, 29°32’17’’S 50°14’44.1’’W; P. Lehmann and students, 15 Mar 2014.—UNICTIO 1670, 2, 50.5–80.0 mm SL. Maquiné: Arroio Carvão; P. Lehmann, L. Schaumbach et al., 4 Apr 2014. 29°32’18.3’’S, 50°13’45.7’’W.—Rio Grande do Sul: Rio Piratini basin UNICTIO 641, 1, 68.3 mm SL; Pedro Osório: Arroio Passo dos Negros, 31°48’41.4’’S, 52°48’23.2’’W; P. Brites, C. Bartzos and E. Cavalet, 6 Sep 2012.—UNICTIO 414. 1, 93.0 mm.: Pedro Osório, 31°53ʹ17.4’’S, 52°43’18’’W. P. Brites, C. Bartzos and P. Peixoto, 22 Apr 2012.—UNICTIO 461, 6, 31.5–57.4 mm SL. Pedro Osório, 32°00’41’’S 52°55’13.4″W; 22 Apr 2012, P. Brites, C. Bartzos and P. Peixoto, 22 Apr 2012.—UNICTIO 555, 11, 26.2–39.8 mm SL; Fazenda Pedro Osório, 31°50’40.3’’S 52°51’58’’W; P. Brites, 30 Apr 2002.—UNICTIO 696, 4, 27.0–40.8 mm SL; Cerrito: Rio Piratini, 31°43’09.6’’S 52°53’59.9’’W; P. Brites, C. Bartzos, P. Peixoto and E. Cavalet, 7 Jun 2012.

—Rio Grande do Sul: Rio dos Sinos basin: UNICTIO 1251. 2, 52.1–77.2 mm SL; Igrejinha: Arroio Solitario. U. Schultz, 6 Apr 2013. 29°33’46.60’’S, 50°60’12.28’’W.—UNICTIO 1343, 1, 74.0 mm SL; Igrejinha: Arroio Solitario, 29°33’46.60’S 50°60’12.28″W; U. Schultz, 6 Apr 2013.—MCP 17628, 3, 107.3 mm SL; Caraá: Rio dos Sinos, bridge 7 km north of Caraá, road to Fundo Quente, 29°47’0"S, 50°19’0"W; L.R. Malabarba, P.A. Buckup, A. Cardoso and G. Guazeli, 12 Jan 1995.—

Rio Grande do Sul: Rio Caí basin: MCP 11558, 38, 17.8–49.5 mm SL (3 measured and counted, 44.1–49.5 mm SL); Gramado: Arroio Linha Bonita, 29°22’00.0"S, 50°52’00.0"W; L. Mardini, 17 Jun 1987.—Rio Grande do Sul: Tramandai: MCP 9812, 4, 1 measured, 63.3 mm SL, Tramandai: Terra de Areia: Rio Sanga Funda on road BR 101, 29°37’00.0"S 50°07’00.0"W, 27 Jan1983, C.A.S. Lucena and Z.M. Lucena.—Rio Grande do Sul: Rio Camaquã basin: MCP 25787, 19, 63.8–66.0 mm SL; Caçapava do Sul: Arroio do Banhado on BR-153 to Minas do Camaquã, about 8 km NW of Minas do Camaquã, 30°51’48"S 53°29’50"W; C.A.S. Lucena, J.F.Pezzi and V.A. Bertaco, 27 Apr 2000.—

Rio Grande do Sul: Lagoa Mirim: MCP 43212, 1, SantaVitória do Palmar; Lagoa Mirim, coordinates for Lagoa Mirim near Santa Vitória do Palmar, 33°30’20.8"S 53°26’34.8"W; Mario V. Candine, 1 Dec 2004.—MCP 43213, 1, Santa Vitória do Palmar: Lagoa Mangueira, coordinates for Lagoa Mirim near Vitória do Palmar, 33°30’20.8"S 53°26’34.8"W; Mario V. Candine, 1 Oct 2004.—

Santa Catarina: Rio Araranguá basin: MCP 50385, 4, 64.9–81.4 mm SL, 64.9–81.4 mm SL; Timbé do Sul: Rio Amola Faça, tributary of Rio Itoupava, 28°50’23″S 4948’0″W; G. Deprá, J. Dourdall and G. Aguilera, 18 Dec 2015.—UFRGS 19912, 3, 58.6–100.4 mm SL; Santa Catarina: Siderópolis: Rio Jordão upstream of Cachoeira Bianchini, 28°35’11.7"S 49°31’24.6"W; R. Angrizani, L.R Malabarba, M.C. Malabarba, 21 Feb 2015.—UFRGS 12548, 1, 74.3 mm SL; Santa Catarina: Nova Veneza: Jordão Alto [São Bento Alto]: Rio São Bento, tributary of the Rio Araranguá, 28°39’29.1"S 49°32’35.7"W; Ferrer, Thomas, Vogel and Weiss, 29 Mar 210. Lagoa Imaruí: MCP 8426, 1, 68.7 mm SL. Imbituba: Lagoa do Imarui, 28°16’00.0"S 48°43’00.0"W; C.R. Poli and L.

Borsato, 22 Jan 1971. Rio Tubarão basin: MCP 11057. 1, 56.1 mm SL; Tubarão: Rio Tubarão basin. Canal linking the Rio Tubãro with Lagoa Santa Marta, including pools and lateral canals, 28°31’0"S, 48°49’0"W; J.J. Bertoletti and E.P. Lerner, 18 Dec 1986.—MCP 13587. 7, 35.7–107.0 mm SL. Santa Catarina: Tubarão: Rio Tubarão basin. Rio Tubarão and lateral canals near Campo Verde, 28°31’0’’S, 48°49’W; MCP team, 28 Nov 1986.—Rio Grande do Sul: Rio Pelotas basin: MCP 49694, 21, 15.1–77.2 mm SL; Bom Jesus: Arroio tributary of Rio dos Touros ca 4 km NE of BR 285 (secondary road), 28°41’06.0"S 50°12’51.0"W; José Silva, 12 Feb 2016.—MCP 48688, 7 18.9–56.4 mm SL; MCP 48993, 42, 41.1–45.5mm SL; Bom Jesus: Rio dos Touros, on road Rondinha-Pascoal, next to Usina Hidreletrica, 28°38’44.0"S 50°17’06.0"W; José Silva, 11 Mar 2015.—MCP 53174, 3, 64.4 mm SL; Bom Jesus: Rio dos Touros, below barrage on road Rondinha to São Joaquim, 28°38’45.0"S 50°17’08.0"W; R. E. Reis, E. H.L. Pereira, P. Fagundes, 5 Jan 2018.—UFRGS 21893, 1, 81.0 mm SL; São José dos Ausentes, Rio da Divisa, tributary of Rio Pelotas, 28°40ʹ16.8ʺS 49°57ʹ56.2ʺW; L.R. Malabarba, J. Ferrer, T.P Carvalho, R. Angrizani, 29 Apr 2016.—MCP 14387, 16, 46.7–94.1 mm SL; Bom Jesus: tributary of Rio dos Touros, tributary of Rio Pelotas on road Silveira-Rondinha; 28°39’18.0"S 50°18’25.0"W; C. Lucena, P. Azevedo and E.H.L. Pereira, 14 Jan 1989.—Santa Catarina: Rio Pelotas basin: MCP 14359, 1, 72.1 mm SL; São Joaquim: Arroio da Velha Sílvia on road Bom Jesus to São Joaquim, tributary of Rio São Mateus, tributary of Rio Pelotas, 28°20’0"S 49°57’0’W. C.A.S. Lucena, P. Azevedo and E.H.L. Pereira, 15 Jan 1989.—MCP 22439, 2, 32.2–68.9 mm SL; Bom Jardim da Serra: Rio Cachoeira on road from Bom Jardim da Serra to Santa Barbara, ca. 5.1 km N of Bom Jardim da Serra, 28°18’26"S 49°37’02"W; R.E. Reis, A.R. Cardoso, P.A. Buckup and F. Melo, 18 Dec 1988.—MCP 22438, 5, 35.7–47.0 mm SL; Bom Jardim da Serra: Rio Capivaras on road from Silveira to Bom Jardim da Serra, 28°24’21"S 49°38’26"W; R.E. Reis, A.R. Cardoso, P.A. Buckup, F. Melo, 17 Dec 1998.—Uruguay: Rio Cebollatí basin: NRM 36495, 2, 45.2–63.5 mm SL; NRM 36774,2; 53.2–56 mm SL; NRM 36848, 330.6–35.8 mm; Arroyo Local at margin of Valentines, 33°15’11"S 55°05’59"W; F. Cantera and S. Kullander, 20 Nov 1997.—NRM 39527,13,42.9–72 mm SL; NRM 37035, 1 C&S; 37037,1C&S; 37036,1, c&s; Treinta y Tres: Arroyo Local at Valentines, 33°16’0"S 55°40"W; F. Cantera et al., 17 Jan 1997.—NRM 61491, 7; Rocha: Velázquez: Arroyo de la India Muerta at Paso Santiago, 8 km from Velázquez, 2 km from km 77 on Ruta 15m 34°05’52"S 54°15’53"W; S. Kullander et al., Mar 2011.—NRM 43943, 2, Treinta y Tres: Valentines, 33°16’00"S 55°04’00"W; F. Cantera, Dec 1997.—Uruguay: Rio Olimar Grande basin: NRM 39546, 2 juvs, Treinta y Tres: Arroyo Averías at Las Pavas, 30 km from Valentines and 8 km from Ruta Nacional, 19 33°12"S 54°43’59.9"W; F. Cantera, 24 Nov 1996.—NRM 61383, 3, juvs; NRM; 70180, 1, juv.; 70181,1, 28.4 mm SL; Treinta y Tres: Vergara; Tributary of the Arroyo del Parao under bridge, Ruta 18, W of Vergara, 32°55’12"S 53°55’18.1"W; S. Kullander et al., 1 Mar 2011.—NRM 61427,1, juv.; Rocha: Tributary of the Arroyo de las Averías at Estancia El Ytay close to junction with Arroyo de las Pavas, 33°14’28"S 54°50’44"W; S. Kullander et al., 4 Mar 2011.

#### *Australoheros minuano Říčan* & Kullander, 2008

*Australoheros minuano* Říčan & Kullander, 2008 [4]: 35, fig. 15; holotype MCP 12710; Brazil, Rio Grande do Sul, Arroyo Canoin, Rio Uruguay basin, road from Pirapo to São Nicolau).

*Definition*. Based on the position in the phylogenetic trees based on *mt*-*cyb and mt-coI*, and minimum uncorrected *p*-distance in *mt-coI* exceeding 2% from all other species of the genus, *Australoheros minuano* is a distinct evolutionary lineage. *Australoheros minuano* is similar only to *A*. *angiru*, *A*. *kaaygua mboapari*, *A*. *acaroides A*. *ricani*, *A*. *sanguineus*, *A*. and *A*. *scitulus* in presence of a zipper band along the middle of the side, and distinguished from all of those species by contrasting pattern of rows of small black spots on lateral scales.

**Fig 22:**
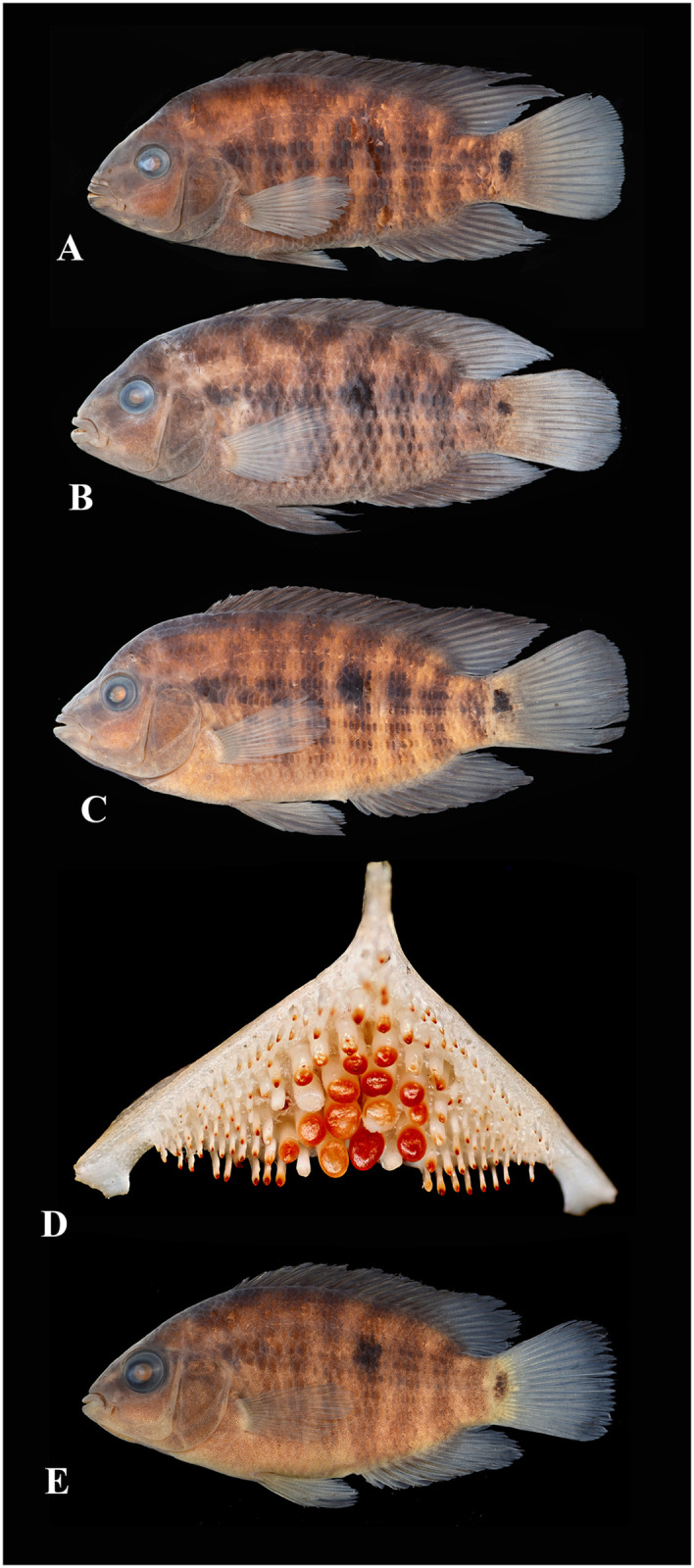
*Australoheros minuano*. **A.** adult female, 83.3 mm SL; Uruguay NRM 54147: Cerro Largo: Centurión: Paso del Centurión: Cañada Vigia, tributary to Rio Yaguarón about 2 km W of Rio Yaguarón. **B.** Adult female 87.8 mm SL; NRM 6140; Uruguay: Cerro Largo: Centurión: Paso del Centurión: Cañada Vigia, 2 km W of Rio Yaguarón. **C.** Adult female, 65.3 mm SL; NRM 54147; Uruguay; Cerro Largo: Centurión: Arroyo Ceibal down from bridge. **D.** NRM 54141, 42.2 mm SL mm SL; Lower pharyngeal tooth-plate in occlusal view. **E.** *Australoheros minuano*, young, 42.2 mm SL; NRM 54147, Uruguay; Cerro Largo: Centurión: Arroyo Ceibal down from bridge.

**Fig 23:**
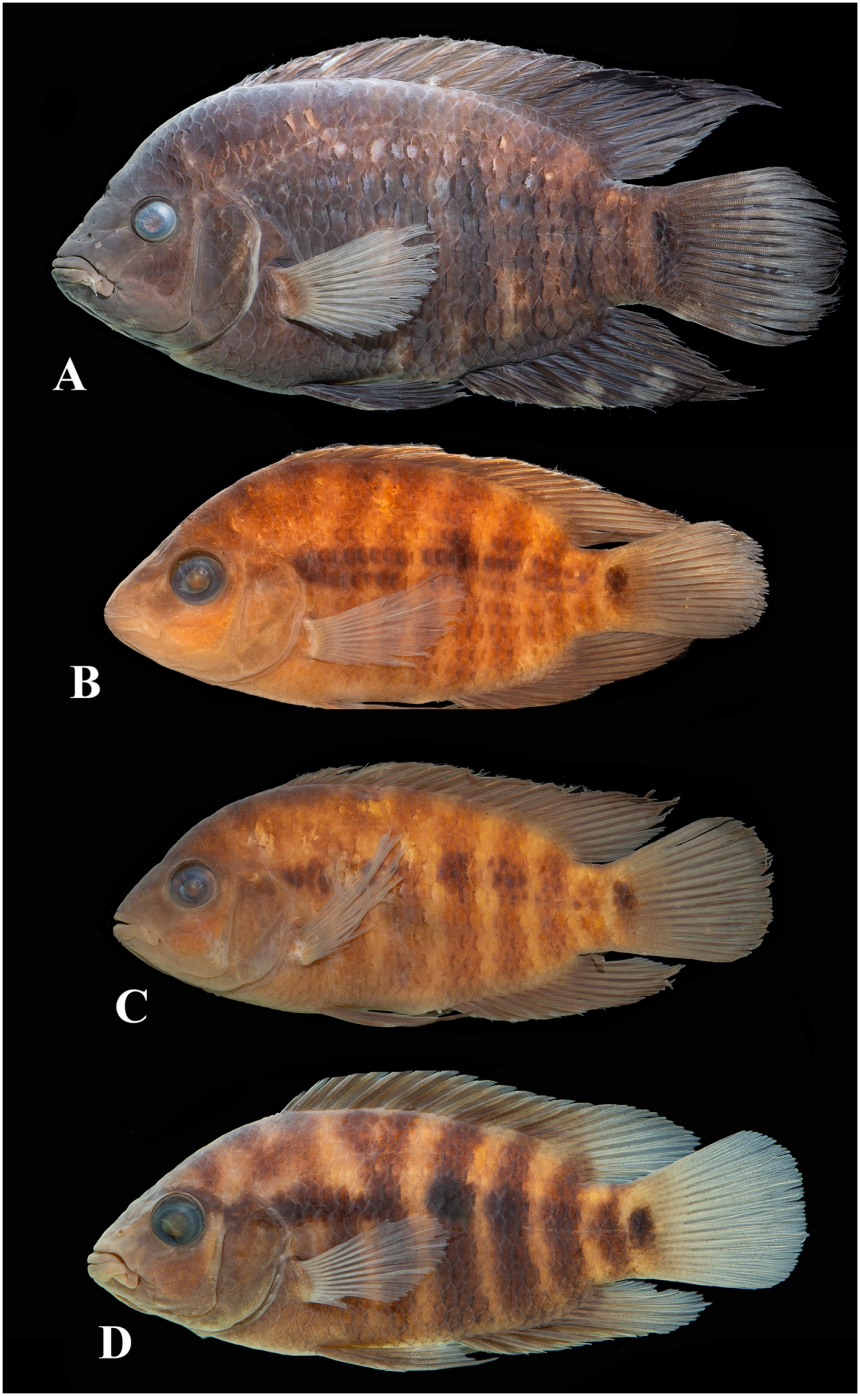
**A**. *Australoheros* sp., “Arapey”, adult female, 138.0 mm SL; NRM 52219; Uruguay: Salto: Rio Uruguay drainage,10 km from Valentin, below road 31 bridge, small stream within the Rio Arapey drainage**. B** *Australoheros minuano*, holotype, adult, not sexed, 70.8 mm SL; MCP 12710; Brazil, Rio Grande do Sul, Arroyo Canoin, Rio Uruguay drainage, road from Pirapo to São Nicolau. **C.** *Australoheros minuano*, paratype, adult, not sexed, 83.9 mm SL. MCP 46757; Brazil, Rio Grande do Sul, Arroyo Canoin, Rio Uruguay drainage, road from Pirapo to São Nicolau. **D.** *Australoheros ribeirae*, adult male, 63.4 mm SL; NUP 5514; Brazil: São Paulo: Tapiraí: Rio Ribeira de Iguape basin: Ribeirão do Prumo, tributary of Rio Verde, Sítio União, bairro doChá.

*Description of sample from Uruguay*. Meristic data are given in Tables 3–9, proportional measurements in Table 13. See Figs 22 and 23 for general shape.

Adults, about 50 mm SL and larger, moderately elongate, laterally compressed. Frontal contour ascending at about 30°, straight, slightly curved close to dorsal-fin base, which straight or slightly curved, steeply descending at base of soft dorsal fin. Caudal peduncle margins straight, parallel. Very slight indentation in frontal contour anterior to orbit; only one specimen observed with indication of nuchal protuberance and marked frontal indentation (Fig 23C). Subadults and juveniles with gently curved dorsal and ventral contours, approaching ovoid or rounded outline; frontal contour straight, frontal indentation absent. Gill cover with distinct indentation at dorsal junction of opercle and subopercle.

Mouth terminal, below level of orbit; tip of maxilla exposed, not reaching to vertical from anterior margin of orbit, jaws isognathous, but tooth band of lower lip closing opposite or posterior to upper tooth band. Lips moderately thick, both lip folds interrupted symphysially. Jaw teeth in 2–3 rows; in both jaws anterior 6 teeth in outer row slightly larger than the rest, pointed, slightly recurved; small second cusp present lingually. Teeth in outer hemiseries 6–13 in upper jaw, 10–17 in lower; 1–2 inner rows of smaller caniniform teeth in both jaws (about half height of anterior outer teeth, slightly smaller in posterior sequence). Abraded teeth not present.

Anterior insertion of dorsal fin at vertical from insertion of pectoral-fin base; first dorsal-fin spine about 1/3 length of last spine, length increase gradual from first to last spine. Soft dorsal fin with broad pointed tip, rays 4+5 longest, extending to about vertical through middle of caudal fin or slightly shorter. Anal-fin spines increasing slightly in length from first to last; soft dorsal fin with broad tip formed by rays 3+4, reaching at most to vertical through middle of caudal fin. Caudal fin rounded.

Pectoral fin inserted slightly anterior to pelvic-fin insertion, at distance from pelvic-fin spine corresponding to length of pelvic-fin base. Pectoral-fin tip rounded; pectoral fin reaching nearly to vertical from genital papilla. Pelvic fin long, pointed, first soft ray longest, reaching to genital papilla. Small specimens with more rounded vertical fins, pelvic fin with rounded tip, reaching to anal-fin insertion or slightly shorter.

Bases of dorsal, anal, and caudal fins scaled. Dorsal-fin base with basal scales from 7th spine, rows of interradial scales from12th, 13th or 15th spine, at most 6 slightly elongated scales between two spines, scale layer covering proximal half of fin; soft portion with thick basal scale layer, scales slightly more rounded than on spinous portion. Specimens smaller than 40 mm SL with scales basally on soft dorsal fin only. Anal fin with single row of basal scales, on soft portion added a dense layer of basal scales, up to for between two rays. Caudal fin with dense layer of scales basally, rows of small interradial scales reaching to middle of fin.

*Colouration in preservative*. Ground colour beige; markings dark brown or black. Cheeks pale brown, front and nape grey. Abdomen beige, may be grey along midline. Chest greyish brown or beige. Bar 1a posteriorly on caudal peduncle, brown, not extending onto caudal-peduncle margins. Bar 2 brown, straight vertical or slightly curved, extending between posterior insertions of dorsal- and anal- fins. Bar 3 brown, straight vertical or slightly curved, extending between dorsal-fin base and middle of anal-fin base. Bar 4 brown, straight vertical or slightly curved, extending from anterior insertion of anal fin up to dorsal-fin base, but may be forked or caudad inclined above upper lateral line. Bars 5–6 integer, brown or pale brown, straight vertical, extending from lower abdominal side, dorsally to dorsal-fin base, but may be paler or interrupted in pale zone along upper lateral line, and bar 5 may be forked above upper lateral line. Dark brown blotch immediately below first lateral lines scale, representing bar 7. Two dark brown bands representing bar 7 crossing nape. Bar 8 represented by dark brown blotch between orbit and upper end of preopercle. Bar 9 represented by two indistinct grey stripes between orbits. Vertical bars on side formed by uniform vertically extended pigmentation, overlain by dark scale bases in scale rows E1, 0, H1–4. Dark brown scale bases forming irregular horizontal lines in scale rows E1–2, 0, H1–3/4, separated by indistinct light interstripes. Continuous lateral band absent. Intensified dark scale bases in scale rows 0 and E1 may be contiguous and form two parallel stripes, intensified where crossing vertical bars. Midlateral blotch small, confined to bar 4, on E1 scale, may extend onto E2 scale, round or rectangular, indistinct or absorbed by bar 4. Similar, smaller blotch may be present in Bar 3 and/or Bar 5.

Dorsal-fin base black, rest of fin grey, interradial membranes black, dark grey or brown depending on preservation; vertical bars may be extended onto scaled dorsal fin base, but not forming prominent extension onto the dorsal fin. Anal fin pale grey, interradial membranes area darker grey, black or brown depending on presentation. Caudal fin brown basally, progressively lighter distally, interradial membranes grey, distal margin grey. Pelvic fin pale grey, brown or black. Caudal spot (bar 1p) round, square, or vertically narrowly elliptic, in most specimens present at base immediately dorsal to lower lateral line level, crossing 7 caudal-fin rays, but may extend as midbasal short vertical bar, or show as small blotches, one above and one below lateral line.

Small specimens, less than 50 mm SL, similar to adults in colouration, but vertical bars less contrasted, and not forked on dorsum. Prominent pattern of small dark brown spots formed from dark scale bases. Soft unpaired fins hyaline. Abdominal bars 3 (12), or 4 (6) in NRM 54147, 6397, 61408, and 61526.

*Specimens examined*. **Uruguay:** NRM 54123, 6, 32.6–47.5 mm SL; NRM 55067, 1, 24.5 mm SL; Cerro Largo: Rio Tacuari basin: small stream crossing Ruta 26, near Posta del Chuy, 32°24’50’’S 53°52’16’’W; 25 Oct 2005. S. Kullander et al.—NRM 54147, 13, 38–83.3 mm SL; Cerro Largo: Centurión: Arroyo Ceibal down from bridge, 32°9’6’’S 53°46’55’’W. 26 Oct 2005; S. Kullander et al.

NRM 55152, 1, 40.8 mm SL; NRM 54196, 4, 36.0–96.1 mm SL; Cerro Largo: Ramón Trigo: stream tributary of the Rio Negro crossing Ruta 26 Melo–Tacuarembó, at about km 389, 32°20’17’’S 54°37’57’’W; 27 Oct 2005; S. Kullander. et al.—NRM 61397, 2; 56.4–83.6 mm SL; Cerro Largo: Centurión: small stream crossing Ruta 7 ca 15 km from Paso del Centurión, 32°11’5’’S 53°54’23’’W; S. Kullander, F. Cantera, P. Lasnier, 28 Feb 2011.—NRM 61408, 2, 87.8–92.9 mm SL; Cerro Largo: Centurión: Paso del Centurión: Cañada Vigia, tributary of the Rio Yaguarón about 2 km W of Rio Yaguarón, 32°8’23’’S, 53°45’18’’W; S. Kullander, F. Cantera, P. Lasnier, 27 Feb 2011..—NRM 61526, 1, 41.3 mm SL; Cerro Largo: Paso del Centurión, southern end of small lake slightly east of Rio Yaguarón, 32°8’34’’S 53°43’46’’W; 27 Feb 2011. S. Kullander et al.—NRM 51079, 9, 7.4–74.7 mm SL; Tacuarembó: Ruta 31, km 196, Cañada de los Peña, 31°23’27.2"S 56°07’23.5"W, P. Laurino et al., 16 Mar 2003.**—**NRM 52769, 1 juv.; Rivera: Rio Tacuarembó basin: Arroyo Rubio Chico, at km 239.4 on road 30, under bridge; S. Kullander et al., 11 Oct 2004.—Brazil: Rio Grande do Sul: MCP 16196, 1, 64.7 mm SL; São Luiz Gonzaga: Rio Piratini basin: Arroio Ximbocuzinho on road São Luiz Gonzaga to Bocoroca, 4 km from São Luiz Gonzaga, 28°27’00.0"S 54°58’00.0"W; P.H.Wimberger, R.E.Reis, J.F.Pezzi da Silva et al., 11 Dec 1992.—MCP12724, 1, 75.3 mm SL, paratype: MCP 12724, 42, Brazil, Rio Grande do Sul, Arroyo Paso do Alto, Rio Uruguai basin, 5 Nov 1988. C.AS. Lucena, L. Bergmann, E. H.L. Pereira, P. Azevedo and A. Ramírez.—MCP 11227, 24 paratypes, Arroyo Garupa, divisa Quarai /Alegrete, Rio Uruguai basin, C.A.S. Lucena, L. Bergmann and P. Azevedo,12 Nov.1988.

*Australoheros* “Arapey”: NRM 52121, 1, 99.6 mm SL; Salto: ponded stream, tributary of the Rio Arapey Grande, at ca km 100 on road 4, 31°31’59.9"S 57°01’49.8"W; S. Kullander et al., 7 Oct 2004.—NRM 52219, 1,138.0 mm SL; NRM 52222, 1, 55.5 mm SL; Salto: 10 km from Valentin, below road 31 bridge, small stream within Rio Arapey basin, 31°16’31.7"S 57°09’20.3"W; S. Kullander et al., 7 Oct 2004.

*Geographical distribution* (Fig 1). In Uruguay, upper Rio Yaguarón, upper Rio Tacuarí, both draining to Laguna Merín; and headwaters of the Rio Negro (Rio Tacuarembó); elsewhere, in the Rio Piratini basin in Brazil. Localities near Centurión were recorded as within the Yaguarón basin, but most likely include tributaries of the Rio Negro.

*Comments*. Three specimens of *Australoheros* from the lower Rio Arapey could not be assigned to species with certainty, and are here referred to as *Australoheros* “Arapey”. Two of the specimens had a colour pattern similar to *A*. *facetus*, i.e. without distinct dark spots in the vertical bars and without horizontal stripes; one of those had an *mt-co1* sequence similar to but distinct from that of other *A*. *minuano* (Fig 23A). DNA samples were not available from the other two Arapey specimens, but one of them had the lateral stripe pattern found in *A*. *minuano* and *A*. *acaroides*.

*Australoheros minuano* was described based on four samples from tributaries of the Rios Piratini the and Quaraí in the Rio Uruguay basin in Brazil [4]. The type locality, the Arroio Canoin in the lower Piratini basin, is the same as for *Australoheros charrua*. The paratypes from the Quaraí basin were of small size, and the identification may have to be revised. Only one new record is known from the Rio Piratini basin, from the Rio Ximbocuzinho far south of the earlier records.

Our material from southeastern Uruguay was identified as *A*. *minuano* with reservation. Available evidence is compatible with species identity despite considerable separation of the Brazilian and Uruguayan localities, but the Uruguayan specimens agree with *A*. *minuano* from the type locality by relative slender shape, all meristic data, and the melanophore colour pattern with double blotches in the rande do Sul, Arroyo Canoin, Rio Uruguay basin, road from Pirapo to São Nicolau.

The “*Cichlasoma*” sp. “Tacuarembó” reported by Litz et al. [107] was identified as *A*. *minuano* by Říčan and Kullander [4]. Specimens from the same locality were available at NRM (NRM 51079) but for some reason not recognised as such in the collection and consequently not included in the original description. The live colour description given by Říčan and Kullander [4] was based on the publication by Litz et al. [107], and has not been confirmed for the Arroio Canoin sample.

In addition to *A*. *charrua* and *A*. *minuano* from Arroio Canoin, two specimens were identified by Říčan and Kullander [4] as *A*. *kaaygua* (actually *A*. *angiru*) which is otherwise restricted to the upper Uruguay basin. The records from Centurión were recorded as from the Rio Yaguarón, but more likely pertain to small streams headwaters of the Rio Negro basin, close to the Rio Yaguarón.

Recognition of *A*. *minuano* was based on the distinct mitogenome. No morphological characters were found to discriminate between *A*. *acaroides* and *A*. *minuano*. Both were characterised by the colour pattern, with scale margins forming irregular lateral stripes. The accentuated black spots on the scale bases were unique for *A*. *minuano*, but the rest of the colour pattern variation fell within that of *A*. *acaroides*. Colour pattern distinguished *A*. *minuano* and *A*. *acaroides* from *A*. *facetus*, in which lateral stripes were absent.

Uruguayan *A*. *minuano* had a slightly shorter lower jaw than *A*. *acaroides*. The slope on SL was identical, and the intercepts were significantly different (F = 35.060, P = 0.000), but the size range for *A*. *minuano* was smaller. In general aspect adult *A minuano* was strikingly elongate, with gently curved frontal contour, contrasting with most congeneric species, which are deep-bodied with straight descending frontal contour, often with a slight frontal indentation.

No freshly collected specimens or DNA was available of *Australoheros minuano* from the type locality. *Australoheros minuano* was distinguished mainly based on the colour pattern, including a lateral band described by Říčan and Kullander [4] as shifting one scale up along the side. This, however, is the pattern of dark stripes along the middle of the side present in most southern species, most distinct in in *A*. *ricani*, *A*. *acaroides*, and *A*. *kaaygua*. A similar colour pattern is present in *A*. *charrua*, sharing type locality with *A*. *minuano*. *Australoheros minuan*o and *A*. *charrua* were separated mainly on the colour pattern, *A*. *charrua* with a larger midlateral blotch, and slight differences in the shape of the head [4].

#### *Australoheros ribeirae* Ottoni, Oyakawa & Costa, 2008

*Australoheros ribeirae* Ottoni, Oyakawa & Costa, 2008 [5]: 76, fig. 1 (holotype MZUSP 4228; type locality Brasil: Estado de São Paulo; Município de Sete Barras: lagon [sic] near the Sr. Celso farm, road Sete Barras-EL Dorado [sic]).

*Definition*. Based on the position in the *mt-cyb and mt-coI* trees and minimum uncorrected p*-*distance in *mt-coI* at 2% or more from all other species of the genus, *Australoheros ribeirae* is a distinct evolutionary lineage. Specimens of *A*. *ribeirae* can be distinguished from southern species of *Australoheros* by absence of zipper band, but no morphological autapomorphy was registered.

*Description of sample*. Meristic data are given in Tables 3–9, proportional measurements in Table 14. Fig 23(D) Shows general aspect.

Moderately elongate to deep-bodied, laterally compressed. Predorsal contour straight ascending or with slight indentation anterior to orbit, curved anterior to dorsal-fin origin. Nuchal elevation absent in both sexes. Dorsal profile curved, slightly descending to end of dorsal-fin base Caudal peduncle slightly tapering, contours straight. Prepelvic contour slightly curved or straight. Abdominal contour straight or slightly convex. Anal-fin base contour straight ascending. Head short, laterally compressed. Snout short, blunt, subtriangular in lateral aspect, rounded in dorsal aspect. Mouth terminal, forward directed, close to horizontal line from lower margin of orbit. Lips moderately thick, lower lip fold continuous anteriorly. Jaws equal in anterior extension; maxilla and premaxilla not reaching to vertical from anterior margin of orbit. Orbit removed from frontal contour, in middle of head length, in upper half of head. Teeth in both jaws caniniform, slightly recurved, in outer row slender, increasing in size toward symphysis. Bicuspid teeth not observed. Teeth in outer hemiseries in upper/lower jaw 12–14/15–16. Inner teeth in two rows. Gill rakers externally on first gill-arch 2 epibranchial, one in angle and 6–8 ceratobranchial. Microbranchiospines present externally on second to fourth gill-arch.

Dorsal-fin spines increasing in length to about 6th, from which subequal, last spine longest, soft dorsal-fin rounded, fifth soft fin-ray longest, reaching to about middle of caudal fin. Soft anal fin rounded, with short point formed by fifth soft fin-ray, reaching to about middle of caudal fin. Pectoral fin rounded, fifth ray longest, reaching to vertical from anal orifice. Pelvic fin pointed, first soft ray longest, reaching to base of fourth anal-fin spine. Caudal fin rounded.

Scales on body finely ctenoid. Predorsal midline scales about 11–12, slightly smaller than flank scales, covered by skin, only part with exposed margin, irregularly arranged, mixed cycloid and weakly ctenoid. Chest scales ctenoid, almost size of flank scales. Cheek scales in 3–4 series, covered by skin. Opercular scales cycloid, covered by skin. Between first upper lateral line scale and dorsal-fin origin 3 scales Accessory lateral line scales absent from caudal fin. One large and one small scale separating last scale of upper lateral line from dorsal-fin base. Prepelvic scales anteriorly small, cycloid and without free margin, remaining scales only slightly smaller than flank scales, weakly ctenoid and with free margin. Fin scales ctenoid. Row of minute scales along dorsal-fin base from 9th spine, basal squamation gradually expanded, interradial scales in single series, from 13^th^ spine, on soft fin up to 4 scales between two soft rays, scales absent from last one or two interradial membranes. Soft anal fin with narrow basal scale layer.

*Colouration in preservative*. Adults, about 40 mm and larger with ground colour fawn or pale grey. Bars 1(a, p) to 6 present, dark brown, 2–3 scales wide; Bar 6 absent above lateral band; Interbars 1–3 scales wide. Lateral band on E1 scale row extending from gill cleft caudad to Interbar 4. Bar 5 integer, terminating dorsally in lateral band or extending dorsally at most to dorsal-fin base. Bars vertical or dorsal and ventral portions of Bars 2–4 slightly caudally inclined. Bars 5 and 6 forming Y-marking. Dorsal branches of bar 7 present, expressed as short rostrad inclined light brown or grey bars crossing anterior dorsal-fin base and occiput, respectively. Midlateral blotch round, black or dark brown present in Bar 4 on E1 scales and parts of adjacent scales, barely exceeding Bar 4 in width; corresponding portions of bars 2 and 3 may be slightly widened or more intensely pigmented but distinct spots absent from bars 2 and 3. Bar 5 split in one specimen, resulting in 4 abdominal bars. Bars 2–4 extending dorsad onto base of dorsal fin. In Bars 2–6 ventral to scale row 0, small black spot at middle of posterior margin of each scale. Caudal blotch (Bar 1b) indistinct, minimal, or distinct, light or dark brown, darker on or restricted to upper lobe, or forming vertical bar across middle of caudal-fin base. Vertical fins pale grey, translucent. Pelvic fin dark grey distally, lighter medially.

*Geographical distribution* (Fig 1). Available records were restricted to tributaries of the Rio Juquiá and the lower Rio Ribeira de Iguape.

*Material examined*. All from Brazil: São Paulo: Rio Ribeira de Iguape basin.

LBP 6739, 1, 79.9 mm SL; Apiai: stream near Apiai; C. Oliveira et al., 12 May 1997.—

LBP 16839, 1, 68.5 mm SL; Miracatu: Rio Fau, 24°12’26.5’’S, 47°28’36.9’’W; R. Devidé, 14 Aug 2012.—LBP 2133, 2, 27.8–69.4 mm SL; Pedro de Toledo: Rio do Peixe, 24°16’35.5’’S, 47°13’33.2’’W; M.C. Chiachio, R. Devidé and D.C. Ferreira, 12 Mar 2003.—NUP 5514, 1, 63.4 mm SL; Tapiraí: Ribeirão do Prumo, tributary of the Rio Verde, Sítio União, bairro do Chá, 24° 1’ 42.71"S; 47°36’50.08"W; A. Nogueira Bozza, 14 Feb 2008.

*Comments*. *Australoheros ribeirae* was described [5] based on the holotype, 43.7 mm SL, and 43 paratypes, 12.3–75.5 mm SL from the lower Rio Ribeira de Iguape and its tributaries, the Rio Juquiá and the Rio Pariquera Mirim. The diagnosis exclusively referred to a unique combination of characters, including 9–10 dorsal-fin rays, 6–7 anal-fin spines, 8 anal-fin rays, 14 precaudal and 12 caudal vertebrae, 24–26 scales in longitudinal series, 16–18 scales in upper lateral line, ratios of head depth, last dorsal-fin spine, predorsal length, preorbital depth, head width; narrow ectopterygoid, truncate process of second vertebrae, epibranchial 2 with two long processes, anterior arm of epibranchial 1 long; variation in the vertical bars, absence of spots on the head, and sides usually red. Comparative illustrations emphasise absence of forked vertical bar in comparison with *A*. *facetus* (fig. 2), and differences from *A*. *facetus* in in the shape of pharyngobranchials and epibranchials (fig. 4). There are two figures labelled Fig 5. One of them, referred to as fig. 6 in the text, shows a narrower ectopterygoid in *A*. *ribeirae* than in *A*. *facetus* and the ‘new species of Rio de Janeiro’, but the caption to fig. 5 (= fig. 6) refers to the type locality of *Dicrossus gladicauda*, and consequently is not informative concerning which species is illustrated with a wider ectopterygoid. The other fig. 5 shows the caudal skeleton of *A*. *ribeirae* and ‘New species of Rio de Janeiro‘. The text refers to fig. 5 as showing ‘process of second vertebra truncate [in ‘New species of Rio de Janeiro‘] vs pointed. The process is probably the neurapophysis of preural 2. No voucher data or specimen lengths were provided for the osteological drawings; but they were used again by Ottoni & Costa [6] and are commented on above. The comparative drawing of colour pattern in *A*. *ribeirae* and *A*. *facetus* shows a strange pattern in the illustration of *A*. *facetus*, with multiple bifurcations, and a very large triangular Bar 1, that we have not observed in *A*. *facetus*.

Too few specimens were available to permit a full morphological description of *A*. *ribeirae*; DNA data, however, confirmed the presence of a distinct species of *Australoheros* in the Rio Ribeira de Iguape basin. None of the specimens had the unique combination of characters that made up the diagnosis of the species; for instance, none of them had the required body depth 51.3% of SL. The narrow ectopterygoid remains a synapomorphy shared only with *A*. *ipatinguensis*, but could not be verified. Table 21 shows that counts and proportional measurements overlap with other species from the Sudeste.

In the phylogenetic analysis (Figs 12 and 14), *Australoheros ribeirae* and *A*. *sanguineus* were rerecovered as a clade in a trichotomy with the northern and southern species and they are distributed in separate but adjacent river basins. Only few specimens were available of each species, and collecting localities were restricted to small areas, but the phylogenetic position suggested that they represent two successive isolated speciation events.

*Australoheros ribeirae* was similar to *A*. *sanguineus* in meristic data and proportional measurements. The major difference may be in the colour pattern. In *A*. *ribeirae*, there were small black spots at the bases of the scales of the lower sides, absent in *A*. *sanguineus*, but similar to the ventral spots in *A*. *minuano*; the lateral band had irregular margins but did not show the zipper pattern characteristic of southern species of *Australoheros*.

#### *Australoheros ipatinguensis* Ottoni & Costa, 2008

*Australoheros ipatinguensis* Ottoni & Costa, 2008 [6]: 214, fig. 7]6]; spelt *ipatiguensis* on p. 216. (holotype UFRJ 7553; type locality Brazil: Estado de Minas Gerais: Município de Ipatinga: córrego Braúna, rio Doce basin; corrected by Ottoni et al. (2020) [108], to Brazil: Estado de Minas Gerais: Municipio de Belo Oriente: Córrego Braúna, a tributary of Rio Santo Antônio, Rio Doce Basin, nearby[sic] a secondary road to the MG-232 road between Santana do Paraíso and Belo Oriente, between coordinates 19°16’10’’S 42°30’48’’W and 19°17’09’’ S 42°31’26’’W.

*Australoheros autrani* Ottoni & Costa, 200 [6]: 209: fig. 1 (holotype UFRJ 7256; type locality Brazil: Estado do Rio Janeiro: Município de Silva Jardim: rio Aldeia Velha, BR-101, rio São João basin).

*Australoheros muriae* Ottoni & Costa, 2008 [6]: 220, fig. 10 (holotype MNRJ 32181; type locality Rio de Janeiro: Itaperuna: rio São Domingos, tributary from [sic] rio Muriaé, rio Paraíba do Sul basin)

*Australoheros saquarema* Ottoni & Costa, 2008 [6]:225, fig. 13 (holotype UFRJ 7255, type locality Brazil: Estado de Rio de Janeiro: Município de Saquarema: rio Buração)

*Australoheros macaensis* Ottoni & Costa 2008 [6]: 218, fig. 9 Holotype UFRJ 7573, 66.8 mm SL; type locality Brazil: Estado do Rio de Janeiro: Município de Macaé: rio dos Quarenta, BR-101 (S 22° 13,3`/W41°,45,580`).

*Australoheros capixaba* Ottoni, 2010 [8]: 20, fig. 1. (holotype UFRJ 7725; type locality Brazil: Estado do Espírito Santo stream under a bridge in a ES street, between Município de Jaguaré and Município de São Mateus (S 18°34,953’ e WO 4026,115’).

*Australoheros perdi* Ottoni, Lezama, Triques, Freagoso.Moura, Lucas & Barbosa, 2011 [10]: 140, fig. 2 (holotype DZUFMG 071; type locality Brazil, Minas Gerais state, Marliéria municipality, lacustrine region of the middle Doce River basin, Doce River Valley, Parque Estadual do Rio Doce, Lagoa Gambazinho, 19°47’10.6’’S, 42°34’48.3’’W).

*Definition*. Based on the position in the *mt-cyb* and *mt-coI* trees and minimum *p*-distance in *mt-coI* exceeding 2% from all other species of the genus, *Australoheros ipatinguensis* is a distinct evolutionary lineage. No morphological autapomorphy was registered. Specimens of *A*. *ipatinguensis* can be distinguished from most congeneric species by colour pattern and scale cover: zipper band absent (present in *A*. *acaroides*, *A kaaygua*, *A*. *minuano*, *A*. *sanguineuus*, *A*. *mboapari*, and *A*. *ricani*), absence of brown or blue iridescent markings on sides of head (present in *A*. *scitulus*, *A*.*forquilha* and *A*. *ykeregua*); similar to *A oblongus* in adults with long middle dorsal and anal fin rays reaching end of caudal fin or beyond, and pelvic fin reaching to middle of anal-fin base fin (anal dorsal and anal fins reaching at most to middle of caudal fin; pelvic fin reaching at most bases of anterior anal-fin spines in all other species of *Australoheros*); adults distinguished from *A*. *oblongus* by subovoid lateral contour with smoothly descending frontal contour and slight or absent indentation at level of upper margin of orbit; bar 1p situated at middle of caudal-fin base vs mainly confined to the dorsal half of the caudal-fin base, and Y mark almost omnipresent vs typically absent.

**Fig 24:**
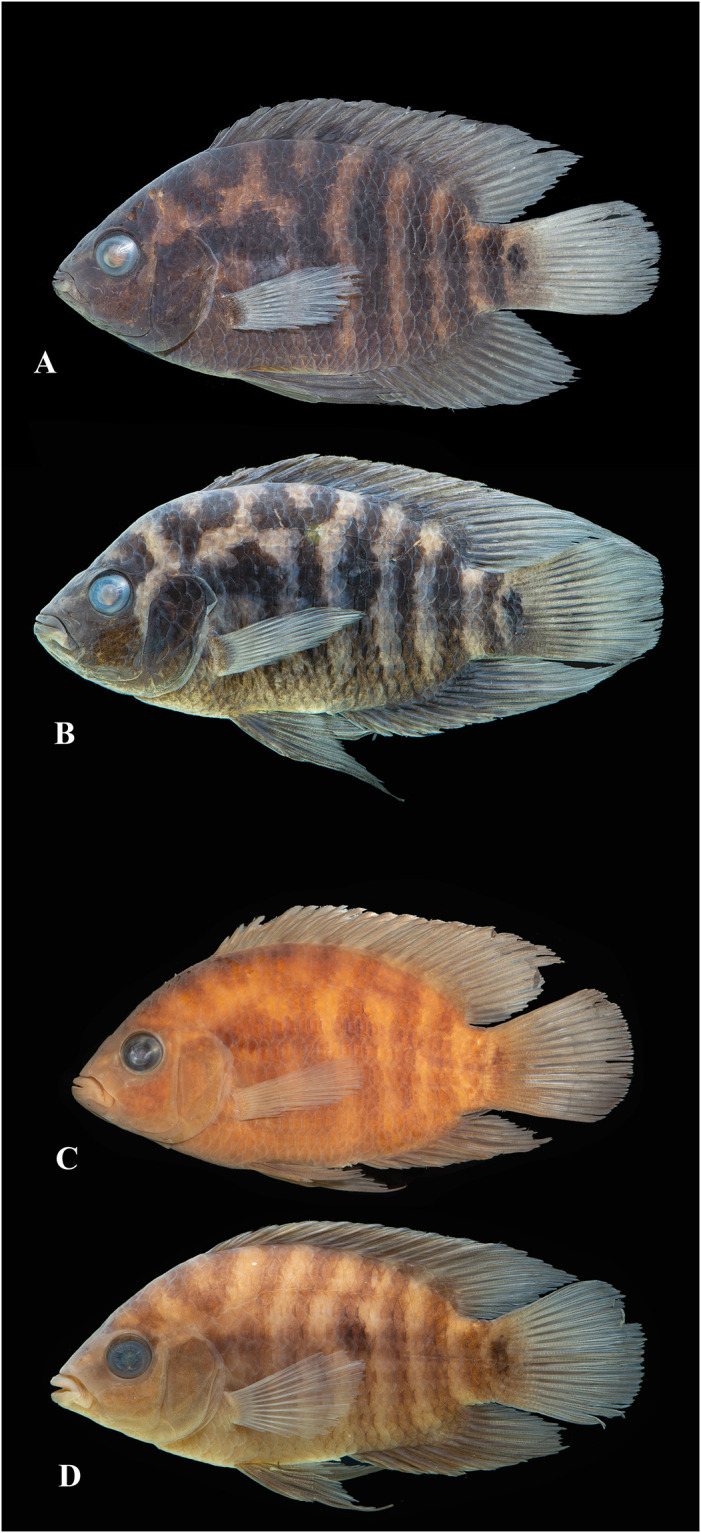
Australoheros ipatinguensis. **A**. young male, 54.5 mm SL; MCP 36657; Brazil: Rio de Janeiro: São Francisco de Itabapoana: Rio Guaxindiba on Fazenda Santa Cruz, near Maniva. **B**. Adult male, 83.3 mm SL; MNRJ 47307; Brazil: Rio de Janeiro: Itaperuna: bridge over the Córrego São Domingos, on road toward Fazenda São Domingos. **C**. Adult female, MZUSP 16178, 77.3 mm SL; Brazil: Rio de Janeiro: Rio Paraíba do Sul at São João da Barra. **D.** Adult male, 60.6 mm SL; UFBA 4995; Espírito Santo: Pedro Canário: Rio da Samambaia, on BR.

*Description of sample*. Meristic data are given in Tables 3–9, proportional measurements in Table 15. Fig 24 shows representative specimens for variation in general shape and colour pattern.

Sexes isomorphic except vertical and pelvic fins slightly longer in some large males than in large females, and genital papilla in males slender, conical, in females wider, blunt. Moderately elongate to deep-bodied; frontal contour straight ascending or with minor curvature, continuous with curved dorsal-fin base contour; only large specimens with indication of nuchal swelling and minor indentation in frontal contour at level of orbit. Prepelvic contour straight, less inclined than frontal contour. Caudal peduncle contours contour slightly curved or straight. Abdominal contour straight horizontal, except slightly convex in females. Anal-fin base slightly convex, ascending. Head short, laterally compressed. Snout short, blunt, subtriangular in lateral aspect, narrowly rounded in dorsal aspect. Mouth terminal, forward directed, at level of lower margin of orbit or slightly more dorsal. Lips moderately thick, Jaws equal in anterior extension; maxilla and premaxilla not reaching to vertical from anterior margin of orbit. Orbit removed from frontal contour, in middle of head length, in upper half of head. Teeth in outer hemiseries in upper/lower jaw 9–14/13–20. Inner teeth in 2 rows, in both jaws. Gill rakers externally on first gill-arch 1–2 epibranchial, one in angle and 5–7 ceratobranchial. Microbranchiospines present externally on 2nd to 4th gill-arch.

Scales on body finely ctenoid. Predorsal midline scales about 11–14, about half size of flank scales, covered by skin, only part with exposed margin, irregularly arranged, weakly ctenoid. Cheek scales in 3(rarely 4 or 5) rows, cycloid, covered by skin. Opercular scales mixed cycloid and ctenoid, covered by skin or margin partly exposed. Accessory lateral-line scales observed in three specimens, with tube between 3rd and 4th dorsal ray and 4th and 5th ventral rays; 2nd and 3rd dorsal rays; and 4th and 5th ventral rays, respectively. Between first upper lateral line scale and dorsal-fin origin 3 or 4 large or 3 large and one small scale. Between last upper lateral line scale and dorsal fin one large or one large and one small scale. Prepelvic scales about half size of flank scales, embedded in skin, without free margin. Lateral chest scales ctenoid, with exposed margin, about 2/3 size of flank scales. Fin scales ctenoid. Row of minute scales along dorsal-fin base from ^12th^ or 13^th^ spine, interradial scales from between 15th to 16th spine caudad, on soft fin in one or two rows with up to 7 scales between two soft rays, scales present also on last two interradial membranes. Anal fin with narrow basal scale layer from 4th spine, on soft-rayed portion up to 6 scales in interradial row.

Dorsal–fin spines increasing in length to 7th from which subequal, last spine longest, soft dorsal-fin rounded or subacuminate in young; in large adults with pointed tip, 5th ray longest, at most reaching to end of caudal fin or slightly longer. Soft anal fin rounded or subacuminate in young; in adults with pointed tip, 4th or fifth ray longest, reaching to beyond middle, or posterior 1/4th of caudal fin. Pectoral fin rounded, 4th longest, reaching to vertical from base of first or second anal-fin spine. Pelvic fin pointed, first ray longest; in young reaching to base of 4rd anal-fin spine; in large adults produced, at most to soft anal- fin base. Caudal fin rounded.

*Colouration in preservative*. Ground colour fawn or pale grey. Bars 1(a, b) to 6 present, dark brown or dark grey, 2–3 scales wide; bar 6 absent above lateral band or extending only short distance above it bar 5 exceptionally interrupted in E1 scale row; Interbars 1–2 scales wide; lateral band on E1 scale row extending from gill cleft caudad to Interbar 4; Bar 5 bars 5 and 6 integer or, typically coalesced dorsally to form U-shaped marking (when Bars 5 and 6 interrupted in E1 scale row) or Y shaped-marking (when Bar 5 continuous across scale row E1), open to dorsal-fin base, extending dorsally at most to dorsal-fin base. Bars vertical or dorsal and ventral portions of bars 2–4 slightly caudally inclined. Dorsal portions of bar 7 present, expressed as short rostrad inclined brown or grey bars crossing anterior dorsal-fin base and occiput, respectively. Midlateral black or dark brown blotch present in Bar 4 on E1 scales and parts of adjacent scales; may be slightly widened or more intensely pigmented but distinct spots absent from Bars 2 and 3. Bars 2–7 extending dorsad onto base of dorsal fin, and deep black blotch particularly prominent in most specimens in above Bar 5. In Bars 2–6, vertical black stripe may be present at middle or posterior margin of scales below scale row 0. Caudal blotch (Bar 1p) distinct, expressed as vertically dark grey or brown extended oval blotch across middle of caudal-fin base; or round blotch on dorsal lobe, and indistinct paler pigmentation on lower lobe. Vertical fins pale grey, semitranslucent. Pelvic fin dark grey distally, lighter medially.

*Geographical distribution* (Fig 1). *Australoheros ipatinguensis* has a wide distribution along the coast of southeastern Brazil, from Saquarema north to Euanópolis in the Rio Buranhém basin based on morphology, and from the Rio Macaé north to Itaúnas based on DNA samples. The corrected type locality [108] is still close to Ipatinga, and within the Rio Doce basin, just in the next municipality, and the correction has no immediate practical consequences.

*Comments*. The name *ipatinguensis* was explained [6] as being ‘in reference to the city where the new species was collected’, referring to the municipality and city of Ipatinga. The extra ‘u” in the name remains unexplained.

*Australoheros ipatinguensis* was described based on the holotype, 53.5 mm SL, and 14 paratypes, 9.6–44.5 mm SL, from the Rio Doce basin. The diagnosis is based on character states in combination, including 12 caudal vertebrae, absence of marks on the side of the head; 15 dorsal fin spines, 10–11 dorsal fin rays, 9 anal fin rays, ‘common snout of *Australoheros’* 24–25 proximal radials in dorsal fin; narrow ectopterygoid, absence of ‘depression on head’ [means indented frontal contour; but minimal indentation shown on the photo of the holotype, fig. 7 although the outline of the specimen on the image has been edited with photo editing software]; 13 proximal radials in anal fin base; arm of epibranchial 1 long, arms of epibranchial 2 with two short tubular processes, 10 rib pairs, 25–26 scales in a longitudinal row, body depth 47.3–51.2, last dorsal-fin spine 14.2–16.6% of SL, last anal-fin spine length 14.3–15.6%; arms of trunk bar 7 with the same width. No autapomorphy is indicated.

Nominal species listed here as synonyms of *Australoheros ipatinguensis* were described by Ottoni and collaborators with diagnoses that refer to character states pertaining to southern species (spots on head absent, 12 caudal vertebrae), number of fin spines and rays, number of proximal radials in dorsal and anal fins, number of pairs of ribs and, caudal peduncle length/SL ratio, one or two long tubular processes on arms of epibranchial 2, long or short anterior arm on epibranchial 1, width of the ectopterygoid. Characters unique to particular species in the Uruguay basin are irrelevant here. The caudal peduncle is very short in most species of *Australoheros*, including *A*. *ipatinguensis*, and besides that no regression analysis was made, values may have low reproducibility. The counts of proximal radials in the dorsal and anal fins is a duplication of fin-ray counts, but minus one for the compound anterior anal-fin pterygiophore, and better resolution in whether the last fin ray is independent or attached to the pterygiophore of the penultimate fin ray. Osteological characters were commented on above p. 00. There are no indications on how many specimens or sizes were used to generate the osteological data. Ottoni and Costa [6] did not provide frequencies for their meristic data; such were available for species described in later papers, and visualise modality and potential extreme values in ranges, but the only representatives of *A*. *ipatinguensis* with frequencies are then the nominal species *A*. *capixaba* and *A*. *perdi*. In the end, what we can comment on is mainly fin-ray counts, scale counts and colour pattern.

Table 21 displays characters taken from the species diagnoses in the original descriptions that lists proposed differences within *A*. *ipatinguensis* as here understood. Although Ottoni et al. [47] indicated synonymy of *A*. *autrani* and *A*. *saquarema*, the morphological diagnoses of those species were not updated at the same time; hence it may be appropriate here to refer to the original diagnoses. In this tabulation, only the low number of pectoral-fin rays in *A*. *perdi* might suggest a unique condition for the type series.

The tabular comparison shows relative internal consistency Some characters stand out and disagree with our data, particularly the count of scales along the middle of the side (E1 scales in our terminology, indicated as the same E1 by Ottoni [9]. The counts are too high whichever method was used, but internally consistent. *Australoheros oblongus* and *A*. *ipatinguensis* have 24, rarely 23 E1 scales. Counting scales on the image of the holotype of *A*. *muriae* (Ottoni & Costa. 2008: fig. 10), we arrived at maximum of 24, same as on the paratype MCP 42584, although the range given for *A*. *muriae* is the extreme 27–29. The number of pectoral fin-rays (14–15) is higher than our counts (12–13). In the osteological data, *A*. *ipatinguensis* sensu Ottoni & Costa, is distinguished by narrow ectopterygoid and short epibranchial 2 processes. The dorsal-fin counts for *A*. *perdi* are somewhat low, as commented below, but not out of range. None of the meristic characters or proportional measurements is unique for any of the nominal species in Table 21.

*Australoheros autrani* was based on the holotype from Silva Jardim in the Rio São João basin, and 25 paratypes, all from the Rio São João, a small coastal stream just south of Rio Paraíba do Sul. The holotype was not figured. The diagnosis distinguishes *A*. *autrani* from other species in the same region by 10 pairs of ribs; anal-fin rays 9–10; proximal radials in dorsal fin 25–26; 15–16 dorsal fin spines, 10–12 soft dorsal-fin rays; 13–14 proximal radials in the anal-fin base: absence of ‘depression on head’ [indented frontal contour]; caudal peduncle length 10.2–11.9% of SL; arms of bar 7 of equal width, two long tubular processes on arms of epibranchial 2, and wide ectopterygoid. No autapomorphy is signalled, and no character states discriminating from *A*. *saquarema*. Identified only on the basis of the locality, our sequences of *A*. *autrani* confirmed a unique haplotype, but at less than 2% uncorrected p distance in *mt-coI* from other *A*. *ipatinguensis* and no morphological autapomorphy; hence our data did not support species status.

The original description of *Australoheros muriae* was based on 20 specimens, 20.9–121.3 mm SL from the Rio Muriaé and tributaries. The Rio Muriaé is a left bank tributary of the lower Rio Paraíba do Sul. The diagnosis lists character states in combination only, including 12 caudal vertebrae; marks absent from sides of head; 15 dorsal-fin spines; 14–15 pectoral-fin rays; 11–12 soft dorsal-fin rays; 9–10 anal-fin rays; 24–25 proximal dorsal-fin radials; caudal peduncle length 10.2–11.9% SL; wide ectopterygoid; epibranchial 2 arms with long tubular processes; arm of epibranchial 1 long; arms of trunk vertical bar 7 of equal width, and ‘common snout of *Australoheros’*. It is distinguished from *A*. *saquarema* and *A*. *macaensis*. by dorsal spine number (16 vs 15). In our material from the Rio Muriaé, seven had 15 spines, two had 16. It was distinguished from *A*. *saquarema*, *A*. *macaensis and A*. *ipatinguensis* by more soft dorsal-fin rays (11–12 vs 10–11). This range overlapped also with the counts given for *A*. *autrani* (10–12). *Australoheros muriae* is further distinguished by more anal-fin rays than *A*. *macaensis* (9–10 vs 8–9), which again is overlapping counts within the range of 8–10 for *A*. *ipatinguensis* in the restricted sense. *A*. *muriae* is distinguished from *A*. *autrani* by fewer dorsal-fin proximal radials (24–25 vs 25–26), again overlapping, and potentially different only from *A*. *perdi* (22–24) in the species range 22–26; and shorter caudal peduncle. From *ipatinguensis* it is distinguished by wide ectopterygoid and more scales in longitudinal row (27–29 vs 25–26).

*Australoheros saquarema* was based on the holotype, 80.3 mm SL, and 25 paratypes, 30.4–79.2 mm SL from the Rio Buração and Rio Tinguí, two small streams flowing to the Lagoa Saquarema and located close to the Rio São João. The Lagoa de Saquarema itself is a marine lagoon. The diagnosis is based on characters in combination only, and refers to 14–15 proximal radials in the anal fin; head with depression near the snout in specimens longer than 30.0 mm SL; 10 rib pairs; 17–18 scales in the upper lateral line; wide ectopterygoid; arms of epibranchial 2 with two long tubular processes; arm of epibranchial 1 long, and arms of trunk vertical bar 7 with the same width. The description is illustrated with a live colour photo of one of the paratypes. No character state discriminating from *A*. *autrani* isas listed in the diagnosis. Proximal anal radials 13–14 in *A*. *autrani* vs 14–15 in *A*. *saquarema* are mentioned reciprocally, but the overlap is non-discriminating.

A potential autapomorphy is described as ‘depression near the snout in specimens over 30 mm SL’. This state is not illustrated, but as it was expressed on the paratypes that we examined, it is an artifact—the collapsed recess for the premaxilla.

*Australoheros macaensis* was described based on 24 specimens, 24.7–73.7 mm SL, from two localities in the coastal Rio Macaé basin, north of Silva Jardim. Distinguishing from *A*. *ipatinguensis*, *A*. *autrani*, and *A*. *muriae* the original diagnosis refers to ‘head with a depression near the snout in individuals above 30.0 mm SL’, vs absence of such depression. This character state, shared only with *A*. *saquarema*, is referable to collapse of the premaxillary recess in preserved specimens. It does not show on fig. 9, of a specimen 65.1 mm SL. In the diagnosis of *A*. *capixaba*, it is stated that *A*. *capixaba* differs from *A*. *macaensis* and *A*. *saquarema* in ‘not having a detached snout, with a depression on the snout (vs. detached snout, with depression on head in specimens above 30.0 mm SL)’. There is no further reference to ‘detached snout’.

*Australoheros macaensis* is stated to be different from *A*. *saquarema* in fewer proximal radials on anal-fin base (13–14 vs 14–15) but this is not discriminating; from *A*. *ipatinguensis* and *A*. *muriae* by the number of dorsal-fin spines (16 vs 15), but even if valid for the modal values, dorsal-fin spine counts vary in these nominal species, including 15 spines in a paratype of *A*. *macaensis* (MCP 42403), and 16 in a paratype of *A*. *ipatinguensis* (MCP 42369); from *A*. *muriae* and *A*. *autrani* by the number of soft rays in the anal fin (8–9 vs 9–10, but this is not discriminating; from *A*. *autrani*, *A*. *ipatinguensis*, and *A*. *saquarema* by 11 rib pairs vs 10, but the number of ribs should match the number of precaudal vertebrae minus 3, and all are stated to have 14 precaudal vertebrae, corresponding to 13 precaudal vertebrae as we count them. Aside from the rib count, there seems to be no character separating *A*. *macaensis* from *A*. *saquarema*, which in turn is shown here to be identical to *A*. *autrani*.

The description of *Australoheros capixaba* was based on 29 specimens, 29.0–101.8 mm SL, from the Rio Itaúnas, Rio São Mateus, and Rio Doce in Espírito Santo. The holotype is figured (fig. 1), apparently in a photo tank before preservation. The outlines of the dorsal, caudal, and anal fins have been retouched using image manipulation software. The original diagnosis distinguishes *A*. *capixaba* from *A*. *saquarema*, *A*. *muriae*, *and A*. *ipatinguensis* by longer caudal peduncle (9.6–11.4% SL vs 6.6–7.9 combined in the other three nominal species.

*Australoheros autrani* has similar caudal-peduncle length ratio (10.2–11.9%SL) [6], and *A*. *perdi* is reported with even longer caudal peduncle (9.3–11.6%) [10]. *A*. *capixaba* is further distinguished from *A*. *autrani*, *A*. *ipatinguensis*, *A*. *muriae*, and *A*. *saquar*ema by 8 soft anal-fin rays (9–10 in the others. We find 8–10 soft anal-fin rays in our material from Rio Itaúnas and Rio São Mateus, and specimens from the Rio Muriaé with down to 8. *Australoheros capixaba* is further distinguished from *A*. *macaensis* and *A*. *saquarema* by large red spots on the dorsal portion of the trunk (vs absent); and from *A*. *autrani*, *A*. *macaensis* and *A*. *saquarema* by having reddish chest (vs chest not reddish). Large red spots are absent from the image of the holotype (fig. 19). Most or all species of *Australoheros* have an indistinct layer of erythrophores on yellow, green, or blue background colour; this is probably the case here as well. As formulated, the description of the colour characteristics seems to distinguish *A*. *autrani*, *A*. *macaensis*, and *A*. *saquarema* from the rest of *Australoheros*.

No specimens were available from the type locality of *A*. *perdi*, but the locality and description suggest that this name was based specimens of *A*. *ipatinguensis—*as it appears from the figure, in a very poor state of preservation. The diagnosis makes various comparisons with other species. Foremost is the vertebral number 13+12, the total of 25 stated to be unique for the genus, which it is not (cf. Table 3). The count of 13+12 vertebrae is uncommon in *Australoheros*, however. Because Ottoni and collaborators count vertebrae in a way different from us, however, their count possibly equals our 12+13, which is very rare in the genus (Table 3). *Australoheros perdi* is said to differ from a series of other species by having ‘head with depression’. On fig. 2 it looks like the premaxillary recess has collapsed in the upper specimen, but there is no obvious ‘depression’ in the front of the lower specimen. The sentence with this statement may also be incomplete or partly duplicated: ‘*Australoheros perdi* differs from *A*. *autrani*, *A*. *barbosae*, *A*. *capixaba*, *A*. *ipatinguensis*, *A*. *macacuensis*, *A*. *macaensis*, *A*. *muriae*, *A*. *paraibae*, *A*. *robustus*, *A*. *ribeirae and A*. *saquarema*; and *from A*. *autrani*, *A*. *barbosae*, *A*. *capixaba*, *A*. *ipatinguensis*, *A*. *macacuensis*, *A*. *muriae*, *A*. *paraibae*, *A*. *robustus*, *A*. *ribeirae* and *A*. *ribeirae* by having head with depression in the region above the eyes (vs. head without depression).’ *Australoheros perdi* is also stated to be different from *A*. *barbosae*, *A*. *muriae*, *A*. *autrani*, *A*. *capixaba*, *A*. *macaensis* in having 11–13 vs. 14–15 pectoral-fin rays. Ottoni et al.’s table 2 gives frequencies for the pectoral fin count: 11 (2), 12 (15), 13 (3). We counted 13 pectoral-fin rays in 4 specimens from the Rio Macacu. In the total material of *A*. *ipatinguensis*, 13 was the most common count, 12 in 10 specimens, 11 in one, and 14 in three; *A*. *oblongus* had almost exclusively 13; one with 12,10 with 14. Thus, the count in *A*. *perdi* matches the frequency in *A*. *ipatinguensis* and *A*. *oblongus*. *Australoheros perdi* is stated to differ from *A*. *robustus* by 14–16 dorsal spines vs 17. Our *A*. *robustus* have (in *A*. *oblongus*), 16–17 dorsal-fin spines. The commonest number of dorsal-fin spines in *Australoheros* was 16 followed by 15 and 17, and thus 14 was uncommon (Table 6). The range given in Ottoni et al.’s table 2 shows, however, a low frequency for 14 dorsal-fin spines [(14 (2), 15 (16), 16 (2)]. Proportional measurements were not discriminating, as demonstrated in Table 21, No colour characters were included in the diagnosis. Whereas the sample of *Australoheros* from Lagoa Gambazinho (91 specimens, including some heads and many juveniles), may represent a distinct species indicated by lower vertebral counts, low pectoral-fin count (Table 21) all data are compatible with an expanded concept of *A*. *ipatinguensis*.

*Material examined*. All from Brazil. Not assigned to nominal species: MNRJ 39366, 3, 69.2–99.1 mm SL; Rio de Janeiro: São José da Barra: Barra do Açu, 21°51’2’’S 41°0’2’’W; F.Pupo and I.Verissimo, 8 Jun 2009.—MCP 17849, 1,73.6 mm SL; Minas Gerais: Teófilo Otoni: Rio Santana (tributary of the Rio Mucuri), on road Teófilo Otoni to Carlos Chagas (BR-418) at about 22 km E of Teófilo Otoni,17°50’39.0"S 41°20’54.0"W; R.E. Reis, S.A. Schaefer, E.H.L. Pereira, J.F. Pezzi, 19 Jan 1995.—MCP 18140, 1, 31.1–46.9 mm SL; Minas Gerais: Teófilo Otoni: Ribeirão da Areia, tributary of Rio Mucuri, on road from Poté to Ladainha, 17°42’34.0"S 41°47’35.0"W; W.G. Saul, J.C.Garavello and A.S. Santos, 19 Jan 1995.—MCP 17840, 2, 19.7–44.5 mm SL; Espírito Santo: Rio Novo do Sul: Rio Novo do Sul, tributary of the Rio Moá, on road BR-101 little south of Rio Novo do Sul, 20°52’33’’S, 40°57’50’’W.; R.E. Reis, W.G. Saul and E.H.L. Pereira, 22 Jan 1995.—MCP 36955, 2, 21.8–22.2 mm SL; Espírito Santo: Linhares: Rio Ipiranga basin: Rio Ipiranga in Pontal do Ipiranga,19°12’1’’S, 39°43’22’’W; J.F.Pezzi, 8 Oct 2004.—MZUSP uncat., 9, 19.0–46.4 mm SL; Espírito Santo: Linhares: Rio Doce basin: Lagoa Nova; [19°31’08.4"S 39°47’07.2"W]; H.A. Britzki and I.A. Dias, 3 Feb 1965.—MZUSP 16178, 9, 31.0–81.7 mm SL; Rio de Janeiro: Rio Paraíba do Sul at São João da Barra Collector and date not recorded.[approx. 21°51’00"S 41°00’00"W].—UFBA 4717, 2 (of 5), 40.9–59.7 mm SL; Bahia: Eunápolis: Rio Buranhém, in village Colônia, near Ilhas; R. Burger and J.A. Reis, 24 Oct 2008.—UFBA 4735, 2 (of 5), 50.7–60.6 mm SL; Bahia: Eunápolis, Rio Buranhém in Eunápolis; R. Burger and J.A. Reis, 24 Oct 2008.—UFBA 4944, 2 (of 4), 45.5–61.6 mm SL; Bahia: Eunápolis: Rio Buranhém, near village Colônia, 16°09’14.8"S 39°21’08.3"W, 140 mASL; A.M. Zanata, R. Burger, A.B.A. Góes, T.A. Carvalho, 26 Feb 2009.—MCP 44943, 1, 83.3 mm SL; Espírito Santo: Domingos Martins: Rio Jucu basin: stream on road between Domingos Martins and Melgaço, tributary of Rio Jucu, 20°15’23’’S, 40°40’14’’W.; R.E. Reis et al., 24 Jan 2010.—MCP 36657, 5, 24.6–61.4 mm SL; Rio de Janeiro: São Francisco de Itabapoana: Rio Guaxindiba on Fazenda Santa Cruz, near Maniva, 21°22’58’’S 41°10’58’’W; J.F. Pezzi, 11 Sep 2004.

Assigned to nominal species: [*Australoheros autrani*]: MCP 42364, 2 paratypes, 47.3–52.1 mm SL; Rio de Janeiro: Silva Jardim: Rio Águas Claras, tributary of Rio São João, 29 km N of Silva Jardim, 22°40’00’’S 42°22’00’’W; F. Autran, M. Landim, C. Moreira and A. Vianna, 30 Aug 1997.—UFRGS 18879, 3, 17.1–53.4 mm SL; Rio de Janeiro: Silva Jardim, ditch on road between Gaviões and Japuíba, 22°34’33.0"S 42°33’39.0"W; P.C. Silva, 1 Jan 2014. Near type locality of *A*. *autrani*.*—*[*Australoheros muriae*]: MCP 42584, 1, paratype, 53.2 mm SL; Minas Gerais: Itaperuna: Rio Paraíba do Sul basin, Rio Muriaé near crossing BR 256/RJ186 (acampamento Restaurante Barra Vento), 21°15’00’’S 41°45’00’’W; D.F. Moraes and J.D.H. Halboth, 23 Jan 1990.—MHNG 2514.85, 3, 41.4–62.9 mm SL; Rio de Janeiro: Itaperuna: Rio Muriaé on road BR 393, 17 km south of Itaperuna [21°16’23.0"S 41°47’00.1"W]; W. Costa, M. Melgaço, R. Mazzoni-Buchas and C. Weber, 11 Dec 1990.—MNRJ 47291, 1, 66.4 mm SL; Rio de Janeiro: São José de Uba: Rio Paraíba do Sul basin, Córrego São Domingos at bridge of RJ-198, 200 m from crossing with BR-393, 21°18’56’’S 41°51’58’’W; D.F. Moraes, Jr., E.B. Neuhaus and V. Brito, 10 May 2016.—MNRJ 47307, 82, 10.0–97.8 mm SL; Rio de Janeiro: Itaperuna, bridge over the Córrego São Domingos, on road toward Fazenda São Domingos, 21°15’44’S 41°47’47’’W; D.F. Moraes, Jr., E.B. Neuhaus and V. Brito, 10 May 2016. [Topotypical specimens, 4 measured, 60.8–97.8 mm SL.]—

[*Australoheros saquarema*]: MCP 42366, 2 paratypes, 45.3–48.0 mm SL; Rio de Janeiro: Saquarema: Córrego Buracão, tributary of Rio Mato Grosso, 22°52’24’’S 42°36’22’’W; W. Costa, L. Villa Verde, F.P. Ottoni, J.L. Mattos and E. Mattos, 9 Oct 2005.—[*Australoheros macaensis*]:—MCP 20195, 1, 84.7 mm SL; Rio de Janeiro: Macaé: Rio do Salto, about 14 km NW of Rio Dourado on BR101, tributary of the Rio Macaé, 22°23’11’’S 42°07’19’’W; J.F. Pezzi, E.H.L. Pereira and J. Montoya, 20 Jan 1997.—MCP 42403, 2 paratypes of *A*. *macaensis*, 30.9–40.4 mm SL; Rio de Janeiro: Macaé: Reserva União, 22°25’35’’S 42°02’21’’W; F.P. Ottoni, A. Barbosa and J. Mattos, 29 Oct 2007.—MNRJ 24226, 3, 30.6–49.0 mm SL. Rio de Janeiro: Macaé: Parque Nacional Restinga de Jurubatiba, Lagoa de Carapebus, [approx. 22°14’ 50’’S, 41°36 20’’W]; P.H. Carvalho, J.I.S. Botero and J.F. Caluca, 7 Aug 2001.—MNRJ 47279, 3, 59.2–61.7 mm SL; Rio de Janeiro, Macaé: Rio dos Quarenta, under bridge on BR-101, about 500 m from the interchange with RJ-106 highway, 22°13’6’’S 41°45’32’’W; P.A. Buckup, M.R. Britto, C.R. Moreira, 29 Apr 2016. Topotypical specimens of *A*. *macaensis*.—NPM 5307, 2, 23.4–35.4 mm SL; Rio de Janeiro: Macaé: Parque Nacional Restinga de Jurubatiba Lagoa Cabiunas, Lagoa de Jurubatiba, 22°18’05"S, 41°41’36"W; A.C. Petry, 12 Aug 2015..—UFRGS 18911, 1, 53.2 mm SL; Rio de Janeiro: Macaé: pool on country road between Barra de Macaé and Carapebus, 22°13’38.0"S 41°41’07.0"W; P.C. Silva, U. Santos, A. Hirschmann, A. Thomaz and T.P Carvalho, 1 November 2014.—[*Australoheros capixaba*]: Rio Itaúnas basin: MCP 18141, 6 paratypes, 10.5–49.2 mm SL; Espírito Santo: Montanha: stream tributary of the Rio Itaúnas crossing road from Nanuque to Montanha about 5 km south of Nanuque; 17°57’46’S, 40°23’46’’W; R.E. Reis, W.G. Saul and E.H.L. Pereira, 26 Jan 1995.—CZNC 1099, 4 (of 6), 134–85.5 mm SL. Espírito Santo: Pedro Canário: Cristal do Norte: Rio Itaúnas, above and below the dirt road at the foot of the Córrego Limoeiro, 18°12’46"S 40°54’49"W; L.F.S. Ingenito et al., 10 Jun 2015.—UFBA 4995, 1, 60.5 mm SL; Espírito Santo: Pedro Canário: Rio da Samambaia, on BR 10, 18°07’20.6"S 39°33’20.2"W, 28 mASL; A.M. Zanata, R. Burger, A.B.A. Góes and T.A. Carvalho, 27 Feb 2009.—Rio São Mateus basin: MCP 18139, 6, paratypes, 9.6–64.9 mm SL; Espírito Santo: Nova Venécia: Rio São Mateus basin: Rio Cricaré, about 1 km above Nova Venécia, R.E. Reis, W.G. Saul and E.H.L. Pereira, 26 Jan 1995. 18°42’2’’S, 40°24’58’’W.—CZNC 87, 3, 15.3–35.5 mm SL; Espírito Santo: São Mateus: Rio São Mateus basin: Lago do Córrego Canivete, tributary of the Córrego Grande, tributary of the Rio São Mateus, within CEUNES/UFES, at ES-422, 18°40’8"S 39°50’49"W; F.M. Chagas et al., 17 Nov 2011.—CZNC 954, 1, 28.3 mm SL; Espírito Santo: São Mateus: Córrego Canivete (tributary of the Córrego Grande, tributary of the Rio São Mateus), within CEUNES/UFES, Bairro Litoraneo, 18°4016’’S 39°51’25"W; L.F.S. Ingenito et al., 23 Apr 2014.

CZNC 994, 3, 15.7–47.6 mm SL; Espírito Santo: São Mateus: Córrego Canivete (tributary of the Córrego Grande, tributary of the Rio São Mateus), within CEUNES/UFES, Bairro Litoraneo, 18°40’16’’S 39°51’25"W; S. Bitti et al., 25 Jun 2014.—CZNC 1017, 2, 18.5–19.4 mm SL; Espírito Santo: São Mateus: Córrego Canivete (tributary of the Córrego Grande, tributary of the Rio São Mateus), within CEUNES/UFES, Bairro Litoraneo, 18°40’16"S 39°51’25"W; L.F.S. Ingenito et al., 28 Jul 2014.—CZNC 1027, 1, 23.8 mm SL; Espírito Santo: São Mateus: Córrego Canivete (tributary of the Córrego Grande, tributary of the Rio São Mateus), within CEUNES/UFES, Bairro Litoraneo, 18°40’16’’S 39°51’25"W; L.F.S. Ingenito et al., 28–29 Aug 2014.

#### *Australoheros sanguineus* Ottoni, 2013

*Australoheros sanguineus* Ottoni, 2013 [12]: 161, fig. 2. (holotype MCP 14556; type locality Brazil: Santa Catarina state: arroio Lindo, tributary of the rio Cubatão, near the road SC-301, near BR-101, Pirabeiraba, Joinville municipality).

*Definition*. Based on the position in the *mt-cyb and mt-coI* trees and minimum uncorrected *p*-distance in *mt-coI* exceeding 2% from all other species of the genus, *Australoheros sanguineus* is a distinct evolutionary lineage. Specimens of *A*. *sanguineus* are similar to *A*. *acaroides*, *A*. *kaaygua*, A. *minuano*, *A*. *ricani*. and *A*. *mboapari* in presence of zipper band, but no morphological synapomorphy was registered.

**Fig 25:**
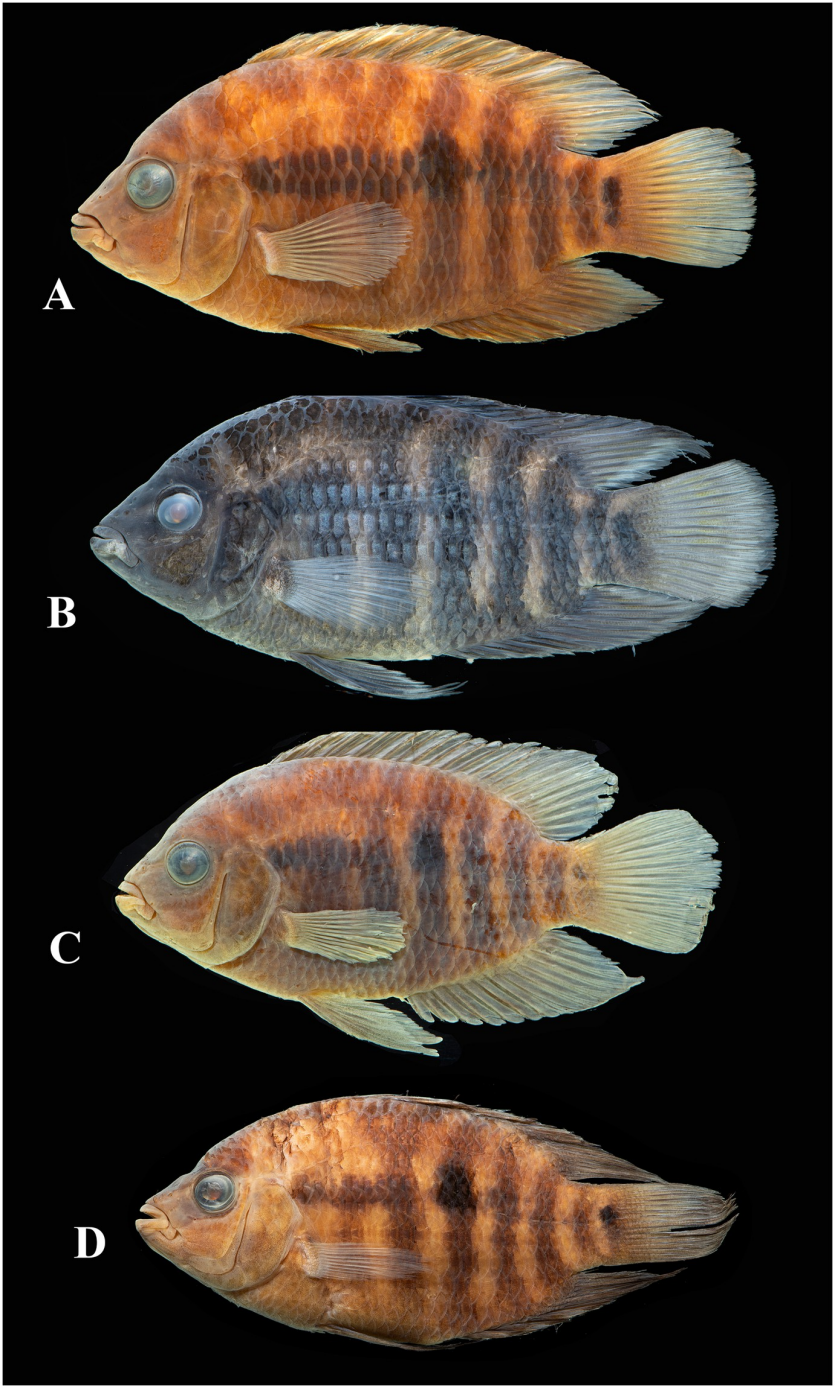
Australoheros sanguineus. **A.** holotype, adult female, 84.2 mm SL; MCP 14556; Brazil: Santa Catarina: Joinville: Rio Cubatão basin: Arroio Lindo. **B.** Adult male, 82.1 mm SL; MCP 40635; Joinville: Rio Cubatão Norte. **C.** *Australoheros* cf. *sanguineus*, adult male, 63.2 mm SL; Brazil: Santa Catarina: Petrolândia: stream tributary of the Rio Perimbo on road between BR 282 and Petrolândia (tributary of the Rio Itajai-Açu); MCP 22440. **D.** *Australoheros sanguineus*, adult female, 69.0 mm SL; NUP 8861; Brazil: Paraná: Palmas: border with Pinhão: Rio Iratim, tributary of the Rio Iguaçu.

*Description of sample*. Meristic data are given in Tables 3–9, proportional measurements in Table 16. Fig 25 shows general appearance of adults.

Moderately elongate to deep-bodied, laterally compressed. Sexes isomorphic except vertical and pelvic fins slightly longer in some large males than in large females. Predorsal contour straight ascending or with slight indentation anterior to orbits, curved anterior to dorsal fin origin. Nuchal elevation absent in both sexes. Dorsal profile curved, slightly descending to end of dorsal-fin base. Caudal peduncle slightly tapering, contours straight. Prepelvic contour slightly curved, straight, or slightly concave anteriorly, convex posteriorly. Abdominal contour straight or slightly convex. Anal-fin base contour straight ascending. Head short, laterally compressed. Snout short, blunt, subtriangular in lateral aspect, rounded in dorsal aspect. Mouth terminal, forward directed, close to horizontal line from lower margin of orbit or slightly more ventral in large specimens. Lips moderately thick, lip folds interrupted or subcontinuous anteriorly. Jaws equal in anterior extension; maxilla and premaxilla not reaching to vertical from anterior margin of orbit. Orbit removed from frontal contour, in middle of head length, in upper half of head. Teeth in both jaws caniniform, slightly recurved, in outer row slender, increasing in size toward symphysis. Bicuspid teeth not observed. Teeth in outer hemiseries in upper/lower jaw 9–16/10–17. Inner teeth in two rows. Gill rakers externally on first gill-arch 2 epibranchial, one in angle and 6–7 ceratobranchial. Microbranchiospines present externally on 2nd to 4th gill-arch.

Dorsal-fin spines increasing in length to about 6th or 7th, from which subequal, last spine longest, soft dorsal-fin rounded in young, in adults with broad pointed tip, third, fourth or fifth soft fin-ray longest, reaching to about middle or 4/5 of caudal fin. Soft anal fin rounded in young, in adults with a short point formed by fourth soft fin-ray, reaching to about middle or 4/5 of caudal fin. Pectoral fin rounded, 4th or 5th ray longest, reaching to vertical from anal orifice or first anal-fin spine. Pelvic fin pointed, first soft ray longest, reaching to base of third anal-fin spine (broken in holotype). Caudal fin rounded.

Scales on body finely ctenoid. Predorsal midline scales about 10–13, slightly smaller than flank scales, covered by skin, only part with exposed margin, irregularly arranged, mixed cycloid and weakly ctenoid. Chest scales ctenoid, almost size of flank scales. Cheek scales cycloid, in 3–5 series, covered by skin. Opercular scales cycloid, covered by skin. Between first upper lateral line scale and dorsal-fin origin 3 large scales or 3 large and one small scale. Lateral line scales absent from caudal fin, except in one specimen (NRM 30953 73.6 mm SL), with two tubed scales in ventral lobe. One large and one small or two small scales separating last scale of upper lateral line from dorsal-fin base. Prepelvic scales anteriorly small, cycloid and without free margin, remaining scales only slightly smaller than flank scales, weakly ctenoid and with free margin. Fin scales ctenoid. Row of minute scales along dorsal-fin base from spine IX or XI, basal squamation gradually expanded, interradial scales in single series, from spine XII–XVI, on soft fin up to 5 interradial scales, scales absent from last one or two interradial membranes. Soft anal fin with narrow basal scale layer, interradial scales from behind 6th spine, up to 6 scales in interradial row.

*Colouration in preservative*. Adults, about 50 mm and larger with ground colour beige, fawn, or pale grey. Bars 1(a, b) to 6 present, dark brown, 2–3 scales wide; bar 6 distinct or obsolete absent below lateral band; interbars 2–3 scales wide, except Interbar 1 usually narrower than one scale width; lateral band extending from gill cleft caudad to Interbar 4 composed of dark spots on scales in rows 0 and E1, forming a pattern reminding of a zipper; or band mainly on E1 row, with irregular margins; dark scale spots on posterior scales in E1 row may extend band to include Bar 2; Bar 5 terminating dorsally in lateral band or extending dorsally at most to dorsal-fin base. Bar 5 entire Bars generally vertical, dorsal portions of bars 2–4 usually slightly caudally inclined. Dorsal portions of any combination of Bars 4 and 5 or Bars 5 and 6 commonly forming a Y mark with posterior branch distinctly paler than anterior branch. Dorsal portions of Bars 5 and 6 otherwise expressed as independent irregular light brown blotches. Dorsal portions of bars 7 and 8 present, expressed as short rostrad inclined light brown or grey bars crossing anterior dorsal-fin base and occiput, respectively. Midlateral black or dark brown blotch present in Bar 4 on E1 scales and parts of adjacent scales barely exceeding bar 4 in width; corresponding portions of bars 2 and 3 may be slightly widened or more intensely pigmented but distinct spots absent from bars 2 and 3. Caudal spot (Bar1b) indistinct, or distinct, light or dark brown, darker on upper lobe, but extending as a dark vertical bar across middle of the caudal-fin base. Brown spot close to margin of each scale below level of midlateral band forming indistinct narrow lines on abdominal side, gradually weaker and disappearing ventrad. Black or dark brown blotch usually present at anterior end of horizontal band and at upper base of pectoral fin. Dorsal fin translucent or pale grey, with grey, or dark grey blotches at interradial bases, continuous with or slightly separate from bars 2 to 6 and/or 7. Anal and caudal fins translucent, pale grey. Pelvic fin grey or brown, paler medially.

*Geographical distribution* (Fig 1). Rio Cubatão and Rio Pirai basins; headwaters of the Rio Iguaçu; tentatively in the Rio Itajai-Açu and Rio Sambaqui basins.

*Comments*. *Australoheros sanguineus* was described based on the holotype and 10 paratypes, 57.9–105.3 mm SL according to Ottoni [12], all from the Rio Cubatão basin near Joinville. The diagnosis is based on character states in combination, including colour pattern, fin-ray counts, proportional measurements, frontal contour, number of proximal radials, extent of scale row at dorsal-fin base. The only potential synapomorphy is the presence of a ‘blood red’ spot at the dorsal and ventral corners of the caudal fin, distinguishing from species in which only reddish pigment is present, red pigment is absent, or when a ‘complete bar’ of red pigment is present along the posterior margin of the caudal fin. The description was based on specimens preserved in 1908, 1974 and 1985, and no voucher is given for live colours. The he colour description on p. 165 matches the photograph, fig. 1 of a living specimen without locality information. It is consequently not known if the type series conforms to the diagnosis regarding live colours. As far as can be ascertained, the photo was first published in small size in an article about aquarium fish in the area of Joinville [109]. *Heros facetus* was mentioned in the article, but there was no indication that the photo would be related to the observations in the article; more likely the several vignette photos on p. 31 were provided by the editor of the journal. Several species of *Australoheros*, including, apparently *A*. *facetus* as illustrated by (Calviño [62], fig. 3), also have red blotches at the corners of the caudal fin, and there seems to be no information on variation in intensity of this red colour neither in *Australoheros* from the lower Rio Cubatão nor from elsewhere. The specimen illustrated by Ottoni [12], fig. 1 seems indistinguishable from *Australoheros acaroides*. As explained under *A*. *acaroides*, Ottoni [12], fig. 3, used a photo of *A*. *facetus* to illustrate live colouration in *A*. *acaroides*.

The original description [12] characterises *Australoheros sanguineus as* having 14 pectoral-fin rays, but the holotype and other specimens from Joinville had 12 or 13 as counted by us. The caudal spot is said to be diagnostically round, but, as apparent already from the photograph of the holotype [12], fig. 2), it has a caudal blotch in the shape of a vertical midbasal bar. Other characters listed In the original description refer to absence of metallic blotches on the anal-fin base, the number of proximal dorsal- and anal-fin radials, anal-fin (soft) rays, pleural ribs, as well as various proportional measurements, and more colour characters in living specimens, referring to figs 1 and 3. The absence of metallic blotches was confirmed in our specimens from Joinville. The number of proximal radials, i.e. the pterygiophore portion that extends down between the neurapophyses or haemapophyses, is probably correlated with the number of fin rays, but in both the dorsal and anal fin, the proximal radial of the last ray may be absent or very reduced. On the X-radiograph (fig. 5), there seems to be 25 dorsal proximal radials as stated [12], but then there are two associated with the first dorsal-fin spine, possibly indicating absence of an anterior dorsal-fin spine. The first two anal-fin spines share proximal radial, so there are 13 proximal radials in the anal fin, as also stated [12]. There seems to be 11 pleural ribs on fig. 5, as stated [12], but this number was not unique within the genus. Also, the count of 9 anal-fin soft rays was common in the genus and present in all or most of our specimens from Joinville. The basal dorsal-fin squamation started after the 8th spine in the holotype, not after the 11th as stated in the original description [12] (but after the 10th or 11th in NRM 30953), and the basal anal-fin squamation in the holotype started after the first spine (4th or 5th in NRM 30953), not the 7th as stated [12], and interradials appeared after the 4th spine.

With regard to proportional measurements, the supposed differences are applied in comparison with specimens of a wide range of sizes, and does not account for allometry. They may also be flawed because the standard length of the holotype was apparently taken to the posterior margin of the caudal spot instead of the base of the caudal fin (end of the hypural plate). The holotype was 84.2 mm in standard length, not 88.1 mm as stated in the original description [12]. This means that, e.g. the caudal peduncle is not longer (9.8–10% of SL) in the type series than in a range of species with a combined interval of 5.1–9.2% of SL according to Ottoni [12], but at least the holotype is contained in that interval with 8.9% of SL.

Our comparison of measurements of specimens from Joinville with other *Australoheros* suggested that the proportional measurements were in line with other coastal *Australoheros*. The conclusion is that there was no character or character combination in the original diagnosis by which to recognise specimens of *Australoheros* from Joinville as a separate species of *Australoheros*. Mitochondrial DNA, however, showed that there was a distinct species in the area, potentially sister species to *A*. *ribeirae*, and we apply the name *A*. *sanguineus* on that MOTU. The material here referred to *A*. *sanguineus* was limited, and did not enable a comprehensive description. Collecting efforts in the Rio Lindo failed to find specimens of *Australoheros*, and the holotype remains the only specimen known from the type locality. Other specimens from near Joinville were from the northern branch of the Rio Cubatão, and from the Rio Pirai, south of Joinville.

Specimens of *Australoheros* from the Rio Itajai-Açu basin (Fig 25C) and Morretes may represent *A*. *sanguineus*, but the absence of strong morphological discriminating data and DNA data prevented a conclusive determination.

The *mt-cyb* sequences from LBP 1050 (Papanduva, Rio Negro basin), and NRM 49554 (Rio Chopim; as *A angiru* in Říčan et al. [13]), both from the upper Rio Iguaçu basin, were identical to sequences from the coastal streams near Joinville, representing *A*. *sanguineus* (MCP 40635; MK414416-414418). The upper Iguaçu samples were more similar in body shape and colour pattern to coastal *A*. *sanguineus* than to *A*. *angiru*, being relatively deep-bodied and having a more or less distinct zipper style lateral band, but lacking the dark spots on the side characterising *A*. *angiru*. Apparently, *A*. *sanguineus* is distributed in both the interior Paraná River basin as well as in coastal Atlantic streams. The voucher specimen for our *mt-co1* sequence LBP 1050 was identified by the provider as *A*. *kaaygua*, probably based on the locality.

Říčan et al. [13] described *A*. *angiru* on the basis 27 specimens from the upper Rio Uruguay in Santa Catarina, previously identified as *A*. *kaaygua* by Říčan and Kullander [3, 4], but listed also four non-type specimens from Palmas and Pinhão, in the Rio Iguaçu basin. Considerable weight was given also to aquarium specimens reported by Staeck [112] from the Iguaçu and upper Uruguay basin. The only specimens available from the Iguaçu basin, however, are the four specimens NUP 7743, 3683, 3697, and 8861, which we identified as *A*. *sanguineus*, and which were in a very bad state of conservation, two of them eviscerated, and one without caudal fin.

Říčan and Kullander [3], and Říčan et al. [13] listed a specimen NRM 49554, tissue number 1179, GenBank Accession number for *mt-cyb* AY998658, with data suggesting that it came from the Rio Iguaçu. This specimen/tissue sample was recorded later as lost from the NRM collection. The specimen was identified as ‘*A*. jacutinga’ by Říčan and Kullander [3] and as *A*. *angiru* by Říčan et al. [13]. The *mt-cyb* sequence grouped it with *A*. *sanguineus* (Fig 13), suggesting that it represents the Iguaçu population of *A*. *sanguineus*.

Staeck [110] published a short note reporting on a species of ‘*Cichlasoma*’ related to ‘*C*. *facetum’* that he and party collected in the Rio do Peixe in 1995, and subsequently propagated in the German aquarium hobby. The note is illustrated with aquarium images of a male and female. Staeck ([111] reported on cichlids related to *‘C*.’ *facetum* collected in 1995 in Rio Chopim and Rio do Peixe, which he obviously considered to be of the same species. The article is illustrated with an image of the same specimen shown by Staeck [110], but also breeding male and territorial male from the Rio Chopim. Říčan et al. ([13]: fig. 7,) republished the territorial male image as ‘male in neutral phase’; a different angle of the breeding male; and a new image with a breeding female. All those images were identified by Říčan et al. [13] as *A*. *angiru*). Because Staeck considered the *Australoheros* collected in Rio Chopim and Rio do Peixe to be the same species, it is most likely that the aquarium population mentioned by Staeck, and source of the NRM 49554 specimen, may have consisted only of the Chopim species; less likely that the two species hybridised in captive breeding—or that it represents an aquarium import of coastal *Australoheros sanguineus* unrelated to Staeck’s observations.

We identified the four NUP specimens assigned by Říčan et al. [13] to A. *angiru* as specimens of *A*. *sanguineus* in a poor state of conservation (Fig 25D).

Consequently, as far as known, *Australoheros angiru* is endemic to the upper Rio Uruguay. Staeck’s specimens from the Rio Chopim and the NUP specimens were similar to other *A*. *sanguineus* in colour pattern and deep body. In a pictorial guide to the fishes of the Rio Iguaçu, Baumgartner et al. [112] published an image of a specimen similar to the preserved NUP specimens, but with deformed caudal peduncle and caudal fin. Paiz et al. [113] studied karyotypes of specimens identified as *A*. *angiru* collected in São Lourenço do Oeste, Santa Catarina, in isolated lagoons not connected to the Iguaçu or Uruguay rivers. This study was uninformative concerning the systematic position of *A*. *angiru* as the issue was not addressed. In passing, however, Paiz et al. mentioned another record of *A*. *angiru* from the Rio Guarani (about 25.51667°S 53.15°W), in the lower Rio Iguaçu basin, which may as well rather represent *A*. *sanguineus* or A. *kaaygua*, It seems thus that the population of *A*. *sanguineus* in the upper Rio Iguaçu basin may have gone undetected. Available data on *Australoheros kaaygua*, the other species of the genus recorded from the Iguaçu basin is limited. The colour pattern places it with the southern species with a distinct zipper type lateral band; the only sequence available (13)

Does not support identity with *Australoheros sanguineus*.

*Specimens examined*. All from Brazil. Atlantic versant: MCP 14556, 1, holotype, adult female, 84.2 mm SL; Santa Catarina: Joinville: Rio Cubatão basin: Arroio Lindo, tributary of the Rio Pirabeiraba, on side of road SC-301 close to BR-101, 26°13’0"S 48°54’W; C.A.S. Lucena, L.R. Malabarba and R.E. Reis, 19 Sep 1985.—MCP 6912, 2 paratypes, 54.7–60.1 mm SL; Rio Cubatão (norte), close to road BR-101, 26°12’0"S 48°55’0"W; C.A.S. Lucena, L.R. Malabarba and R.E. Reis, 19 Sep 1985.—MCP 40635. 1, 82.1 mm SL; Joinville: Rio Cubatão Norte on road Quiriri de Baixo, 26°8’33.0’’S, 48°59’45.0’’W; 7 Oct 2016, J. Pezzi da Silva and E. H.L. Pereira.—NRM 30953, 2, 73.6–84.1 mm SL; Joinville: Rio Pirai basin, 18 km SE Joinville, 26°30’00.2"S 48°43’58.9"W; K.H. Lüling, 20 Oct 1980.—Rio Iguaçu basin: LBP 1050, 5, 40.2–56.9 mm SL; Santa Catarina: Papanduva: Rio Iguaçu basin; tributary of Rio São João, 26°22.049’S 50°07.149’W; C. Oliveira et al., 12 Jan 2001.—MCP 13732, 5 18.7–56.9 mm SL; Santa Catarina: Mafra: Rio São Lourenço in Cabo de São Lourenço, at 12 km from Mafra; tributary of the Rio Negro, tributary of the Rio Iguaçu, 26°09’00.0"S 49°53’00.0"W; P. Azevedo, A. Bergmann, E.H.L. Pereira and L. Amato, 5 May 1989.—NUP 3967, 59.6, mm SL; Paraná: Pinhão: Rio São Pedro, tributary of the Rio Iguaçu, 26°S, 51°45’W. NUPELIA staff, 29 Mar 1993.—NUP 7743, 67.1 mm SL; Paraná: Palmas, border with Pinhão: Rio Iratim, tributary of the Rio Iguaçu, 26°5’S, 51°35’W. Nupelia staff, 25 Apr 1993.—NUP 8861, 70.4 mm SL; Paraná: Pinhão: Rio São Pedro, tributary of the Rio Iguaçu, 26°S, 51°45’W. Nupélia staff, 27 Apr 1993.—NUP 3683, 1, 71.8 mm SL; Paraná: Pinhão: Rio São Pedro, tributary of the Rio Iguaçu, 26°S, 51°45’W; Nupélia staff, 28 Mar 1993.

*Australoheros* sp. cf. *A*. *sanguineus*: MCP 12234, 1, 59.3 mm SL; Paraná: Morretes: Rio Sambaqui basin, Rio Sagrado at Posto Florestal, 25°29’00.0"S 48°50’00.0"W; C.A.S. Lucena, L.R. Malabarba and A. Bergmann, 26 Jul 1988.—MCP 22440, 14, 1 measured 63.2 mm SL; Santa Catarina: Petrolândia: stream tributary of the Rio Perimbo on road between BR 282 and Petrolândia (tributary of the Rio Itajai-Açu), 27°35’41.0"S 49°44’35.0"W; R.E. Reis, A.R. Cardoso, P.A. Buckup and F. Melo, 21 Dec 1998.—NUP 10504, 1, 78.5 mm SL: Santa Catarina: Ibirama: Rio Itajai-Açu drainage: Rio Hercilio: Rio Hercilio, 27°18’56.0"S 49°32’52.0"W; GERPEL, 7 Aug 2010.—NUP 2738, 1, 67.5 mm SL; Paraná: Tijucas do Sul: Rio São João, Reservatório Vossoroca (Chaminé), 25° 49’13’’S 49°04’04’’W, Copel ichthyology staff, Nov 2001.—CAS 66934pt. 3, 24.6–40.1 mm SL. Brazil: State of Santa Catarina: Rio Iguassu basin Porto União A.L. Carvalho, 22 Apr 1944 [with 25 specimens of ‘*Geophagus’ brasiliensis*, 15.1–40.8 mm SL].

#### *Australoheros mboapari*, new species

*Definition*. Based on the unique concealed or absent cheek squamation and the combination of character states in colouration, squamation, vertebrae count shared only with *A*. *tembe*, *A*. *forquilha*, and *A*. *ricani Australoheros mboapari* is hypothesised to be a distinct evolutionary lineage. Specimens of *A*. *mbapoari* can be distinguished from all other species of *Australoheros* by scales absent from all or lower half of cheek, or present deeply embedded (vs exposed by thin skin layer or free margins and covering all of cheek). *Australoheros mbapoari* shares with *A*. *forquilha*, *A*. *ricani*, *A*. *tembe*, and *A*. *ykeregua* a row of minute scales along the base of the caudal fin reaching anteriorly to or almost to the anterior insertion of the dorsal fin (vs scale layer ending at middle or more posterior position along the dorsal-fin base); with *A*. *ricani* black soft dorsal fin in adult females (vs soft dorsal fin light). It differs from all species of *Australoheros* except for *A*. *forquilha*, *A*. *tembe*, and *A*. *ykeregua* by the absence of abbreviated portions of dorsal parts of bars 5–6 anterodorsally on the side; prepelvic scales deeply embedded (vs with free margins); caudal fin subtruncate (vs. rounded); broad pelvic-fin tip with first and second rays subequal in length (vs first ray longest (first may be longer also in *A*. *ykeregua*); from all except *A*. *forquilha*, *A*. *kaaygua*, *A*. *tembe*, and *A*. *ykeregua* by deep lachrymal bone). Stout lower pharyngeal tooth plate with packed, mainly molariform teeth (vs. tpph plate with slender teeth laterally and a few stout teeth posteromedially potentially distinguishing from congeneric species, but comparative data equivocal). Other characteristics include caudal spot absent or diminutive in adults; absence of Y-shaped bars; scales in E1 row usually 25–26 (vs. usually 23–24 in congeneric species); single abdominal bar or bar absent in adults (vs 2–3); vertebrae 13+14 = 27 (vs. 13+13, rarely any other number), absence of dark suborbital markings (vs present in *A*. *forquilha* and *ykeregua*); absence of spots in unpaired fins (vs present in *A*. *forquilha*, *A*. *scitulus* and variable in *A*. *oblongus;*); and autapomorphic cheek squamation and female breeding colour. Distinguished from *A*. *tembe* and *A*. *kaaygua* by moderately thick lips (vs hypertrophied). Lateral band of zipper type, similar to most southern congeneric species, excepting only *A*. *facetus*.

**Fig 26:**
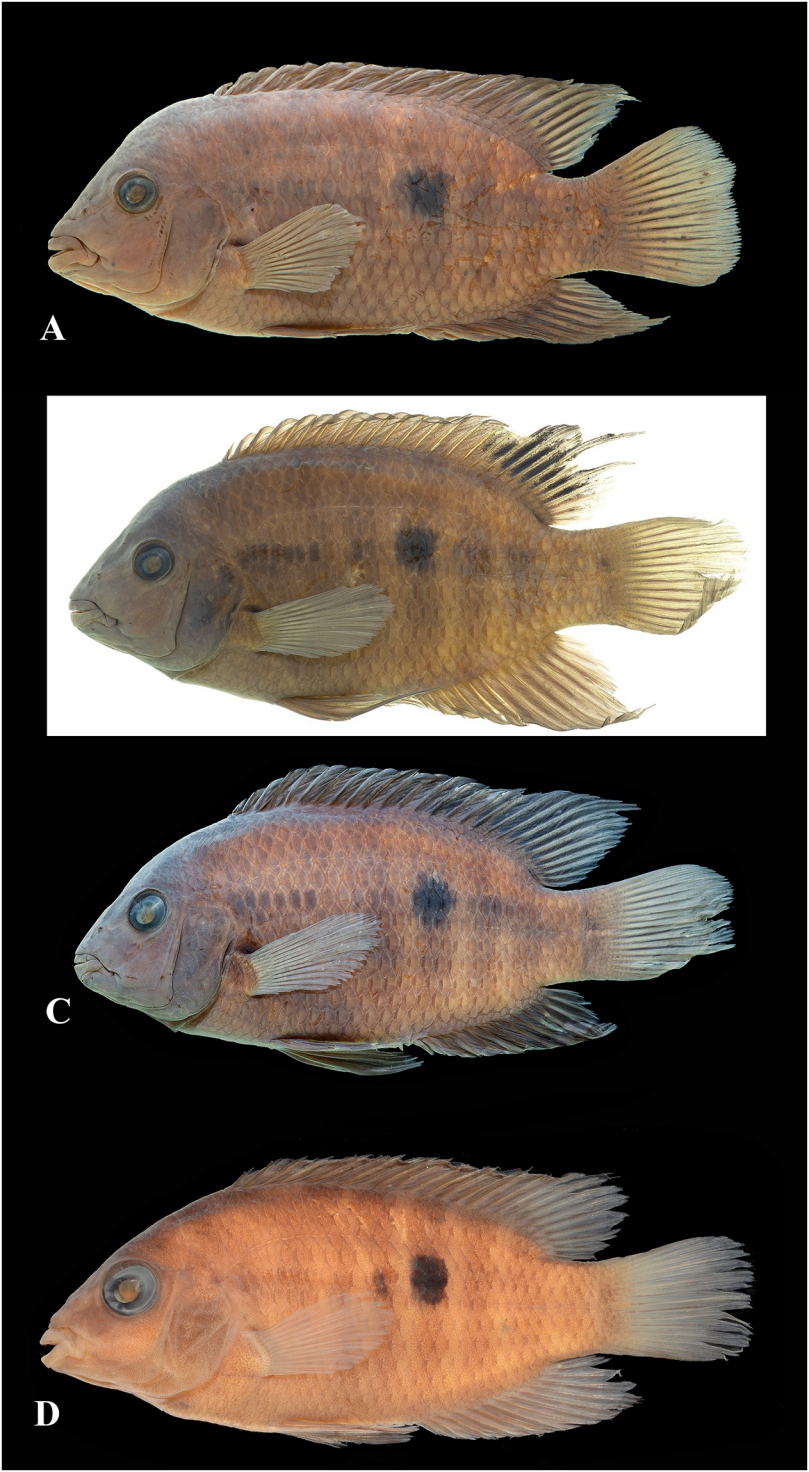
Australoheros mboapari. ***A*.** holotype, MCP 49000, adult male, 101.7 mm SL; Brazil: Rio Grande do Sul: Antônio Prado: Rio Taquarí drainage, Quaresma, close to the Rio das Antas. **B**. Paratype, adult female, 95.1 mm SL: MCP 33554; Brazil: Rio Grande do Sul: Nova Roma do Sul: Rio Taquari drainage: Arroyo do Carma, near mouth in the Rio das Antas. **C.** Paratype, young male, 81.1 mm SL: MZUSP 33554; Brazil: Rio Grande do Sul: Nova Roma do Sul: Rio Taquari drainage: Arroyo do Carma, near mouth in the Rio das Antas. **D.** paratype, juvenile, 39.3 mm SL; MCP 23044; Rio Grande do Sul: Rio Taquari drainage: Antônio Prado: Rio Toro, on the road from Vila Flores to Antônio Prado.

*Holotype*. MCP 49000, adult male, 101.7 mm SL (Fig 26A); Brazil: Rio Grande do Sul: Antônio Prado: Rio Taquarí drainage, Quaresma, close to the Rio das Antas, 28°52’46’’S 51°19’12’’W; J.D. Latini et al., Oct 2003.

*Paratypes*. All from Brazil, Rio Grande do Sul, Rio Taquari drainage: MCP 23044, 46, 17.2–90.4 mm SL; Antônio Prado: Rio Toro, on the road from Vila Flores to Antônio Prado, 28°52’19’’S 51°26’57’’W; R.E. Reis, J.F. Pezzi and E.H.L. Pereira, 21 Jan 1999.–MCP 33554, 4, 81.1–95.6 mm SL; Nova Roma do Sul: Arroio do Carma, near mouth in Rio das Antas, 28°58’93’’S 51°23’12’’W; J.D. Latini et al., 2003.–MCP 33639, 1, 97.3 mm SL; Santa Bárbara: Rio das Antas close to mouth of Rio Carreiro, 29°5’29’’S. 51°42’42’’W; J.D. Latini et al., 28 Sep 2002.–MCP 33659, 7, 65.7–84.8 mm SL; Same data as holotype.–MCP 34988, 4, 62.1–77.5 mm SL; Brazil: Antônio Prado: Rio Ituím at Cachoeira do Saltinho, tributary of the Rio Turvo, 28°37’S 51°23’W; A.R. Cardoso and V.A. Bertaco, 6 Mar 20004.–MCP 40953, 3, 56.0–97.3 mm SL; Veranópolis: Rio da Prata, tributary of the Rio das Antas, 28°58’16"S 51°27’20"W; J.D. Latini, V.A. Capatti and S. Rodrigues, Nov 2005.—MCP 44382. 2, 78.5–87.2 mm SL. Veranópolis: mouth of the Rio Pratinha, 28°56’S 51°27’59’’W; J.F. Pezzi, 14 Jan 2007.—MCP 47552, 1, 106.2 mm SL; Nova Roma do Sul: Rio da Prata, tributary of the Rio das Antas, 28°58’15’’S 51°27’20’’W; J. D. Latini et al., Oct 2011.–UFRGS 9688, 23 (3, 92.5–107.9 mm SL; 20, 21.3–36.1 mm SL); Dois Lajeados: Rio Carreiro above PCH Linha Emília, 28°56’24"S 51°46’47"W; J. Ferrer and G. Frainer, 17 Jan 2008.—UFRGS 13184. 2, 46.5–48.1 mm SL; Muitos Capões: Rio Taquari drainage: Rio Ituím at PCH Saltinho, 28°37’09"S 51°21’14"W; J. Anza and G. Frainer, 22 Mar 2010.

*Description of type series*. Meristic data area given in Tables 3–9, proportional measurements in Table 17. For general appearance, see Fig 26.

Sexes isomorphic except fins slightly longer in some large males than in large females, and genital papilla in males slender, conical, in females wider, blunt. Moderately elongate, laterally compressed. Predorsal contour straight ascending to above orbit where curved, almost horizontal slightly anterior to dorsal-fin origin; occiput posteriorly markedly compressed and with slight compressed nuchal elevation in large specimens of both sexes. Dorsal contour anteriorly horizontal, soft dorsal-fin base slightly curved and descending. Caudal peduncle contours slightly sloping. Prepelvic contour slightly curved or straight. Abdominal contour straight horizontal except for slightly convex in females. Anal-fin base slightly convex, ascending. Head short, laterally compressed. Snout short, blunt, subtriangular in lateral aspect, narrowly rounded in dorsal aspect. Mouth terminal, far removed from lower margin of orbit. Lips moderately thick, lip folds interrupted anteriorly. Jaws equal in anterior extension; maxilla and premaxilla not reaching to vertical from anterior margin of orbit. Orbit removed from frontal contour, in middle of head length, in upper half of head. Teeth in both jaws subcaniniform, erect, in outer row slender, increasing in size toward symphysis; anterior teeth frequently worn apically. Bicuspid teeth observed in one specimen, in which 2 anterior teeth in lower jaw bicuspid (MCP 33354, 82.0 mm SL). Teeth in outer hemiseries in upper/lower jaw 8–13/11–16. Inner teeth in 1–3, usually 2 rows in both jaws. Gill rakers externally on first gill-arch 1–3 epibranchial, one in angle and 5 (3), 6 (24), 7 (1) ceratobranchial. Microbranchiospines present externally on 2nd to 4th gill-arch.

**Fig 27:**
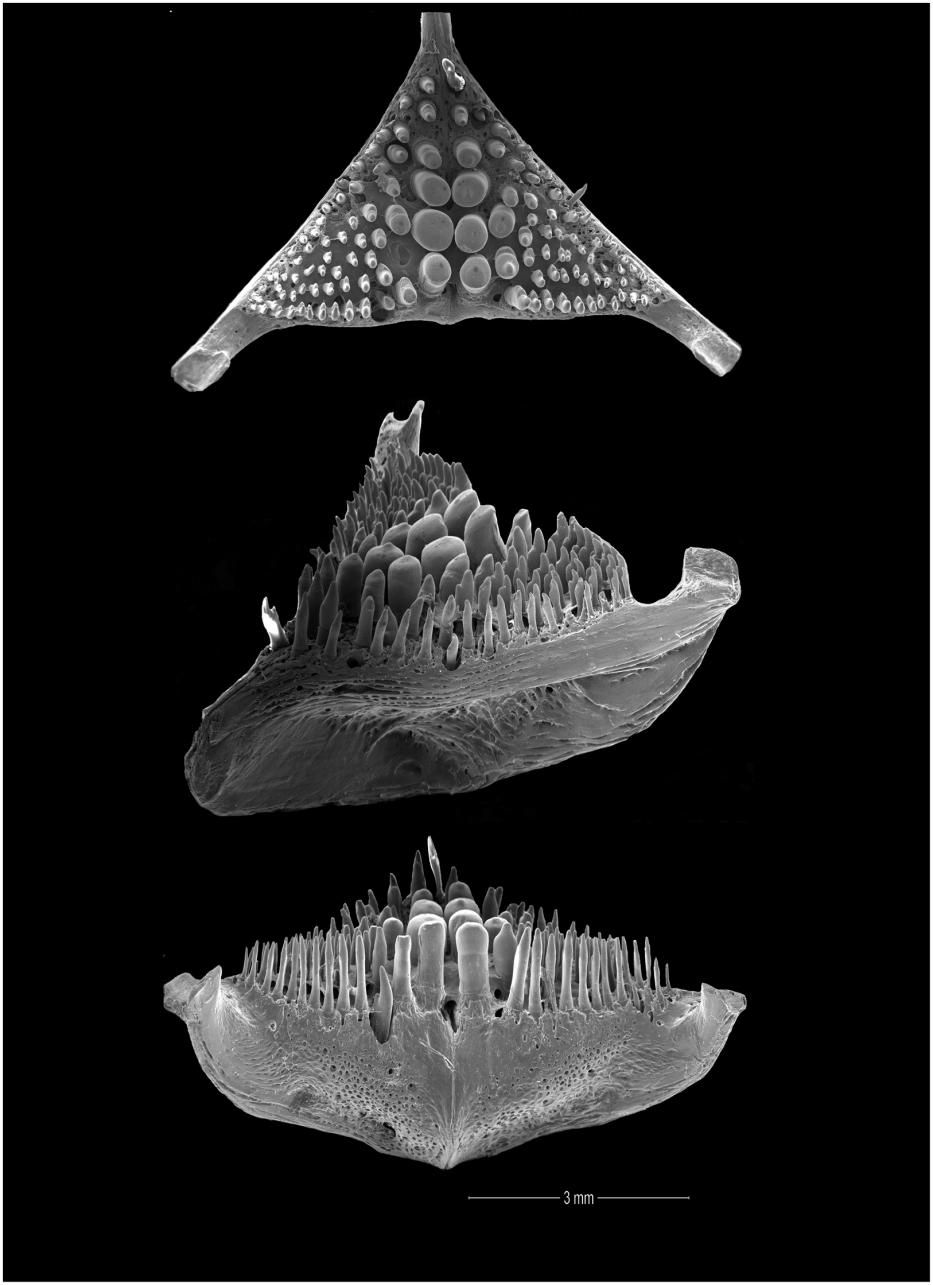
*Australoheros mboapari*; lower pharyngeal tooth plate in occlusal (top), anterolateral (middle) and caudal (bottom) aspect; MCP 230444, 68.4 mm SL.

Lower pharyngeal tooth plate (Fig 27) moderately stout, wider than long (length 73% of width); dentigerous area triangular (length 60% of width); In admedian row of 6 teeth and flanking teeth on right side, anteriormost teeth slender, erect, posterior teeth in sequence gradually stouter, with apex flat or with minor median cusp; remaining teeth slender, erect, with minor anterior subapical widening and antrorse posterior cusp, gradually smaller toward bone margins.

Scales on body finely ctenoid. Circumpeduncular scale rows 16 (28). Predorsal midline scales about 13–15, about half size of flank scales, covered by skin, only part with exposed margin, irregularly arranged, weakly ctenoid. Cheek scales absent or in 2–3 series on upper half of cheek, cycloid, covered by skin. Opercular scales mixed cycloid and ctenoid, covered by skin or margin partly exposed. Accessory lateral line scales absent from caudal fin. Between first upper lateral line scale and dorsal-fin origin four large scales. Between last upper lateral line scale and dorsal fin one large and two smaller scales, or one large and one small scale, exceptionally two large scales. Prepelvic scales about half size of flank scales, embedded in skin, without free margin. Lateral chest scales ctenoid, with exposed margin, about same size as flank scales. Fin scales ctenoid. Row of minute scales along dorsal-fin base from anterior spines or more posterior, basal squamation gradually expanded, interradial scales from between 9th to 14^th^ spine caudad, on soft fin in one or two rows with up to six scales between two soft rays, scales present also on last two interradial membranes. Anal fin with narrow basal scale layer, on soft-rayed portion up to six scales in interradial row.

Dorsal-fin spines increasing in length to 6th from which subequal, last spine longest, soft dorsal-fin rounded or subacuminate in young and some females, otherwise with broad pointed tip, fifth fin-ray longest, reaching to about 1/3 or middle of caudal fin. Soft anal fin rounded or subacuminate in young and some females, otherwise with a short point formed by fourth soft ray, reaching to about 1/3 or middle of caudal fin. Pectoral fin rounded, fifth ray longest, not reaching to vertical from genital papilla. Pelvic fin pointed, in males reaching to genital papilla or anterior 1–2 spines of anal fin, in females to genital papilla; first and second rays of nearly same length, outer or inner branch of first branched ray, or outer branch of second branched ray longest. Caudal fin subtruncate.

Vertebrae 13+14 = 27 (MCP 23044).

*Colouration in preservative*. Ground colour pale beige-yellow. Markings on head indistinct, including two grey stripes between orbits. A black spot on upper margin of pectoral-fin base. Occiput dark grey. Snout and gill cover light grey. Cheek pale greyish brown. Chest pale yellow or greyish yellow. In most specimens, caudal spot absent and caudal-fin base uniformly pale grey; in some specimens caudal spot present as dark grey bar across middle of caudal-fin base or as ill-defined greyish spot close above middle of caudal-fin base. Vertical bars indistinct; in large specimens (>70 mm) sides nearly uniform except for black midlateral blotch and variably expressed lateral band. Three dark stripes across scaled predorsal region (bars 7, 8, 9) indistinct. Bar 1 and Interbar 1 commonly absent, as caudal peduncle of same colour as caudal-fin base. Bars and Interbars straight vertical. Lateral band indistinct, expressed as contiguous black spots on anterior scales in E1 row and variably on anterior scales in row 0; continuous to Bar 5, variably expressed, but not distinct posteriorly on side.

When at all distinguishable, Interbar 2 indistinct, between dorsal and ventral margin of caudal peduncle; Bar 2 about three scales wide, from end of dorsal-fin base, not reaching anal-fin base; Interbar 3 about two scales wide, between down from middle of soft dorsal fin base but not quite reaching anal-fin base; Bar 3 about two or three scales wide, down from posterior two or three spines in dorsal fin but not reaching down to anal-fin base; Interbar 4 one scale wide, between about 3rd–4th from last spines in dorsal and anal fins. Bar 4 three scales wide, straight vertical from slightly above anal-fin origin to dorsal-fin base. Interbar 4 indistinct, about one scale wide, from above genital papilla dorsad to dorsal fin base. Bar 5 indistinct, about three scales wide, reaching dorsally to dorsal-fin base. Bar 6 absent below lateral band from about 30 mm SL, but present indistinct in some specimens up to 60 mm SL. Bar 7 expressed as a dark spot at anterior end of lateral band. Y-shaped markings absent from dorsal side.

Spinous dorsal and anal fins, and pelvic fin dark grey. Soft dorsal and anal fins, and caudal fin with light grey fin rays and contrasting dark brown (anal fin) or black (dorsal fin) interradial membranes. In males, black interradial pigment in dorsal fin concentrated to margin of next fin-ray; in females all of dorsal fin interradial membrane black, giving the appearance of a large black blotch. Dark blotches along base of spinous dorsal fin absent.

Juveniles 17.2–38.8 mm SL (MCP 23044) pale yellowish brown, fins hyaline. Caudal spot expressed either as black bar across middle of caudal-fin base or minute spot on dorsal lobe immediately above middle of base. Lateral band absent or faintly indicated in scale rows 0 and E1. Lateral bars 1–6 present, usually also bar 7, pale brownish, straight vertical, extending between dorsum and venter (total three abdominal bars). Bars absent or faint from about 40 mm SL. Large black midlateral spot, round or slightly deeper than long. Small spots or short dark dashes on interradial membranes of soft dorsal and caudal fins.

*Explanation of specific name*. The specific name is a noun in apposition, referring to the geographical distribution of the species, the Rio das Antas, which was previously known as Mboapari [114].

*Geographical distribution (*Fig 1*)*. Rio das Antas and tributaries from about Rio São Marcos to and in the Rio Carreiro.

*Comments*. *Australoheros mboapari* is parapatric with *Australoheros acaroides* in the region of Nova Roma do Sul. The two species can be separated by colour pattern, meristic data and proportional measurements. In *A*. *acaroides* the preorbital depth is distinctly larger and both the upper and lower jaw are slightly longer than in *A*. *mboapari*. The cheek scales are clearly evident in *A*. *acaroides*, arranged in 3–4 rows and covering almost all of the cheek unlike in *A*. *mboapari* in which the scales are few, deeply embedded, or absent.

*Australoheros mboapari* shares general appearance with *A*. *forquilha*, *A*. *ykeregua*, and *A*. *tembe* being relatively elongate, with deep lachrymal bone, low mouth, slightly elevated occiput at least in large specimens, embedded scales on head, cheek, and chest, indistinct or absent caudal spot, distinct midlateral spot, subdued vertical bars, relatively long caudal peduncle, and subtruncate caudal fin. It differs from those species by thinner lips, absence of small dark spots on dorsal and caudal fins, and slightly shorter basal scale row on dorsal fin. We suggest that the similarities with *A*. *forquilha*, *A*. *ykeregua*, and *A*. *tembe* reflect rheophily rather than phylogenetic relationship. Adults of all these species usually have strongly truncate anterior teeth usually with flat tips, whereas the coastal *A*. *acaroides* only has sharp caniniform teeth. We interpret the flat-tipped teeth as abraded, and resulting from sand taken in with food extracted from the bottom and/or hard shelled prey like snails. This interpretation follows also from the stout pharyngeal bone with enlarged molariform teeth typical of cichlids eating hard prey.

In the Rio Jacuí drainage, *A*. *mboapari* was most similar to *A*. *ricani*, e.g. in the long row of minute scales along the dorsal-fin base, and absent or indistinct caudal spot. The rare number of five anal-fin spines were recorded from both species. Non-parametric comparison of counts showed that they were distinct (Mann-Whitney U test, p = 0.000–0.001) in overlapping number of anal-fin spines (modally 5 in *A*. *ricani*, 6 in *A*. *mboapari*), dorsal-fin spines (modally 15 in *A*. *ricani*, 16 in *A*. *mboapari*), E1 scales, upper and lower lateral line scales, but not in number of soft dorsal- and anal-fin rays, or pectoral-fin rays. *Australoheros mboapari* had the highest number of E1 scales recorded in the genus, modally 26, occasionally 27. Twenty-six E1 scales were frequent also in *A*. *forquilha* and *A*. *ricani*.

*Australoheros mbapoari* differed from the similar and geographically adjacent *A*. *ricani* in slopes on SL of preorbital depth, lower jaw length, head width, pectoral-fin length, and length of last dorsal-fin spine (ANOVA p <0.05), and on intercept on head length, snout length, orbital diameter, interorbital width, and upper jaw length (ANCOVA, p <0.05). The slight difference in jaw lengths showed in the proportional measurements, Figs 13–16, where the size ranges are reasonably comparable), *A*. *mboapa*ri has slightly shorter upper jaw (8.8–10.9%SL (vs 10.2–11.5 in *A*. *ricani*), and lower jaw (11.7–14.2%SL vs 13.1–15.7% in *A*. *ricani*). *Australoheros mboapari* had deeper preorbital than the other coastal *Australoheros* excepting *A*. *acaroides* (Tables 13–16). This character, however, was positively allometric, and only extremely large specimens of *A*. *acaroides* had developed a deep lachrymal bone.

MCP 31176, from the Rio Guaporé, a tributary of the Rio Taquarí with mouth downstream from the mouth of the Rio Carreiro may represent *Australoheros ricani*, but grouped with *A*. *mboapari* in the PCA, and was left unidentified. It was included in the PCA, but not in the proportional measurements in Tables 13–16. The only adult included in the PCA deviated from *A*. *mboapari* in body proportions and relatively exposed scales. The upper Rio Guaporé is very close to headwaters of the Rio Jacuí.

*Australoheros taura* was based on specimens from the Antas and Tainhas rivers in the upper Rio Taquarí drainage [7]. Specimens and DNA sequences of *Australoheros* from the upper Rio das Antas and Rio Tainhas were identified as *A*. *acaroides*. The original description highlighted similarities with *A*. *tembe* in long snout and deep head, and indirectly suggested a morphology similar to *A*. *mboapari*. Known localities of *A*. *mboapari* are more downstream in the Rio das Antas than the recorded localities for *A*. *taura*. The original description of *A*. *taura* and examination of topotypes and paratypes agree with *A*. *acaroides* in rounded rather than subtruncate caudal fin, up to 25 vs 26–27 E1 scales, shorter row of minute scales along the dorsal-fin base, and fully scaled cheek.

*Additional specimens examined*. Not type:. MCP 31176, 13, 30.3–94.3 mm SL. Brazil: Rio Grande do Sul: Rio Taquarí drainage: Guaporé: stream tributary of the Rio Guaporé at PCH Guaporé. A. R. Cardoso and V. A. Bertaco, 2 Dec 2002. 28°54’S, 51°56’W.

#### *Australoheros ricani*, new species

*Definition*. Based on the position in the phylogenetic trees based on *mt-cyb* and *mt-coI*, and more than 3% divergence in minimum uncorrected *p*-distance from other species of *Australoheros*, *A*. *ricani* is a distinct evolutionary lineage. No morphological autapomorphy was registered. Specimens of *A*. *ricani* share with *A*. *mboapari*, *A*. *forquilha*, and *A*. *ykeregua* a row of minute scales along the dorsal-fin base extending cephalad to close to the anterior insertion of the dorsal fin (4th dorsal-fin spine), and with *A*. *mboapari* a black soft dorsal fin in females (vs dorsal fin black throughout, or with black blotches at intervals). It is distinguished from *A*. *mboapari* by 3–5 rows of scales on cheek, exposed or discernible under thin skin cover.

**Fig 28:**
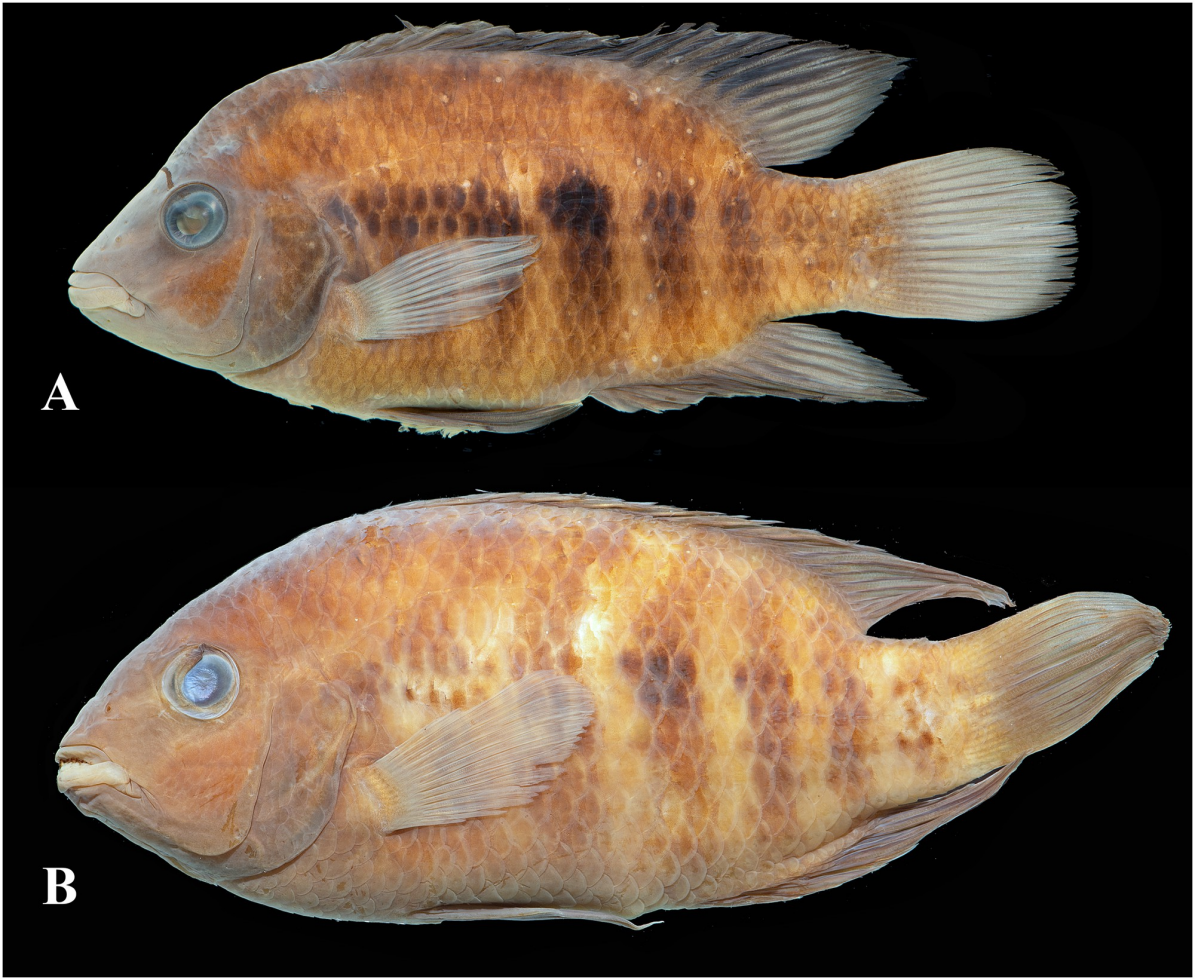
**A.** *Australoheros ricani*, holotype adult female, 75.5 mm SL; UFRGS 28500; Brazil: Rio Grande do Sul, Rio Jacuí drainage: Espumoso: Rio Morcego, tributary of the Rio Jaquí-mirim. **B.** *Australoheros* cf. *ricani*, “Jacuí”, adult female, 105.2 mm SL; MZUSP 30411; Brazil:_Rio Grande do Sul: Marau: Rio Jacuí drainage: Arroio Três Passos, tributary of the Rio Jacuí-mirim.

*Holotype*. UFRGS 28500, adult female, 75.5 mm SL (Fig 28A); Espumoso: Rio Morcego, 28°53’55’’S 52°49’0.5’’W; K. Bonato and R. Dala-Corte, 18 Dec 2012.

*Paratypes*. All from Brazil: Rio Grande do Sul, upper Rio Jacuí drainage. Rio Jacuí-mirim: MCP.

Saldanha, 28°26’39’’S 53°12’37’’W; R.E. Reis, E.H.L. Pereira and V.A. Bertaco, 2 Apr 1999.—Mormaço: Rio Quati, 28°38’52’’S 52°37’11’’W: UFRGS 22015, 1 juv.; K.O. Bonato, P.C. Silva, C. Hartmann, and A. Langoni, 26 Aug 2012—UFRGS 22016, 4 juv.; K.O. Bonato, J. Wingert, L.G. Artioli, 27 Aug 2012—UFRGS 22020, 3, 20.9–56.9 mm SL; K.O. Bonato, A. Hirschmann, A. Hartmann and A. Langoni, 20 Apr 2013—UFRGS 22026, 4, 27.4–90.7 mm SL; UFRGS 22028, 4, 39.8–77.3 mm SL; K. Bonato, N. Bertier, A. Hirschmann, 21 Jun 2012—UFRGS 22036, 4, 22.6–92.0 mm SL; K. Bonato, N. Bertier, A. Hirschmann, 19 Oct 2012.—UFRGS 22037, 4: 41.1–76.6 mm SL; K. Bonato, N. Bertier, A. Hirschmann, 19 Dec 2012.—Espumoso: Rio Morcego, 28°53’55’’S 52°49’0.5’’W: UFRGS 19968, 11, 4, 22.4–84.9 mm SL; K.O. Bonato, J. Ferrer, C. Voguel and L. Cavalheiro, 19 Jun 2012.—UFRGS 22027, 8, 14.0–81.6 mm SL, K. Bonato, A. Hirschmann, C. Hartmann and S. Langoni, 20 Apr 2013.—UFRGS 22029, 9, 19.5–100.9 mm SL; K.O. Bonato, P.C. Silva, C. Hartmann and A. Langoni, 27 Jun 2013—UFRGS 22033, 4, 24.1–29.8 mm SL; K.O. Bonato, J. Wingert, and L.G. Artioli, 24 Aug 2012—UFRGS 22038, 3, 29.2–72.5 mm SL; K.O Bonato, N. Bertier and A. Hirschmann, 18 Oct 2012—UFRGS 22039, 4, 37.3–73.2 mm SL; K. Bonato, and R. Dala-Corte, 18 Dec 2012—UFRGS 22040, 15, 13.7–64.9 mm SL; K.O. Bonato and J. Ferrer, 22 Feb 2013.—Espumoso, Rio Turvo, 28°43’47’’S 52°47’40.4’’W: UFRGS 22017, 54.7–63.7 mm SL; K.O. Bonato, and R. Dala-Corte, 17 Dec 2012—UFRGS 22019, 1, 51.0 mm SL; K.O. Bonato, A. Hirschmann, A. Hartmann and A. Langoni, 19 Apr 2013.—UFRG 22025, 3, 57.8–68.3 mm SL; K.O. Bonato, J. Ferrer, N. Bertier and A. Hirschmann, 29 Oct 2012.—UFRGS 22032, 1 juv.; K.O. Bonato, J. Wingert and L.G. Artioli, 26 Aug 2012.—UFRGS 22034, 2 juvs; K.O. Bonato, N. Bertier, and A. Hirschmann, 29 Oct 2012.—Espumoso, Rio Turvo, 28°43’S 52°47’W’’: UFRGS 22021, 2 juvs; K.O. Bonato, J. Ferrer and C. Voguel, 29 Jun 2012.

*Description of type series*. Meristic data are given in Tables 3–9, proportional measurements in Table 18: Fig 28A shows the general aspect.

Sexes isomorphic except genital papilla in males slender, conical, in females wider, blunt. Both sexes with minor nuchal elevation present or absent. Moderately elongate, laterally compressed. Predorsal contour straight ascending to slightly posterior to orbit where curved, almost horizontal, slightly anterior to dorsal fin origin; a minor indentation in frontal contour anterior to orbit in most large specimens; occiput posteriorly markedly compressed and with slight compressed nuchal elevation in large specimens. Dorsal contour horizontal along about half of spinous dorsal-fin base, posteriorly descending, straight, or slightly curved. Caudal-peduncle contours slightly sloping or straight. Prepelvic contour slightly curved or straight. Abdominal contour straight horizontal. Anal-fin base slightly convex, ascending. Head short, laterally compressed. Snout short, blunt, subtriangular in lateral aspect, narrowly rounded in dorsal aspect. Mouth terminal, forwards directed, distinctly removed from lower margin of orbit. Lips moderately thick, lip folds interrupted anteriorly. Jaws equal in anterior extension; maxilla and premaxilla not reaching to vertical from anterior margin of orbit. Orbit removed from frontal contour, but close in specimens with frontal indentation; in middle of head length, major part in upper half of head. Teeth in both jaws subcaniniform, erect, in outer row slender, increasing in size toward symphysis; anterior teeth occasionally worn apically. No bicuspid teeth observed. Teeth in outer hemiseries in upper/lower jaw 7–15, 11–19; inner teeth in 2 rows in both jaws, much smaller than outer teeth. Gill rakers externally on first gill-arch 1–2 (usually) epibranchial, one in angle and 7 (11) ceratobranchial. Microbranchiospines present externally on 2nd to 4th gill-arch.

Scales on body finely ctenoid. Predorsal midline scales about 10–13, about 2/3 size of flank scales, covered by skin, only part with exposed margin, irregularly arranged, weakly ctenoid or cycloid. Cheek scales in 3–5 rows, covering all of cheek except narrow area anteroventrally; scales embedded in skin, but clearly visible, cycloid. Opercular scales mixed cycloid and ctenoid, covered by skin or margin partly exposed. Between upper lateral line and anterior insertion of dorsal fin, dorsal-fin origin four large scales One large and one small scale, or one large and two smaller scales separating last scale of upper lateral line from dorsal-fin base. Accessory lateral line scales absent from caudal fin. Prepelvic scales cycloid, about half size of flank scales, embedded in skin, with free margin posteriorly. Lateral chest scales finely ctenoid, with exposed margin, about same size as flank scales. Fin scales ctenoid. Row of minute scales along dorsal-fin base from between about 4th spine caudad, basal squamation gradually expanded, interradial scales from between 10th and 13th spine caudad, on soft fin in one or two rows with up to 8 scales between two soft rays, scales present also on last two interradial membranes. Anal fin with narrow basal scale layer, interradial scales from 6th spine, on soft-rayed portion up to 4 scales in interradial row. Caudal fin scales on proximal 1/3, in large specimens up to middle of fin.

Dorsal-fin spines increasing in length to 6th or 7th from which subequal, last spine longest, soft dorsal-fin subacuminate or with broad pointed tip, 5th fin-ray longest, reaching at most to about middle of caudal fin. Soft anal fin subacuminate or with a short point formed by fifth soft ray, reaching to about 1/3 of caudal fin, or slightly longer. Tip of pectoral fin rounded, 4th or 5th ray longest, not reaching to vertical from genital papilla. Pelvic fin pointed, reaching to or slightly beyond base of anterior insertion of anal fin; outer branch of first fin-ray longest, or both branches equal. Caudal fin rounded.

*Colouration in preservative*. Ground colour fawn or pale beige-yellow, each scale with distinct brown spot at distal margin; pale greyish along ventral surfaces. A black spot on upper margin of pectoral-fin base. Occiput brown. Snout, preopercle, gill cover, lower jaw light grey. Lips pale grey. Cheek light brown. Two faint brown stripes between orbits, crossing front. Caudal spot often obsolete, otherwise small, light brown, midbasal or located slightly dorsal to middle of base of fin. Vertical bars 1–5 distinct, brown. Bar 4 present above anal-fin origin. Bar 5 present anterior to vent and extending dorsad to lateral band. Interbar 4 between bars 4 and 5 present. Bar 6 and Interbar 5 present slightly posterior to pectoral fin-base. Bar 6 indistinct, not reaching below level of pectoral-fin base, but variably extended above lateral band. Bar 5 not divided vertically. In adult females Bars 1–4 or 1–5 may be dark brown or blackish brown except for dorsal and ventral terminal portions. Interspace between dark caudal-fin base and Bar 1 narrow or absent. Bar 1 not reaching lower caudal-peduncle margin; darker above lateral line where commonly also including black spot. Bar 2 between posteriormost soft rays of dorsal fin and anal fin, but usually not reaching anal-fin base. Bar 3 between about last 1–3 dorsal- and anal-fin spines, reaching to or not to anal-fin base. Short stripes crossing top of head just above eye (corresponding to Bar 8), across occiput (and through anterior dorsal-fin base (Bar 7).

Lateral band indistinct, from head to, but not crossing Interbar 4, formed by black or dark contiguous spots on scales in E1scale row and upper halves of scales in row 0; posterior vertical bars with intensified black or brown scales in same scale rows forming indistinct or deep black blotches in bars 4 and 3. Bars 2–4 distinct, bar 1 variably expressed; bars with variably darker or lighter scales, forming irregular and indistinct lines along posterior side. Y-mark formed by upper portions of Bars 5 and 6 present or absent.

Spinous dorsal- and anal-fins brown; in adult females, spinous dorsal fin and anterior soft dorsal-fin interradial membranes black. Pectoral fin pale grey or diaphanous. Pelvic fin brown or dark grey anteriorly, paler, to hyaline on inner rays; black in some adult females. Caudal fin with grey interradial spaces and lighter fin rays, slightly darker along posterior margin. Juveniles similar to adults in colouration, but vertical bars indistinct; distinct rows of small dark spots along flank; blotch in Bar 4 distinct; caudal spot absent.

*Explanation of specific name*. Oldřich Říčan, University of South Bohemia, was the first to recognise *Australoheros* species richness,, and made the pioneering analyses, discovering and highlighting the phylogeny and species richness of inland species of *Australoheros* [3, 4]. The specific name is a noun in the genitive case.

*Geographical distribution (*Fig 1*)*. Tributaries of the Rio Jacuí-Mirim, in the Rio Jacuí drainage, State of Rio Grande do Sul.

*Comments*. The type series of *Australoheros ricani* was most similar to that of *Australoheros mboapari*, and comments on measurements and meristic data are given under that species.

Most of the specimens were in bad condition, juveniles, and/or eviscerated. Two tissue samples from the Rio Morcego, tributary of the Rio Jacuí-mirim supported recognition of samples from the upper Rio Jacuí as representing a distinct species of *Australoheros* in the upper Rio Jacuí basin. DNA samples of *Australoheros* from downstream the Saltos do Jacuí, at Ibarama, Arroio Corupá, and further in the Rio Soturno basin, clustered with samples of *A*. *acaroides*. Samples from Rio dos Caixões and Rio Jacuizinho, with the mouth far below the Saltos do Jacuí, were assigned to *A*. *acaroides* based on morphology. This distribution pattern suggests that the Saltos do Jacuí formed a dispersal barrier, separating *A*. *ricani* from the more widespread *A*. *acaroides*. Two specimens from a tributary of the Rio Ingaí, originally draining to the Rio Jacuí-Mirim, were assigned to *A*. *acaroides*, with hesitation. It is possible that *A*. *ricani* may have been restricted to just the original Rio Jacuí-Mirim and upper Rio Jacuí, most of which now submerged in the Passo Real dam, but the pre-dam collecting record is limited to what is reported here.The Passo Real dam, blocking the Rio Jacuí in the region of Salto Grande do Jacuí, was inaugurated in 1973 The sample of *A*. sp. Jacuí”, MZUSP 30411, seems to be the only record of *Australoheros* from the upper Rio Jacuí before 1973. Several pre-dam samples are available from the region of the more downstream Dona Francisca dam, inaugurated in 2001, and those samples were identified here as *A*. *acaroides*.

*Australoheros* sp. “Jacuí” was recognised as a distinct species by Říčan and Kullander [3, 4], who included it in a phylogenetic analysis and in descriptive comparisons with other species of *Australoheros*. They disposed of only one sample (MZUSP 30411), in suboptimal state of preservation, which they referred to as “sp. jacui” and “sp. Jacui”. The collecting site of MZUSP was recorded only as ‘Arroio Três Passos, afl. do Jacuí Mun. Marau RS’, which we believe was a stream in the town Três Passos, southwest of Marau, and consequently in the Rio Jacuí drainage at approximately 28°28’00.0’ ’S 52°21’60.0’ ’W. The Três Passos stream was most likely a tributary of the Rio Jacuí-mirim. Records of other fishes collected by the same collector refer to ‘Rio Três Passos’.

The morphology and colour pattern of the sample of *A*. sp. “Jacuí” (Fig 28B) described by Říčan and Kullander ([4] and confirmed by us, agreed with the morphology and colour pattern of the the type series of *A*. *ricani* and consequently are inclined to refer *A*. sp. “Jacui” to *A*. *ricani*, with reservation for the differences in meristic data. Říčan and Kullander grouped *A*. sp.”jacuí*”* with *A*. *forquilha* and *A*. *tembe*, based on a morphological parsimony cladogram, specifically characterized by long cover of minute scales along the dorsal-fin base, with two scales instead of one, long caudal peduncle with more than two vertebral centra, and highest number (4) of scale rows between the anterior insertion of the dorsal fin and the upper lateral line. *Australoheros* sp. *“*jacuí” and *A*. *forquilha* were observed as unique in sharing a characteristic shape of the head and mouth, and possessing two instead of one scale in the anterior portion of the scale cover along the dorsal-fin base. The lower axial meristic data agreed better with *A*. *acaroides* than with *A*. *forquilha* and *A*. *tembe*. The phylogenetic analysis here (Figs 11–13), recovered *A*. *ricani* as closest to *A*. *acaroides* and similar species with horizontal stripes.

Meristic data and proportional measurement are given for *Australoheros* “Jacuí” in Tables 3–9, and 19, respectively. Low metameric meristic data distinguished the Três Passos sample from the type series of *Australoheros ricani*. This was particularly obvious in the consistently few anal-fin spines (modally 5) distinguishing the Três Passos sample from other *A ricani* as well as all other species of *Australoheros* which typically had at least 6 anal-fin spines, but there was a slight overlap (Table 7). The number of anal-fin spines was significantly different from the type series of *A*. *ricani* and other southern species with 5 anal spines (*A*. *mboapari*, *A*. *facetus*, and *A*. *acaroides*) (Mann-Whitney U test, p = 0.000–0.001). Low numbers of dorsal-fin spines (94% with 15 spines, vs 53%) and E1 scales (50% with 24, vs none contrasted with the type series of *A*. *ricani*¸ but were not otherwise exceptional. Low meristic data was reason for hesitation whether the Três Passos sample was of the same species as the other specimens here referred to *A*. *ricani*. PCA with and without pelvic-fin length and caudal-peduncle depth showed *A*. *ricani* as relatively distinct both from *A*. *acaroides* and from the Três Passos sample, the latter mainly overlapping *A*. *acaroides*. X-radiographs were available for the Três Passos sample only, with 13+13 (16) or 13+12 (2) vertebrae, reflecting the common condition (13+13) in the genus. The Três Passos specimens, however, had 2 (3 in one) vertebrae contained within the caudal peduncle, an unusual condition in the genus remarked on by Říčan and Kullander [4] (2008), and which may agree with a relatively long caudal peduncle in the type series of *A*. *ricani*.

*Additional specimens examined*. Not types: *Australoheros* cf. *ricani*: MCP 31176, 13, 30.3–94.3 mm SL. Brazil: Rio Grande do Sul: Rio Taquarí drainage: Guaporé: stream tributary of the Rio Guaporé at PCH Guaporé. A.R. Cardoso and V. A. Bertaco, 2 Dec 2002. 28°54’S 51°56’W.—*Australoheros* Jacuí”: MZUSP 30411, 18, 45.0–115.2 mm SL; Marau: Arroio Três Passos, affluent of the Rio Jacuí, 28°28’00.0’’S 52°21’’60.0’’W; G.Q. Benvegnú, 28 Jul 1971.

## Discussion

The present attempt to characterise species of coastal *Australoheros* using traditional morphometrics, a coalescence-based species delimitation method, and phylogeny, was moderately successful. Traditional counts and distance measurements showed very little variation across the species eventually defined despite that all those past species descriptions were founded mainly on meristic data and proportional measurements. Likewise, melanophore pattern was found to be very similar across the coastal putative species, although colour pattern was frequently an important component of species descriptions. The bPTP analyses of mitochondrial genes provided conflicting patterns of species delimitation, possibly a consequence of strong similarities across the ingroup, but also reflecting shortcomings of the methodology. The phylogenetic analysis, however, provided reciprocal strong support for a branching pattern resolving some of the problematic MOTUs in the bPTP analyses, and not conflicting with morphological data.

The main result of the present analysis is a new view of species composition, relationships and geographical structure of coastal species of *Australoheros*. Among the 33 nominal species in the genus, we recognise 15 as valid, and add two new species. Thirteen species were described recently from the Sudeste region of Brazil, but our data support only three species from this region (*A*. *ribeirae*, *A*. *oblongus*, and *A*. *ipatinguensis*) We recovered *A*. *acaroides* with a huge distribution in the Laguna dos Patos basin and *A*. *facetus* from the Paraná and Uruguay basins in Argentina and Uruguay. We discovered two new species (*A*. *mboapari*, *A*. *ricani*), and confirmed the distinctness of *Australoheros “*Jacuí”. Those species have limited distribution and possess distinct morphological characters by which they can be identified. Unlike in the well-demarcated congenerics in the Rio Uruguay basin, morphological identification is otherwise difficult with Atlantic versant species of *Australoheros*. This may be a consequence of very recent separation, shared crypsis or insufficient data.

The conception of two well separated groups of *Australoheros* was not corroborated. Most species have a southern distribution, and within that group, there are several distinct lineages. A disrupted distribution along the coast cannot be demonstrated, as localities of *Australoheros ribeirae* and *A*. *sanguineus* are relatively close. Indeed, coastal species tend to be more similar to each other than to the inland species, so that the contrast is rather between the species from the upper Uruguay and Paraná drainages and the coastal species, than between southern and northern species.

Our general conclusion is similar to the earliest phylogenetic analysis of the group in 2006 [3]. It built on the approach used by Wiens and Penkrot [115], contrasting morphological and molecular analyses in species delimitation, an approach inspiring integrative taxonomy [116]. The philosophy of integrative taxonomy was then that shortcomings of one analytical method may be balanced by the strength of another method but it may also be applied in a battery of data sets as envisaged by Pante et al. [117]. Říčan and Kullander’s 2006 analysis [3] included both morphology- and DNA-based analyses, and contrasted character-based species delimitation using multivariate and discriminant analyses on morphometric characters against parsimony-based phylogenetic analyses of DNA and morphology. The morphological analysis of species-discriminant character states (species as terminal units), differed only slightly from one using sets of character states (populations as terminal units). There were marked differences between the morphology-based and the DNA based tree topologies, however. Part of the explanation for that may be that in 2006 DNA was available only for about half of the species, and some samples were misidentified, affecting correct identification of *A*. “jacutinga” (morphological sample from *A*. *angiru*, but DNA sample from *A*. *sanguineus*, as identified herein; DNA sample from A. “uruguai” from Uruguay identified as *A*. *acaroides* herein, and not likely to be from Uruguay). With the necessary adjustments of identifications, the results from Říčan and Kullander [3] are corroborated here. Říčan et al. [13] used the same methodology as Říčan and Kullander [3], but with improved molecular coverage. Those results are corroborated here as far as species overlap.

The most distinctive species in the genus reported previously were those in the Uruguay and Paraná basins that are associated with fast flowing water–*Australoheros forquilha*, *A*. *tembe* and *A*. *ykeregua–*whereas other species have a generalised body shape and colouration shared with most cichlasomatin and many heroin cichlid species. Also, the new species described here belong in this category, and were recognized for their morphology. Whereas species status was confirmed with DNA analysis for *A*. *ricani*, no fresh collections of *A*. *mboapari* are known, and it was recognised as distinct only by morphological characteristics.

### Crypsis in *Australoheros*

The introductory message of Ottoni et al. [47], as well as the conclusion, characterised portions of *Australoheros* as cryptic: species were misidentified as *A*. *facetus*; ‘species of the ‘autrani’ group are very similar and difficult to diagnose’; ‘*Australoheros* has a taxonomically confused and controversial history’.

We have found that it may be difficult to establish a formal ‘diagnosis’ for species of *Australoheros*, but even so, by reducing the Sudeste species to three, there remain no truly cryptic species, only nominal species that still have not been analysed morphologically to any depth. Species of *Australoheros* may be cryptic in one respect, however. Observations on live *A*. *minuano* and *A*. s*citulus* showed that they were capable of changing the melanophore and erythrophore pattern rapidly and frequently (SOK, pers. obs.). Older aquarium literature [60] also mentions this characteristic for *A*. *facetus*. Notwithstanding, and somewhat unexpected, there exist no controlled studies on the pigmentation or behaviour of species of *Australoheros*. The only dedicated ethological study of *Australoheros* [25] did not mention colour pattern or colours, but different expressions of the colour pattern were illustrated by Baduy et al. [21]. We nevertheless suggest that species of *Australoheros* maintain, by stabilising selection, a disruptive melanophore pattern that can be adjusted to habitat, probably for camouflage, i.e. their colour and behaviour is cryptic.

### Unresolved identifications and missing data

Our results point to several problem cases. One tissue sample from the Rio Arapey was identified as *Australoheros minuano*, but is highly divergent. It may represent yet another taxon or intraspecific variation. Only additional material and analysis can shed light on this case. Our material is deficient in representation of the type locality of *A*. *autrani*, and absence of material from the type locality of *A*. *saquarema*.

Absence of tissue samples representing *Australoheros minuano* from the type locality provides for some hesitation in describing new species from the Rio Uruguay basin, as the type series of *A*. *minuano* is very similar to *A*. *acaroides*, *A*. *minuano*, and *A*. *ricani*, and the original description combined data from *A*. *minuano*, juveniles from the Rio Quaraí, and large preserved adults from the type locality in the Rio Piratini drainage.

The distinct scale morphology and unique low meristic data of *Australoheros “*Jacuí” might justify species status of the single sample available to us. Nevertheless, we prefer the recognition without a formal scientific name pending the availability of fresh preserved specimens and DNA for a strong basis for a decision.

*Australoheros mboapari* emerged out of morphological comparisons with the parapatric *A*. *acaroides*. It remains known only from specimens fixed in formalin; no DNA is available.

Limited observations suggest that at least some species of *Australoheros* have a specific breeding colour pattern with black breast region and increased amount of yellow or red on the sides, and probably highly species specific. Again, there are no behaviour studies available on colour pattern in *Australoheros*. South American cichlid species, as diurnal and biparental with distinct sexual dimorphism, for the most part characterised by elements of the colour pattern, predomimantly the melanophore pattern in preserved specimens [118–121]. Other characters are usually close to uniform across a genus. Many of the South American cichlid species are or have been in the aquarium hobby and there are numerous observations and published photographs documenting species specific live colours and colour patterns that are informative for species taxonomy and make useful diagnostic characters [61, 26, 121]. Species of *Australoheros*, however, have not been important in the aquarium trade for decades, and there is very little information from hobbyists over the past 50 years. It seems highly likely that controlled behaviour studies including live colour variation will contribute to species taxonomy among species of *Australoheros*.

#### Species delimitation

Analyses of *mt-cyb and mt-coI* confirmed species status for previously described species from the Paraná and Uruguay basins, as well as southern species including *A*. *sanguineus* and justify recognition of *A*. *ricani*, and the sample here referred to *A*. *minuano*. In fishes, 2–3% uncorrected *p*-distance in *mt*-*coI* has commonly been considered as a species level cutoff [49, 122]. This may be sufficient for a uniform group of species, but may not reflect a genetically diverse group with several different evolutionary histories, or extend across different genes. Coalescence analyses seek to find multiple branching points (monophyly) along gene trees, and are implemented in several DNA-based species delimitation methods. We used here bPTP [73] which takes a fully resolved Bayesian tree as a start, and which gives a probability measure for each identified ‘species’. For the most part of the results, the bPTP terminals agree with character or DNA- based species determined by morphometry and phylogenetic analysis. Failure to find a credible gene tree, as in the *mt-cyb* analysis here, may reflect weaknesses in the data (largely from GenBank) or ongoing gene flow among the many nominal species from the Sudeste, which latter can impact the delimitation [123]. The bPTP analysis with *mt-coI* agrees with the BI trees of both *mt-coI* and *mt*-*cyb*, and the uncorrected *p*-distances, and we assume this as the best phylogenetic scenario hypothesis and species delimitation.

Our molecular analysis is methodologically similar to that of Ottoni et al. [47] in the application of Bayesian Inference and bPTP, and the data overlap; the major difference is our *mt-coI* dataset., whereas Ottoni et al. only used the *mt-cyb* gene. Where we do not see any species differences, and we can show that genetic distances are minor in the northern species, especially compared with the interspecies distances in the southern species, Ottoni et al. distinguish species. This is not due to difference in length of the *mt-cyb* fragment (Ottoni et al.’s sequences are slightly shorter) or to major differences in the data sets (the *mt-*cyb datasets are almost identical). However, the minor differences/short branches between the nominal taxa of the “Autrani” group in the BI tree were regarded as species and the bPTP analysis was made with a very much reduced specimen sampling compared to the BI tree, with only one or two specimens of each nominal species, and a reduced outgroup of non-Sudeste species. Support values were not reported. The bPTP analysis is otherwise not incompatible with our rejected *mt-cyb* analysis.

When we assigned samples from the Sudeste drainages to localities according to original descriptions, only two species were recognisable from *mt-coI* and *mt-cyb*, with practically no within-group variation. Consequently, *A*. *autrani*, *A*. *macaensis*, *A*. *muriae* and *A*. *capixaba* are considered to represent a single species with *A*. *ipatinguensis* as the oldest available name; and *A*. *macacuensis*, *A*. *robustus*, *A*. *montanus*, *A*. *paraibae*, *A mattosi*, and *A*. *barbosae* are considered to represent a single species, with *A*. *oblongus* as the oldest available name. DNA was not available from type localities of *A ipatinguensis* or *A*. *saquarema*, and consequently absent in the *mt-coI* dataset. *Australoheros perdi* is represented in the *mt-coI* dataset by samples from Pingo-d’Água, adjacent to the type locality. Our *mt-cyb* sequences combined with GenBank data confirm the *mt-co1* result, and extend synonymisation to *mt-cyb* MOTUs. *Australoheros oblongus* is restricted to the middle course of the Rio Paraíba do Sul in Minas Gerais, the Rio das Velhas drainage in the adjacent Rio São Francisco drainage, tributaries of the upper Rio Paraná (Rio Tietê, Rio Grande and the isolated Rio Macacu, which drains to the Baia de Guanabara, and a small costal river in Ubatuba, south of Rio de Janeiro. DNA was not available from the Rio Guandu population. *Australoheros ipatinguensis* has a wide distribution close to the Atlantic coast, extending from Rio Saquarema near Rio Janeiro to the Rio Buranhém in southern Bahia, but DNA samples were available only to the Rio Itaúnas drainage in northern Espírito Santo to the north, and to the Rio Macaé in Rio de Janeiro to the south.

Ottoni et al. [47] used two species *mt-cyb* delimitation analyses that we did not try to replicate. ‘WP’, based on Wiens & Penkrot’s [117] proposal of species delimitation based on phylogenies of haplotypes; and one they called “CBB’ and described as character-based DNA barcoding referring to DeSalle et al. [124]. DeSalle et al. and their references focused on barcode reader construction and expressed a critique of tree-based species delimitation, particularly the use of distances in species delimitation. Their counterproposal was mainly to use nucleotides as taxonomic characters. Ottoni et al. [47] implemented this in maps of nucleotide substitutions. Ottoni et al. also referred to BI values, but not for species delimitation.

The WP and CBB analyses were reported as both delimiting 11 species: *A*. *ipatinguensis*, *A*. *macaensis*, *A*. *macacuensis*, *A*. *muriae*, *A*. *perdi*, *A*. *sanguineus*, *A*. *ribeirae*, *A*.cf. *capixaba*, *A*. *autrani*, *A*. *barbosae*, and *A*. *robustus*, synonymising *A*. *mattosi A*. *paraibae*, *A*. *saquarema*, *A*. *tavaresi*, and *A*. *montanus*. Seven species (*A*. *autrani*, *A*. *barbosae*, *A*. *mattosi*, *A*. *paraibae*, *A*. *robustus*, *A*. *saquarema*, *tavaresi*, and *Australoheros* cf. *montanus*) ended up in three species: *A*. *autrani*, *A*. *barbosae*, and *A*. *robustus*).

For practical purposes, uncorrected *p*-distances in the BI tree, used by us, equates the WP method as a measure of gene flow.

The bPTP analysis was reported as recovering nine lineages within the “*A*. *autrani* group”, of which *A*. *macacuensis*, *A*. *macaensis*, *A*. *muriae*, *A*. *ribeirae* and *A*. *sanguineus* were stated to corroborate morphologically established species. Nine other nominal species (*A*. *autrani*, *A*. *barbosae*, *A*. *ipatinguensis*, *A*. *mattosi*, *A*. *paraibae*, *A*. *perdi*, *A*. *robustus A*. *saquarema*, *A*. *tavaresi*), and two tentatively identified species (*Australoheros* cf. *capixaba*, *Australoheros* cf. *montanus*) were reduced to four: *A*. *autrani*, *A*. *barbosae*, *A*. *ipatinguensis*, and *A*. *robustus*. *Australoheros paraibae*, and *A*. *tavaresi* were treated as synonyms of *A*. *barbosae*. This is not in conflict with our analysis. *Australoheros saquarema* was treated as a synonym of *A*. *autrani*. This is not in conflict with our analysis. *Australoheros mattosi* and *A*. cf. *montanus* were treated as synonyms of *A*. *robustus*. This is not in conflict with our analysis given that the identity of the *A*. cf. *montanus* can be confirmed. *Australoheros perdi* and *A*. cf. *capixaba* were treated as synonyms of *A*. *ipatinguensis*. This agrees with our analysis, given that the identity of the *A*. cf. c*apixaba* can be confirmed.

Weighting the bPTP against the WP and CBB methods, Ottoni et al. [47] concluded that there were nine or 11lineages of *Australoheros* in the Sudeste, i.e. *A*. *ribeirae*, *A*. *sanguineus*, *A*. *barbosae*, *A*. *macacuensis A*. *robustus*, *A*. *muriae*, *A*. *macaensis*, *A*. *ipatinguensis*, and *A*. *autrani* for nine bPTP); add *A*. *perdi* and *A*. cf. *capixaba* for the sum of 11 (WP and CBB).

In the BI tree [47], fig. 2, there is a very strong difference between northern and southern species in branch lengths, which includes even very similar OTUs in the south (e.g. *A*. *facetus*) and which rather suggests that the northern MOTUs are homogenous rather than distinctly branching, but this was not commented on.

#### Phylogeny and geographical distribution

The coastal species of *Australoheros* do form a clade distinct in morphology and DNA from the Uruguay and Paraná species studied by Říčan and Kullander [3, 4] and Říčan et al. [13]. The Uruguayan species as a rule exhibit distinct colour patterns and morphologies, and show more intra-group variation than the coastal species. Geographically, there are no sharp limits in distribution, however. *Australoheros scitulus* is the most common species of *Australoheros* in Uruguay, present from the coast upstream to the Brazilian part of the river; *A*. *acaroides* is widespread in the Jacuí drainage and adjacent streams in the Laguna dos Patos basin, but also present in the uppermost Rio Uruguay (Rio Pelotas).

Ottoni et al.’s [4, 6, 7] suggestion of a southern and a Sudeste group of species based on morphology, was based on the vertebral count, and due to different methods of counting only. Nonetheless, some species from the Upper Uruguay and Paraná drainages (*A*. *scitulus*, *A*. *tembe*, *A*, *forquilha*, and *A*. *ykeregua*), average more caudal vertebrae (24 or 25) than other *Australoheros*.

Whereas *Australoheros sanguineus* is by geographical distance closer to the southern species, the *mt-coI* tree (Fig 11) places *A*. *sanguineus+A*. *ribeirae* as sistergroup to the northern species, and the *mt-cyb* tree has *A*. *sanguineus*+*A*. *ribeirae* as part of a trichotomy with the northern and southern species (Fig 13).

The most surprising discovery concerning distribution was the presence in the upper Rio Iguaçu drainage of *A*. *sanguineus*, a species described from the coastal Rio Cubatão. There are no published records of *Australoheros* from the Rio Iguaçu drainage before 2006 (cf. Casciotta et al. [83]: *A*. *kaaygua*; Říčan and Kullander [3]: misidentified *A*. *sanguineus* as *A*. *kaaygua*). This could be due to insufficient collecting and but also slow expansion of unintentional introductions from fish farms. Another cichlid species found in several localities in the upper Rio Iguaçu is a member of the *‘Geophagus’ brasiliensis* species group, common in coastal rivers, was considered to be most likely introduced in the Rio Iguaçu drainage basin [1]. A sample reported here under *Australoheros* sp. cf. sanguineus collected in 1944 in the Rio Iguaçu basin near União da Vitoria opens for alternative scenarios. It consists of juveniles of a species of *Australoheros* and *‘Geophagus’ brasiliensis*. Unfortunately, the small size and condition of the specimens prevents identification at species level.

Samples from the Laguna dos Patos drainage and the coast of Santa Catarina south of Imbatuba, as well as samples from the upper Rio Pelotas in the Uruguay River basin and tributaries to Laguna Merín in Uruguay, belong to a widely distributed species, *A*. *acaroides*. *Australoheros facetus* is widely distributed in the Uruguayan and Argentinian portions of the lower Rio Uruguay. Distinct species are present in the upper Rio Jacuí and, with reservation, the Rio Yaguarón. A distinct species is present in the Rio das Antas, but no DNA was available. *mt-coI* sequences were not available for several species: *Australoheros mboapari*, *A*. *minuano*, *A*. *guarani*, and the nominal species *A*. *tavaresi* and *A*. *perdi*.

In the extreme south, *Australoheros acaroides* is a widespread species in the rivers draining to Laguna dos Patos and Lagoa Mirim, and tributaries of the Rio Pelotas (uppermost Rio Uruguay). *Australoheros facetus* is present in rivers along the Uruguayan coast and the adjacent lower Rio Paraná. In Uruguay, the Cuchilla Grande complex separates the Rio Negro drainage from rivers draining to the Lagoa Mirim, what apparently also keeps *A*. *facetus* confined to rivers west of the Cuchilla Grande divide. The only anomaly is *A*. *minuano*, which was collected both in the Yaguarón and Negro drainages and in the Rio Taquari, draining to the Laguna Merín.

The Cuchilla Grande Range (maximum elevation 513 mASL), and contiguous ranges to the northwest on the Brazilian-Uruguayan border, seems to form a barrier separating *A*. *facetus* and Uruguay basin species from the neighbouring *A*. *acaroides* (sister species of *A*. *facetus*) and remaining northern species. Outcrops of the Serra Geral north of Imaruí separate *A*. *acaroides* and *A*. *sanguineus*. The further northern distribution of the genus largely extends along the coast, with distinct populations in isolated rivers, but notably without records from many rivers and lagoons.

Thomaz et al. [125, 126] presented a Brazilian Pleistocene coastline model based on a -125 m glacial maximum sea level drop about 18kya BP, affecting the many isolated coastal drainages along the Brazilian coast. It supports palaeodrainages with extended courses among which the named Tramandaí, Laguna dos Patos, Itajaí Ribeira de Iguape, Paraíba do Sul, Doce, Ubatuba, Macacu, Saquarema, Itabapoana, São Francisco, Tietê, Macaé/São João, Mucuri, and Itabapoana are relevant for *Australoheros* as extensions of the present drainages. The example species studied by Thomaz et al. [125, 127, 128], *Hollandichthys multifasciatus* (Eigenmann and Norris, 1900) did show variation along the coast. Except for the description of a second species of the genus at the extreme south of the distribution of the genus, *Hollandichthys taramandahy* Bertaco & Malabarba, 2013 [129], no species level taxonomic analysis was made. The latter species, restricted to the Araranguá, Mampituba Maquiné, and Tramandaí and Três Forquilhas river drainages [129] overlaps with the northern distribution of *Australoheros acaroides*.

The South American coastline line was further affected by the Holocene transgression reaching about 4 metres 5 kya BP from the Rio de La Plata to northern Brazil [130–133] and flooding the coastal lagoons with salt water.

Because several coastal species occur in more than one river basin, e.g. *A*. *acaroides* in the Jacuí and Pelotas (Uruguay) basins, *A*. *oblongus* in the Paraná, Doce, Ubatuba, Macacu, and São Francisco basins; *A*. *ipatinguensis* in several small river basins, and the Rio Doce basin; *A*. *minuano* in the Laguna Merín and Rio Uruguay basins; and possibly *A*. *sanguineus* in the Cubatão and Iguaçu basins, events of stream capture may have been significant in dispersal and speciation in coastal *Australoheros*. Particularly southeastern and northeastern Brazil has a long history of tectonic activity and stream capture expressed in Recent fish distributions [134]. The extensive overlap of *A*.*ipatinguensis* and *A*. *oblongus* in the lower Rio Paraíba do Sul, as well as widely distributed in coastal rivers north of the Rio Paraíba do Sul, might fit in the palaeodrainage scenario, but it is uncertain what the effects could have been. Rather than a coastal biogeography, we propose that *Australoheros* is mainly associated with the Paraná and Uruguay River basins, and the coastal species are derived not from coastal river jumping, but rather stream capture events in the Rio Paraná drainage.

### Species as concepts/hypotheses vs diagnoses

Říčan et al. [13] showed that the morphological characters presented by Ottoni et al. [5–8] did not support the proposed species, and we have also demonstrated here that those species and others described from the Sudeste up till 2012 were not justified on the basis of the data provided in the original diagnoses.

*autochthon*. In lieu of other justification, each nominal species was given a complex diagnosis based on character states in combination, including both character states observed on the type series, and states cited from literature (mainly from Říčan and Kullander8 [4]). Reservations concerning the diagnoses are detailed in the species accounts above. Whereas all conclusions herein also are open to testing, and have known limitations, the wide discrepancy in species recognition may require a comment.

In systematic biology, a diagnosis is ‘a brief listing of the most important characters or character combinations that are peculiar to the given taxon and by which it can be differentiated from other similar or closely related ones’ [135], but it also has the function of complying in descriptions of new species with the requirement in the International Code of Zoological Nomenclature [43] of a statement of distinguishing characters (Article 13.1.1). Already Blackwelder [136]. noticed that diagnoses were often very sketchy. Diagnoses are typically given only in the original description of a species to comply with the Code, a ‘diagnosis’ does not need to be correct, and incorrect information later detected in the diagnosis have no consequences for the availability of the name. Providing a diagnosis, hence, does not equal a species delimitation or showing a justification for species status of a sample. Dubois [137], also recognising the duality of ‘diagnosis’, considered taxonomic diagnoses as intensional (character-based) definitions of taxa (concepts), and provided a thorough review of the concept of diagnosis itself. Obviously, a name and a ‘diagnosis’ for the fulfilment of requirements of the Code is not a justification that a certain sample described by character states in the definition justifies recognition of a new taxon. The best examples here come from composite morphology-based species, i.e. an invalid concept that at some time defined a species. Consequently, the justification for a species based on a specific sample of individuals and/or specimens, becomes the crucial part of the taxonomic analysis. Almost all named species of South American fishes were based on ad hoc definitions describing a sample–character-based delimitation as used in Říčan and Kullander 2006 [3]–rather than conceptualisation underpinned by autapomorphy or phylogenetic position. As demonstrated by Říčan et al. [13]) and above, diagnoses of multiple Sudeste species of *Australoheros* fail the discrimination criterion but fulfil the nomenclatural requirement.

Dichotomous identification keys in biology are related to ‘diagnoses’, enabling less experienced users to make identifications using simple markers [135, 136]. This requires that the species in question is valid, that it has a simple marker, preferably unique in the genus or family, and that the user enters the key at a valid dichotomy (same family or genus as the target). In poorly known groups, or groups with less conspicuous markers, keys may be inefficient or insufficient for informed identifications.

Previous studies of *Australoheros* were mainly descriptive or analytical, and did not attempt to popularise the findings. That is reasonable given the absence of documented characters for identifying nominal species in the genus as a whole. Out of 18 taxonomic or phylogenetic papers on *Australoheros* published from 1995 until 2017, two contain identification keys [4, 84]. The genetic data presented herein are public and can be used for species determination of unidentified samples. Species distributions are relatively well demarcated and can be used to reduce options for species identification, particularly in combination with available meristic data. Practical tools such as determination keys are not available for non-specialists to confidently identify specimens of *Australoheros* in the field or under lab conditions, or living individuals in the ornamental fish trade. Such tools should ideally be worked out in future research and by practitioners in a relevant geographical context, observing autapomorphies and ontogenetic stages as well as sexual dimorphism and reproductive status. Because determination keys are intended for users with little experience in identifying species of the group concerned and require at least one autapomorphy or unique marker for each species, our presentation of discriminating characters must mark the limit of confident morphological species markers in *Australoheros*.

Alpha taxonomy is a science that traditionally has been based on positive evidence only, as exemplified by diagnosis-/character-based species delimitation and characters in combination. Validity is not based on tests or falsifiability, but on opportune continuous corroboration over time, except that often not even corroboration is achieved over time. ‘*Cichlasoma facetum*’ is a good example of a species that has been recognised and referred to through generations of ichthyologists, despite that it has not been more than a name. ‘Characters in combination’ is a similar phenomenon but somewhat in reverse when based on sample data. The more character states put into the combination, the more likely it is that one of the character states is not found in a specimen and then the species is invalidated in toto, whereas additions do not affect validity. Species by species comparisons is also a method that can provide false corroboration. Most likely any two samples will be different in some way, it is just a matter of digging a bit or mixing own data with data published by others. The method is also sensitive to revised knowledge of the comparison species. The best known weakness in species delimitation is number of specimens; a single specimen may just be an aberrant individual and small geographically separated samples may reflect local conditions only. Very large samples, on the other hand, may obfuscate existence of rare species markers. Those practices suggest that, implicitly, the null hypothesis is that samples are different, rather than same. On top of that, the human factor is a taxonomist, and the main occupation of a taxonomist is to describe species or fail professionally.

We also, have not been successful in providing morphological discriminating identification tools for species proposed, i.e. defining species. Aside from practical shortcomings, particularly access to well-preserved specimens and tissue for DNA analysis, limit the option for corroborated species hypotheses.

Naming or just proposing a species based on properties of a sample is an act of classification that can be justified as a hypothesis essential for testing [138] but this also means that species taxonomy must challenge the doctrine of stability of names because every named unit must primarily be a considered a working hypothesis and not an established fact. Absence of tests of the particular hypothesis indicates a low level of corroboration of a species taxon as a valid evolutionary unit or lineage. Relieving taxonomy of the naming procedure and accepting that existing names are temporary designations is probably to ask too much of the system, but emphasising that particular species are concepts subject to indefinite testing, allowing competing results at every moment, may contribute to a more stable taxonomy.

The unified species concept [50] provides a framework for species recognition as it focuses on the evidence of lineage separation and a view of species as concepts defined by specific properties, in contrast to defining species by ad hoc sample properties. Conversion to a hypothesis-based South American fish taxonomy will be a major challenge, but also open new perspectives to fish evolution.

## Supporting information

S1 File GenBank and BOLD sequenes used. (PDF)

S2 File Extralimital species of *Australoheros*. (PDF)

S3 File Box plots of proportional measurements. (PDF)

S4 File Principal component analyses: Plots and tables. (DOCX)

S5 File Bayesian phylogenetic tree of concatenated *mt-coI* and *mt-cyb* sequences. (PDF)

S1 Table Uncorrected p-distances. (XLSX)
