# Correction: Conjectures and refutations: Species diversity and phylogeny of *Australoheros* from coastal rivers of southern South America (Teleostei: Cichlidae)

**DOI:** 10.1371/journal.pone.0323504

**Published:** 2025-04-30

**Authors:** Carlos A. Santos de Lucena, Sven Kullander, Michael Norén, Bárbara Calegari

The following Zoobank ID is missing from the first page of the article: Zoobank LSID urn:lsid:zoobank.org:pub:4467D5D3-0198-4E7A-9909-7CA40B7EECCD.
