# Supplementary material for: Conjectures and refutations: Species diversity and phylogeny of Australoheros from coastal rivers of southern South America (Teleostei: Cichlidae)
Source: PLoS One. 2022 Dec 9;17(12):e0261027. doi: 10.1371/journal.pone.0261027 (PMC9733902; doi:10.1371/journal.pone.0261027)
Supplement: S1 File — (PDF) [file pone.0261027.s001.pdf]

S1 Table **Supporting information : GenBank and Bold sequencesf Australoheros used**

Ref.	*mt-CoI*	*mt-cyb*	Collcode	Cat no	Tissue	Species	Source ID	Country	State
NCBI	JX111689		UNMDP-T	359		*A. facetus*	*A. facetus*	Argentina	Buenos Aires
NCBI	JX111687		UNMDP-T	314		*A. facetus*	*A. facetus*	Argentina	Buenos Aires
NCBI	JX111681		UNMDP-T	312		*A. facetus*	*A. facetus*	Argentina	Buenos Aires
NCBI	JX111688		UNMDP-T	360		*A. facetus*	*A. facetus*	Argentina	Buenos Aires
NCBI	JX111684		UNMDP-T	311		*A. facetus*	*A. facetus*	Argentina	Buenos Aires
NCBI	JX111691		UNMDP-T	313		*A. facetus*	*A. facetus*	Argentina	Buenos Aires
NCBI	JX111683		UNMDP-T	315		*A. facetus*	*A. facetus*	Argentina	Buenos Aires
NCBI	JX111685		UNMDP-T	500		*A. facetus*	*A. facetus*	Argentina	Buenos Aires
NCBI	JX111682		UNMDP-T	499		*A. facetus*	*A. facetus*	Argentina	Buenos Aires
NCBI	JX111686		UNMDP-T	501		*A. facetus*	*A. facetus*	Argentina	Buenos Aires
NCBI	JX111690		UNMDP-T	358		*A. facetus*	*A. facetus*	Argentina	Buenos Aires
NCBI	JX111691		UNMDP-T	313		*A. facetus*	*A. facetus*	Argentina	Buenos Aires
NCBI	KJ552359		MUHNAC	4040		*A. facetus*	*A. facetus*	Portugal	Alentejo
NCBI	KJ552539		MUHNAC	4039		*A. facetus*	*A. facetus*	Portugal	Alentejo
BOLD	DSFRE330		No data	No data		No data	*A. tembe*	No data	No data
BOLD	DSFRE200		No data	No data		No data	*A. tembe*	No data	No data
BOLD	NFSB270		UFRGS	19207		*A. acaroides*	*Australoheros* sp.	Brazil	RGS
3		AY9988658	NRM	49554	1179	A. sanguineus	*A. angiru*	Brazil	SC
13		HQ1977709			A24	A. facetus	*A. facetus*	Paraguay	Itapua P09-03
13		HQ1977710			A25	*A. facetus*	*A. facetus*	Paraguay	Itapua P09-04
13		HQ1977711			A26	*A. facetus*	*A. facetus*	Argentina	Corrientes
13		HQ1977712			A27	*A. facetus*	*A. facetus*	Argentina	Corrientes
13		HQ1977703			H18	*A. facetus*	*A. facetus*	Argentina	Catamarca
13		HQ1977704			H19	*A. facetus*	*A. facetus*	Argentina	Catamarca
3		AY843387				*A. facetus*	*A. facetus*	Argentina	Entre Ríos
3		AY998665				*A. facetus*	*A. facetus*	Argentina	Entre Ríos
3		AY998667			Arg.	*A. facetus*	*A. facetus*	Argentina	Entre Ríos

3	AY998666	NRM	49538	1163	*A. facetus*	*A. facetus*	Uruguay	Maldonado
13	HQ197707			A22	*A. forquilha*	*A. forquilha*	Brazil	Rio Grande do Sul
13	HQ197708			A23	*A. forquilha*	*A. forquilha*	Brazil	RGS
13	HQ197686			H1	*A. kaaygua*	*A. kaaygua*	Argentina	No data
3	AY998659	NRM	49555	121	*A. minuano*	*A. acaroides*	Uruguay	Salto
13	HQ197705			A20	*A. scitulus*	*A. scitulus*	Argentina	Misiones
13	HQ197706			A21	*A. scitulus*	*A. scitulus*	Argentina	Misiones
13	HQ197701			H16	*A. scitulus*	*A. scitulus*	Argentina	Corrientes
13	HQ197702			H17	*A. scitulus*	*A. scitulus*	Argentina	Corrientes
3	AY998662	NRM	41626	116	*A. scitulus*	*A. scitulus*	Uruguay	Colonia
3	AY998661	NRM	36435	121	*A. scitulus*	*A. scitulus*	Uruguay	Colonia
3	AY998663	NRM	33048	Arg.	*A. scitulus*	*A. scitulus*	Argentina	Entre Ríos
13	HQ197687			H2	*A. tembe*	*A. tembe*	Argentina	Misiones
13	HQ197688			H3	*A. tembe*	*A. tembe*	Argentina	Misiones
3	AY998660				*A. tembe*	*A. tembe*	Argentina	Misiones
3	AY843373				*A. tembe*	*A. tembe*	Argentina	Misiones
13	HQ197689			H4	*A. ykeregua*	*A. ykeregua*	Argentina	Misiones
13	HQ197690			H5	*A. ykeregua*	*A. ykeregua*	Argentina	Misiones
13	HQ197691			H6	*A. ykeregua*	*A. ykeregua*	Argentina	Misiones
13	HQ197692			H7	*A. ykeregua*	*A. ykeregua*	Argentina	Misiones
13	HQ197693			H8	*A. ykeregua*	*A. ykeregua*	Argentina	Misiones
13	HQ197694			H9	*A. ykeregua*	*A. ykeregua*	Argentina	Misiones
13	HQ197695			H10	*A. ykeregua*	*A. ykeregua*	Argentina	Misiones
13	HQ197696			H11	*A. ykeregua*	*A. ykeregua*	Argentina	Misiones
13	HQ197697			H12	*A. ykeregua*	*A. ykeregua*	Argentina	Misiones
13	HQ197698			H13	*A. ykeregua*	*A. ykeregua*	Argentina	Misiones
13	HQ197699			H14	*A. ykeregua*	*A. ykeregua*	Argentina	Misiones
13	HQ197700			H15	*A. ykeregua*	*A. ykeregua*	Argentina	Misiones
47	MK414409	UFRJ	UFRJ 7770.1		*Ipatinguensis*	*A. autrani*	Brazil	RJ
47	MK414410	UFRJ	UFRJ 7564.1		*Ipatinguensis*	*A. autrani*	Brazil	RJ
47	MK414411	UFRJ	UFRJ 7564.2		*Ipatinguensis*	*A. autrani*	Brazil	RJ
47	MK414412	UFRJ	UFRJ 7564.3		*Ipatinguensis*	*A. autrani*	Brazil	RJ
47	MK414384	UFRJ	UFRJ 8291.1		*A. oblongus (*	*A. barbosae*	Brazil	RJ

47	MK414385	UFRJ	UFRJ 8291.2	*A. oblongus*	*A. barbosae*	Brazil	MG
47	MK414386	UFRJ	UFRJ 8291.3	*A. oblongus*	*A. barbosae*	Brazil	MG
47	MK414387	UFRJ	UFRJ 8291.4	*A. oblongus*	*A. barbosae*	Brazil	MG
47	MK414380	UFRJ	UFRJ 8559.1	*A. oblongus*	*A. barbosae*	Brazil	MG
47	MK414381	UFRJ	UFRJ 8559.2	*A. oblongus*	*A. barbosae*	Brazil	MG
47	MK414382	UFRJ	UFRJ 8559.3	*A. oblongus*	*A. barbosae*	Brazil	MG
47	MK414383	UFRJ	UFRJ 8559.4	*A. oblongus*	*A. barbosae*	Brazil	MG
47	MK414395	UFRJ	UFRJ 9853.1	*A. ipatinguensis*	*A. ipatinguensis*	Brazil	MG
47	MK414396	UFRJ	UFRJ 9853.2	*A. ipatinguensis*	*A. ipatinguensis*	Brazil	MG
47	MK414399	UFRJ	UFRJ 10634.2	*A. oblongus*	*A. macacuensis*	Brazil	RJ
47	MK414397	UFRJ	UFRJ 7567.1	*A. ipatinguensis*	*A. macaensis*	Brazil	RJ
47	MK414398	UFRJ	UFRJ 7567.2	*A. ipatinguensis*	*A. macaensis*	Brazil	RJ
47	MK414373	UFRJ	UFRJ 9484.1	*A. oblongus*	*A. robustus*	Brazil	MG
47	MK414370	UFRJ	UFRJ 9801.1	*A. oblongus*	*A. robustus*	Brazil	MG
47	MK414371	UFRJ	UFRJ 9801.2	*A. oblongus*	*A. robustus*	Brazil	MG
47	MK414372	UFRJ	UFRJ 9801.3	*A. oblongus*	*A. robustus*	Brazil	MG
47	MK414378	UFRJ	UFRJ 8565.1	*A. oblongus*	*A. robustus*	Brazil	RJ
47	MK414379	UFRJ	UFRJ 8565.2	*A. oblongus*	*A. robustus*	Brazil	RJ
47	MK414391	UFRJ	UFRJ 8393.1	*A. ipatinguensis*	*A. muriae*	Brazil	RJ
47	MK414392	UFRJ	UFRJ 8393.2	*A. ipatinguensis*	*A. muriae*	Brazil	RJ
47	MK414393	UFRJ	UFRJ 8393.3	*A. ipatinguensis*	*A. muriae*	Brazil	RJ
47	MK414394	UFRJ	UFRJ 8393.4	*A. ipatinguensis*	*A. muriae*	Brazil	RJ
47	MK414406	UFRJ	UFRJ 7823.1	*A. oblongus*	*A. barbosae*	Brazil	MG
47	MK414407	UFRJ	UFRJ 7823.2	*A. oblongus*	*A. barbosae*	Brazil	MG
47	MK414408	UFRJ	7823.3	*A. oblongus*	*A. barbosae*	Brazil	MG
47	MK414400	UFRJ	7823.4	*A. oblongus*	*A. barbosae*	Brazil	MG
47	MK414401	UFRJ	7887.1	*A. oblongus*	*A. barbosae*	Brazil	MG
47	MK414402	UFRJ	7887.2	*A. oblongus*	*A. barbosae*	Brazil	RJ
47	MK414390	UFRJ	8506.1	*A. ipatinguensis*	*A. ipatinguensis/A. perdi*	Brazil	MG
47	MK414389	UFRJ	8507.1	*A. ipatinguensis*	*A. ipatinguensis/A. perdi*	Brazil	MG
47	MK414388	UFRJ	85081	*A. ipatinguensis*	*A. ipatinguensis/A. perdi*	Brazil	
47	MK414374	UFRJ	9511.1	*A. ipatinguensis*	*A. ipatinguensis/A. capixaba*	Brazil	BA
47	MK414375	UFRJ	9511.2	*A. ipatinguensis*	*A. ipatinguensis/A. capixaba*	Brazil	BA

47	MK414376	UFRJ	9511.3	*A. ipatinguensis*	*A. ipatinguensis/A. capixaba*	Brazil	BA
47	MK414377	UFRJ	9511.4	*A. ipatinguensis*	*A. ipatinguensis/A. capixaba*	Brazil	BA
47	MK414422	UFRJ	10495.1	*A. ribeirae*	*A. ribeirae*	Brazil	SP
47	MK414423	UFRJ	10495.2	*A. ribeirae*	*A. ribeirae*	Brazil	SP
47	MK414424	UFRJ	10495.3	*A. ribeirae*	*A. ribeirae*	Brazil	SP
47	MK414425	UFRJ	10495.4	*A. ribeirae*	*A. ribeirae*	Brazil	SP
47	MK414405	UFRJ	7825.1	*A. ribeirae*	*A. ribeirae*	Brazil	MG
47	MK414403	UFRJ	7828.1	*A. ribeirae*	*A. ribeirae*	Brazil	MG
47	MK414404	UFRJ	7828.2	*A. ribeirae*	*A. ribeirae*	Brazil	MG
47	MK414415	UFRJ	10502.1	*A. sanguineus*	*A. sanguineus*	Brazil	SC
47	MK414416	UFRJ	10502.2	*A. sanguineus*	*A. sanguineus*	Brazil	SC
47	MK414417	UFRJ	10502.3	*A. sanguineus*	*A. sanguineus*	Brazil	SC
47	MK414418	UFRJ	10502.4	*A. sanguineus*	*A. sanguineus*	Brazil	SC
47	MK414413	UFRJ	10605.1	*A. ipatinguensis (AU)*	*A. autrani*	Brazil	RJ
47	MK414414	UFRJ	10605.4	*A. ipatinguensis*	*A. autrani*	Brazil	RJ
47	MK414367	UFRJ	9840.1	*A. oblongus*	*A. barbosae*	Brazil	SP
47	MK414368	UFRJ	9840.2	*A. oblongus*	*A. barbosae*	Brazil	SP
47	MK414369	UFRJ	9802.1	*A. oblongus*	*A. robustus*	Brazil	MG
47	MK414419	UFRJ	10499.1	*A. acaroides*	" "timbé do sul" , A. *minuano*	Brazil	SC
47	MK414420	UFRJ	10499.2	*A. acaroides*	" "timbé do sul" , A. *minuano*	Brazil	SC
47	MK414421	UFRJ	10499.3	*A. acaroides*	" "timbé do sul" , A. *minuano*	Brazil	SC

References:

3 Říčan O, Kullander SO. Character- and tree-based delimitation of species in the *'Cichlasoma' facetum* group (Teleostei, Cichlidae) with the descriptic

13 Říčan O, Piálek L, Almirón A, Casciotta J. Two new species of *Australoheros* (Teleostei: Cichlidae), with notes on diversity of the genus and biogeography of the Río de la Plata basin.Zootaxa, 2011; 2982: 1–26

47 Ottoni, FP, Mattos O, Katz, AM, Bragança PHN. Phylogeny and species delimitation based on molecular approaches on the species of the Australohe

NCBI GenBank database

BOLD Barcode of Life Database

Municipality	Drainage	Lat	Long	Locality
Talpaque	de La Plata	-36.268	-59.98	Tapalque stream
Salado	de La Plata	-35.7792	-57.8792	Salada de Monasterio
Salado	de La Plata	-35.7792	-57.8792	Salada de Monasterio
Salado	de La Plata	-35.7792	-57.8792	Salada de Monasterio
Salado	de La Plata	-35.7792	-57.8792	Salada de Monasterio
Salado	de La Plata	-35.7792	-57.8792	Salada de Monasterio
Salado	de La Plata	-35.7792	-57.8792	Salada de Monasterio
Rojas	de La Plata	-34.0828	-61	Tributary of Rio Rojas
Rojas	de La Plata	-34.0828	-61	Tributary of Rio Rojas
Rojas	de La Plata	-34.0828	-61	Tributary of Rio Rojas
Talpaque	de La Plata	-36268	-59.98	Tapalque stream
Talpaque	de La Plata	-35.7792	-57.8792	Salada de Monasterio
Moura	Guadiana arvão	-7.5	Guiadiana	Guadiana at Vale do Carvão
Moura	Rio Guadianao Carvão	-7.5	Guiadiana	Guadiana at Vale do Carvão
No data	No data	No data	No data	No data
No data	No data	No data	No data	No data
Mostardas	Laguna dos Patos	30° 32' 07.56" S	50° 26' 32.98" W	aprox. 2km antes da vila de Bacupari
	Rio Iguazu			Aquarium stock
	Paraná	27°05'26.16''S	55°53'13.02''W	
	Paraná	27°05'26.16''S	55°53'13.02''W	
	Paraná	28°32'47.28''S	57°11'44.70''W	Laguna Iberá
	Paraná	28°32'47.28''S	57°11'44.70''W	Laguna Iberá
	Paraná	28°08.37''S	65°46'44.30''W	
	Paraná	28°08.37''S	65°46'44.30''W	
	Uruguay			
	Uruguay			Aquarium
	Uruguay			La Reja

La Plata			
Uruguay	27°37'26.34''S	51°45'00.12''W	B902, Rio Forquilha
Uruguay	27°37'26.34''S	51°45'00.12''W	B902, Rio Forquilha
Rio Iguazu	25°37'26.34.79''S	54°05'39.42''W	Ao7-02 Arroyo Lobo
Uruguay			
Uruguay	27°52'33.80''S	55°16'35.07''W	Arroyo Itacruareurare
Uruguay	27°52'33.80''S	55°16'35.07''W	Arroyo Itacruareurare
Uruguay	29°23'49.60''S	58°07'6.12''W	
Uruguay	29°23'49.60''S	58°07'6.12''W	
La Plata	34°19'07''S	59°20'13''W	
La Plata	34°19'07''S	59°20'13''W	
Uruguay	31°53'55''	58°19'55''W	Arroyo El Palmar
Paraná	25°58'26.20''S	54°15'28.78''W	Arroyo Falso Urugua-í
Paraná	25°58'26.20''S	54°15'28.78''W	Arroyo Falso Urugua-í
Paraná			Aquarium, Arroyo Tirica
Paraná	25°58'26.20''S	54°15'28.78''W	
Uruguay	26°45'56.63''S''	54°10''57.43''W	Arroyo Fortaleza
Uruguay	26°45'56.63''S''	54°10''57.43''W	Arroyo Fortaleza
Uruguay	27°14'15.07''	54°02'39.49.38''W	Arroyo Paraíso
Uruguay	27°14'15.07''	54°02'39.49.38''W	Arrpyo Paraíso
Uruguay	27°28'13.83''S	54°'24.52''W	Arroyo Paraíso
Uruguay	27°28'13.83''S	54°'24.52''W	Arroyo Paraíso
Uruguay	27°45'57.5''	55°09'33.75''W	Arroyo Shanghai
Uruguay	27°45'57.5''	55°09'33.75''W	Arroyo Shanghai
Uruguay	27°45'57.5''	55°09'33.75''W	Arroyo Guerrero
Uruguay	27°45'57.5''	55°09'33.75''W	Arroyo Guerrero
Uruguay	27°05'56.53''S	54°45'48.89''W	Arroyo Tamandua
Uruguay	27°05'56.53''S	54°45'48.89''W	Arroyo Tamandua
São João	22° 28' 4.82"S	42° 17' 52.7" W	Rio Aldeia Velha, SilvaJardim
São João	22° 29' 55.5"S	42° 16' 4" W	Rio AldeiaVelha, Silva Jardim
São João	22° 29' 55.5"S	42° 16' 4" W	Rio Aldeia Velha, Silva Jardim
São João	22° 29' 55.5"S	42° 16' 4" W	Rio AldeiaVelha, Silva Jardim
Paraná	20° 38' 23.4"S	46° 59' 1.7" W	Afluente do rioGrande, Cássia

Paraná	20° 38’ 23.4”S	46° 59’ 1.7” W	Afluente do rio Grande, Cássia
Paraná	20° 38’ 23.4”S	46° 59’ 1.7” W	Afluente do rio Grande, Cássia
Paraná	20° 38’ 23.4”S	46° 59’ 1.7” W	Afluente do rio Grande, Cássia
Paraíba do Sul	22° 8’ 28.7”S	44° 10’ 33.3” W	Afluente do rio Bananal, Sta.Rita da Jacutinga
Paraíba do Sul	22° 8’ 28.7S	44° 10’ 33.3” W	Afluente do rio Bananal, Sta.Rita da Jacutinga
Paraíba do Sul	22° 8’ 28.7”S	44° 10’ 33.3” W	Afluente do rio Bananal, Sta.Rita da Jacutinga
Paraíba do Sul	22° 8’ 28.7S	44° 10’ 33.3” W	Afluente do rio Bananal, Sta.Rita da Jacutinga
	19° 11’ 59.5S	42° 27’ 30.2” W	Belo Oriente
	19° 11’ 59.5S	42° 27’ 30.2” W	Belo Oriente
Macacu	22° 27’ 25.9”S	42° 45’ 59.8” W	Rio Guapiçu, Guapimirim
Macaé	22° 13' 6.54"S	41°45'32.60"W	Rio dosQuaranta, BR- 101, Macaé
Macaé	22° 13' 6.54S	41°45'32.60"W	Quaranta, BR- 101, Macaé
São Francisco	20° 17’ 53.5 S	43° 48’ 24.3” W	São Gonçalo do Bação
São Francisco	20° 17’ 53.5”S	43° 48’ 24.3” W	São Gonçalo do Bação
São Francisco	20° 17’ 53.5”S	43° 48’ 24.3” W	São Gonçalo do Bação
São Francisco	20° 17’ 53.5”S	43° 48’ 24.3” W	São Gonçalo do Bação
Paraíba do Sul	22° 20’ 3.38”S	43° 11’ 13.2” W	Rio MariaComprida, Secretário
Paraíba do Sul	22° 20’ 3.38”S	43° 11’ 13.2” W	Rio MariaComprida, Secretário
Paraíba do Sul	21° 17’ 24.92”S	41° 42’ 45.8” W	Rio Palanquim entre Itaperuna e Italva, BR 356, Itaperuna
Paraíba do Sul	21° 17’ 24.92 S	41° 42’ 45.8” W	Rio Palanquim entre Itaperuna e Italva, BR 356, Itaperuna
Paraíba do Sul	21° 17’ 24.92”S	41° 42’ 45.8” W	Rio Palanquim entre Itaperuna e Italva, BR 356, Itaperuna,
Paraíba do Sul	21° 17’ 24.92”S	41° 42’ 45.8” W	Rio Palanquim entre Itaperuna e Italva, BR 356, Itaperuna
Paraíba do Sul	21° 59’ 15.2”S	43° 36’ 37.9” W	Córrego Santa Cecília, Santa Bárbara do Monte Verde,MG
Paraíba do Sul	21° 59’ 15.2”S	43° 36’ 37.9” W	Córrego Santa Cecília, Santa Bárbara do Monte Verde
Paraíba do Sul	21° 59’ 15.2”S	43° 36’ 37.9” W	Córrego Santa Cecília, Santa Bárbara do Monte Verde,
Paraíba do Sul	21° 59’ 15.2”S	43° 36’ 37.9” W	Córrego Santa Cecília, Santa Bárbara do Monte Verde
Paraíba do Sul	22° 33’ 20.8”S	43° 57’ 23.08” W	Riberão MariaPreta, Piraí
Paraíba do Sul	22° 33’ 20.8”S	43° 57’ 23.08” W	Riberão MariaPreta, Piraí
Doce	19° 47’ 10.6”S	42° 34’ 48.3” W	LagoaGambazinho, ParqueEstadual do rio Doce
Rio Doce	19° 47’ 10.6”S	42° 34’ 48.3” W	Lagoa Gambazinho,Parque
Rio Doce	19° 47’ 10.6”S	42° 34’ 48.3” W	Lagoa gambazinho, parque estadualdo rio Doce
Buranhém	16° 24’ 46.7” S	39° 35’ 13.7” W	Eunápolis, rio Buranhém
Buranhém	16° 24’ 46.7”S	39° 35’ 13.7” W	Eunápolis, rio Buranhém

Buranhém	16° 24’ 46.7”S	39° 35’ 13.7” W	Eunápolis, rio Buranhém
Buranhém	16° 24’ 46.7”S	39° 35’ 13.7” W	Eunápolis, rio Buranhém
Ribeira do Iguapebasin	24° 32' 35.08" S	48° 6' 20.56" W	Eldorado
Ribeira do Iguape	24° 32' 35.08"S	48° 6' 20.56" W	Eldorado
Ribeira do Iguapebasin	24° 32' 35.08"S	48° 6' 20.56" W	Eldorado
Ribeira do Iguapebasin	24° 32' 35.08S	48° 6' 20.56" W	Eldorado
Paraíba do Sul	21° 58’ 40.7”S	43° 1’ 47.3” W	Córrego ementre Chiador e Mar de Espanha,Chiador
Paraíba do Sul	21° 57’ 49.7”S;	42° 56’ 2.7” W	Início da MG 126 saindo deSapucaia, Chiador
Paraíba do Sul	21° 57’ 49.7”S;	42° 56’ 2.7” W	Início da MG 126 saindo de SapucaiaChiador
Cubatão	26° 8’ 42.3”S	48 ° 54’ 25.5 W	Joinville, rio Pirabeiraba
Cubatão	26° 8’ 42.3”S	48 ° 54’ 25.5 W	Joinville, rio Pirabeiraba
Cubatão	22° 50’ 7.3”S	48 ° 54’ 25.5 W	Joinville, rio Pirabeiraba
Cubatão	22° 50’ 7.3”S	X 48 ° 54’ 25.5 W	Joinville, rio Pirabeiraba
Saquarema lagunar system	22° 50’ 7.3”S	42° 36’ 4.11” W	Estrada para Tingui,Saquarema
Saquarema lagunar system	22° 50’ 7.3”S	42° 36’ 4.11” W	Estrada para Tingui,Saquarema
Tietê drainage, Paraná	23° 31’ 35.4”S	45° 45’ 53” W	Cachoeira da Porteira Preta no rio Paratinga,Alfredo Rolim de Moura
Tietê drainage, Paraná	23° 31’ 35.4”S	45° 45’ 53” W	Cachoeira da Porteira Preta no rio Paratinga, Alfredo Rolimde Moura
Doce	20° 33’ 15.1”S	43° 37’ 5.5” W	Ouro Branco
Araranguá	28° 50’ 23.2”S	49° 48’ 08” W	Rio Amola Faca, BR 285 entre Timbé doSul e Turvo
Araranguá	28° 50’ 23.2”S	49° 48’ 08” W	Rio Amola Faca, BR 285entre Timbé do Sul e Turvo
Araranguá	28° 50’ 23.2”S	49° 48’ 08” W	Rio Amola Faca, BR 285 entre Timbé do Sul e Turvo

on of a new genus. J. Zool. Syst. Evol. Res. 2006; 44: 136–152.

eros autrani group (Teleostei, Cichlidae), with biogeographic comments. Zoosyst. Evol. 2019; 95 (1): 49–64. doi: 10.3897/zse.95.31658
