# Supplementary material for: Conjectures and refutations: Species diversity and phylogeny of Australoheros from coastal rivers of southern South America (Teleostei: Cichlidae)
Source: PLoS One. 2022 Dec 9;17(12):e0261027. doi: 10.1371/journal.pone.0261027 (PMC9733902; doi:10.1371/journal.pone.0261027)
Supplement: S2 File — (PDF) [file pone.0261027.s002.pdf]

Conjectures and refutations: species diversity and phylogeny of *Australoheros* from coastal rivers of southern South America (Teleostei: Cichlidae)

Supporting material S2

Extralimital species of *Australoheros*

Carlos A. Santos de Lucena[1], Sven Kullander[2], Michael Norén[2], Bárbara Calegari[1,3]

[1]Laboratório de Ictiologia, Museu de Ciências e Tecnologia da Pontifícia Universidade Católica do Rio Grande do Sul, Porto Alegre, Brazil.

[2]Department of Zoology, Swedish Museum of Natural History Stockholm, Sweden.

[3]Department of Vertebrate Zoology, National Museum of Natural History, Smithsonian Institution,
Washington, DC., USA.

The species of *Australoheros* in the Uruguay and lower Paraná drainages were alreadydescribed in some detail [1–5]. Comparative notes are justified not least because of uncertainty about identifications touches both on species in the inland Rio Uruguay basin, and in coastal Brazilian rivers.

Australoheros tembe

'Cichlasoma' tembe Casciotta, Gómez & Toresani, 1995[1]: 193, fig. 1 (holotype MLP 9059; Argentina: arroyo Urugua-í, above Salto del Urugua-í, at 'Alto Paraná' company fields.

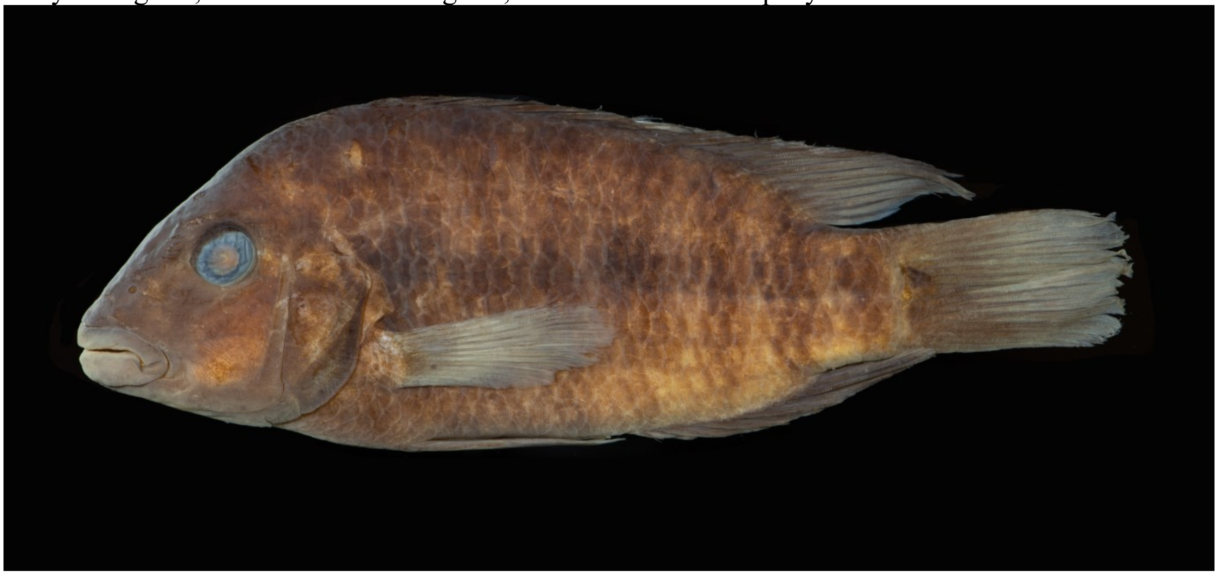

Figure 1. *Australoheros tembe*, adult male, 117.3 mm SL; STRI 2524; Argentina: Misiones: Rio Paraná basin, Arroyo Falso Urugua-i, tributary of the Río Paraná.

Australoheros tembe (Fig. 1) was described before *Australoheros* was erected and before the rapid increase of nominal species of *Australoheros*, and consequently the original description only compared

with *'Cichlasoma' facetum* and a number of Middle American species of "*Cichlasoma"* currently contained in other genera.

Based on data from the original description and STRI specimens[2] (but note that STRI 2467 is a small specimen of *Australoheros kaaygua*), *A. tembe* is characterised by thick upper and lower lips, unique in the genus; mouth far below the orbit; short pectoral and pelvic fins reaching vertical through vent or slightly shorter; long row of minute scales along dorsal-fin base, but shorter than in *A. forquilha*; indistinct vertical bars; indistinct horizontal band formed by blotches on scales in 0 and E1 rows, caudad to Interbar 4; caudal spot small or absent. Bicuspid teeth were not observed.

The morphometric analysis by Casciotta et al. [1] demonstrated longer caudal peduncle, longer snout, and shallower body in *A. tembe* compared to *A. facetus.*

Australoheros tembe has recorded only from the Arroyo Urugua-í, a tributary of the Rio Paraná in Argentina[1,2]. — Material examined: STRI 2524, 1, 117.3 mm SL; Argentina: Misiones: Rio Paraná basin, Arroyo Falso Urugua-i, tributary of the Río Paraná. STRI, no date.
STRI 2514, 2517, 2518, 4, 71.6–99.6 mm SL; Argentina: Misiones: Rio Paraná basin, Arroyo Uruzu, tributary of Arroyo Falso Urugua-i, tributary of the Río Paraná. STRI, no date.

Australoheros scitulus

'Cichlasoma' scitulum Říčan & Kullander, 2003 [3]: 794, fig. 1 ([holotype NRM 36647, now in Museo Nacional de Historia Nacional, Montevideo; Uruguay, Departamento Colonia, Río de La Plata basin, Río Rosario drainage, Arroyo Colla, 500 m upstream from mouth into Río Rosario, upstream of Paso Arballo (34°19'7"S 59°20′13"W).

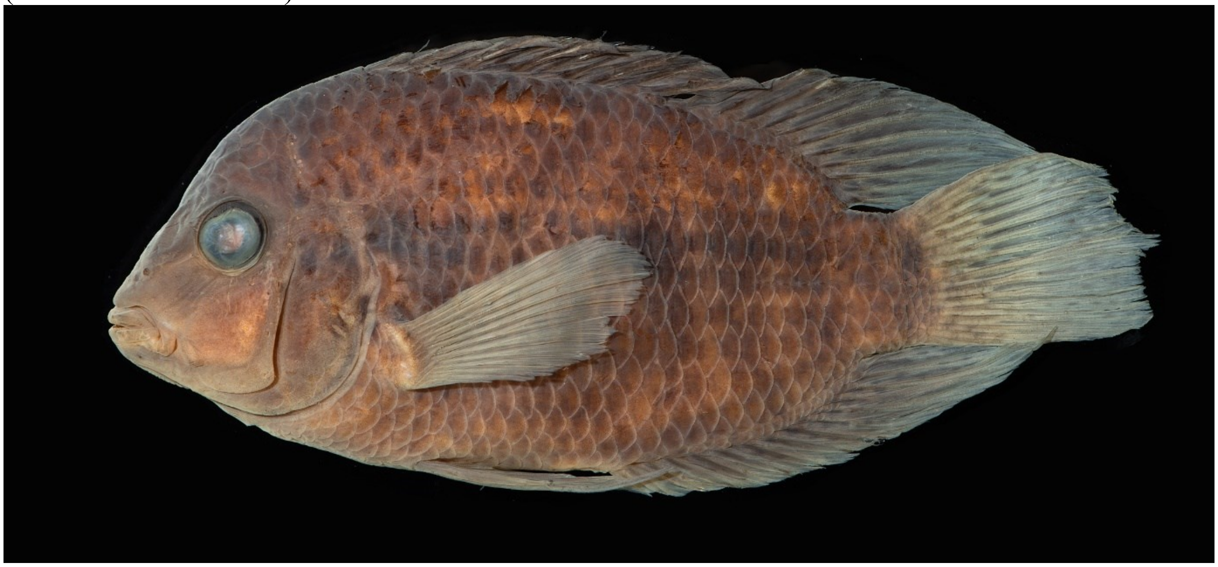

Figure 2. *Australoheros scitulus*, paratype, not sexed, 102.0 mm SL; NRM 36465; Uruguay: Colonia: Rio Rosario drainage: Arroyo Colla.

Australoheros scitulus (Fig. 2) is a distinctive species with a wide distribution in the lower Rio Uruguay basin from the La Plata region to the Quaraí River in Brazil. It is characterised by autapomorphic dark spots on the side of the head, and further distinguished from most other species of *Australoheros* by more dorsal- and anal-fin spines (16–17 and 8–9, respectively), and 14 caudal vertebrae (Říčan and Kullander[2, 3]. The lateral band is restricted in width to the E1 scale row, ending with Interstripe 4, but extended by

diffuse blotches in bars 3 and 2. The midlateral blotch is large, wider than Bar 4 and extending below and above the midlateral band. The caudal spot is restricted to the dorsal lobe. Bicuspid teeth were not observed. —Material examined Uruguay: NRM 43136, 5; 36465, 1; 36647, 3; 33048, 1; 36638, 1; 40063, 10; 36435, 1;39533, 7; 36866, 11; 36671, 7; 39514, 1. NRM 55104, 1, juvenile; Tacuarembó: Las Toscas: Arroyo Caraguatá at Los Talas, under bridge, 32°09'28.4"S 55°01'28.3"W; 27 Oct 2005; S. Kullander et al.— Brazil: MCP 13589, 5, 43.3–72.2 mm SL; 13588, 6, 35.6–96.6 mm SL.

Australoheros kaaygua

Australoheros kaaygua Casciotta, Almirón & Gómez, 2006[4]6: 77, fig 1. (Holotype, MACN-ict 8917; Argentina, Misiones, río Iguazú basin, arroyo Ñandú.)

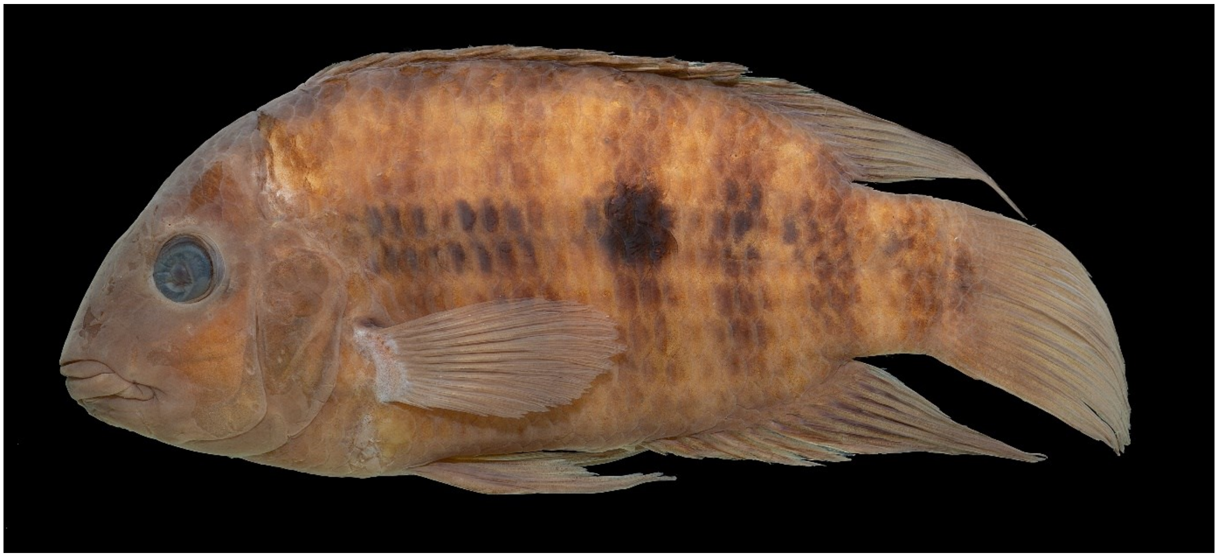

Figure 3. *Australoheros kaaygua,* adult male, 116.4 mm SL; NUP 1839; Brazil: Paraná: Salto do Lontra: Rio Jaracatiá (upstream), tributary of the Rio Iguaçu.

Australoheros kaaygua (Fig. 3) was the first species of the genus to be described from the Rio Iguaçu drainage, and is so far recorded only from the lower Rio Iguaçu drainage. It is similar to *Australoheros acaroides,* and *A. minuano* in colour pattern, meristic data, and morphometric data. Říčan and Kullander[2] (; fig. 13) identified a species from the upper Rio Uruguay basin as *A. kaaygua.* Říčan et al.[5] revised the identification and described the Uruguayan species as *Australoheros angiru*, but did not add any morphological data. The colour pattern includes a zipper type lateral band on scales in Rows 0 and E1 to Interbar 4, wide and rounded midlateral spot in Bar 4, and dark paired spots in E1 and E2 scales in Bars 2 and 3. The midbasal caudal blotch may be indistinct or small, rounded or as a vertical stripe. Bicuspid teeth were not observed. — Material examined: STRI 2467, 1, 50.8 mm. SL. Argentina: Misiones: Rio Paraná drainage: Yacuy: Arroyo Uruzu. STRI, no date.
NUP 700, 1, 113.2 mm SL; Brazil: Paraná: Três Barras do Paraná, Rio Adelaide, tributary of the Rio Iguaçu, 25°18’01"S 53°11"28W; Nupelia, 13 Jan 1998.

Australoheros forquilha

Australoheros forquilha Říčan & Kullander, 2008[2]:14, fig. 4 (holotype MCP 13936; Brazil, Rio Grande do Sul. Rio Forquilha, Rio Uruguai drainage, road from Maximiliano de Almeida to Machadinho).

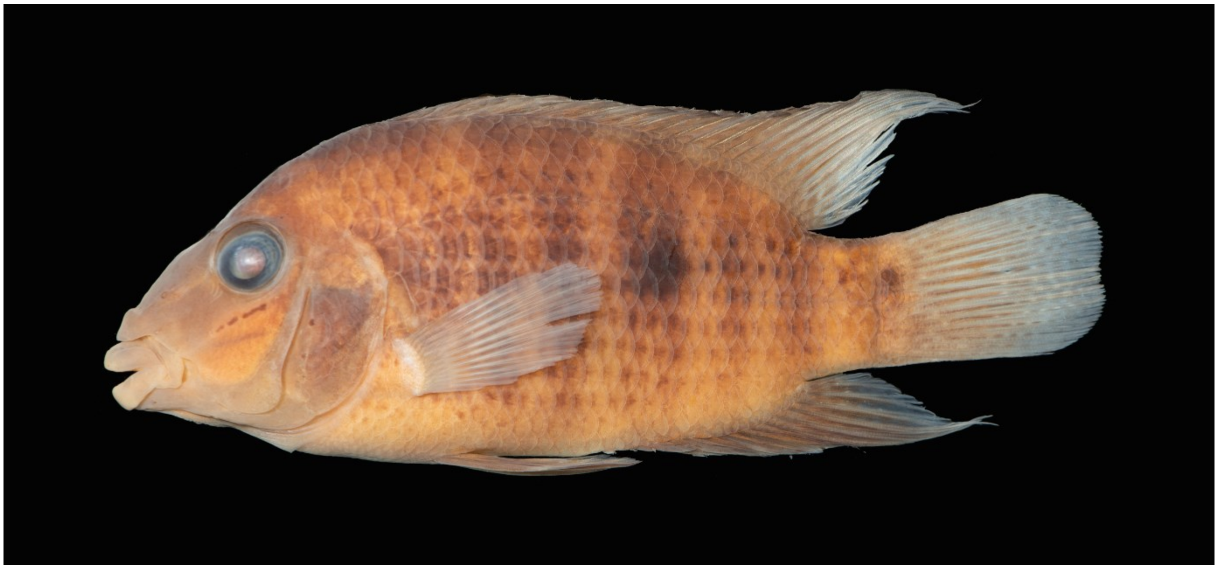

Figure 4. *Australoheros forquilha*, adult female, 92.3 mm SL; NRM 13389; Brazil, Rio Grande do Sul; Maximiliano Almeida; Rio Uruguay drainage: Rio Forquilha on road Maximiliano de Almeida–Machadinho.

Australoheros forquilha (Fig. 4) is probably the most distinct species in the genus, characterised by a checkerboard pattern of dark spots on unpaired fins; short dark stripes along the posterolateral border of the infraorbital series; relatively thick lips; short pectoral and pelvic fins reaching at most to base of first anal-fin spine; row of minute scales along dorsal fin base reaching to anterior insertion of dorsal fin; caudal fin subtruncate; long caudal peduncle containing 1½–3 vertebrae; caudal spot minute or absent; abdominal bars 3–4. The original description included specimens from Soberbio that were identified as *A. ykeregua* by Říčan et al. [5]. There are no records of *A. forquilha* beyond the type series and the tissue sample listed by Říčan et al.[5].—
Material examined**:** Same as in Říčan and Kullander [2].

Australoheros guarani

Australoheros guarani Říčan & Kullander, 2008:[2] 38, fig. 16 (holotype MHNG 2237.58; Paraguay, Caaguazú, Río Guyrau-gua, Río Paraná drainage, km 197 on road from Asunción to Stroessner).

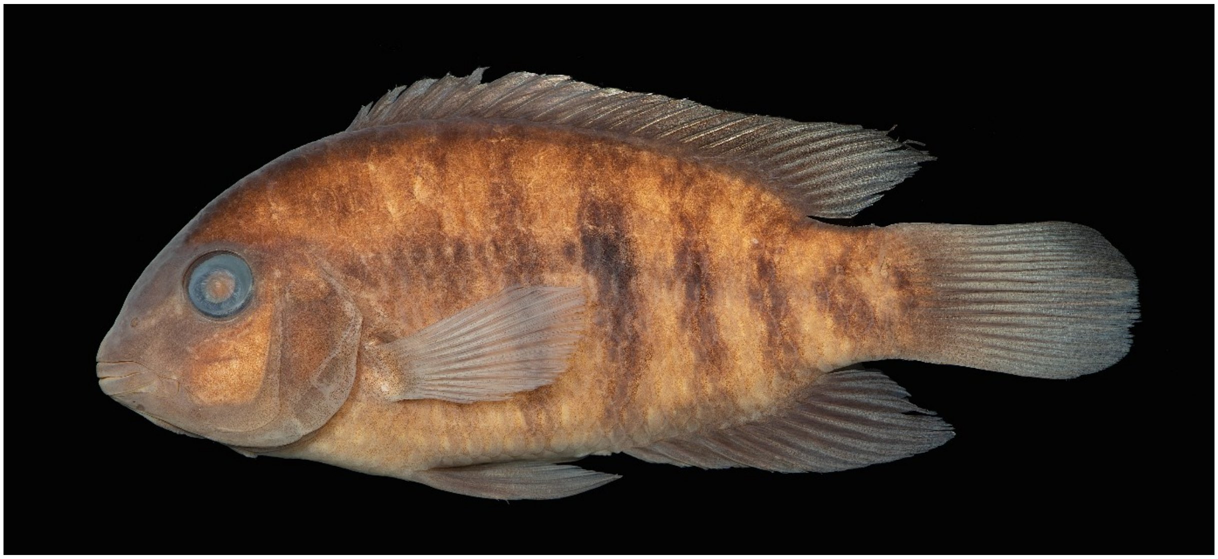

Figure 5. *Australoheros guarani*, adult female, 102.8 mm SL; NRM 42269; Paraguay: Alto Paraná: Cruce: Rio Paraná drainage: Arroyo Agua Fria, left hand side, about 4.5 km from Ruta 6 on road to San Alfredo.

Australoheros guarani (Fig. 5) has been recorded only from widely separated tributaries of the Rio Paraná in Paraguay. The original description was based on specimens from the Rio Guyrau-guá (tributary of the Rio Monday), Arroyo Tembey, an unnamed tributary of the Rio Monday, and Rio Tembey below the falls; two more NRM specimens from the Rio Monday drainage were available at the time but obviously not recognised. Říčan and Říčanová[6] published images of living specimens and a tree from an mtDNA analysis including also the sequences from Říčan et al.[5]. The tree shows *A. guarani* in a cluster together with *A. kaaygua*, and *A. tembe* (sister species, but very short branch). This result is different from the tree in Říčan et al.[13] : fig. 2), where *A. kaaygua* is sister to *A. angiru* [=*A. sanguineus*], *A. minuano* [*A. acaroides*], *A. tembe,* and *A. facetus* from Argentina, Paraguay and Uruguay. The records of *A. facetus* from Itapúa suggest that *A. facetus* and *A. guarani* may be sympatric in Paraguay. The original diagnosis highlighted similarity to *A. facetus* and *A. minuano* in colour pattern. The diagnosis referred to unique large scales between the dorsal fin and upper lateral line (two large and one small anteriorly, vs more; only one large scale posteriorly vs of more). Too few specimens are available for evaluating morphometric data. Although Říčan and Kullander[4] and Říčan and Říčanová [6] emphasised the deep body of *A. guarani*. NRM 42269, from the Rio Monday drainage, is relatively slender (Fig. 5). It was obtained from a fast running stream in the high water season.— Material examined: Same as in Říčan and Kullander[2]; NRM 42269, 1, 102.8 mm SL; Paraguay: Alto Paraná: Cruce: Rio Paraná drainage, Arroyo Agua Fria, left hand side, about 4.5 km from Ruta 6 on road to San Alfredo, 26°08'58.9"S 55°11'47.8"W**;** S. Kullander et al., 2 Feb 1998**.**

Australoheros charrua

Australoheros charrua Říčan & Kullander, 2008[2]: 23, fig. 11 (holotype MCP 13938; Brazil, Rio Grande do Sul, Arroyo Canoin, Rio Uruguai drainage, road from Pirapo to São Nicolau).

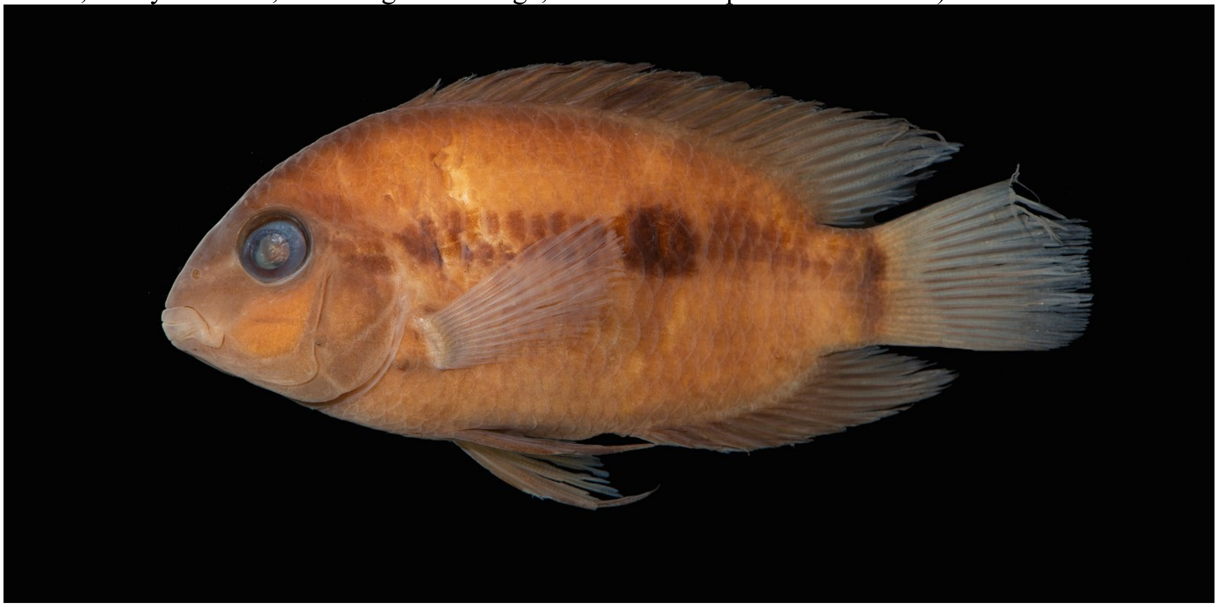

Figure 6. *Australoheros charrua*, paratype, adult female, 70.5 mm SL; NRM 12667; Brazil: Rio Grande do Sul: Rio Uruguay drainage: Arroio Canoin, road from Pirapo to São Nicolau.

Australoheros charrua is known only from eight specimens collected in the Rio Canoin, a tributary of the Rio Piratini, which drains to the Rio Uruguay some distance up from the mouth of the Rio Quaraí. The species was referred to as *Australoheros* "Pirapo" by Říčan and Kullander[7].

Říčan and Kullander [2] considered *A. charrua* to be most similar to *A. scitulus* (p. 23), and to *A. scitulus* and *A. kaaygua* (p. 25), based on the relatively deep body, small mouth, short caudal peduncle, and colouration including a wide, contrasted midlateral band, and very large midlateral blotch. The lateral band consists of blotches on scales in the 0 and E scale rows, extending posterior to Interbar 4. The midlateral bloch is much wider than Bar 4 and separated from Bar 3 by Interbar 3. In some specimens there are dark spots in E2 and E3 scales in Bar 3 and Bar 2; this pattern was described by Říčan and Kullander [2] as a shift in the position of the body bars, present in *A. charrua, A. kaaygua,* and *A. minuano* but specifically in *A. charrua* as 'posterior from the midlateral blotch, the [midlateral] stripe is better described as a series of two blotches, the anterior one being centered in the E2 scale row, the posterior one in the E3 scale row'. Říčan and Kullander [2] distinguished *A. charrua* from *A. scitulus* also by lower number of dorsal-fin spines (16 vs 17), and anal-fin spines (7 vs 8–9). Counts verified were D. XVI.9 (1), XVI.10 (3), XVII (2), XVII.10 (2) (fin count not possible in one specimen with damaged dorsal fin) and A. VII.7 (3), VII.8 (5). The caudal spot (Bar 1p) is very narrow, marking the end of the caudal peduncle as a black vertical stripe across the middle of the caudal-fin base, or the dorsal lobe part slightly wider or darker than the ventral lobe part. Bar 1a, typically a dark marking on the middle of the caudal peduncle is absent. The state of Bar 1 may be an autapomorphy of *A. charrua* The only recorded difference from the sympatric *A. minuano* may be the much larger and rounded caudal blotch. The caudal fin is rounded, tending to subtruncate in *A. charrua*, but definitely rounded in *A. minuano.* Bicuspid teeth were not observed. —Material examined: Same as in Říčan and Kullander, 2008 [2].

Australoheros angiru

Australoheros angiru Říčan, Piálek, Almirón & Casciotta, 2011[13]: 15, fig. 8 (holotype MCP 13937; Brazil, Santa Catarina State, rio Uruguai drainage, rio Jacutinga, road BR 283 from Ceará to Concordia). *Australoheros* jacutinga Říčan & Kullander, 2006[7]: 145. Name only, not made available here; referred elsewhere in paper as *Australoheros* sp. jacutinga.

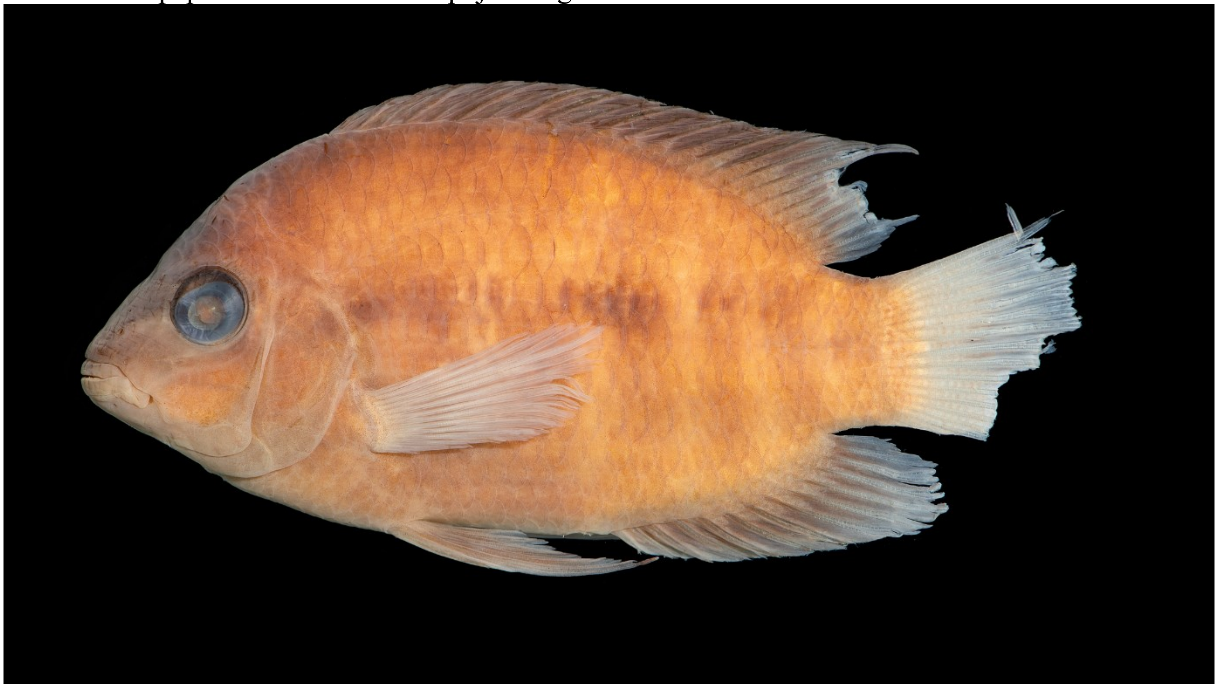

Figure 7. *Australoheros angiru*, paratype, adult female, 77.0 mm SL; NRM 13383; Brazil: Santa Catarina: Rio Uruguay drainage: Rio Jacutinga, BR-283, road Seara-Concórdia.

Říčan et al.[5] described *A. angiru* on the basis 27 specimens from the upper Rio Uruguay in Santa Catarina, previously identified as *A. kaaygua* by Říčan and Kullander[2]; but listed also specimens excluded from the type series from Soberbio on the middle Rio Uruguay in Argentina; specimens mentioned from the Rio Iguaçu basin represent *A. sanguineus*.
Australoheros angiru is a relatively deep-bodied species (Fig 7), somewhat similar to *A. charrua,* but with smaller midlateral spot, contained in the lateral band. In the light of recorded misidentification, this species needs revision—Material examined: Same as in Říčan and Kullander[2].

Australoheros ykeregua

Australoheros ykeregua Říčan, Piálek, Almirón & Casciotta, 2011[5]: 9, fig. 4 (holotype MACN-ict 9467. Argentina, río Uruguay basin, arroyo Paraiso (or Canal Muerto), 27°14'15.1"S,54°02'38.5"W).

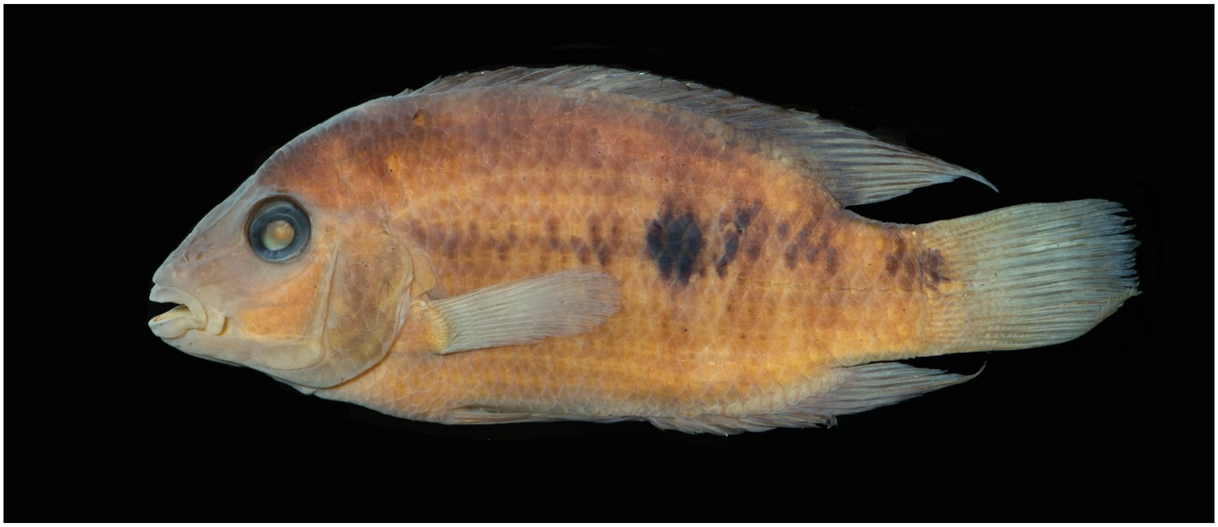

Fig 8. *Australoheros ykeregua*, adult female, 76.7 mm SL; STRI 2559; Argentina: Misiones: Rio Uruguay drainage: Arroyo Acaraguá.

Australoheros ykeregua (Fig 8.) was described on the basis of specimens from Argentinian tributaries of the upper Rio Uruguay between the Arroyo Paraíso and the Arroyo Tamandua.It is an elongate species (Fig. 8), similar only to *A. forquilha* by presence of dark (opalescent pale blue in life) spots along the posterolateral margin of the infraorbital series, and checkerboard pattern of spots in the unpaired fins. — Material examined. Same as in Říčan and Kullander[4](as *Australoheros forquilha*); and STRI 2559.

Literature cited

1. Casciotta JR, Gómez S.E, Toresani, NI. '*Cichlasoma' tembe*, a new cichlid species from the río Paraná basin, Argentina (Osteichthyes, Labroidei). Ichthyol.Explor. Freshw. 1995; 6:193–200.
2. Říčan O, Kullander SO. The *Australoheros* (Teleostei: Cichlidae) species of the Uruguay and Paraná River drainages. Zootaxa 2008; 1724: 1–51.
3. Říčan O, Kullander SO.*Cichlasoma' scitulum*: a new species of cichlid fish from the Río de La Plata Region in Argentina, Brazil, and Uruguay. Copeia 2003; 2003: 794–802.
4. Casciotta, JR, Almirón AE, Gómez E. A new species of *Australoheros* (Teleostei: Perciformes: Cichlidae) from the río Iguazú basin, Argentina. Zool. Abh. Dresden. 2006; 55: 77–83. Říčan O, Piálek L, Almirón A, Casciotta J. Two new species of *Australoheros* (Teleostei: Cichlidae), with notes on diversity of the genus and biogeography of the Río de la Plata basin. Zootaxa, 2011; 2982: 1–26.
5. Říčan O, Kullander SO. *'Cichlasoma' scitulum*: a new species of cichlid fish from the Río de La Plata Region in Argentina, Brazil, and Uruguay. Copeia 2003; 2003: 794–802.
6. Říčan, O, Říčanová, Š. 2017. Live coloration, habitat, biogeography and phylogenetic position of *Australoheros guarani* Říčan & Kullander, 2008 (Teleostei: Cichlidae). Ichthtyological contributions of Peces Criollos 57:1–6.
7. Říčan O, Kullander SO. Character- and tree-based delimitation of species in the *'Cichlasoma' facetum* group (Teleostei, Cichlidae) with the description of a new genus. J. Zool. Syst. Evol. Res. 2006; 44: 136–152.
