# Supplementary material for: Conjectures and refutations: Species diversity and phylogeny of Australoheros from coastal rivers of southern South America (Teleostei: Cichlidae)
Source: PLoS One. 2022 Dec 9;17(12):e0261027. doi: 10.1371/journal.pone.0261027 (PMC9733902; doi:10.1371/journal.pone.0261027)
Supplement: S3 File — (PDF) [file pone.0261027.s003.pdf]

Conjectures and refutations: species diversity and phylogeny of *Australoheros* from coastal rivers of southern South America (Teleostei: Cichlidae)

Supporting material S3. Box and whisker plots

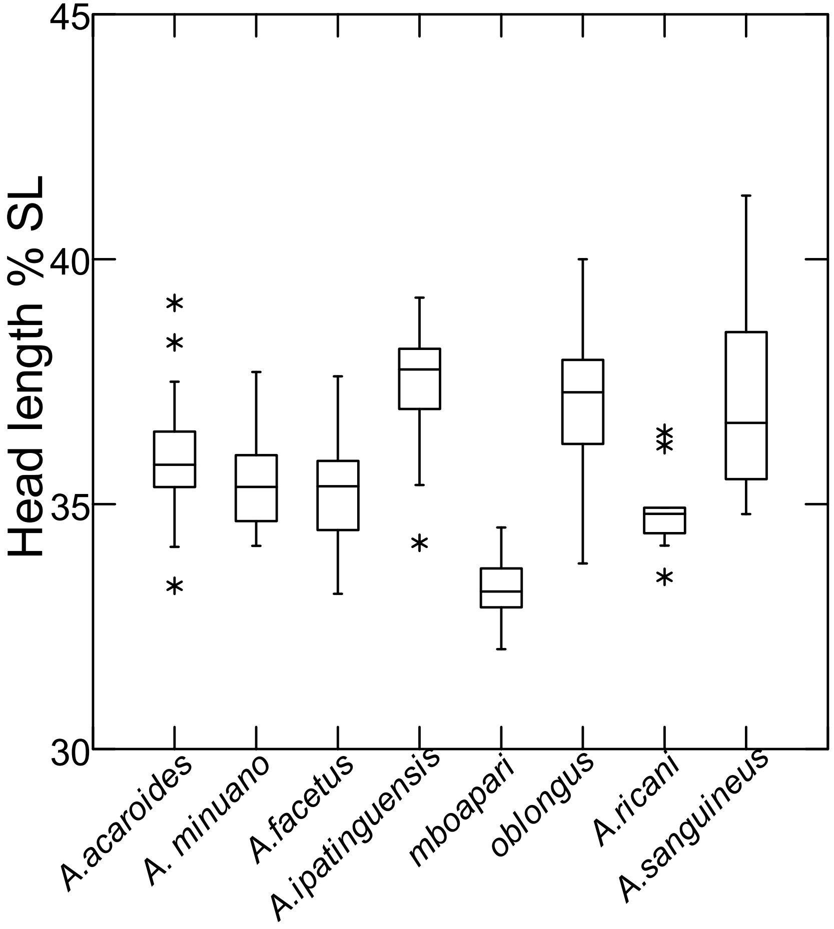

Figure 1. Box and whisker plot of head length in percent of SL within shared standard length interval (40.2–84.2 mm) in coastal species of *Australoheros*.

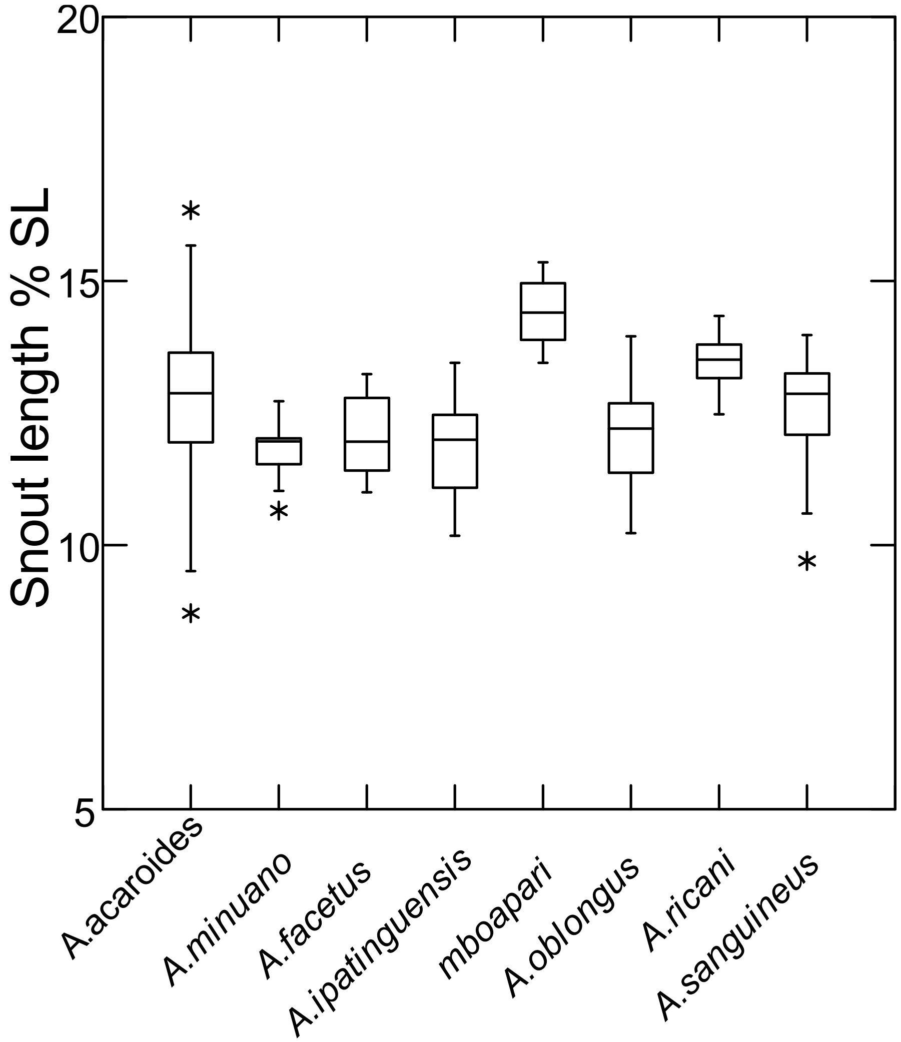

Figure 2. Box and whisker plot of snout length in percent of SL within shared standard length interval (40.2–84.2 mm) in coastal species of *Australoheros.*

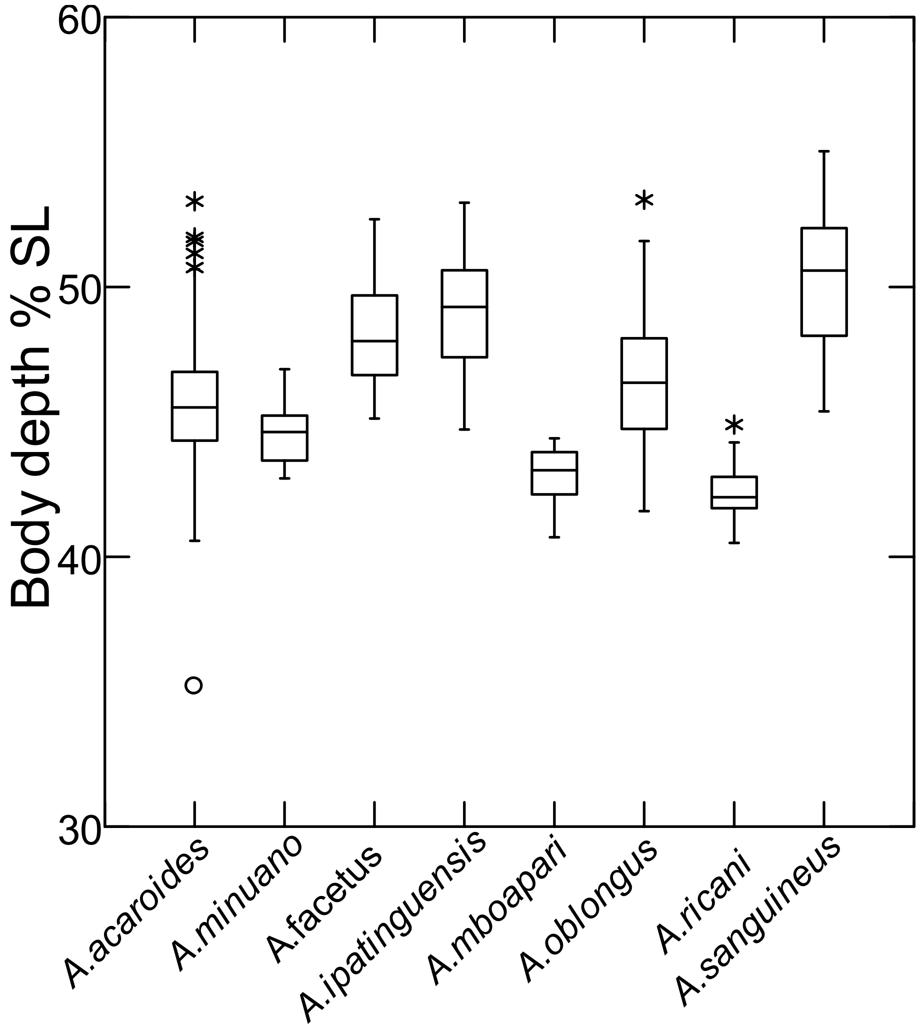

Figure 3. Box and whisker plot of body depth in percent of SL within shared standard length interval (40.2–84.2 mm) in coastal species of *Australoheros*.

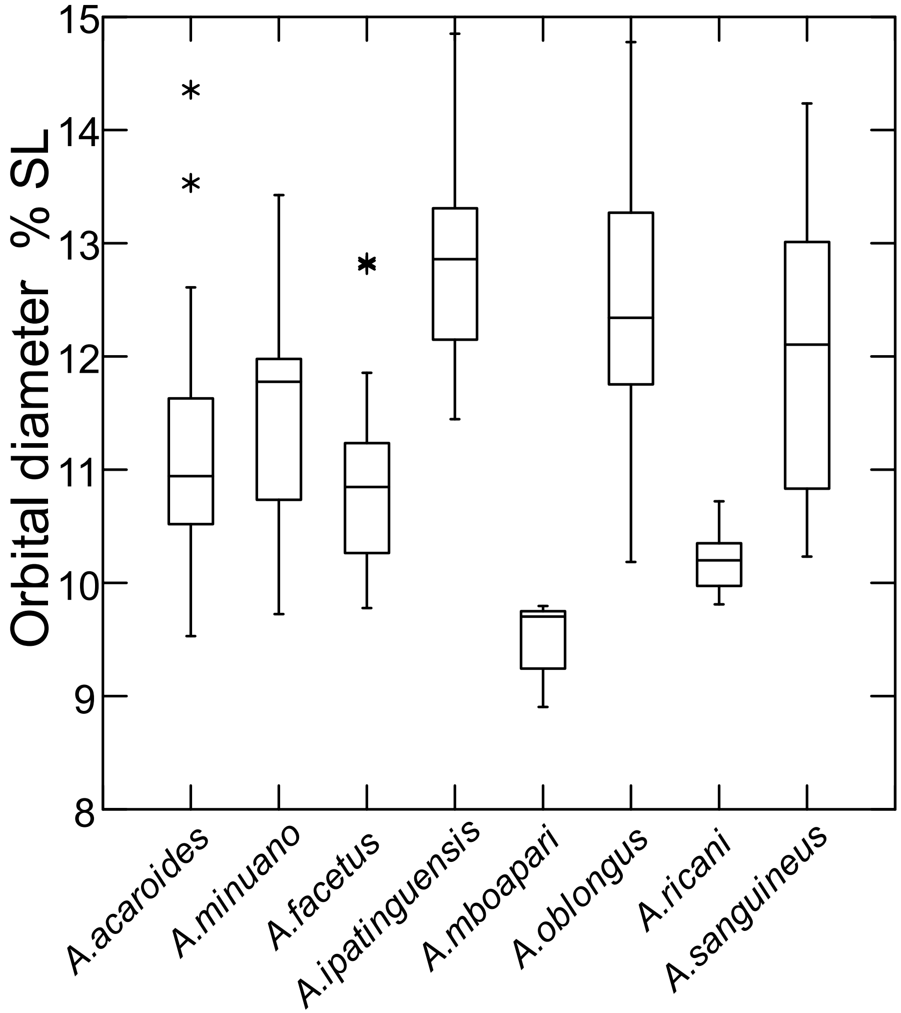

Figure 4. Box and whisker plot of orbital diameter in percent of SL within shared standard length interval (40.2–84.2 mm) in coastal species of *Australoheros*.

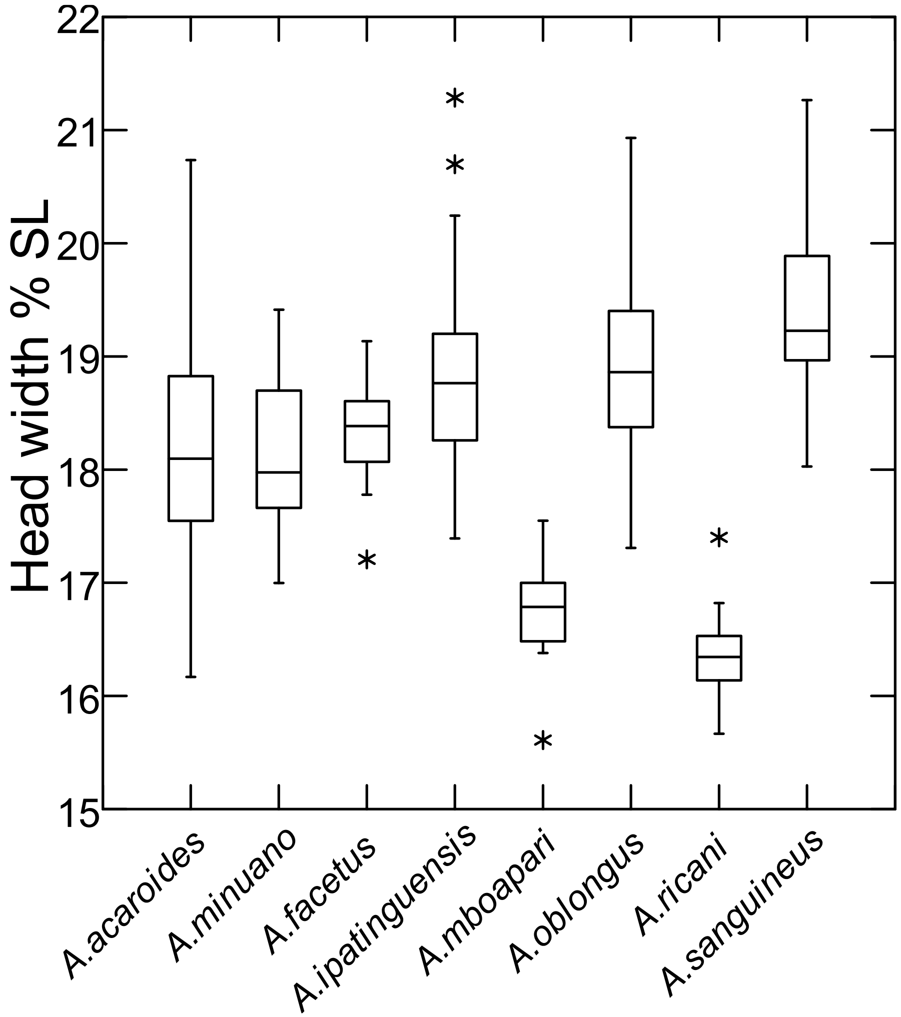

Figure 5. Box and whisker plot of head width in percent of SL within shared standard length interval (40.2–84.2 mm) in coastal species of *Australoheros*.

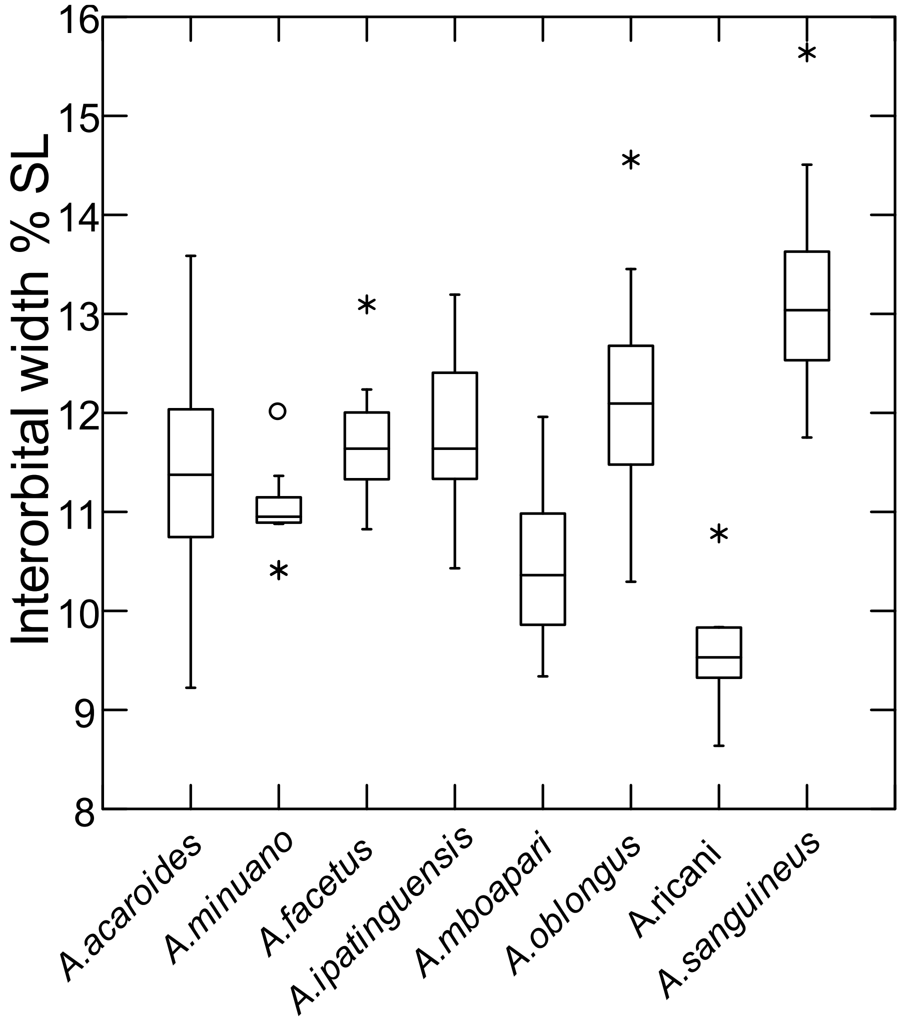

Figure 6. Box and whisker plot of interorbital width in percent of SL within shared standard length interval (40.2–84.2 mm) in coastal species of *Australoheros.*

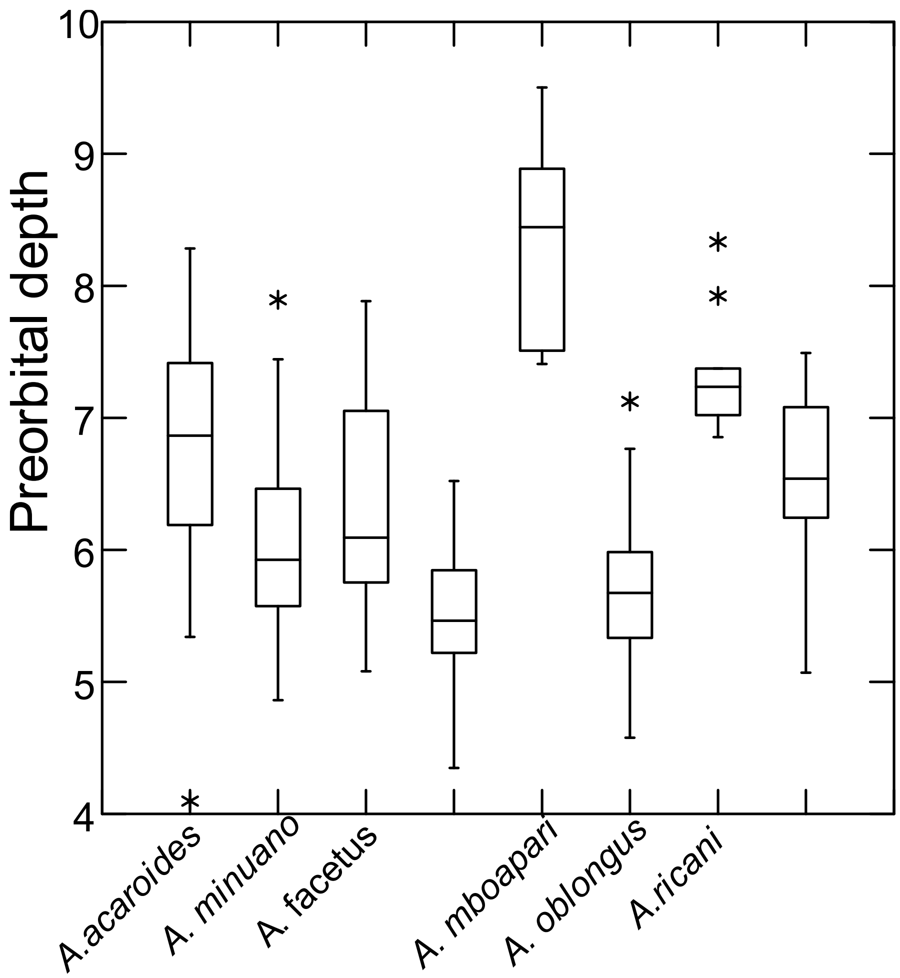

Figure 7. Box and whisker plot of preorbital depth in percent of SL within shared standard length interval (40.2–84.2 mm) in coastal species of *Australoheros*.

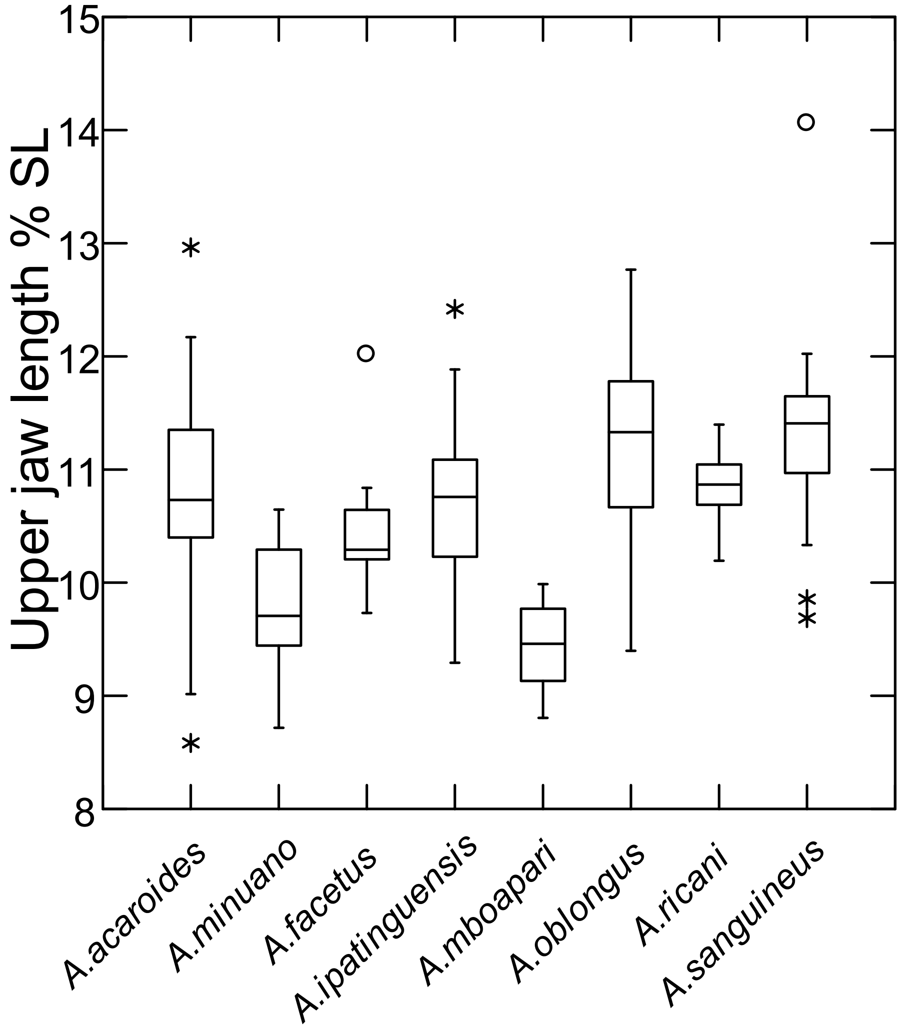

Figure 10. Box and whisker plot of upper jaw length in percent of SL within shared standard length interval (40.2–84.2 mm) in coastal species of *Australoheros.*

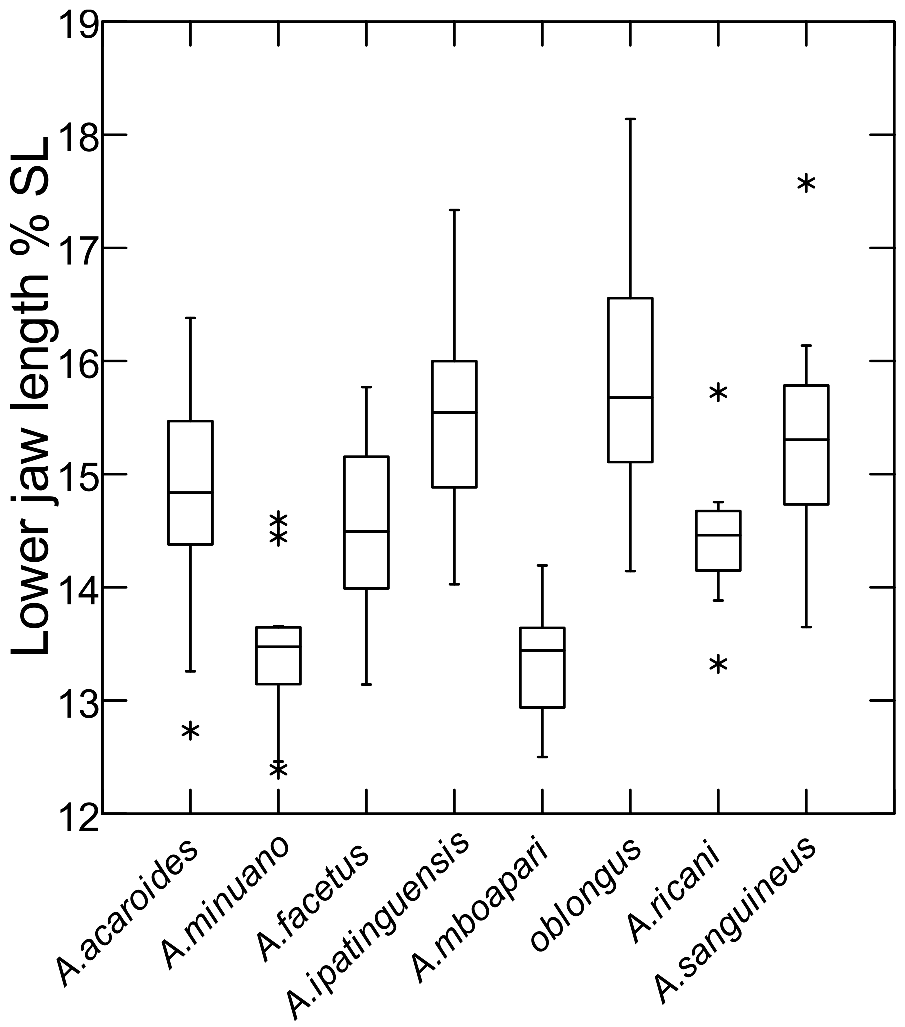

Figure 8. Box and whisker plot of lower jaw length in percent of SL within shared standard length interval (40.2–84.2 mm) in coastal species of *Australoheros.*

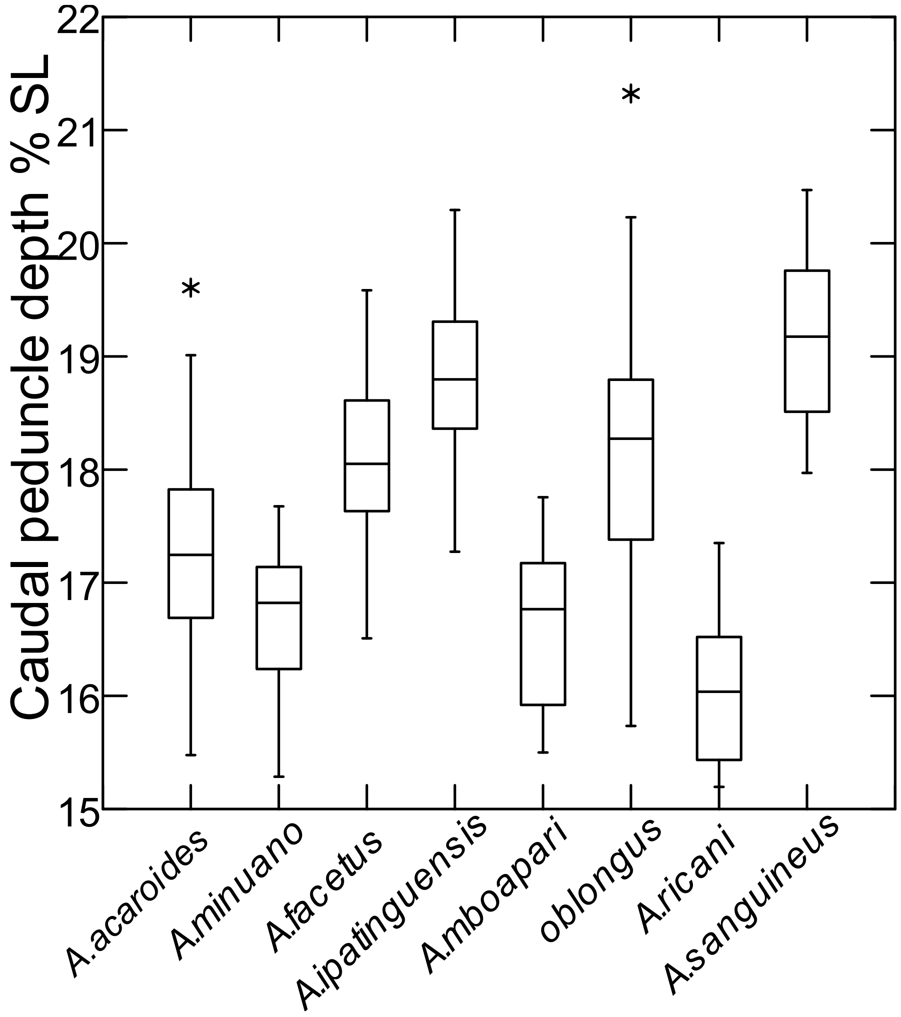

Figure 9. Box and whisker plot of caudal peduncle depth in percent of SL within shared standard length interval (40.2–84.2 mm) in coastal species of *Australoheros.*

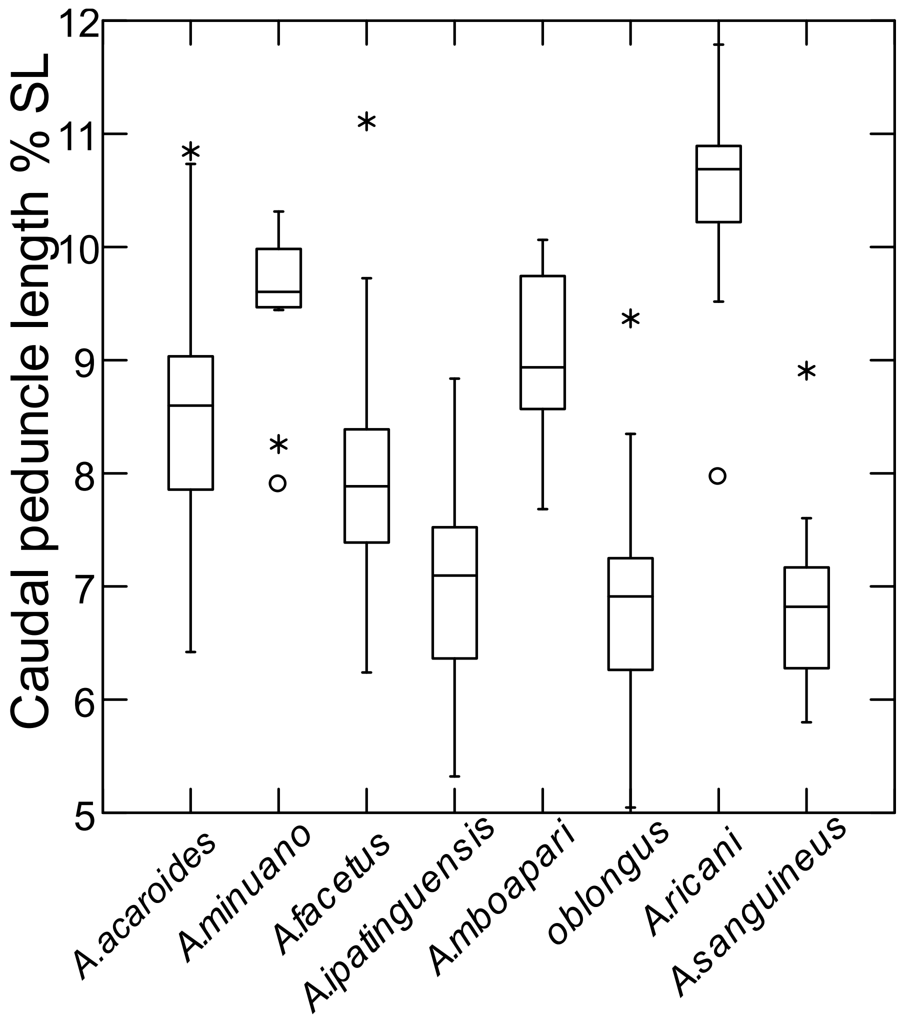

Figure 6. Box and whisker plot of caudal peduncle length in percent of SL within shared standard length interval (40.2–84.2 mm) in coastal species of *Australoheros.*

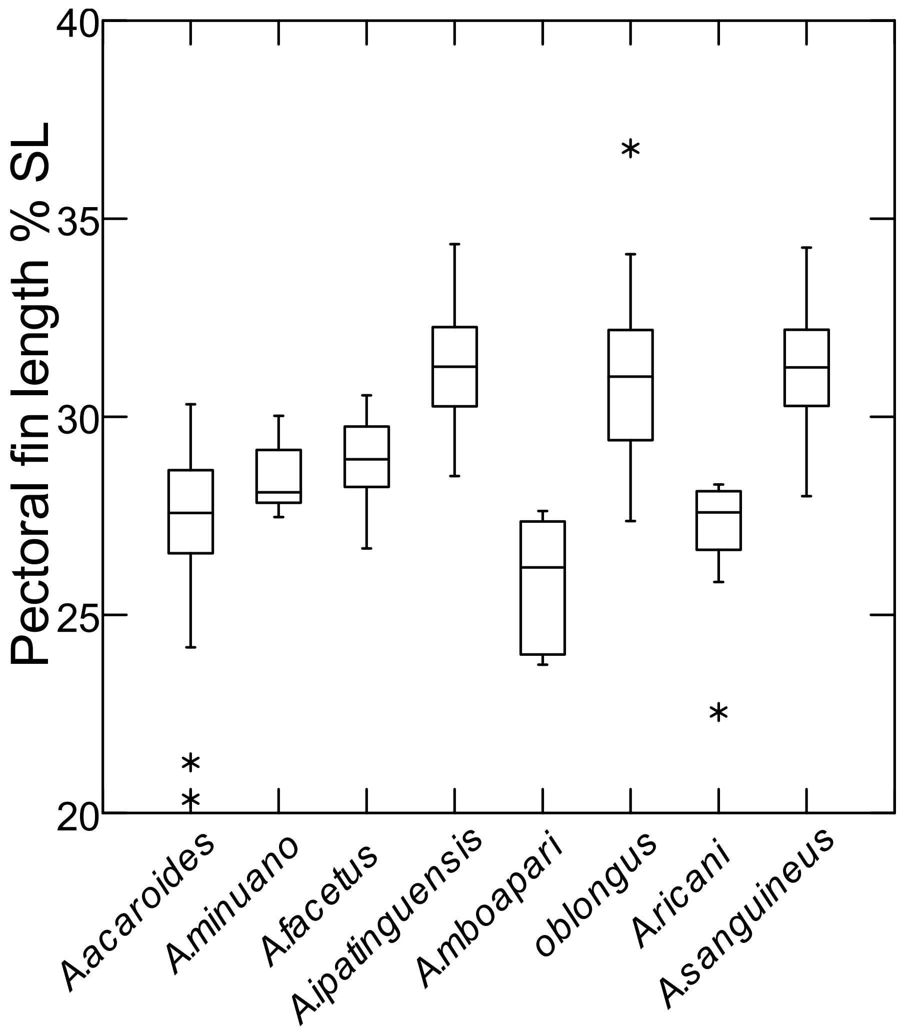

Figure 7. Box and whisker plot of pectoral fin length in percent of SL within shared standard length interval (40.2–84.2 mm) in coastal species of *Australoheros.*

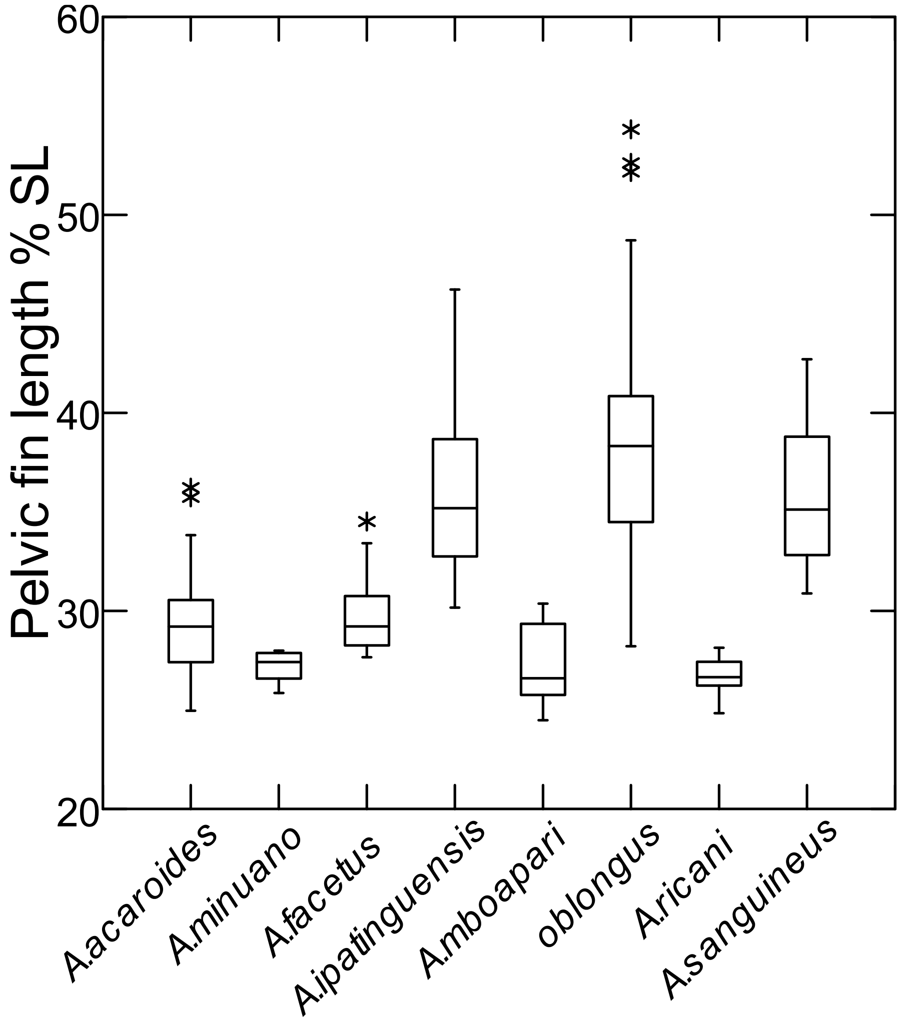

Figure 8. Box and whisker plot of pelvic fin length in percent of SL within shared standard length interval (40.2–84.2 mm) in coastal species of *Australoheros*.

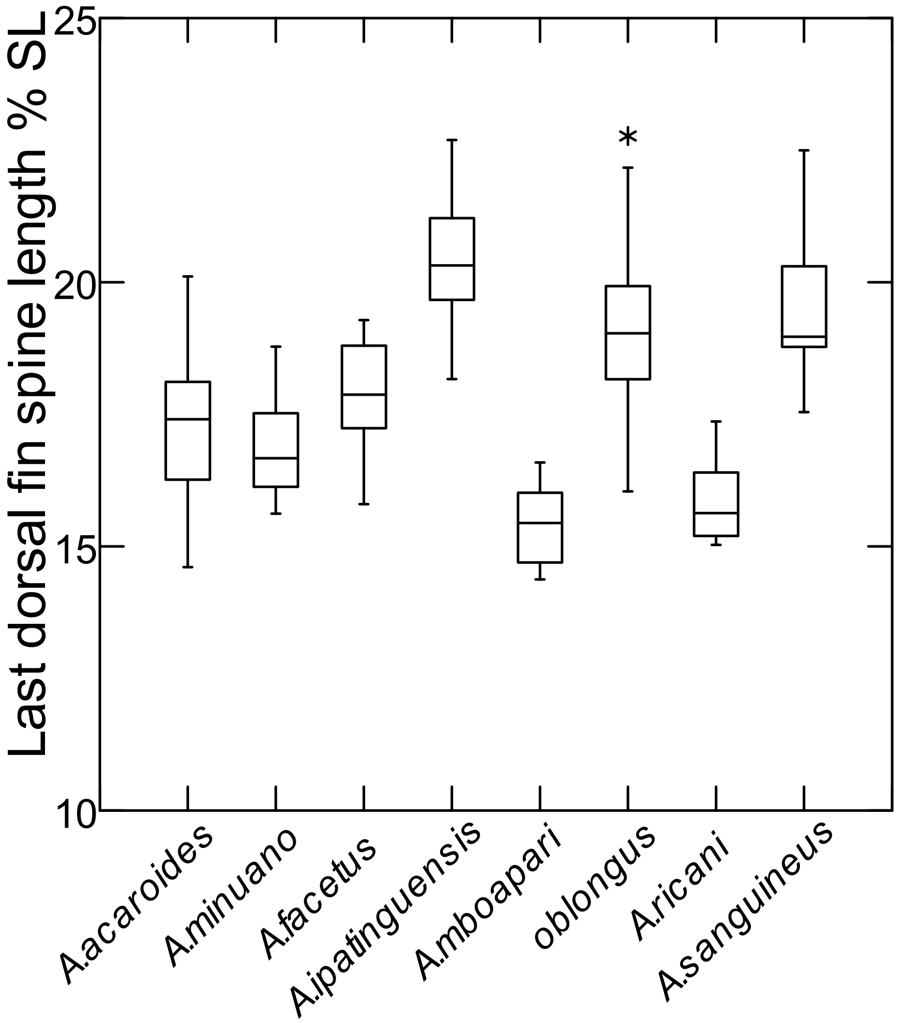

Figure 9. Box and whisker plot of last dorsal-fin spine length in percent of SL within shared standard length interval (40.2–84.2 mm) in coastal species of *Australoheros.*

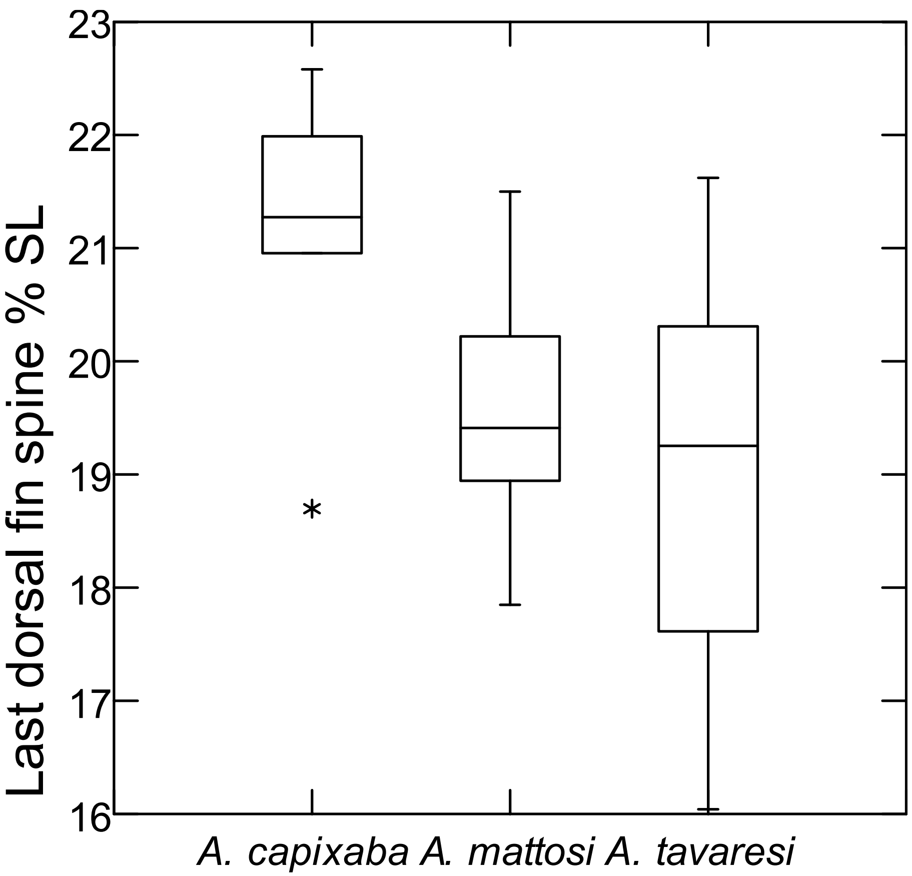

Figure 10. Box and whisker plot of last dorsal-fin spine length in percent of SL in nominal species *A. capixaba A.*, *A. mattosi*, and *A. tavaresi*.
