# Supplementary material for: Conjectures and refutations: Species diversity and phylogeny of Australoheros from coastal rivers of southern South America (Teleostei: Cichlidae)
Source: PLoS One. 2022 Dec 9;17(12):e0261027. doi: 10.1371/journal.pone.0261027 (PMC9733902; doi:10.1371/journal.pone.0261027)
Supplement: S4 File — (DOCX) [file pone.0261027.s004.docx]

**Conjectures and refutations: species diversity and phylogeny of *Australoheros*  from coastal rivers of southern South America (Teleostei: Cichlidae)**

**Supporting material S4. Full set of plots and tables from Principal Component Analyses.**


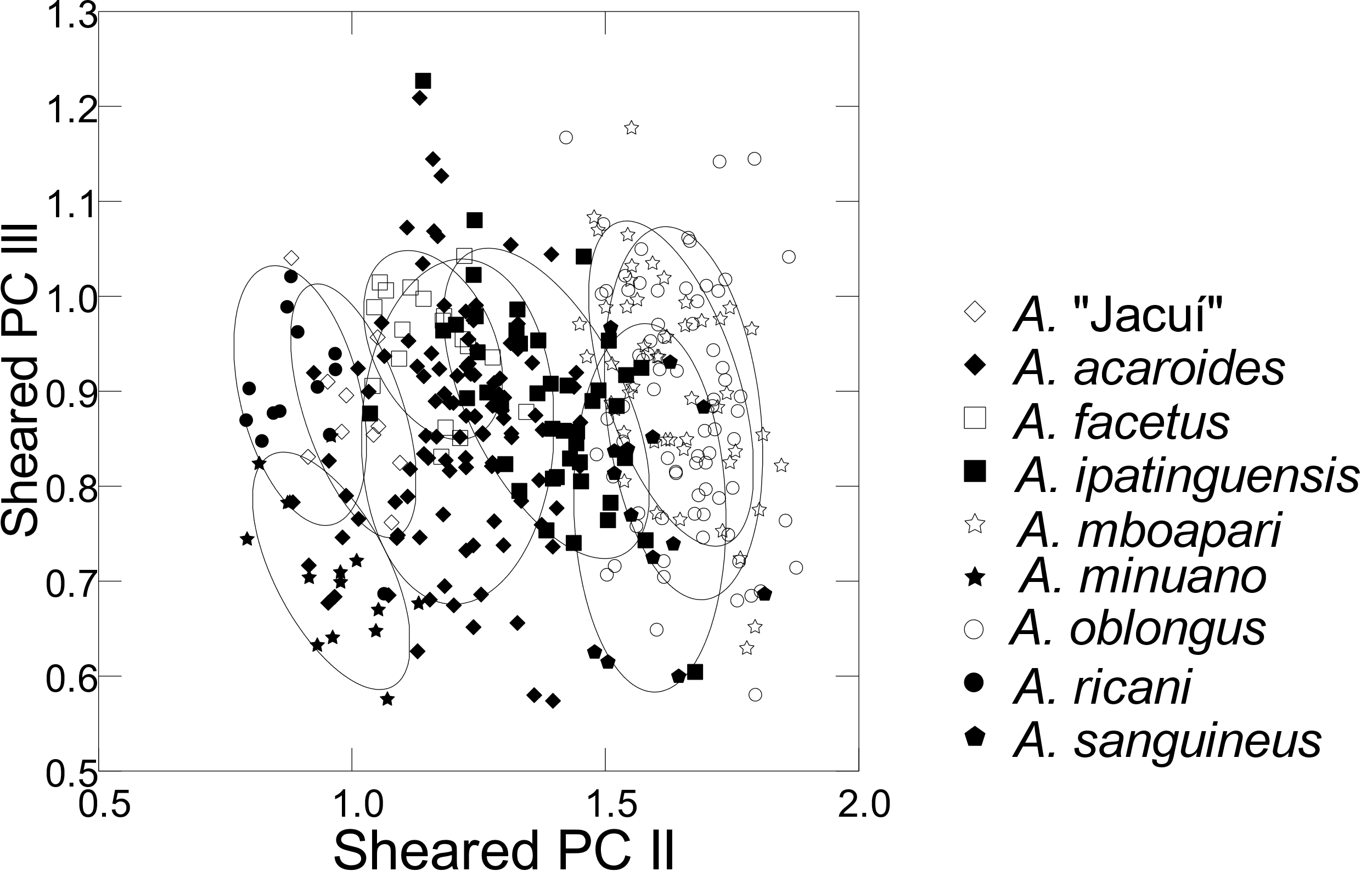


**Figure S4.1.** Plot of specimen scores of PC III on PC II from PCA of 15 distance measurements from pooled coastal species of *Australoheros*, and *A*. “Jacuí.” (Table S4.1).

**Table S4.1.** Character loadings from PCA of 15 distance measurements from pooled specimens of coastal species of *Australoheros (A. acaroides¸A. facetus, A.* *ipatinguensis, A. mboapari, A. minuano, A. oblongus, A. ricani, A. sanguineus, A.* sp. “Jacui”) (Fig. S4.1).

| Component | PC I | PC II | Sheared PC II | PC III | Sheared PC III | PC IV | Sheared PC IV |
| --- | --- | --- | --- | --- | --- | --- | --- |
| SL | 0.241 | -0.077 | -0.086 | -0.017 | -0.018 | 0.019 | 0.018 |
| Head length | 0.225 | 0.02 | 0.011 | -0.005 | -0.006 | 0.028 | 0.028 |
| Snout length | 0.297 | -0.198 | -0.209 | -0.461 | -0.462 | -0.068 | -0.068 |
| Body depth | 0.253 | 0.075 | 0.065 | -0.019 | -0.02 | 0.299 | 0.299 |
| Orbital diameter | 0.162 | 0.156 | 0.149 | 0.206 | 0.205 | 0.073 | 0.073 |
| Head width | 0.242 | 0.076 | 0.067 | 0.031 | 0.03 | 0.122 | 0.121 |
| Interorbital width | 0.28 | 0.142 | 0.132 | -0.047 | -0.048 | 0.175 | 0.175 |
| Preorbital depth | 0.346 | **-0.379** | **-0.391** | **-0.502** | **-0.504** | -0.115 | -0.116 |
| Upper jaw length | 0.27 | 0.05 | 0.04 | 0.027 | 0.026 | -0.095 | -0.096 |
| Lower jaw length | 0.239 | 0.109 | 0.1 | 0.093 | 0.092 | -0.015 | -0.016 |
| Caudal peduncle length | 0.252 | 0.087 | 0.078 | -0.064 | -0.065 | 0.188 | 0.188 |
| Caudal peduncle depth | 0.267 | **-0.674** | **-0.683** | **0.639** | **0.638** | **-0.133** | **-0.133** |
| P length | 0.242 | 0.17 | **0.161** | **0.15** | **0.148** | 0.067 | 0.066 |
| V length | 0.278 | **0.436** | **0.425** | 0.103 | 0.102 | -0.774 | -0.774 |
| Last D spine length | 0.236 | 0.245 | 0.236 | 0.184 | 0.183 | **0.419** | **0.418** |
| Eigenvalue | 1.8411 | 0.0693 | N/A | 0.0143 | N/A | 0.0078 | N/A |
| Variance | 94.00% | 97.50% | N/A | 98.30% | N/A | 98.60% | N/A |


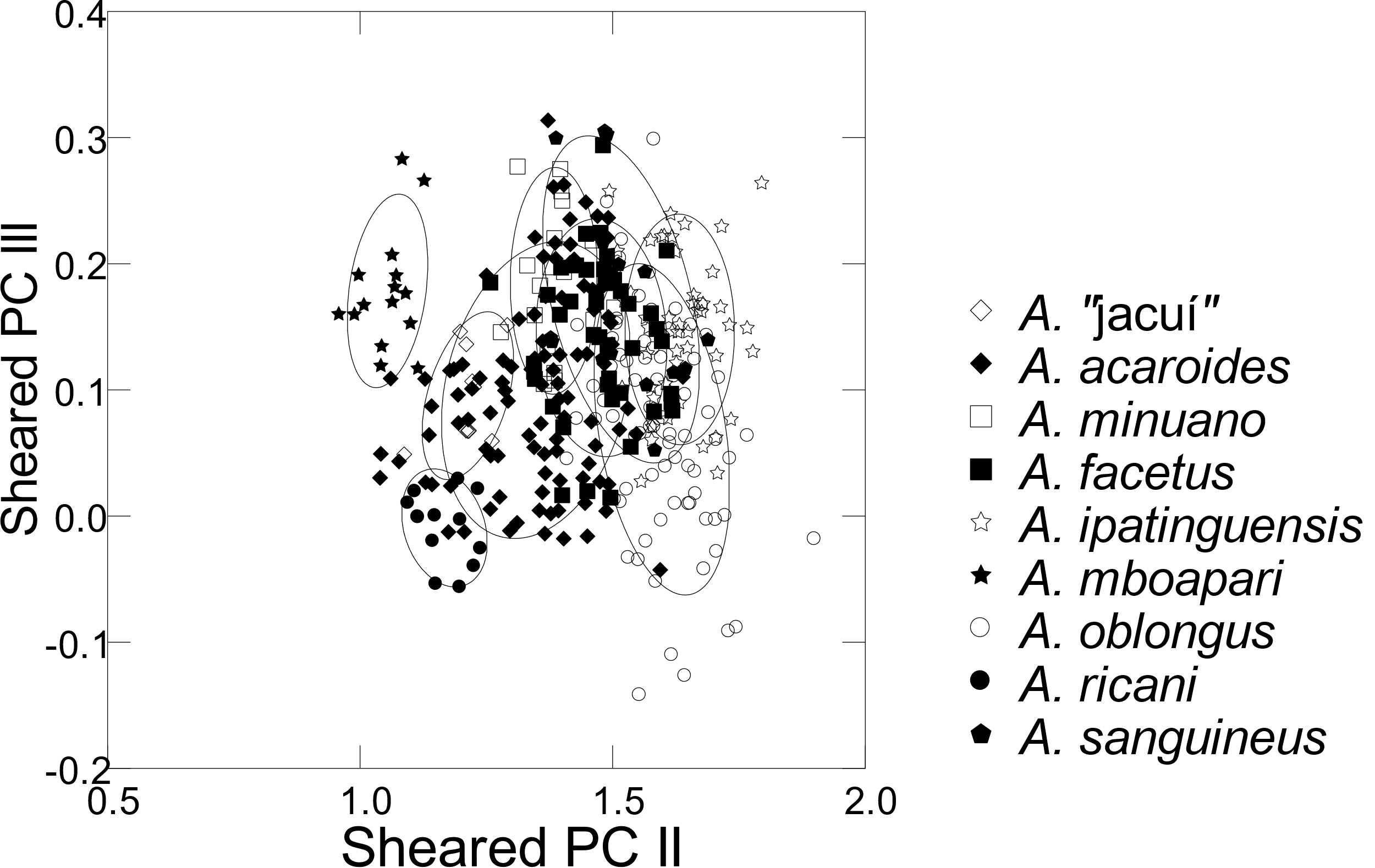


**Figure S4.2 .** Plot of scores of PC II on PC III from PCA of 13 distance measurements from pooled coastal species of *Australoheros*, and *A*. “Jacuí.” (Table S4.2).

Table S4.2**.** Character loadings from PCA of 13 distance measurements from pooled specimens of coastal species of *Australoheros (A. acaroides¸A. facetus, A.* *ipatinguensis, A. mboapari, A. minuano, A. oblongus, A. ricani, A. sanguineus, A.* sp. “Jacui”) (Fig. S4.2).

| Component | PC I | PC II | Sheared PC II | PC III | Sheared PC III | PC IV | Sheared PC IV |
| --- | --- | --- | --- | --- | --- | --- | --- |
| SL | 0.26 | -0.113 | -0.12 | -0.008 | -0.008 | 0.02 | 0.02 |
| Head length | 0.243 | 0.018 | 0.011 | -0.145 | -0.146 | 0 | 0 |
| Snout length | 0.321 | **-0.406** | **-0.415** | -0.128 | -0.129 | **-0.405** | **-0.405** |
| Body depth | 0.275 | 0.101 | 0.094 | **0.356** | **0.355** | 0.069 | 0.069 |
| Orbital diameter | 0.176 | 0.263 | 0.258 | -0.228 | -0.228 | -0.007 | -0.007 |
| Head width | 0.262 | 0.107 | 0.099 | 0.058 | 0.057 | 0.218 | 0.218 |
| Interorbital width | 0.303 | 0.172 | 0.163 | 0.308 | 0.307 | **0.547** | **0.547** |
| Preorbital depth | 0.375 | **-0.668** | **-0.677** | 0.136 | 0.135 | 0.022 | 0.022 |
| Upper jaw length | 0.292 | 0.058 | 0.05 | **-0.542** | **-0.542** | 0.216 | 0.216 |
| Lower jaw length | 0.259 | 0.16 | 0.152 | **-0.474** | **-0.475** | 0.054 | 0.054 |
| Caudal peduncle depth | 0.274 | 0.096 | 0.088 | 0.324 | 0.323 | 0.039 | 0.039 |
| P length | 0.262 | 0.257 | 0.25 | -0.002 | -0.003 | -0.192 | -0.192 |
| Last D spine length | 0.256 | **0.39** | **0.382** | 0.21 | 0.21 | **-0.629** | **-0.629** |
| Eigenvalue | 1.5631 | 0.0322 | NA | 0.0068 | N/A | 0.0045 | N/A |
| Variance | 96.30% | 98.30% | N/A | 98.70% | N/A | 99.00% | N/A |


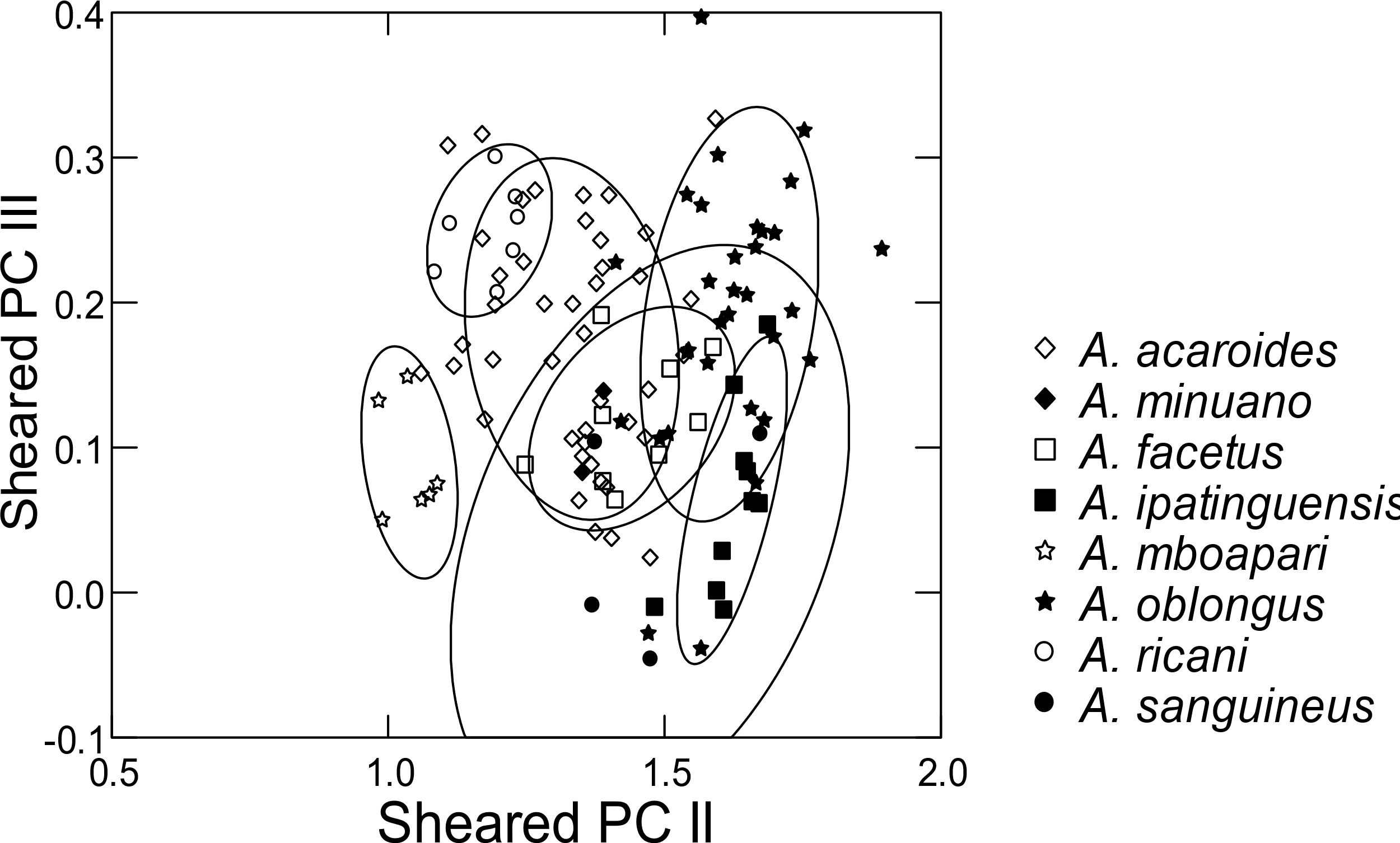


**Figure S4.3.** Plot of scores of PC III on PC II from PCA of 13 distance measurements from pooled coastal species of *Australoheros A. acaroides, A. canterai*, *A. facetus, A. ipatinguensis, A. mbapoari, A. oblongus, A. ricani, A. sanguineus*, restricted to specimens 60–80 mm SL. (Table S4.3).

**Table S4.3.** Character loadings from PCA of 13 distance measurements from pooled specimens 60-80mm SL of coastal species of *Australoheros (A. acaroides*, *A. facetus, A. ipatinguensis, A. mboapari, A. minuano, A. oblongus, A. ricani, A. sanguineus. (*Fig. 20); *A.* sp. “Jacuí” out of range and not included).

| Component | PC I | PC II | Sheared PC II | PC III | Sheared PC III | PC IV | Sheared PC IV |
| --- | --- | --- | --- | --- | --- | --- | --- |
| SL | 0.225 | -0.097 | -0.099 | 0.084 | 0.085 | -0.042 | -0.036 |
| Head length | 0.24 | 0.022 | 0.02 | 0.164 | 0.165 | -0.026 | -0.02 |
| Snout length | 0.302 | **-0.413** | **-0.415** | 0.084 | 0.085 | -0.098 | -0.09 |
| Body depth | 0.282 | 0.074 | 0.072 | -0.303 | -0.302 | 0.108 | 0.116 |
| Orbital diameter | 0.189 | 0.287 | 0.286 | 0.226 | 0.227 | -0.112 | -0.107 |
| Head width | 0.289 | 0.115 | 0.113 | -0.007 | -0.006 | 0.168 | 0.176 |
| Interorbital width | 0.338 | 0.162 | 0.16 | -0.149 | -0.148 | **0.696** | **0.704** |
| Preorbital depth | 0.365 | **-0.689** | **-0.691** | -0.143 | -0.141 | -0.168 | -0.158 |
| Upper jaw length | 0.28 | 0.074 | 0.072 | **0.541** | **0.543** | 0.024 | 0.032 |
| Lower jaw length | 0.255 | 0.124 | 0.122 | **0.492** | **0.493** | -0.056 | -0.049 |
| Caudal peduncle depth | 0.275 | 0.058 | 0.057 | -0.284 | -0.282 | 0.178 | 0.185 |
| P length | 0.25 | 0.299 | 0.297 | -0.138 | -0.137 | **-0.458** | **-0.451** |
| Last D spine length | 0.268 | **0.322** | **0.321** | **-0.373** | **-0.371** | **-0.422** | **-0.414** |
| Eigenvalue | 0.1243 | 0.0399 | NA | 0.0081 | NA | 0.0053 | NA |
| Variance | 65.20% | 86.20% | NA | 90.40% | NA | 93.20% | NA |


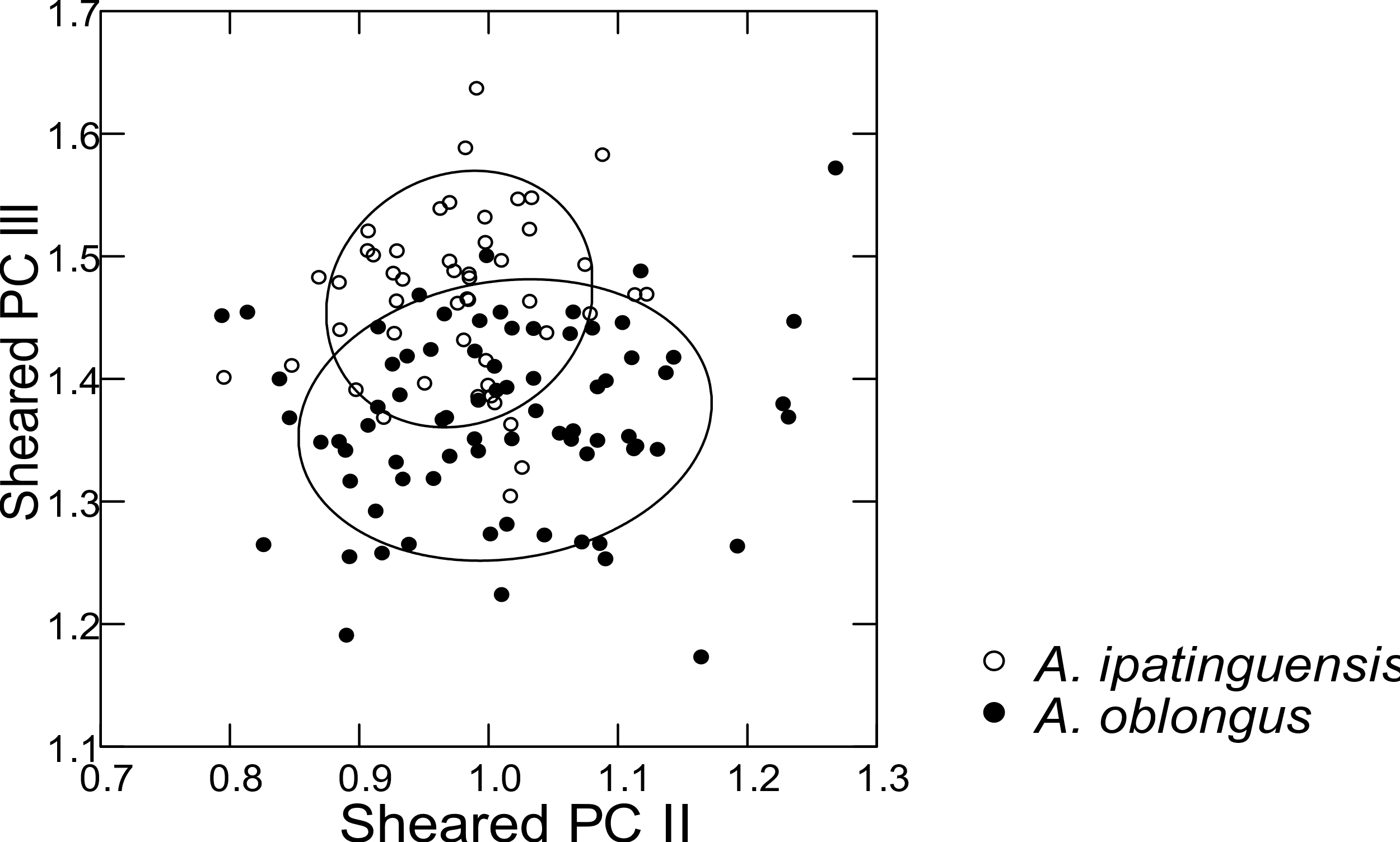


**Figure S4.4.** Plot of scores of PC II on PC III from PCA of 13 distance measurements from pooled samples of  *A. ipatinguensis* and *A. oblongus* (Table S4.4).

**Table S4.4** Character loadings from PCA of 13 distance measurements from pooled specimens of *Australoheros ipatinguensis* and *A. oblongus.* (Fig. 21).

| Component | PC I |  | PC II | Sheared PC II | PC III | Sheared PC III | PC IV | Sheared PC IV |
| --- | --- | --- | --- | --- | --- | --- | --- | --- |
| SL | 0.259 |  | 0.009 | 0.011 | -0.031 | -0.034 | 0.032 | 0.033 |
| Head length | 0.243 |  | 0.102 | 0.103 | -0.076 | -0.08 | 0.065 | 0.066 |
| Snout length | 0.296 |  | -0.318 | -0.317 | -0.324 | -0.328 | **0.705** | **0.705** |
| Body depth | 0.273 |  | -0.168 | -0.167 | 0.325 | 0.321 | 0.167 | 0.167 |
| Orbital diameter | 0.182 |  | **0.471** | **0.472** | 0.069 | 0.066 | -0.109 | -0.109 |
| Head width | 0.271 |  | 0.119 | 0.12 | 0.023 | 0.02 | -0.07 | -0.07 |
| Interorbital width | 0.307 |  | -0.159 | -0.158 | -0.108 | -0.112 | -0.289 | -0.288 |
| Preorbital depth | 0.355 |  | **-0.388** | -0.386 | -0.327 | -0.332 | **-0.592** | **-0.591** |
| Upper jaw length | 0.298 |  | 0.386 | **0.387** | **-0.357** | **-0.36** | 0.075 | 0.075 |
| Lower jaw length | 0.267 |  | **0.406** | **0.407** | -0.1 | -0.103 | 0.066 | 0.066 |
| Caudal peduncle depth | 0.275 |  | -0.334 | -0.332 | 0.263 | 0.26 | **0.086** | **0.086** |
| P length | 0.276 |  | 0.151 | 0.152 | 0.177 | 0.173 | 0.027 | 0.027 |
| Last D spine length | 0.271 |  | 0.03 | 0.031 | **0.649** | **0.645** | -0.046 | -0.045 |
| Eigenvalue | 1.1072 |  | 0.0085 | N/A | 0.0073 | N/A | 0.0035 | N/A |
| Variance | 97.30% |  | 98.00% | N/A | 98.70% | N/A | 99.00% | N/A |


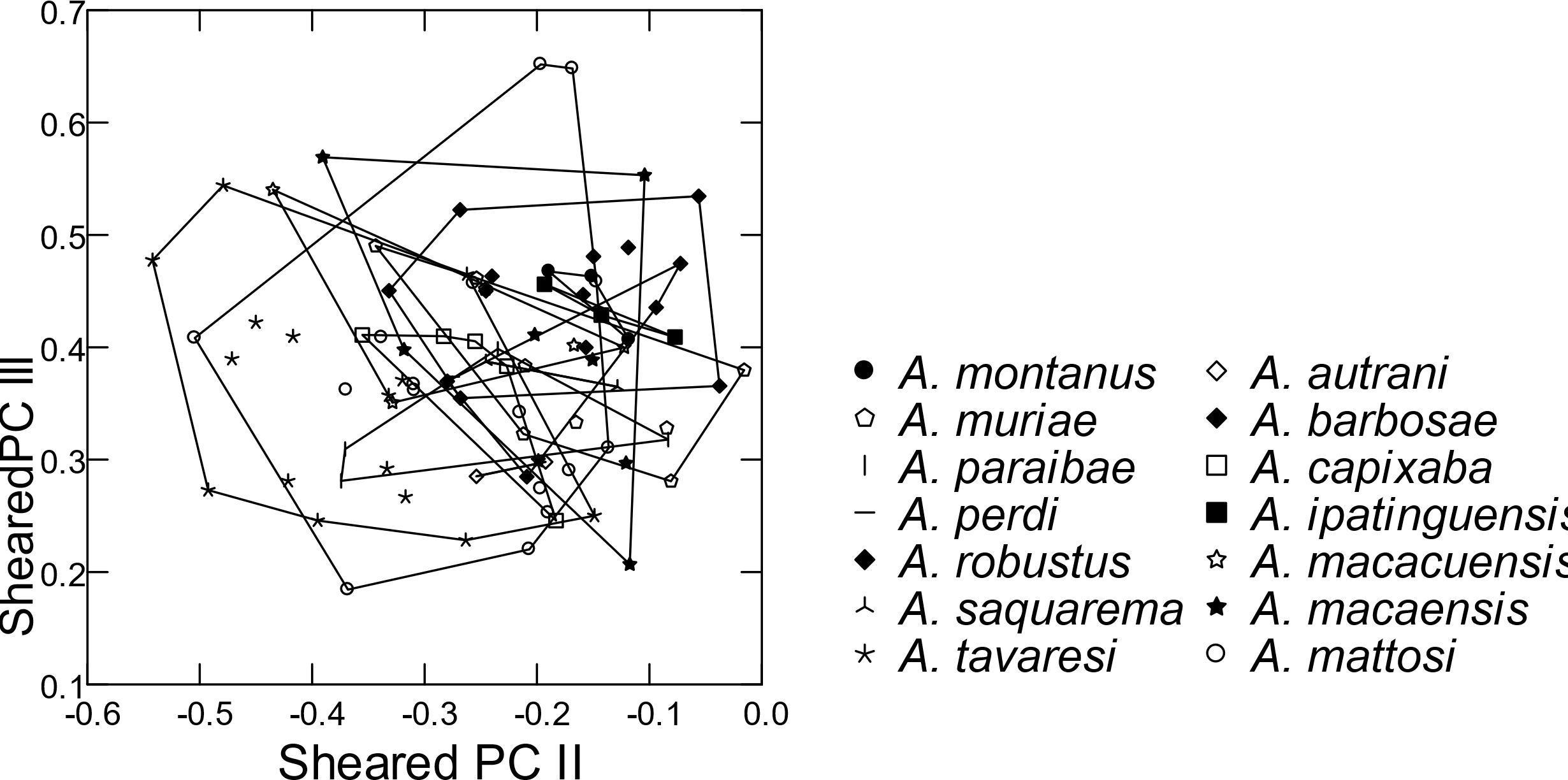


**Figure S4.5.** Scatterplot of specimen scores from PCA of distance measurements from pooled specimens assigned to nominal species of *Australoheros* from the Sudeste region. (Table S4.5).

**Table S4.5.** Character loadings from PCA of 15 distance measurements from pooled specimens of *Australoheros ipatinguensis* and *A. oblongus* assigned to nominal species based on locality and/or type status (*A. autrani*, *A. barbosae, A. capixaba, A. ipatinguensis, A. macacuensis, A. macaensis, A. mattosi, A. muriae, A. paraibae, A. perdi, A. robustus, A. saquarema, A. tavaresi).*(Fig. S4.5).

|  | PCI | PC II | Sheared PC II | PC III | Sheared PC 3 | PC IV | Sheared PC IV |
| --- | --- | --- | --- | --- | --- | --- | --- |
| SL | 0.245 | 0.007 | 0.003 | -0.146 | -0.147 | 0.031 | 0.03 |
| Head length | 0.227 | -0.098 | -0.102 | -0.02 | -0.02 | 0.164 | 0.163 |
| Snout length | 0.277 | **-0.354** | **-0.359** | **-0.301** | **-0.302** | -0.073 | -0.074 |
| Body depth | 0.26 | -0.081 | -0.085 | -0.107 | -0.108 | 0.121 | 0.12 |
| Orbital diameter | 0.173 | 0.107 | 0.104 | 0.174 | 0.174 | 0.5 | 0.5 |
| Head width | 0.257 | -0.041 | -0.046 | 0.066 | 0.065 | 0.177 | 0.176 |
| Interorbital width | 0.291 | -0.141 | -0.146 | -0.059 | -0.06 | -0.219 | -0.221 |
| Preorbital depth | 0.333 | **-0.251** | **-0.257** | **-0.135** | -0.136 | **-0.423** | **-0.424** |
| Upper jaw length | 0.278 | 0.004 | -0.001 | -0.03 | -0.031 | 0.243 | 0.242 |
| Lower jaw length | 0.25 | 0.103 | 0.098 | 0.035 | 0.034 | **0.383** | **0.382** |
| Caudal peduncle depth | 0.262 | -0.07 | -0.075 | **-0.194** | **-0.194** | -0.112 | -0.113 |
| Caudal peduncle length | 0.242 | **0.859** | **0.855** | -0.304 | **-0.305** | -0.248 | -0.249 |
| P length | 0.262 | 0.041 | 0.036 | 0.028 | 0.027 | 0.224 | 0.223 |
| V length | 0.342 | 0.083 | 0.077 | **0.829** | **0.828** | -0.337 | -0.339 |
| Last D spine length | 1.3454 | 0.0135 | NA | 0.009 | NA | 0.0079 | N/A |
| Variance | 96.70% | 97.70% | N/A | 98.30% | N/A | 98.90% | N/A |


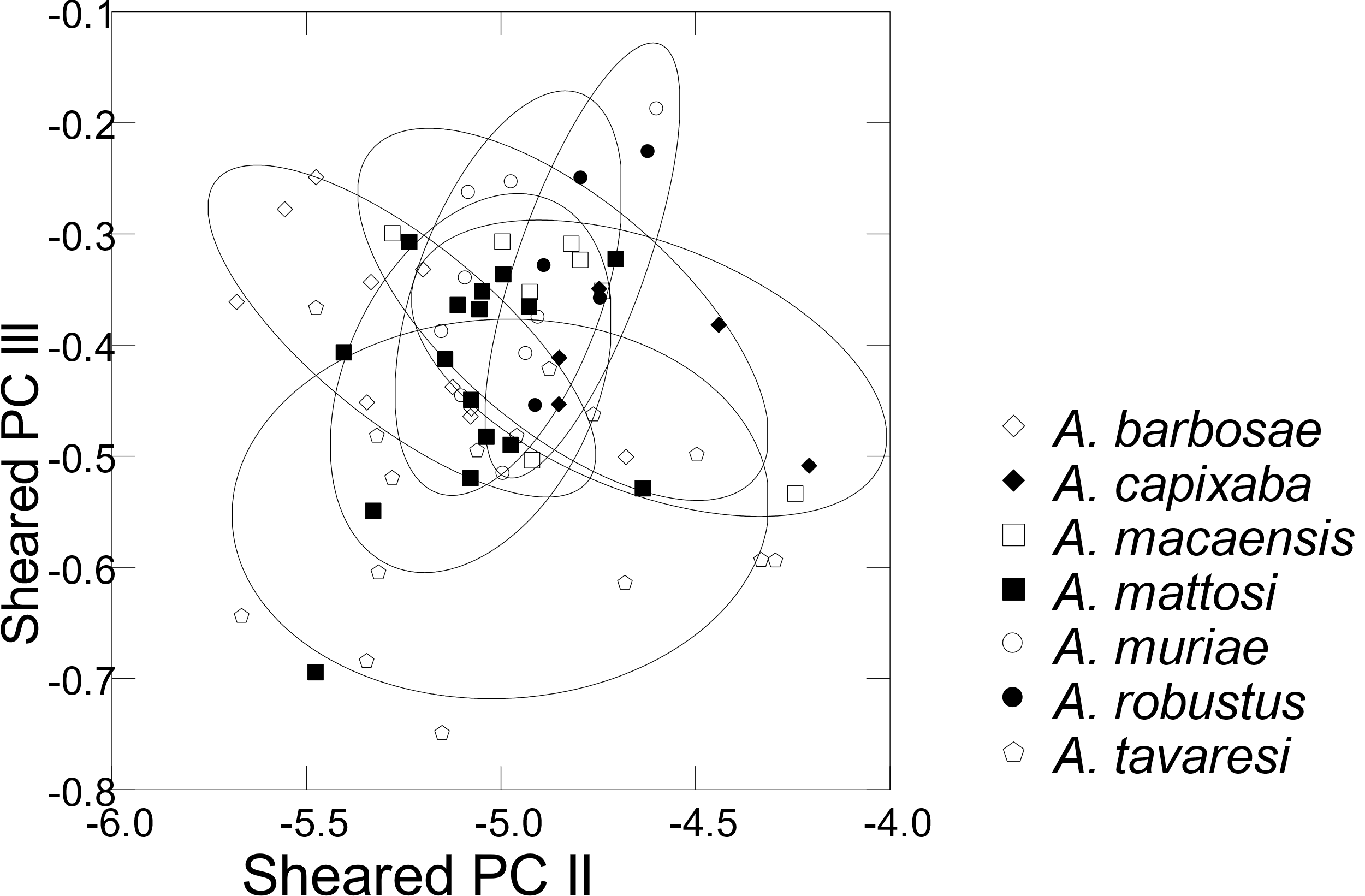


**Figure S4.6** Plot of scores of P C III on PC II from PCA of 15 distance measurements of northern species of *Australoheros,* assigned to nominal species based on locality and/or species status, represented by minimum five specimens. (Table S4.6).

**Table S4.6** Character loadings from PCA of distance measurements from pooled specimens of *Australoheros ipatinguensis* and *A. oblongus* assigned to nominal species based on locality and/or type status and represented by minimum five specimens (*A. barbosae, A. capixaba, A. macaensis, A. mattosi, A. muriae, A. robustus, A. tavaresi)* (Fig. S4.6).

|  | PCI | PC II | Sheared PC II | PC III | Sheared PC 3 | PC IV | Sheared PC IV |
| --- | --- | --- | --- | --- | --- | --- | --- |
| SL | 0.066 | -0.245 | -0.245 | -0.002 | -0.003 | -0.144 | -0.144 |
| Head length | 0.062 | -0.189 | -0.189 | -0.094 | -0.094 | -0.027 | -0.027 |
| Snout length | 0.076 | -0.241 | -0.241 | **-0.331** | **-0.331** | **-0.349** | **-0.349** |
| Body depth | 0.072 | -0.207 | -0.207 | -0.082 | -0.082 | -0.144 | -0.144 |
| Orbital diameter | 0.048 | -0.106 | -0.106 | 0.12 | 0.12 | 0.165 | 0.165 |
| Head width | 0.07 | -0.202 | -0.202 | -0.04 | -0.04 | 0.059 | 0.059 |
| Interorbital width | 0.081 | -0.28 | -0.28 | -0.156 | **-0.156** | -0.003 | -0.003 |
| Preorbital depth | 0.09 | **-0.358** | **-0.359** | **-0.299** | **-0.3** | -0.132 | -0.132 |
| Upper jaw length | 0.076 | -0.271 | -0.272 | -0.034 | -0.035 | -0.092 | -0.092 |
| Lower jaw length | 0.068 | -0.202 | -0.202 | 0.094 | 0.094 | -0.007 | -0.007 |
| Caudal peduncle depth | 0.073 | -0.24 | -0.24 | -0.092 | -0.093 | **-0.192** | **-0.193** |
| Caudal peduncle length | 0.065 | **-0.329** | **-0.33** | **0.85** | **0.85** | -0.218 | -0.218 |
| P length | 0.072 | -0.211 | -0.211 | 0.046 | 0.046 | -0.006 | -0.006 |
| V length | 0.092 | **-0.384** | **-0.384** | -0.027 | -0.027 | **0.834** | **0.834** |
| Last D spine length | 0.962 | 0.269 | 0.266 | 0.022 | 0.017 | 0.007 | 0.006 |
| Eigenvalue | 17.4546 | 0.1048 | NA | 0.0136 | NA | 0.0102 | N/A |
| Variance | 99.10% | 99.70% | N/A | 99.80% | N/A | 99.90% | N/A |


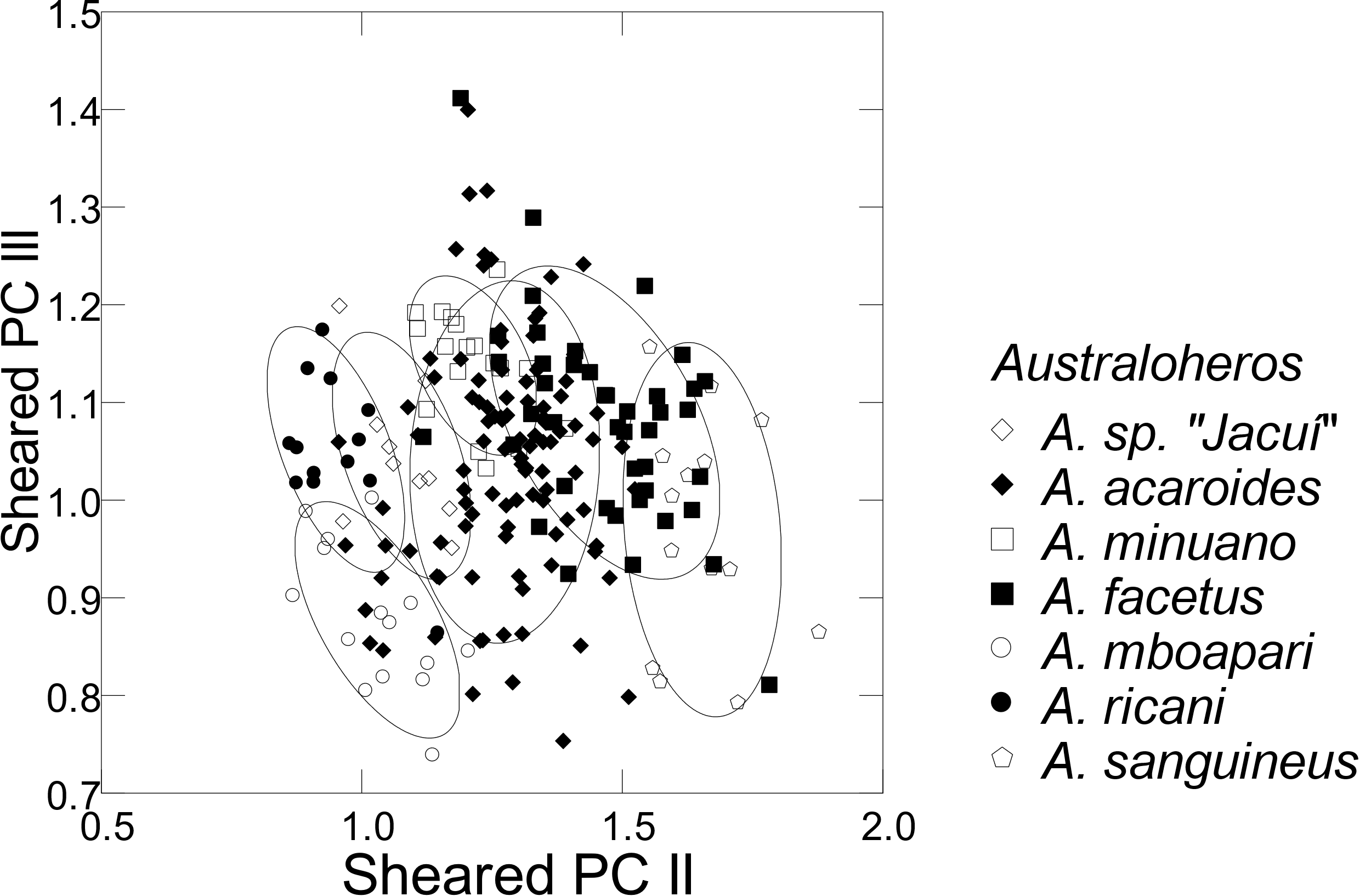


**Figure S4.7** Plot of scores of PC III on PC II from PCA of 15 distance measurements from pooled southern coastal species of *Australoheros* (*A. acaroides, A. minuano*, *A. facetus, A. mbapoari, A. ricani, A. sanguineus*) and *A.* sp. “Jacuí.” (Table S4.7).

**Table S4.7**. Character loadings from PCA of 15 distance measurements from pooled specimens of southern costal species of *Australoheros* (*A. acaroides*, *A.*

*facetus, A. mboapari, A. minuano, A. ricani, A. sanguineus, A.* sp. “Jacuí”; Fig. S4.7)

| Component | PC I | PC II | Sheared PC II | PC III | Sheared PC III | PC IV | Sheared PC IV |
| --- | --- | --- | --- | --- | --- | --- | --- |
| SL | 0.241 | -0.077 | -0.086 | -0.017 | -0.02 | 0.019 | 0.018 |
| Head length | 0.225 | 0.02 | 0.011 | -0.005 | -0.01 | 0.028 | 0.028 |
| Snout length | 0.297 | -0.198 | -0.209 | -0.461 | -0.46 | -0.068 | -0.068 |
| Body depth | 0.253 | 0.075 | 0.065 | -0.019 | -0.02 | 0.299 | 0.299 |
| Orbital diameter | 0.162 | 0.156 | 0.149 | 0.206 | 0.205 | 0.073 | 0.073 |
| Head width | 0.242 | 0.076 | 0.067 | 0.031 | 0.03 | 0.122 | 0.121 |
| Interorbital width | 0.28 | 0.142 | 0.132 | -0.047 | -0.05 | 0.175 | 0.175 |
| Preorbital depth | 0.346 | **-0.379** | **-0.391** | **-0.502** | **-0.5** | -0.115 | -0.116 |
| Upper jaw length | 0.27 | 0.05 | 0.04 | 0.027 | 0.026 | -0.095 | -0.096 |
| Lower jaw length | 0.239 | 0.109 | 0.1 | 0.093 | 0.092 | -0.015 | -0.016 |
| Caudal peduncle depth | 0.252 | 0.087 | 0.078 | -0.064 | -0.07 | 0.188 | 0.188 |
| Caudal-peduncle length | 0.267 | **-0.674** | **-0.683** | **0.639** | **0.638** | **-0.133** | **-0.133** |
| P length | 0.242 | 0.17 | **0.161** | **0.15** | **0.148** | 0.067 | 0.066 |
| V length | 0.278 | **0.436** | **0.425** | 0.103 | 0.102 | -0.774 | -0.774 |
| Last D spine length | 0.236 | 0.245 | 0.236 | 0.184 | 0.183 | **0.419** | **0.418** |
| Eigenvalue | 1.8411 | 0.0693 | N/A | 0.0143 | N/A | 0.0078 | N/A |
| Variance | 94.0% | 97.5% | N/A | 98.3% | N/A | 98.6% | N/A |


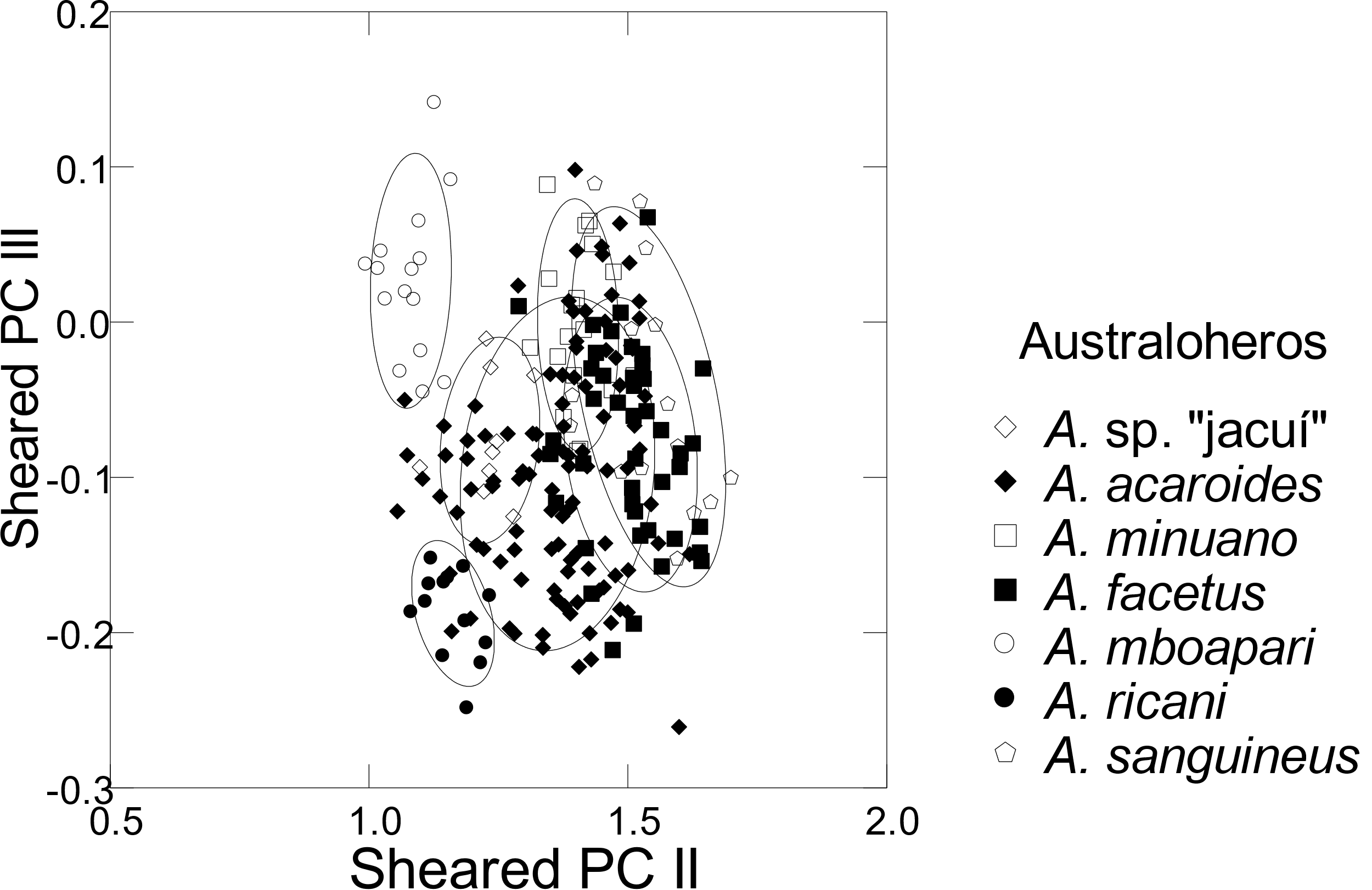


**Figure S4.8** Plot of scores of PCII on PC II from PCA of 13 distance measurements from pooled southern coastal species of *Australoheros* (*A. acaroides, A. minuano*, *A. facetus, A. mbapoari, A. ricani, A. sanguineus*) and *A.* sp. “Jacuí.” (Table S4.8).

**Table S4.8.** Character loadings from PCA of 13 distance measurements from pooled specimens of southern costal species of *Australoheros* (*A.* acaroides, *.A facetus, A. mboapari, A. minuano, A. ricani, A. sanguineus, A.* sp. “Jacuí”; Fig. S4.8).

| Component | PC I | PC II | Sheared PC II | PC III | Sheared PC III | PC IV | Sheared PC IV |
| --- | --- | --- | --- | --- | --- | --- | --- |
| SL | 0.257 | -0.119 | -0.12 | 0.026 | 0.025 | -0.043 | -0.042 |
| Head length | 0.244 | -0.002 | -0.003 | -0.148 | -0.15 | -0.014 | -0.013 |
| Snout length | 0.321 | **-0.502** | **-0.503** | -0.087 | -0.09 | -0.005 | -0.004 |
| Body depth | 0.276 | 0.184 | 0.183 | 0.29 | 0.289 | -0.169 | -0.169 |
| Orbital diameter | 0.184 | 0.182 | 0.181 | -0.106 | -0.11 | 0.096 | 0.096 |
| Head width | 0.261 | 0.151 | 0.15 | 0.103 | 0.102 | -0.142 | -0.141 |
| Interorbital width | 0.306 | 0.258 | 0.257 | **0.366** | **0.365** | **-0.399** | **-0.398** |
| Preorbital depth | 0.359 | **-0.624** | **-0.625** | 0.252 | 0.251 | 0.227 | 0.228 |
| Upper jaw length | 0.292 | 0.022 | 0.021 | **-0.548** | **-0.55** | -0.208 | -0.208 |
| Lower jaw length | 0.262 | 0.105 | 0.104 | **-0.567** | **-0.57** | -0.102 | -0.101 |
| Caudal peduncle depth | 0.277 | 0.11 | 0.109 | 0.204 | 0.203 | -0.253 | -0.253 |
| P length | 0.266 | 0.225 | 0.224 | 0.031 | 0.03 | **0.372** | **0.372** |
| Last D spine length | 0.264 | **0.337** | **0.336** | 0.044 | 0.043 | **0.688** | **0.688** |
| Eigenvalue | 1.6986 | 0.0246 | N/A | 0.0064 | N/A | 0.0048 | N/A |
| Variance | 97.00% | 98.40% | N/A | 98.80% | N/A | 99.00% | N/A |


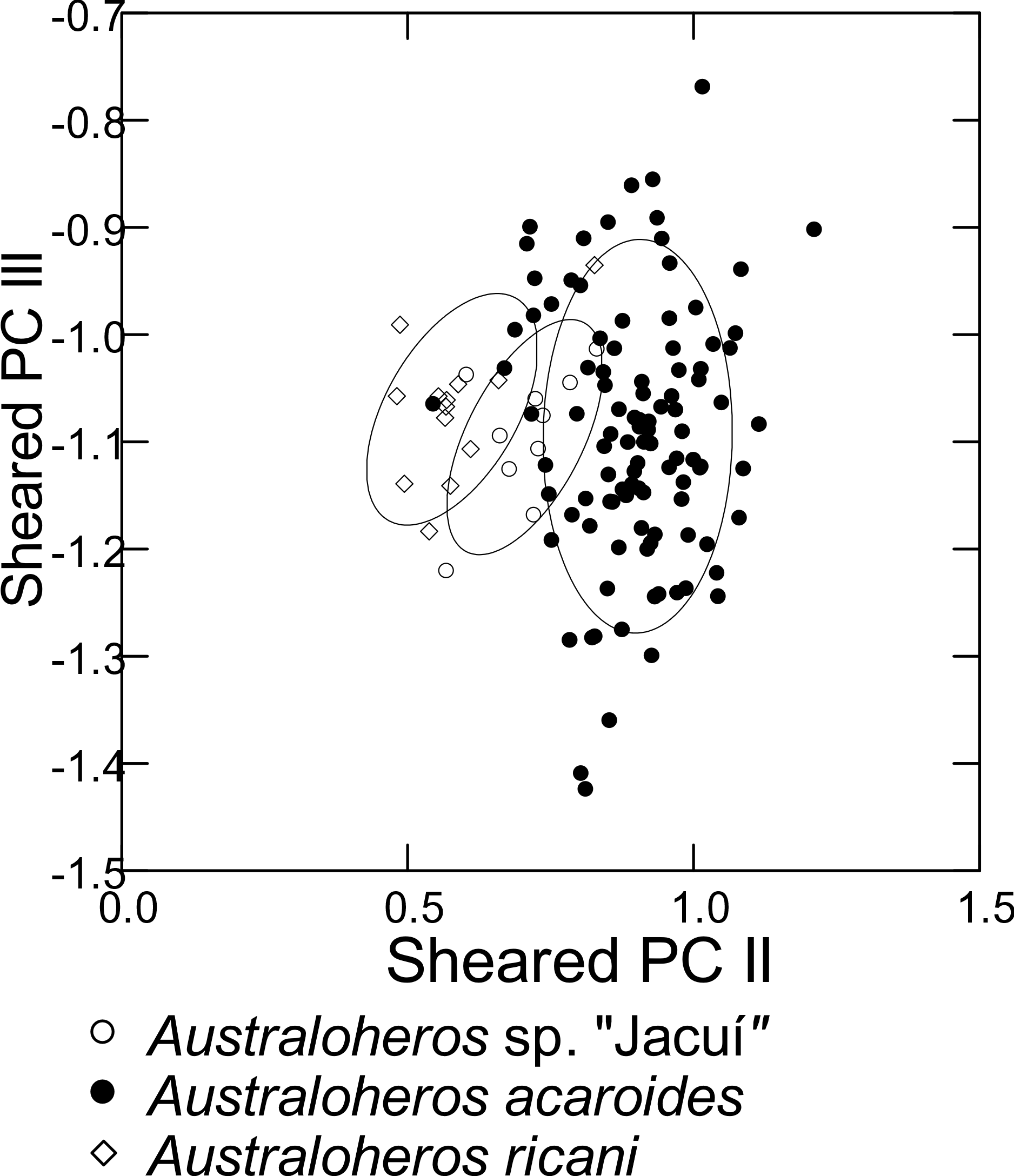


**Figure S4.9** Plot of scores of PC III on PC II from PCA of 13 distance measurements from pooled specimens of *Australoheros* from the Rio Jacuí drainage (*A. acaroides*, *A. facetus, A. mbapoari, A. ricani,* ) and *A.* sp. “Jacuí.” (Table S4.9).

**Table 4.9.** Character loadings from PCA of 13 distance measurements from pooled specimens of *Australoheros* from the Rio Jacuí drainage (*A. acaroides, A. ricani, A.* “Jacuí” ). (Fig. S4.9).

| Component | PC I | PC II | Sheared PC II | PC III | Sheared PC III | PC IV | Sheared PC IV |
| --- | --- | --- | --- | --- | --- | --- | --- |
| SL | 0.256 | -0.037 | -0.048 | 0.016 | 0.016 | 0.008 | 0.005 |
| Head length | 0.239 | 0.014 | 0.004 | -0.003 | -0.003 | 0.106 | 0.103 |
| Snout length | 0.326 | -0.17 | -0.184 | **0.595** | **0.595** | **0.342** | **0.337** |
| Body depth | 0.267 | **0.259** | **0.247** | -0.128 | -0.128 | -0.177 | -0.18 |
| Orbital diameter | 0.178 | 0.109 | 0.101 | -0.246 | -0.246 | 0.111 | 0.108 |
| Head width | 0.25 | 0.232 | 0.221 | -0.141 | -0.141 | -0.007 | -0.01 |
| Interorbital width | 0.302 | **0.483** | **0.469** | -0.152 | -0.153 | -0.16 | -0.164 |
| Preorbital depth | 0.373 | -0.213 | -0.228 | **0.448** | **0.448** | -0.641 | -0.646 |
| Upper jaw length | 0.289 | 0.004 | -0.008 | 0.045 | 0.045 | **0.452** | **0.448** |
| Lower jaw length | 0.249 | 0.055 | 0.045 | -0.014 | -0.014 | 0.408 | 0.405 |
| Caudal peduncle depth | 0.272 | 0.162 | 0.15 | 0.006 | 0.006 | -0.041 | -0.044 |
| P length | 0.307 | **-0.726** | **-0.738** | **-0.516** | **-0.516** | -0.031 | -0.035 |
| Last D spine length | 0.246 | 0.05 | 0.039 | -0.238 | -0.238 | -0.138 | -0.141 |
| Eigenvalue | 1.2652 | 0.02 | N/A | 0.0125 | N/A | 0.0051 | N/A |
| Cumulative variance | 96.00% | 97.50% | N/A | 98.50% | N/A | 98.90% | N/A |
