# Supplementary figures and images for: Conjectures and refutations: Species diversity and phylogeny of Australoheros from coastal rivers of southern South America (Teleostei: Cichlidae)
Source: PLoS One. 2022 Dec 9;17(12):e0261027. doi: 10.1371/journal.pone.0261027 (PMC9733902; doi:10.1371/journal.pone.0261027)

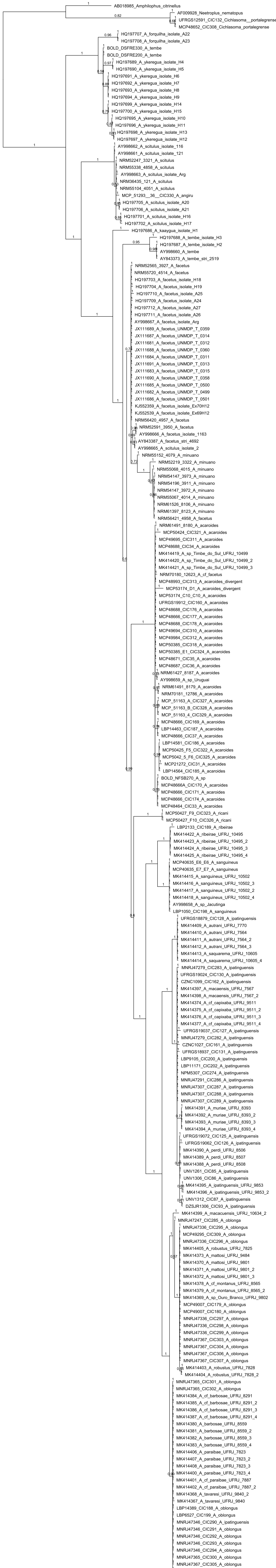

Supplement: S5 File — (PDF) [file pone.0261027.s005.pdf]
